# Mapping global prevalence of menopausal symptoms among middle-aged women: a systematic review and meta-analysis

**DOI:** 10.1186/s12889-024-19280-5

**Published:** 2024-07-02

**Authors:** Yiqiao Fang, Fen Liu, Xinyue Zhang, Lei Chen, Yang Liu, Lin Yang, Xiaofeng Zheng, Jiaye Liu, Kewei Li, Zhihui Li

**Affiliations:** 1 Division of Thyroid Surgery, Department of General Surgery, West China Hospital, Sichuan University, No.37 Guoxue Road, Chengdu, Sichuan 610041 China; 2 Laboratory of Thyroid and Parathyroid diseases, Frontiers Science Center for Disease-Related Molecular Network, West China Hospital, Sichuan University, Chengdu, Sichuan 610041 China; 3 Department of Respiratory and Critical Care Medicine, Frontiers Science Center for Disease-related Molecular Network, Center of Precision Medicine, Precision Medicine Key Laboratory of Sichuan Province, West China Hospital, Sichuan University, Chengdu, Sichuan 610041 China; 4 Department of Operating Room, West China School of Nursing, West China Hospital, Sichuan University, Chengdu, Sichuan 610041 China; 5 Department of Nuclear Medicine, West China Hospital, Sichuan University, Chengdu, Sichuan 610041 China; 6 Department of Obstetrics and Gynecology, The Second Affiliated Hospital, Chongqing Medical University, Chongqing, 400010 China; 7 Department of Gynecology, The First Affiliated Hospital, Chongqing Medical University, Chongqing, 400010 China; 8 Department of Endocrinology and Metabolism, Center for Diabetes and Metabolism Research, West China Hospital, Sichuan University, Chengdu, Sichuan 610041 China; 9 Department of Pediatric Department, West China Hospital, Sichuan University, No.37 Guoxue Road, Chengdu, Sichuan 610041 China

**Keywords:** Menopause, Prevalence, Middle-aged women, Somatic, Psychological, Urogenital

## Abstract

**Background:**

Women at middle age are puzzled by a series of menopausal disturbances, can be distressing and considerably affect the personal, social and work lives. We aim to estimate the global prevalence of nineteen menopausal symptoms among middle-aged women by performing a systematic review and meta-analysis.

**Methods:**

Comprehensive search was performed in multiple databases from January, 2000 to March, 2023 for relevant studies. Random-effect model with double-arcsine transformation was used for data analysis.

**Results:**

A total of 321 studies comprised of 482,067 middle-aged women were included for further analysis. We found varied prevalence of menopausal symptoms, with the highest prevalence of joint and muscular discomfort (65.43%, 95% CI 62.51–68.29) and lowest of formication (20.5%, 95% CI 13.44–28.60). Notably, South America shared dramatically high prevalence in a sort of menopausal symptoms including depression and urogenital symptoms. Besides, countries with high incomes (49.72%) had a significantly lower prevalence of hot flashes than those with low (65.93%), lower-middle (54.17%), and upper-middle (54.72%, *p* < 0.01), while personal factors, such as menopausal stage, had an influence on most menopausal symptoms, particularly in vaginal dryness. Prevalence of vagina dryness in postmenopausal women (44.81%) was 2-fold higher than in premenopausal women (21.16%, *p* < 0.01). Furthermore, a remarkable distinction was observed between body mass index (BMI) and prevalence of sleep problems, depression, anxiety and urinary problems.

**Conclusion:**

The prevalence of menopausal symptoms affected by both social and personal factors which calls for attention from general public.

**Supplementary Information:**

The online version contains supplementary material available at 10.1186/s12889-024-19280-5.

## Background

Female hormones play a pivotal role in women’s life. Their rise initiate puberty, makes motherhood possible, and ensure cardioprotective functions and bone health [1, 2]. However, regardless of their cultural background and medical histories, nearly all women start to have physical, psychological and emotional disturbances after mid-forties [3]. Those turmoil coincide with the loss of ovarian reproductive function, is an inevitable component of ageing and happens at a time in a woman’s life when she is frequently actively involved in raising her family or handling a full-time job, during which time she might also have the responsibility of caring for ageing parents [4]. The majority of women affected by marked fluctuations in levels of sex hormones are often puzzled by the remarkable changes in mood, sleep patterns, and memory, as well as the onset of vasomotor and urogenital symptoms [5]. These menopause-related symptoms, which actually begin before menstrual cycles ends and prevalent in middle-aged women, can be very distressing and considerably affect the personal, social and work lives of women [5, 6].

Nowadays, the relationship between psychosomatic symptoms and the women’s overall well-being is currently the focus of research across many fields, going from medical to social sciences. While epidemiological studies have provided a similar picture of menopausal symptoms trajectories in all geographical regions and ethnicities, there are significant differences in the prevalence of certain symptoms. For instance, vasomotor symptoms (VMS), characterized by hot flashes and/or night sweats, are the main symptoms of menopause. The US-based Study of Women’s Health Across the Nation (SWAN) reports that the prevalence of VMS is 50–82% among US women who go through natural menopause [7]. A radically lower prevalence, ranging from 36 to 50% in Norther America to 22–63% in Asia [8]. Likewise, disparities in the prevalence of depression in middle-aged women across different countries were noted. According to an Indian study, the prevalence of depression was approximately 40.0%, which is comparable to Brazil’s prevalence of 36.8% [9, 10]. Besides, depression is somewhat less common in the Chinese population with an estimate of 25.99% [11]. These differences might be explained by the fact that most cross-cultural studies only involved small numbers of participants and have mostly been restricted to one country or continent.

Over the past decade, data from epidemiological studies involving middle-aged women have been made available for investigators in the field of menopause. However, the current understanding of the epidemiology of menopause-related symptoms is based mostly on a few geographic surveys and very little national evidence, without rigorous systemic data that explores not only the general prevalence of menopause-related symptoms, but also risk factors associated with them. Besides, there is a paucity of articles to describe the global prevalence of menopausal symptoms from multiple domains, and most studies are limited to a certain symptom. For example, a meta-analysis of 10 studies conducted in Indian population showed that the prevalence of depression in perimenopausal and postmenopausal women was 42.47% [12] and another meta-analysis involving 41 studies found that the overall prevalence of sleep disorders among postmenopausal women was 51.6% [13]. Therefore, we performed current study aim to close this void by presenting an updated global epidemiology of nineteen menopause-related symptoms, providing subgroup analysis across geographic regions and synthesizing critical risk factors.

## Methods

We carried out a meta-analysis of all published studies on the prevalence of menopausal symptoms from January, 2000 through March, 2023 in accordance with the guidance of the Preferred Reporting Items for Systematic Reviews and Meta-Analyses (PRISMA). A total of nineteen menopause-related symptoms included in this study were classified into four domains: somatic symptoms (hot flashes, sleep problems, heart discomfort, headache, and joint and muscular discomfort), psychological symptoms (physical and mental exhaustion, depression, anxiety, irritability and mood swings), urogenital symptoms (sexual problems, vaginal dryness, and urinary problems) and others (forgetfulness, difficult concentration, formication, change in the appearance, texture, or tone of skin, increased facial hair, and drying skin). The study protocol was pre-registered in PROSPERO (CRD42023486818).

### Search strategy and selection criteria

A systematic literature search was conducted in Medline, Web of Science, Embase, Cochrane, and Google Scholar databases using the relevant medical subject heading search terms and keywords. Full details of the search strategy for each database can be found in the **Supplementary method**. Datasets from studies that fulfilled the following criteria were deemed eligible: (a) P: participants were middle-aged women in premenopausal, perimenopausal or postmenopausal stages according to the WHO’s classification; (b) O: Adequate information for the pooled estimate of menopausal symptoms prevalence; (c) O: prevalence of menopausal symptoms was determined using standardized instruments, self-reported questionnaires, face-to-face, telephone or mail interviews; (d) S: Cross-sectional, cohort, and case-control study designs; (e) studies in English; (f) studies published between 2000 and 2023. Studies were excluded if (a) P: participants seeking treatment for menopausal symptoms in hospitals; (b) S: studies were conference paper, abstract, letters, review or meta-analysis; (c) study size less than 50.

Pre-determined decision rules were used to screen studies. After removal of duplicate articles, two reviewers (Y.F and J.L) independently screened the titles and abstracts of all articles identified by the literature search, with 10% of studies randomly reviewed by another investigator (K.L). Then the investigators reviewed (Y.F and K.Z) the complete texts of theoretically qualifying papers, with any inconsistencies settled through agreement or by another reviewer (Z.L). Consensus was found in all cases and agreement was reached. More details refer to included articles are presented in the Supplementary materials.

### Quality assessment and data extraction

The methodological quality of epidemiological studies was assessed using a scale developed by Parker et al. [14]. with the following items: sampling methods; response rate; the definition and representative of targeted population and the validation of assessment instrument.

We extracted the following variables from included literature: the first author of the study, country, continent, income level of the country assessed by the World Bank, the status of country development, year of publication, study quality, diagnosis criteria, sample size and prevalence proportion. Moreover, a comparison was made of the prevalence of menopausal symptoms classified by menopausal status (premenopause, perimenopause or postmenopause), marital status (married or single/divorced/widowed), educational level (less than 12 years or more than 12 years), residence (urban or rural), physical activity (regular or irregular), employment (unemployed or employment), BMI (underweight, normal weight, overweight or obesity), current smoking (YES/NO), alcohol use (YES/NO). Menopausal status was defined in accordance to the WHO’s classification. To elucidate this distribution, women with regular menstrual bleeding during the last year were classified as premenopause, those with irregular bleeding during the last 12 months as perimenopause. Finally, women were classified as postmenopaused, if they had no menstrual bleeding from 1 year and above. Body mass index (BMI) was calculated as the actual weight, in kilograms, divided by height, in meters squared, relying on the anthropometric inputs (height, weight) measured respectively by a stadiometer and a digital scale, by the research team, the day of the recruitment. It was then categorized according to the WHO cut-off points: underweight if less than 18.5, normal if between 18.5 and 24.9, overweight if between 25 and 29.9 and obese from 30 and above [15]. When multiple articles of the same study population were identified, we included them if the data differed by time on prevalence of menopausal symptoms. Whenever important information was missing, we contacted corresponding authors.

### Statistical analysis

Meta-analysis was performed using R software (V4.0.0) with “Meta” and “Metafor” statistical packages. Heterogeneity across included studies was measured with I^2^. Estimates with I^2^ of 50% or greater was considered as moderate heterogeneity. The double-arcsine transformation was used for variance stabilization of proportions, and pooled estimates of the prevalence of menopausal symptoms in all studies were calculated using the random-effects approach, due to the heterogeneity. The meta-prop command was used to generate forest plots of pooled prevalence with 95% confidence intervals (CI) using the Wilson score method. Subgroup analyses were conducted and defined by geographical location, income level of the country, the status of country development, year of publication, study quality, diagnosis criteria, and sample size. Social characteristics of participants were compared with the prevalence of each menopausal symptoms to determine the pooled estimates of risk factors. To reduce the probability of committing a type I error due to the high number of subgroup comparisons, Bonferroni correction was used. The *p* value < 0.05 was considered as significant difference. For more details, the R code of this study has been added in the supplementary material.

### Certainty of evidence

The quality of pooled evidence was evaluated using the Grading of Recommendations, Assessment, Development and Evaluations (GRADE) framework.

## Results

### Search results and study characteristics

**Fig. 1:**
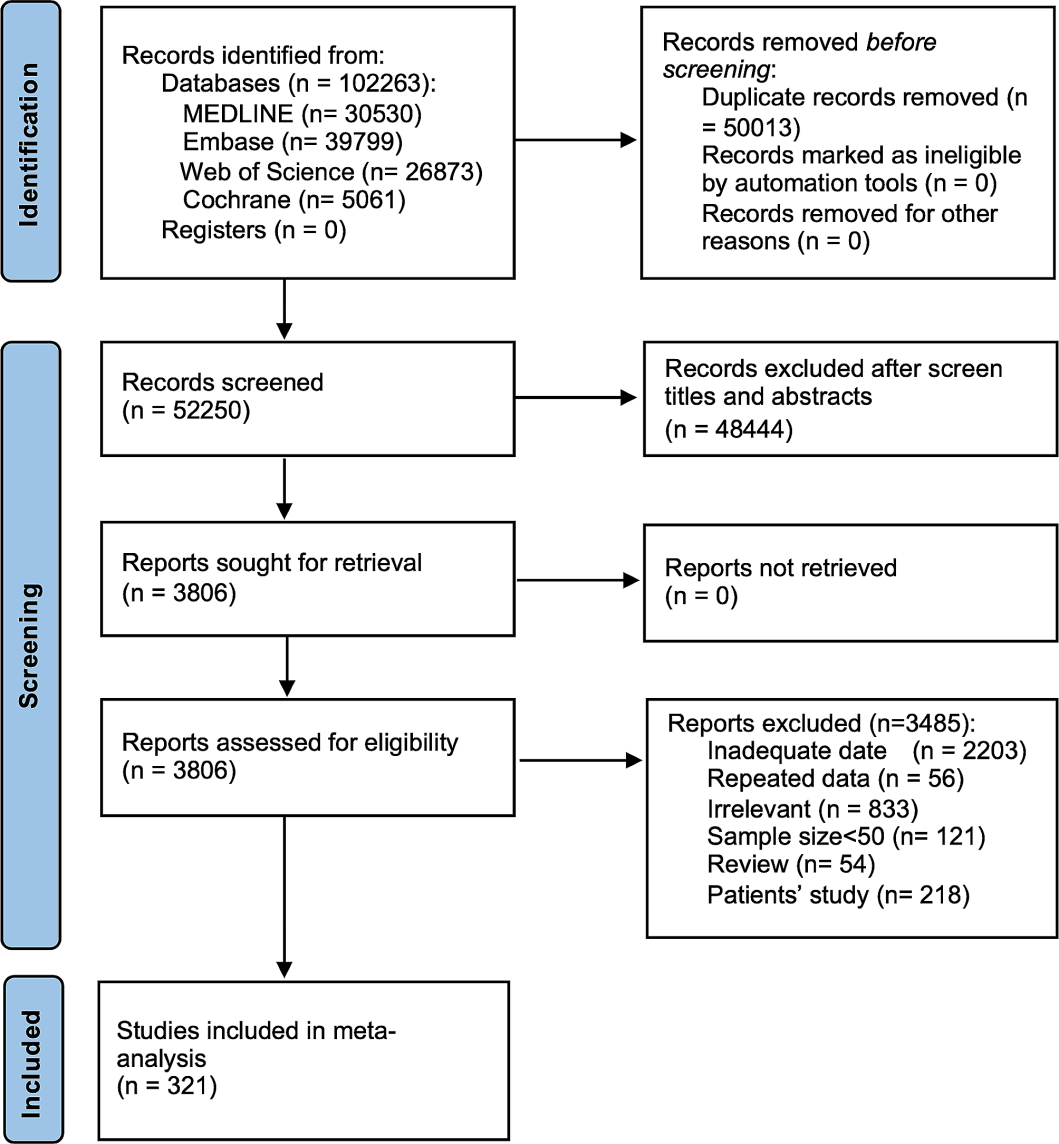
Study flow diagram

**Fig. 2:**
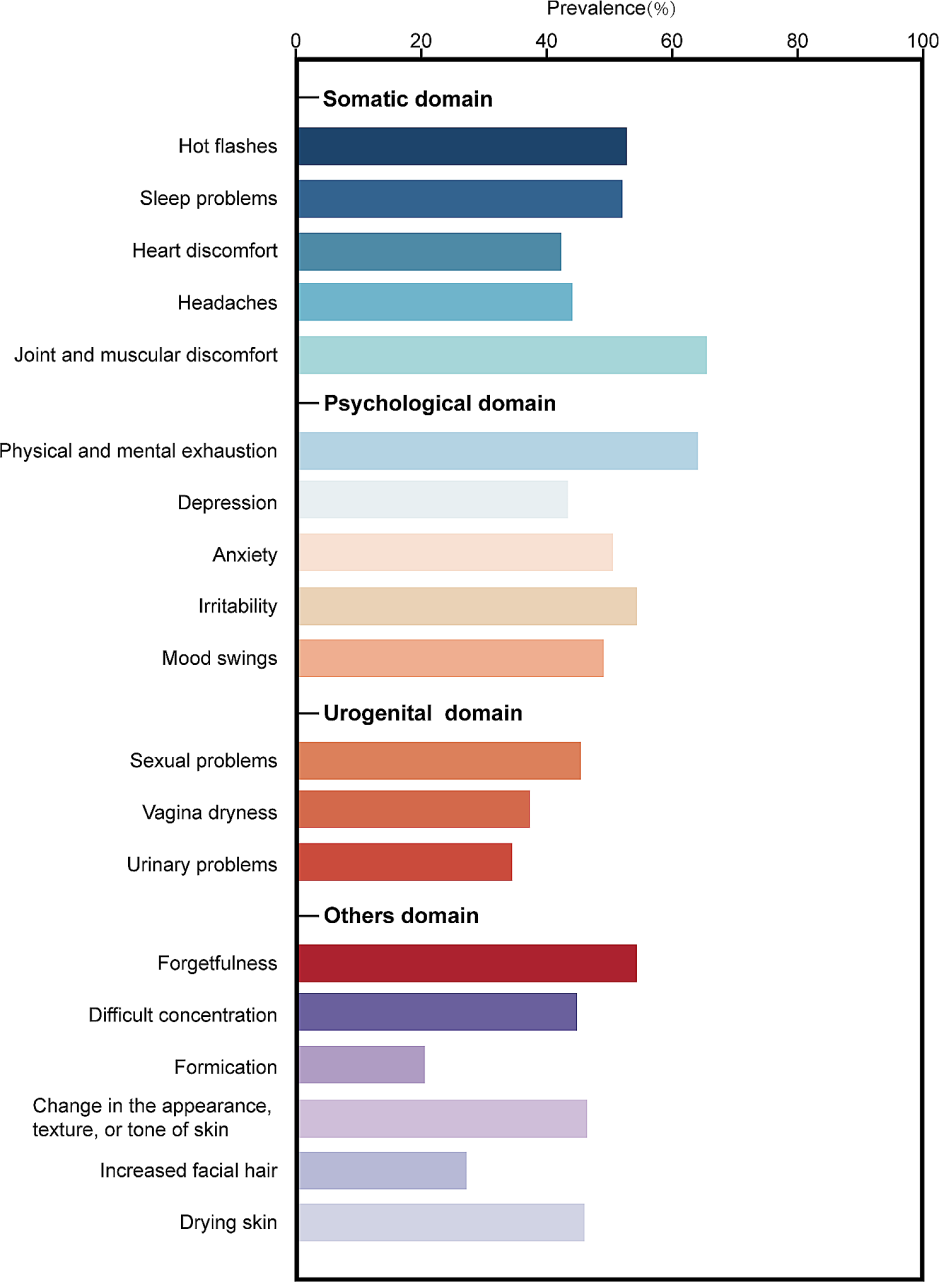
Pooled estimate prevalence of nineteen menopausal symptoms among middle-aged women

Our search strategy identified 102,263 records, of which 52,250 records were retained after removing duplicates. Titles and abstracts were screened, resulting in the exclusion of 48,444 ineligible records. Following an eligibility assessment of the full texts of the remaining 3,806 records, 3,485 were deemed ineligible. Overall, 321 eligible studies with data reporting menopausal symptoms involving 482,067 middle-aged women met our inclusion criteria and included in the final analysis (Fig. 1). Hot flashes were the symptom with the most articles featured which including 265 articles comprising 349,608 middle-aged females, formation had the fewest, with 16 articles containing 52,195 individuals. The majority of included studies had a cross-sectional design. The quality assessment scores of included studies are displayed in Supplementary Table 1. Furthermore, the pooled prevalence of nineteen symptoms is shown in Fig. 2.

### Pooled prevalence, subgroup analysis, and risk factors for somatic symptoms

In somatic domain, hot flashes were one of the most common menopausal symptoms with a pooled prevalence of 52.65% (95% CI 50.24–55.06, I^2^ = 99.51%, Supplementary Fig. 1). Different continents showed varying prevalence, and Africa had the highest prevalence (64.43%, 95% CI 56.78–71.73) while Oceania had the lowest prevalence (39.92%, 95% CI 30.56–49.66, *p* < 0.01, Table 1). Among countries containing at least three relevant studies, Egypt had the highest (72.56%, 95% CI 58.15–84.91) and Finland had the lowest (14.54%, 95% CI 5.82–26.29, *p* < 0.01, Table 1) hot flashes prevalence among middle-aged women. When taking into account the countries’ economic levels, those with high incomes had a significantly lower prevalence of 49.72% (95% CI 46.19–53.25) when compared to upper-middle (54.72%, 95% CI 50.08–59.31), lower-middle (54.17%, 95% CI 49.57–58.73) and low-income countries (65.93%, 95% CI 59.61–71.98, *p* < 0.01, Table 1). Furthermore, the hot flashes prevalence was substantially lower before 2011 (48.7%, 95% CI 44.78–52.63) than publications after 2011 (55.48%, 95% CI 52.51–58.43, *p* < 0.01, Table 1). In terms of diagnostic tool, the 10-item Cervantes Scale (CS-10) [16] produced the highest hot flashes prevalence (69.95%, 95% CI 53.7-83.96) while the Simplified Menopausal Index (SMI) [17] had the lowest prevalence (39.26%, 95% CI 27.45–51.74, Table 1). It should be mentioned that hot flashes in middle-aged women appeared to be universally prevalent in both developing countries (54.02%, 95% CI 50.75–57.27) and developed countries (50.39%, 95% CI 46.91–53.86, *p* = 0.14, Table 1). In order to find the risk factors of hot flashes, we pooled estimate relevant factors. Stratified by menopausal stage, we found middle-aged women in perimenopausal (56.52%, 95% CI 51.54–61.43) and postmenopausal stage (56.74%, 95% CI 52.8-60.64) with dramatically higher hot flashes prevalence than those in premenopausal stage (31.31%, 95% CI 26.46–36.38, *p* < 0.01, Table 1). However, minimal differences were observed among age (*p =* 0.69), physical activity (*p* = 0.82), body mass index (BMI, *p* = 0.86), residence (*p* = 0.39), current employment status (*p* = 0.65), current drinking habit (*p =* 0.76), current smoking habit (*p* = 0.48), marital status (*p =* 0.14) and education level (*p =* 0.71). Besides, we also pooled estimate prevalence of other somatic symptoms. The prevalence of sleep problems, heart discomfort, headaches, and joint and muscular discomfort were 51.89% (95% CI 49.55–54.22, I^2^ = 99.41%, Supplementary Figs. 2), 42.12% (95% CI 38.85–45.42, I^2^ = 99.46%, Supplementary Figs. 3), 43.91% (95% CI 40.64–47.21, I^2^ = 99.43%, Supplementary Figs. 4) and 65.43% (95% CI 65.51–68.29, I^2^ = 99.54%, Supplementary Fig. 5). The subgroup analysis and risk factor analysis for these somatic symptoms were listed in Supplementary Tables 2–5, respectively.

**Table 1:** Subgroup analysis and pooled estimates of risk factors for hot flashes prevalence among middle-aged women

Subgroup		Studies	Event	Total	Prevalence (%)	95% CI (%)	*P* value
**Country**							< 0.01
	China	32	31,022	82,951	43.39	36.78–50.11	
	Nepal	7	1810	4386	45.91	30.52–61.71	
	Nigeria	8	1790	3567	57.03	45.38–68.3	
	Ecuador	11	3132	5067	62.8	56.68–68.72	
	Spain	7	7214	14,049	50.45	42.07–58.81	
	Iran	11	5484	8474	68.35	54.6-80.64	
	India	41	6897	13,448	52	45.71–58.27	
	Ethiopia	1	149	226	65.93	59.61–71.98	
	Turkey	11	4411	6548	68.92	54.7-81.52	
	Saudi Arabia	8	1887	2771	66.54	57.46–75.05	
	Korea	9	6315	13,802	54.33	41.69–66.7	
	Taiwan	7	11,293	23,754	37.23	21.4-54.59	
	UK	7	8538	16,002	61.77	51.04–71.95	
	France	2	609	900	64.07	38.53–85.95	
	Germany	3	1878	2789	61.11	44.42–76.57	
	Belgium	1	487	594	81.99	78.79–84.98	
	Netherlands	3	2608	6014	57.3	35.52–77.7	
	Switzerland	2	594	901	61.98	34.56–85.8	
	Australia	14	5465	16,232	40.79	30.83–51.13	
	Japan	9	4904	9286	48.68	38.62–58.79	
	Oman	1	202	472	42.8	38.36–47.29	
	Multi	5	11,183	21,197	53.25	41.54–64.78	
	Macau	1	251	442	56.79	52.14–61.38	
	Peru	2	865	1002	92.19	71.02–100	
	Pakistan	8	3420	6267	40.23	24.15–57.45	
	Malaysia	7	771	1504	53.26	44.24–62.18	
	Sri Lanka	2	429	1033	42.43	35.49–49.53	
	Mexico	4	3354	9086	47.33	27.3-67.82	
	Brazil	5	1610	2878	50.08	41.09–59.07	
	USA	19	12,233	24,827	51.5	46.37–56.62	
	Lebanon	2	522	898	55.49	41.15–69.38	
	Singapore	2	192	1151	16.67	14.57–18.89	
	Greece	1	704	1025	68.68	65.81–71.49	
	Philippines	1	145	195	74.36	67.98–80.26	
	Indonesia	4	808	1622	37.23	10.68–68.85	
	Thailand	4	668	1080	59.9	53.16–66.46	
	Vietnam	1	100	100	100	98.29–100	
	Italy	2	544	1329	44.27	31.62–57.32	
	Sweden	2	3485	7017	57.14	40.74–72.77	
	Poland	1	226	349	64.76	59.66–69.69	
	Iraq	2	612	842	72.7	69.62–75.68	
	Finland	3	467	4003	14.54	5.82–26.29	
	Egypt	5	2507	3704	72.56	58.15–84.91	
	Bangladesh	4	578	1375	44.39	24.47–65.29	
	Qatar	1	431	1158	37.22	34.46–40.03	
	United Arab Emirates	1	129	390	33.08	28.49–37.83	
	Norway	1	8333	12,985	64.17	63.35-65	
	Cambodia	1	118	177	66.67	59.53–73.44	
	New Zealand	1	1030	3616	28.48	27.02–29.97	
	South Africa	1	46	63	73.02	61.3-83.34	
	Israel	1	208	612	33.99	30.28–37.79	
	Libya	1	64	86	74.42	64.61–83.14	
	Hong Kong	3	373	1433	33.79	6.18–69.72	
	Morocco	1	182	299	60.87	55.27–66.33	
	Panama	1	93	129	72.09	64.01–79.53	
	Chile	1	120	198	60.61	53.69–67.31	
	Portugal	1	251	728	34.48	31.07–37.97	
	Bolivia	1	58	125	46.4	37.7-55.21	
	Colombia	2	1279	1954	80.37	42.14–99.8	
	Paraguay	1	117	216	54.17	47.48–60.78	
	Jordan	2	93	280	27.73	0-88.34	
**Continent**							**< 0.01**
	Asia	183	84,073	186,451	50.99	47.67–54.3	
	Africa	17	4738	7945	64.43	56.78–71.73	
	South America	24	10,555	17,519	63.34	56.24–70.16	
	Europe	38	39,790	77,277	53.67	47.61–59.67	
	Oceania	15	6495	19,848	39.92	30.56–49.66	
	North America	24	15,680	34,042	51.62	46.2-57.03	
	Multi	2	3957	6526	59.94	31.08–85.46	
**Income level**							**< 0.01**
	Upper-Middle-Income	87	49,648	117,005	54.72	50.08–59.31	
	Lower-Middle-Income	97	24,848	45,670	54.17	49.57–58.73	
	High-Income	117	87,269	180,628	49.72	46.19–53.25	
	Low-Income	1	149	226	65.93	59.61–71.98	
**Development status**							0.14
	Developing	189	84,578	183,516	54.02	50.75–57.27	
	Developed	112	75,983	157,007	50.39	46.91–53.86	
**Publication date**							**< 0.01**
	Before 2011	127	51,295	118,568	48.7	44.78–52.63	
	After 2011	176	113,993	231,040	55.48	52.51–58.43	
**Study size**							**0.01**
	< 1000	223	42,756	79,780	54.45	51.58–57.31	
	> 1000	80	122,532	269,828	47.74	43.49–52.01	
**Study quality**							0.28
	< 8	42	29,871	75,903	53.21	50.6-55.82	
	≥ 8	261	135,417	273,705	49.44	43.19–55.71	
**Diagnostic tool**							**< 0.01**
	KMI [18]	25	30,173	75,754	42.43	36.31–48.67	
	MRS [19]	82	35,063	62,420	58.52	54.85–62.15	
	Others	73	40,151	78,506	54.77	49.38–60.1	
	Face-to-face interview	62	33,366	78,534	45.85	39.84–51.91	
	The Greene Climacteric Scale	17	6618	14,279	47.63	36.15–59.23	
	The Keio questionnaire [20]	3	2331	3420	61.78	39.22–81.95	
	SMI	2	920	2338	39.26	27.45–51.74	
	MENQOL [21]	28	8782	20,224	54.4	47.14–61.58	
	Hot Flush Rating Scale [22]	7	6238	11,569	56.1	47.11–64.89	
	CS-10	2	1427	2190	69.95	53.7-83.96	
	WHAS [23]	2	219	374	61.17	31.27–87.09	
**Risk factors**		**Studies**	**Event**	**Total**	**Prevalence (%)**	**95% CI (%)**	**P value**
**Menopausal stage**							**< 0.01**
	Premenopause	68	12,966	50,939	31.31	26.46–36.38	
	Perimenopause	75	18,525	37,720	56.52	51.54–61.43	
	Postmenopause	115	47,621	89,453	56.74	52.8-60.64	
**Age**							0.69
	< 50	13	4561	14,554	49.61	34.17–65.09	
	≥ 50	28	11,331	20,805	53.22	44.84–61.51	
**Physical activity**							0.82
	Regular	6	1097	2138	48.95	39.28–58.65	
	Irregular	5	950	1914	51.44	32.29–70.37	
**Body mass index**							0.86
	Underweight	3	186	314	48.45	23.82–73.43	
	Normal weight	3	1270	2013	53.8	32.73–74.18	
	Overweight	5	677	1249	57.58	46.74–68.08	
	Obesity	7	1043	1853	58.81	50.38–66.98	
**Urban or rural**							0.39
	Rural	23	9275	17,423	51.98	45.6-58.33	
	Urban	17	10,383	22,115	57.59	46.34–68.47	
**Work**							0.65
	Working	7	1286	3124	55.55	39.86–70.71	
	Non-working	6	806	1544	61.41	41.17–79.8	
**Current drinking habit**							0.76
	Yes	5	914	1850	48.68	39.33–58.08	
	No	4	1274	2510	51.12	38.86–63.3	
**Current smoking**							0.48
	Yes	9	343	630	53.42	42.76–63.95	
	No	8	2983	5953	49.07	43.46–54.7	
**Marital status**							0.14
	Single	3	115	241	47.97	39.25–56.76	
	Married	3	769	1353	57.03	50.99–62.98	
	Divorced or Widowed	3	247	399	63.67	47.24–78.63	
**Education level**							0.71
	< 12 years	8	2177	3962	52.77	42.07–63.34	
	> 12 years	9	1449	3105	49.88	39.11–60.64	

*KMI: The modified Kupperman Menopausal Index; MRS: The Menopause Rating Scale; SMI: Simplified; Menopausal Index; MENQOL: The Menopause-Specific Quality of Life; CS-10:10-item Cervantes Scale; WHAS: the Women’s Health Assessment Scale

### Pooled prevalence, subgroup analysis, and risk factors for psychological symptoms

Depression was the psychological symptoms that had the greatest number of included publications. The pooled depression prevalence in middle-aged women was 43.34% (95% CI 40.29–46.42, I^2^ = 99.65%, Supplementary Fig. 6). The prevalence varied by countries, with Cambodia having the highest prevalence (81.36%, 95% CI 75.26–86.78) and Bolivia having the lowest one (10.4%, 95% CI 5.58–16.43, Table 2). When depression was measured by continents, the greatest estimate was found in South America (54.38%, 95% CI 42.23–66.27), whereas lowest estimate in Europe (33.88%, 95% CI 30.08–37.79, *p* < 0.01, Table 2). The lowest prevalence was seen in studies conducted in high-income countries (37.64%, 95% CI 33.78–41.58), compared with those in upper-middle (42.78%, 95% CI 37.38–48.26), lower-middle (49.99%, 95% CI 43.74–56.24) and low-income countries (46.02%, 95% CI 39.55–52.55, *p* < 0.01, Table 2). When studies were categorized by diagnostic tools, we found that studies using the Menopause-Specific Quality of Life (MENQOL) [24] (58.91%, 95% CI 50.28–67.28) had a significantly higher prevalence of depression than those using the Taiwanese Depression Questionnaire [25] (7.21%, 95% CI 1.85–15.32, *p* < 0.01, Table 2). Besides, results indicated that a significant difference in depression prevalence was found in the pooled estimate among development status (developing/developed, 45.57% vs. 39.08%, *p* = 0.03, Table 2), publication date (before 2011 or after 2011, 37.48% vs. 47.35%, *p* < 0.01, Table 2), and study size (more than 1000 participants or less than 1000 participants, 36.09% vs. 45.69%, *p* < 0.01, Table 2). Similar to most menopausal symptoms, women in premenopausal stage (36.27%, 95% CI 30.14–42.63) shared a significantly lower depression prevalence than those in perimenopausal (47.3%, 95% CI 40.89–53.76) and postmenopausal stage (47.62%, 95% CI 42.48–52.78, *p* = 0.01, Table 2). It is interesting to note that women with normal weight had lowest prevalence of depression (*p* < 0.01, Table 2). Moreover, we pooled prevalence of other four psychological symptoms. Physical and mental exhaustion had the highest prevalence (64.13%, 95% CI 60.93–67.27, I^2^ = 99.54%, Supplementary Fig. 7), followed by irritability (54.37%, 95% CI 50.80–57.92, I^2^ = 99.35%, Supplementary Fig. 8), anxiety (50.53%, 95% CI 46.65–54.40, I^2^ = 99.50%, Supplementary Fig. 9), and mood swings (49.03%, 95% CI 43.65–54.43, I^2^ = 99.55%, Supplementary Fig. 10). The subgroup analysis and risk factor analysis for these psychological symptoms were listed in Supplementary Tables 6–9, respectively.

**Table 2:** Subgroup analysis and pooled estimates of risk factors for depression prevalence among middle-aged women

Subgroup		Studies		Event	Total		Prevalence (%)	95% CI (%)		*P* value		
**Country**										< 0.01		
	China	24		14,860	67,753		27.51	21.97–33.41				
	Nepal	7		1463	4386		42.52	19.95–66.86				
	Nigeria	9		1187	3947		30.91	16.06–48.11				
	Iran	9		3701	5580		69.31	51.43–84.62				
	India	32		3585	7415		49.03	39.91–58.17				
	Ethiopia	1		104	226		46.02	39.55–52.55				
	Turkey	10		2305	4587		53.37	41.41–65.15				
	Saudi Arabia	7		1699	2361		64.22	46.82–79.89				
	UK	6		2122	6646		35.58	29.35–42.07				
	France	2		225	900		24.99	22.21–27.88				
	Germany	2		269	896		28.61	20.21–37.82				
	Belgium	2		228	673		35.02	28.5-41.83				
	Netherlands	2		243	901		25.51	17.2-34.83				
	Switzerland	2		225	901		23.49	15.25–32.87				
	Spain	5		5118	13,600		34.34	28.95–39.93				
	Australia	9		2108	4563		43.64	31.75–55.91				
	Japan	5		2706	5662		47.27	28.41–66.53				
	Oman	1		182	472		38.56	34.21-43				
	Multi	4		7238	14,740		47.24	39.18–55.38				
	Macau	1		317	442		71.72	67.42–75.83				
	Ecuador	5		1188	1618		72.85	66.62–78.66				
	Peru	1		578	771		74.97	71.85–77.96				
	Malaysia	5		634	1316		50.74	35.29–66.12				
	Sri Lanka	2		327	1033		34.95	14.07–59.44				
	Mexico	3		4345	12,938		41.23	19.75–64.64				
	Brazil	4		1219	2745		43.84	34.89-53				
	Korea	5		7765	50,745		40.85	22.56–60.56				
	Pakistan	4		2788	4176		46.69	25.53–68.49				
	Greece	2		515	1125		45.76	42.85–48.69				
	Italy	2		132	635		20.8	12.11–31.09				
	Iraq	2		306	842		39.88	0-97.57				
	USA	13		5403	20,366		36.21	29.13–43.61				
	Egypt	5		2192	3704		62.03	45.14–77.54				
	Bangladesh	4		879	1375		71.55	46.25–91.18				
	Qatar	2		645	2259		28.57	23.74–33.67				
	United Arab Emirates	1		101	390		25.9	21.66–30.37				
	Cambodia	1		144	177		81.36	75.26–86.78				
	Taiwan	7		12,866	26,137		23.71	11.51–38.63				
	Sweden	1		72	108		66.67	57.46–75.28				
	Indonesia	2		638	1318		58.23	23.07–89.16				
	New Zealand	1		1045	3616		28.9	27.43–30.39				
	South Africa	1		17	63		26.98	16.66–38.7				
	Libya	1		56	86		65.12	54.69–74.88				
	Morocco	1		84	299		28.09	23.13–33.33				
	Singapore	1		132	656		20.12	17.14–23.28				
	Thailand	2		163	298		54.86	35.07–73.89				
	Hong Kong	1		89	150		59.33	51.35–67.08				
	Portugal	1		266	579		45.94	41.89–50.01				
	Poland	1		92	241		38.17	32.13–44.41				
	Belarus	1		57	119		47.9	38.95–56.92				
	Bolivia	1		13	125		10.4	5.58–16.43				
	Canada	1		2436	13,216		18.43	17.78–19.1				
	Jordan	1		57	143		39.86	31.96–48.03				
	Lebanon	1		111	271		40.96	35.17–46.88				
	Finland	1		32	158		20.25	14.32–26.9				
**Continent**										**< 0.01**		
	Asia	137		58,463	189,944		45.61	41.28–49.98				
	Africa	18		3640	8325		41.71	30.11–53.8				
	Europe	31		12,025	32,273		33.88	30.08–37.79				
	Oceania	10		3153	8179		42.08	31.13–53.44				
	South America	13		6136	12,202		54.38	42.23–66.27				
	North America	17		12,184	46,520		35.96	29.17–43.04				
	Multi	1		1671	3006		55.59	53.81–57.36				
**Income level**										**< 0.01**		
	Upper-Middle-Income	63		28,084	97,591		42.78	37.38–48.26				
	Lower-Middle-Income	78		17,112	33,806		49.99	43.74–56.24				
	Low-Income	1		104	226		46.02	39.55–52.55				
	High-Income	84		49,145	162,747		37.64	33.78–41.58				
**Development status**										**0.03**		
	Developing	146		56,394	154,561		45.57	41.33–49.83				
	Developed	79		36,380	136,803		39.08	35.26–42.96				
**Publication date**										**< 0.01**		
	Before 2011	91		27,348	77,182		37.48	33.77–41.26				
	After 2011	136		69,924	223,267		47.35	43.02–51.7				
**Study size**										**< 0.01**		
	< 1000	173		27,620	61,286		45.69	42.09–49.31				
	> 1000	54		69,652	239,163		36.09	30.93–41.42				
**Study quality**										0.76		
	< 8	35		15,311	52,827		44.41	37.07–51.87				
	≥ 8	192		81,961	247,622		43.14	39.79–46.53				
**Diagnostic tool**										**< 0.01**		
	KMI	13		12,177	47,121		29.78	22.36–37.76				
	MRS	66		28,908	51,731		58.64	53.92–63.29				
	Face-to-face interview	44		11,745	47,432		29.38	24.76–34.21				
	Others	33		18,916	41,213		34.87	28.16–41.9				
	The Greene Climacteric Scale	13		4736	10,813		47.36	39.31–55.48				
	SMI	2		695	2338		28.46	4.94–61.45				
	MENQOL	22		4569	8439		58.91	50.28–67.28				
	SDS [26]	4		778	4254		31.56	3.91–70.18				
	PHQ-9 [27]	6		3320	15,512		44.32	19.73–70.5				
	BDI [28]	10		1057	2670		40.09	26.77–54.18				
	CES-D [29]	9		8370	61,762		31.29	22.71–40.57				
	HAM-D [30]	3		1834	3608		49.65	39.12–60.19				
	Taiwanese Depression Questionnaire	2		167	3556		7.12	1.85–15.32				
**Risk factors**		**Studies**	**Event**			**Total**	**Prevalence (%)**		**95% CI (%)**		**P value**	
**Menopausal stage**											**0.01**	
	Premenopause	58	13,274			67,522	36.27		30.14–42.63			
	Perimenopause	57	12,111			38,119	47.3		40.89–53.76			
	Postmenopause	97	31,129			91,152	47.62		42.48–52.78			
**Age**											0.97	
	< 50	14	1604			6203	36.77		24.91–49.5			
	≥ 50	23	3302			10,049	37.08		28.2-46.42			
**Physical activity**											0.85	
	Regular	7	1053			5260	38.11		15.37–64.02			
	Irregular	7	2119			10,091	41.05		23.69–59.62			
**Body mass index**											**< 0.01**	
	Underweight	3	227			929	24.35		21.62–27.18			
	Normal weight	3	1965			11,380	17.56		15.62–19.58			
	Overweight	7	1611			7935	27.09		16.99–38.54			
	Obesity	9	1479			5285	43.1		25.46–61.67			
**Urban or rural**											0.81	
	Rural	20	7027			17,856	43.73		32.44–55.35			
	Urban	10	5862			19,046	46.95		24.5-70.07			
**Work**											0.92	
	Working	11	2691			10,574	39.36		26.42–53.06			
	Non-working	10	1532			5031	40.61		24.17–58.2			
**Current drinking habit**											0.24	
	Yes	5	985			6496	16.15		9.56–24.02			
	No	5	4940			46,123	27.99		11.14–48.88			
**Current smoking**											0.31	
	Yes	10	787			3002	25.61		17.38–34.74			
	No	10	10,035			77,160	20.26		13.18–28.41			
**Marital status**											0.69	
	Single, Divorced or Widowed	12	1984			8689	40.65		22.69–59.97			
	Married	12	4893			27,465	35.41		18.91–53.92			
**Education level**											0.36	
	< 12 years	16	6187			36,671	35.98		22.08–51.2			
	> 12 years	12	5468			45,214	26.03		13.99–40.05			

*KMI: The modified Kupperman Menopausal Index; MRS: The Menopause Rating Scale; SMI: Simplified Menopausal Index; MENQOL: The Menopause-Specific Quality of Life; SDS: Self-rating Depression Scale; PHQ-9: Patient Health Questionnaire-9; BDI: Beck depression inventory; CES-D: the Center for Epidemiological Studies Depression Scale; HAM-D: Hamilton Depression Rating Scale

### Pooled prevalence, subgroup analysis, and risk factors for urogenital symptoms

Sexual problems account for the highest prevalence (45.45%, 95% CI 41.89–49.04, I^2^ = 99.56%, Supplementary Fig. 11) among urogenital symptoms, with vagina dryness (37.34%, 95% CI 34.30-40.44, I^2^ = 99.40%, Supplementary Fig. 12) and urinary problems (34.49%, 95% CI 31.70-37.34, I^2^ = 99.42%, Supplementary Fig. 13) following closely behind. Moreover, the results indicated that there was a substantial variation in the prevalence of these three urogenital symptoms among countries (*p* < 0.01, Table 3). When assessed by continents, South America (60.94%, 95% CI 53.24–68.38, *p* < 0.01, Table 3) had the highest estimate of sexual problems. Nevertheless, there was no statistical difference was found in vagina dryness (*p* = 0.45) and urinary symptoms (*p* = 0.11) by continents (Table 3). Additionally, compared with publications after 2011, the prevalence of sexual problems (40.86% vs. 49.08%, *p* = 0.02), vagina dryness (33.23% vs. 40.47%, *p* = 0.02) and urinary problems (29.38% vs. 37.73%, *p* < 0.01) was consistently lower in publications after 2011 (Table 3). However, there was minimal difference observed among development status of countries in urogenital symptoms (sexual problems, 45.37% vs. 45.94%, *p* = 0.87; vagina dryness, 38.28% vs. 36.1%, *p* = 0.47, urinary symptoms, 35.89% vs. 31.58%, *p* = 0.13, Table 3). Studies with more than 1000 participants reported a lower prevalence of vagina dryness (32.13% vs. 38.77%, *p* = 0.03) and urinary symptoms (29.52% vs. 35.97%, *p* = 0.03) than those with less than 1000 participants (Table 3). Prevalence varied significantly by diagnostic tools, with the highest by using the Greene Climacteric Scale [31] (63.44%, 95% CI 52.47–73.75) for sexual problems, CS-10 (51.48%, 95% CI 22.27–80.14) for vagina dryness, and MENQOL (48.13%, 95% CI 40.32–55.99) for urinary problems, shown in Table 3. With regard to menopausal stage, we found that for each of the three urogenital symptoms, women in postmenopausal stage resulted in the highest prevalence (53.97%, 44.81%, and 40.27% for sexual problems, vagina dryness, and urinary problems, respectively), followed by premenopausal stage (35.24%, 21.16%, and 22.21% for sexual problems, vagina dryness, and urinary problems, respectively) and perimenopausal stage (48.82%, 36.07%, and 33.29% for sexual problems, vagina dryness, and urinary problems, respectively, *p* < 0.01, Table 3). Intriguingly, we found BMI of middle-aged women were linearly correlated with prevalence of urinary problems, those of obesity had a highest prevalence of 31.73% (95% CI 19.13–45.86), followed by overweight (20.41%, 95% CI 10.24–32.94), normal weight (13.03%, 95% CI 10.72–15.54), and underweight (10.61%, 95% CI 3.09–21.71, *p* = 0.01, Table 3). The subgroup analysis and risk factor analysis for these urogenital symptoms were listed in Table 3.

**Table 3:** Subgroup analysis and pooled estimates of risk factors for prevalence of urogenital symptoms among middle-aged women

1. Sexual Problems									
Subgroup		Studies	Event	Total	Prevalence (%)	95% CI (%)	*P* value		
**Country**							< 0.01		
	China	21	22,284	64,498	40.76	30.12–51.86			
	Nepal	7	1655	4386	46.3	23.24–70.23			
	Nigeria	7	1299	3020	46.78	20.42–74.15			
	Iran	8	3265	6517	53.06	30.04–75.42			
	India	26	2924	6522	45.5	32.58–58.72			
	Ethiopia	1	61	226	26.99	21.39–32.98			
	Turkey	7	1018	2529	48.47	30.78–66.36			
	Saudi Arabia	7	1240	2238	47.27	33.43–61.33			
	UK	4	2368	5282	45.66	42.33–49.02			
	France	2	351	900	38.31	32.57–44.22			
	Germany	2	337	896	36.71	30.1-43.59			
	Belgium	2	318	673	50.19	39.75–60.61			
	Netherlands	2	417	901	45.83	40.88–50.81			
	Switzerland	2	315	901	31.06	12.81–53.06			
	Spain	5	1659	3346	50.23	45.51–54.94			
	Australia	8	2600	4347	60.52	51.14–69.53			
	Japan	2	1639	2249	73.29	69.44–76.98			
	Oman	1	121	472	25.64	21.79–29.68			
	Macau	1	311	442	70.36	66.01–74.53			
	Ecuador	5	975	1502	64.88	52.49–76.35			
	Peru	1	453	771	58.75	55.26–62.21			
	Malaysia	6	576	1335	47.35	31.92–63.03			
	Sri Lanka	2	123	1033	13.49	0.89–37.06			
	Brazil	2	934	1775	56.59	42.97–69.72			
	Korea	3	2797	4352	47.41	15.5-80.55			
	Singapore	2	255	1151	21.43	13.53–30.56			
	Pakistan	5	2139	4412	28.1	13.2-45.96			
	Greece	2	670	1125	60.19	55.6-64.69			
	Philippines	1	64	195	32.82	26.39–39.59			
	Indonesia	3	602	1377	52.7	28.52–76.22			
	Taiwan	3	10,313	21,263	37.59	19.93–57.12			
	Thailand	3	190	448	41.71	16.75–69.17			
	Vietnam	1	69	100	69	59.55–77.73			
	Italy	1	96	301	31.89	26.74–37.28			
	Iraq	1	150	342	43.86	38.63–49.15			
	USA	8	3207	12,185	34.98	26.71–43.72			
	Egypt	5	1573	3704	39.5	10.13–73.92			
	Bangladesh	2	409	899	47.51	17.51–78.54			
	Mexico	1	228	290	78.62	73.7-83.16			
	Qatar	1	280	1158	24.18	21.76–26.69			
	United Arab Emirates	1	93	390	23.85	19.74–28.21			
	Cambodia	1	54	177	30.51	23.93–37.51			
	Sweden	1	67	109	61.47	52.12–70.42			
	Multi	2	2959	7797	35.41	17.77–55.39			
	South Africa	1	38	63	60.32	47.9-72.11			
	Libya	1	42	86	48.84	38.29–59.44			
	Morocco	1	60	299	20.07	15.71–24.81			
	Hong Kong	1	96	150	64	56.13–71.51			
	Portugal	1	223	728	30.63	27.33–34.03			
	Poland	1	149	241	61.83	55.59–67.87			
	Belarus	1	83	119	69.75	61.16–77.7			
	Bolivia	1	64	125	51.2	42.41–59.95			
	Lebanon	1	141	271	52.03	46.06–57.97			
**Continent**							**< 0.01**		
	Asia	117	52,808	128,906	44	39.15–48.91			
	Africa	16	3073	7398	42.33	26.76–58.72			
	Europe	27	9233	20,313	46.34	42.06–50.66			
	Oceania	8	2600	4347	60.52	51.14–69.53			
	South America	9	2426	4173	60.94	53.24–68.38			
	North America	9	3435	12,475	40	27.99–52.65			
	Multi	1	779	3006	25.91	24.36–27.5			
**Income level**							**< 0.01**		
	Upper-Middle-Income	52	28,061	77,206	48.04	41.79–54.32			
	Lower-Middle-Income	71	14,441	33,037	43.89	36.66–51.26			
	Low-Income	1	61	226	26.99	21.39–32.98			
	High-Income	63	31,791	70,149	45.38	41.2–49.6			
**Development status**							0.87		
	Developing	122	51,788	127,944	45.37	40.4-50.38			
	Developed	64	21,787	49,668	45.94	41.68–50.22			
**Publication date**							**0.02**		
	Before 2011	82	20,899	53,390	40.86	36.38–45.41			
	After 2011	105	53,455	127,228	49.08	43.9-54.27			
**Study size**							0.27		
	< 1000	151	23,542	50,476	46.29	42.13–50.47			
	> 1000	36	50,812	130,142	42.04	35.89–48.32			
**Study quality**							0.99		
	< 8	26	16,966	48,856	45.52	37.39–53.76			
	≥ 8	161	57,388	131,762	45.44	41.52–49.39			
**Diagnostic tool**							**< 0.01**		
	KMI	13	17,151	48,291	40.62	29.52–52.22			
	MRS	64	19,335	41,588	46.42	39.9–53			
	Face-to-face interview	39	10,180	34,326	37.1	30.12–44.36			
	Others	37	17,387	39,061	41.94	35.32–48.69			
	The Greene Climacteric Scale	11	4607	6803	63.44	52.47–73.75			
	MENQOL	21	4252	8039	57.48	46.09–68.48			
	FSFI [32]	2	1442	2510	53.13	41.9-64.21			
**Risk factors**		**Studies**	**Event**	**Total**	**Prevalence (%)**	**95% CI (%)**	**P value**		
**Menopausal stage**							**< 0.01**		
	Premenopause	43	9217	34,406	35.24	28.76-42			
	Perimenopause	47	9674	22,999	48.82	41.59–56.08			
	Postmenopause	73	24,494	49,059	53.97	47.39–60.48			
**Age**							0.69		
	< 50	5	494	1210	39.62	31.42–48.12			
	≥ 50	11	2400	4795	44.28	24.43–65.13			
**Urban or rural**							0.17		
	Rural	15	4677	9669	49.18	32.86–65.59			
	Urban	6	3316	6123	69.83	45.49–89.38			
**Work**							0.22		
	Working	3	882	1877	59.1	38.64–78.06			
	Non-working	2	161	185	85.14	46.35–100			
**2. Vagina dryness**									
**Subgroup**		**Studies**	**Event**	**Total**	**Prevalence (%)**	**95% CI (%)**	**P value**		
**Country**							**< 0.01**		
	Nepal	7	1564	4386	47.05	25.15–69.56			
	Nigeria	5	565	2138	34.82	19.22–52.28			
	Iran	9	2100	4703	42.87	22.79–64.25			
	India	22	2035	6862	31.6	21.07–43.17			
	Ethiopia	1	69	226	30.53	24.68–36.71			
	Turkey	7	1149	3215	41.73	26.27–58.07			
	Saudi Arabia	7	1316	2361	50.68	37.35–63.96			
	UK	5	1543	5531	27.08	22.09–32.38			
	France	2	252	900	25.24	12.17–41.12			
	Germany	2	176	896	19.63	17.08–22.3			
	Belgium	2	250	673	50.24	17.63–82.73			
	Netherlands	2	276	901	30.63	27.65–33.68			
	Switzerland	2	218	901	21.23	8.37–37.96			
	Spain	4	1350	2447	56.95	26.32–84.89			
	Oman	1	70	472	14.83	11.76–18.19			
	Macau	1	213	442	48.19	43.54–52.86			
	Taiwan	4	10,545	22,623	30.97	17.38–46.46			
	Ecuador	5	808	1565	51.56	31.7-71.16			
	Peru	1	265	771	34.37	31.06–37.76			
	Malaysia	7	700	1504	49.45	41.46–57.45			
	China	12	5626	22,135	34.44	23.16–46.67			
	Sri Lanka	2	160	1033	17.38	3.91–37.6			
	Australia	6	674	2314	26.95	10.81–47.08			
	Mexico	2	1829	7925	38.93	8.78–74.92			
	Brazil	3	813	2375	30.21	20.72–40.63			
	Korea	5	3165	5665	52.15	45.74–58.52			
	Japan	2	948	3030	31.95	26.89–37.23			
	Singapore	2	267	1151	22.94	18.65–27.53			
	USA	11	4555	17,589	30.51	24.39–37.01			
	Pakistan	4	1139	3549	33.11	21.6-45.74			
	Greece	2	433	1125	45.7	27.51–64.49			
	Philippines	1	115	195	58.97	51.98–65.8			
	Indonesia	3	431	1377	46.27	16.51–77.57			
	Thailand	3	228	448	50.97	39.86–62.03			
	Vietnam	1	90	100	90	83.25–95.22			
	Italy	1	48	301	15.95	12.01–20.31			
	Sweden	2	2061	7016	37.32	20.69–55.65			
	Poland	2	254	590	44.24	30.04–58.94			
	Iraq	2	368	842	41.73	23.23–61.52			
	Egypt	5	1459	3704	43.08	20.53–67.27			
	Bangladesh	2	427	899	49.2	24.12–74.5			
	Qatar	1	296	1158	25.56	23.09–28.12			
	United Arab Emirates	1	107	390	27.44	23.11–31.98			
	Cambodia	1	66	177	37.29	30.29–44.56			
	Multi	3	3807	11,317	32.72	20-46.89			
	New Zealand	1	1263	3616	34.93	33.38–36.49			
	Libya	1	21	86	24.42	15.86–34.11			
	Morocco	1	45	299	15.05	11.21–19.34			
	Hong Kong	1	67	150	44.67	36.77–52.7			
	Portugal	1	229	728	31.46	28.13–34.88			
	Belarus	1	26	119	21.85	14.84–29.76			
	Bolivia	1	51	125	40.8	32.32–49.57			
	Colombia	1	626	1739	36	33.76–38.27			
	Lebanon	1	35	271	12.92	9.17–17.19			
**Continent**							0.45		
	Asia	109	33,227	89,138	39.35	35.03–43.75			
	Africa	13	2159	6453	35.13	24.18–46.94			
	Europe	29	8702	26,919	34.54	28.14–41.23			
	South America	11	2563	6575	41.51	31.16–52.26			
	Oceania	7	1937	5930	28.07	13.72–45.19			
	North America	13	6384	25,514	31.77	25.12–38.81			
	Multi	2	2221	6526	32.52	12.09–57.3			
**Income level**							0.11		
	Lower-Middle-Income	65	10,282	29,818	37.69	31.33–44.27			
	Low-Income	1	69	226	30.53	24.68–36.71			
	Upper-Middle-Income	47	13,309	46,172	40.24	35.04–45.56			
	High-Income	71	33,533	90,839	35.21	31.25–39.28			
**Development status**							0.47		
	Developing	112	33,419	95,452	38.28	33.99–42.67			
	Developed	71	23,137	68,597	36.1	32.07–40.22			
**Publication date**							**0.02**		
	Before 2011	79	18,895	69,009	33.23	29.2-37.38			
	After 2011	105	38,298	98,046	40.47	36.18–44.84			
**Study size**							**0.03**		
	< 1000	146	18,308	47,658	38.77	35.13–42.48			
	> 1000	38	38,885	119,397	32.13	27.61–36.82			
**Study quality**							0.79		
	< 8	22	7590	24,131	36.24	27.93–44.99			
	≥ 8	162	49,603	142,924	37.49	34.23–40.82			
**Diagnostic tool**							**< 0.01**		
	MRS	66	15,944	42,577	39.92	34.81–45.14			
	Face-to-face interview	42	9291	45,059	25.4	20.94–30.14			
	KMI	4	1942	7182	27.7	12.01–46.92			
	MENQOL	20	3598	8070	43.86	32.5-55.56			
	The Keio questionnaire	2	948	3030	31.95	26.89–37.23			
	Others	44	21,245	51,669	43	37.18–48.92			
	The Greene Climacteric Scale	4	3297	7278	39.67	21.68–59.23			
	CS-10	2	928	2190	51.48	22.27–80.14			
**Risk factors**		**Studies**	**Event**	**Total**	**Prevalence (%)**	**95% CI (%)**	**P value**		
**Menopausal stage**							**< 0.01**		
	Premenopause	40	4743	21,621	21.16	16.42–26.3			
	Perimenopause	46	7186	20,967	36.07	30.54–41.78			
	Postmenopause	74	22,880	54,304	44.81	39.03–50.67			
**Age**							0.16		
	< 50	5	405	1078	43.39	31.34–55.85			
	≥ 50	15	5088	14,666	32.27	23.06–42.22			
**Urban or rural**							0.11		
	Rural	11	2648	9005	29.49	18.91–41.31			
	Urban	6	2184	7143	61.64	24.5-92.26			
**3.Urinary problems**									
**Subgroup**		**Studies**	**Event**	**Total**	**Prevalence (%)**	**95% CI (%)**	**P value**		
**Country**							**< 0.01**		
	China	23	13,804	64,261	24.2	18.98–29.84			
	Nepal	6	1097	2386	39.38	21.98–58.3			
	Nigeria	7	452	3020	19.37	5.8-38.27			
	Iran	9	2578	6819	44.1	24.39–64.82			
	India	26	3066	7578	40.09	31.06–49.46			
	Ethiopia	1	59	226	26.11	20.57–32.04			
	Turkey	9	2315	4535	51.6	39.48–63.64			
	Saudi Arabia	7	1312	2361	51.37	39.34–63.31			
	UK	4	1895	5447	31.53	24.27–39.28			
	France	2	210	900	23.32	20.61–26.15			
	Germany	3	1020	2789	28	13.78–44.94			
	Belgium	2	198	673	39.84	13.71–69.54			
	Netherlands	2	324	901	35.23	29.41–41.27			
	Switzerland	2	171	901	16.65	6.68–29.92			
	Spain	4	570	1676	33	17.6-50.54			
	Oman	1	112	472	23.73	19.99–27.68			
	Macau	1	244	442	55.2	50.54–59.82			
	Taiwan	5	9654	22,784	29.96	21.2-39.52			
	Ecuador	5	797	1684	45.63	32.37–59.21			
	Peru	1	429	771	55.64	52.12–59.14			
	Malaysia	7	426	1504	28.99	21.69–36.87			
	Sri Lanka	2	235	1033	24.01	15.21–34.08			
	Brazil	3	547	2375	21.02	15.39–27.27			
	China	1	13,804	64,261	8.69	8.14–9.26			
	Korea	4	2506	4922	46.77	33.17–60.61			
	Japan	2	1161	3030	42.07	17.07–69.49			
	Singapore	2	245	1151	21.2	18.39–24.16			
	Pakistan	7	1803	5467	34.34	23.09–46.55			
	Philippines	1	129	195	66.15	59.35–72.64			
	Indonesia	2	255	377	45.83	0.43–97.41			
	Thailand	3	165	448	36.01	9.4-68.44			
	Vietnam	1	59	100	59	49.18–68.48			
	Australia	7	2373	10,803	35.97	21.29–52.12			
	Italy	2	60	635	9.34	3.84–16.85			
	Poland	2	198	590	34.17	25.47–43.44			
	Iraq	3	923	1949	46.52	30.81–62.59			
	USA	5	1886	9877	32.52	18.43–48.43			
	Egypt	5	1522	3704	45.16	32.59–58.05			
	Bangladesh	3	798	2489	34.73	8.6-67.34			
	Qatar	1	266	1158	22.97	20.59–25.44			
	United Arab Emirates	1	104	390	26.67	22.39–31.17			
	Cambodia	1	83	177	46.89	39.57–54.28			
	Sweden	1	55	108	50.93	41.47–60.35			
	New Zealand	1	160	3616	4.42	3.78–5.12			
	Multi	1	145	360	40.28	35.26–45.4			
	Morocco	1	57	299	19.06	14.8-23.72			
	Hong Kong	1	59	150	39.33	31.64–47.29			
	Portugal	1	111	728	15.25	12.72–17.95			
	Belarus	1	18	119	15.13	9.19–22.18			
	Greece	1	21	100	21	13.52–29.58			
	Colombia	1	452	1739	25.99	23.96–28.08			
	Jordan	1	43	143	30.07	22.81–37.87			
	Lebanon	1	68	271	25.09	20.1-30.44			
**Continent**							0.11		
	Asia	131	44,346	146,209	36.76	33.21–40.39			
	Africa	14	2090	7249	28.43	17.42–40.92			
	Europe	28	4996	15,927	27.93	23.14–32.98			
	South America	10	2225	6569	36.66	26.38–47.6			
	Oceania	8	2533	14,419	30.83	15.96–48.07			
	North America	5	1886	9877	32.52	18.43–48.43			
**Income level**							**< 0.05**		
	Upper-Middle-Income	59	20,999	89,587	32.6	28.01–37.35			
	Lower-Middle-Income	72	12,202	33,915	37.79	32.29–43.46			
	Low-Income	1	59	226	26.11	20.57–32.04			
	High-Income	64	24,816	76,522	32.74	28.74–36.87			
**Development status**							0.13		
	Developing	133	43,025	146,131	35.89	32.27–39.6			
	Developed	63	15,051	54,119	31.58	27.54–35.75			
**Publication date**							**< 0.01**		
	Before 2011	75	11,784	52,557	29.38	25.6-33.31			
	After 2011	121	46,292	147,693	37.73	33.98–41.56			
**Study size**							**0.03**		
	< 1000	153	18,038	49,798	35.97	32.69–39.32			
	> 1000	43	40,038	150,452	29.52	24.71–34.56			
**Study quality**							0.54		
	< 8	27	11,101	54,188	32.06	23.88–40.84			
	≥ 8	169	46,975	146,062	34.89	31.94–37.89			
**Diagnostic tool**							**< 0.01**		
	KMI	16	11,479	56,232	22.96	17.18–29.31			
	MRS	63	13,085	34,214	39.62	34.24–45.13			
	Face-to-face interview	45	10,015	47,253	24.84	20.66–29.28			
	Others	48	17,653	49,128	35.33	30-40.85			
	MENQOL	20	3955	8203	48.13	40.32–55.99			
	The Keio questionnaire	2	1161	3030	42.07	17.07–69.49			
	CS-10	2	728	2190	43.08	12.38–77.13			
**Risk factors**			**Studies**	**Event**	**Total**	**Prevalence (%)**	**95% CI (%)**		**P value**
**Menopausal stage**									**< 0.01**
		Premenopause	43	6877	34,966	22.21	17.31–27.53		
		Perimenopause	47	7419	24,305	33.29	27.49–39.36		
		Postmenopause	82	108,693	148,061	40.27	34.59–46.09		
**Age**									0.08
		< 50	8	2223	10,596	24.27	15.61–34.12		
		≥ 50	17	2224	7402	36.32	27.04–46.14		
**Body mass index**									**0.01**
		Underweight	2	37	392	10.61	3.09–21.71		
		Normal weight	2	422	3215	13.03	10.72–15.54		
		Overweight	3	187	1112	20.41	10.24–32.94		
		Obesity	4	290	856	31.73	19.13–45.86		
**Urban or rural**									0.32
		Rural	14	3685	10,852	40.59	29.99–51.66		
		Urban	8	3179	8001	53.96	30.4-76.62		
**Work**									0.36
		Working	4	547	1938	34.13	4.59–73.27		
		Non-working	3	217	416	56.56	29.81–81.43		
**Education level**									0.83
		< 12 years	5	1130	4691	30.95	21.24–41.58		
		> 12 years	4	239	675	32.3	20.14–45.73		

*KMI: The modified Kupperman Menopausal Index; MRS: The Menopause Rating Scale; MENQOL: The Menopause-Specific Quality of Life; CS-10:10-item Cervantes Scale; FSFI: The Female Sexual Function Index

### Pooled prevalence, subgroup analysis, and risk factors for other symptoms

The prevalence of poor memory, difficulty concentrating, formication, changing in the appearance, texture, or tone of skin, increased facial hair, and drying skin were 54.44% (95% CI 48.87–59.95, I^2^ = 99.43%, Supplementary Figs. 14), 44.85% (95% CI 37.71–52.09, I^2^ = 99.32%, Supplementary Figs. 15), 20.50% (95% CI 13.44–28.60, I^2^ = 99.75%, Supplementary Figs. 16), 46.48% (95% CI 36.21–56.89, I^2^ = 98.75%, Supplementary Figs. 17), 27.19% (95% CI 21.09–33.74, I^2^ = 99.00%, Supplementary Figs. 18) and 46.03% (95% CI 38.81–53.34, I^2^ = 99.48%, Supplementary Fig. 19). The subgroup analysis and risk factor analysis for these symptoms were listed in Supplementary Tables 10–15, respectively.

### Grading of recommendations, Assessment, Development and evaluations (GRADE) quality of evidence

The certainty of evidence for different menopausal symptoms (very low) were assessed using the GRADE framework. The results of this assessment are shown in Supplementary Table 16.

## Discussion

This was the first and largest systematic review and meta-analysis to explore the global prevalence of menopause-related symptoms among middle-aged women from multiple domains involving somatic, psychological, urogenital and others symptoms. The meta-analysis found that the prevalence of these symptoms varies considerably, with the highest prevalence of joint and muscular discomfort (65.43%, 95% CI 62.51–68.29) and lowest of formication (20.5%, 95% CI 13.44–28.60). Menopausal symptom epidemiology was significantly influenced by factors such as countries, continents, country development, country income level and diagnostic tools. Furthermore, it was shown that the prevalence of most symptoms in postmenopausal stage increased dramatically. Additionally, a noteworthy distinction was observed between BMI and sleep problems, depression, anxiety and urinary problems.

Menopause is characterized by vasomotor symptoms, which include hot flashes, perspiration, and occasionally shaking and a cold feeling. Because of their abrupt and seemingly random onset throughout the day or even at night, these are usually the most common and irritating menopausal symptoms. Vasomotor symptoms can start up to two years before to the final menstrual period (FMP), peak one year following the FMP, and last for four years in about half of the female population. The multiethnic, community-based Study of Women’s Health Across the Nation (SWAN) [33–35] reported that vasomotor symptoms were more prevalent among African-American and Hispanic women and less prevalent among Japanese-American and Chinese-American women than white women. As the most important vasomotor symptom, emerging analyses of studies revealed that the prevalence of hot flashes in Asian women is similar to those of Western countries [36, 37]. As a result, the current study’s pooled estimates of different continents find that women in Africa with highest prevalence of hot flashes, whereas women in Asia, Europe, and North America are of comparable prevalence, which validates prior studies [33–35]. Besides, our result found the prevalence of sleeping problems (51.89%, 95% CI 49.55–54.22) are similar to pooled estimates of a previous meta-analysis (51.6%, 95% CI 44.6–58.5) [13]. Six out of ten middle-aged women reported having joint and muscular discomfort, which was the most common somatic symptom. The idea that a decline in ovarian function may have a direct detrimental impact on muscle and joint tissue stems from the fact that these tissues have estrogen receptors (ERs) [38, 39]. Importantly, pooled prevalence estimates show that, with the exception of headache, all somatic domain complaints are more common in the perimenopause and postmenopause than in the premenopause. According to community-based studies, women’s migraine headache prevalence has been shown to rise throughout the perimenopause and fall during the postmenopause [40, 41]. This study found a similar tendency, albeit it was not statistically significant. It’s interesting to note that women who have abnormal weight—that is, underweight, overweight, or obese—are more likely to experience sleep problems. This finding is in line with a study by Prather et al. that discovered a link between sleep disturbance and obesity or overweight [42]. The worrying trend of rising obesity rates among postmenopausal women globally necessitates further attention [43–45].

Menopause can be psychologically distressing for women. The global prevalence of depression among middle-aged women was found to be approximately 43.34% with equally matched prevalence of study from global perspective [46]. Furthermore, current findings revealed strong correlation between the prevalence of depression among middle-aged women with country development. This is in line with previous research, which has shown that middle-aged women from developing countries have a higher prevalence of depression. This could be explained by governments from developed countries have greater beneficial and supportive policies for public health [47]. In contrast, middle-aged women were disadvantaged in healthcare and living conditions, which in turn predisposed them to depression. Different from somatic symptoms, only exhaustion and depression in psychological domain are related to menopausal stage, with climbing prevalence from premenopausal to postmenopausal stage, while anxiety, irritability and mood swings have no statistical difference. Consistent with other studies [46, 48], irritability levels in our study rise throughout menopause and diminish following menopause, though not statistically significant. While other research [10, 49–52] revealed a tenuous connection between depression and being overweight or obese, our investigation showed that these conditions raise the risk of depression in middle-aged women.

Interestingly, the prevalence of symptoms in urogenital domain is similar across countries with different status of development where middle-aged women from, which indicates minimal relationship between develop status of countries and urogenital symptoms among middle-aged women. Although they are not frequently reported, urogenital symptoms are often present after menopause [53]. Longitudinal and cross-sectional studies have reported that the menopausal transition is associated with urogenital symptoms, independent of aging [54]. Our findings are in line with previous research that prevalence of urogenital problems is sharp rise across menopausal stage (*p* < 0.01), but a weaker correlation with age (*p* = 0.69, 0.19, 0.08 for sexual problems, vagina dryness, and urinary problems, respectively). Pastore, et al [55] found that overweight seems to be linked with a two to four folds higher incidence of urogenital symptoms in women with normal weight. Current study is consistent with it that overweight or obesity are found to be important correlates of urinary problems.

Greendale et al. [56]. discovered an intriguing circumstance: women going through the perimenopausal stage of the transition frequently report experiencing a decrease in memory and focus. The current study also discovered, while not statistically significantly, that middle-aged women going through the perimenopausal stage are more likely to experience memory loss and concentration problems. More precisely, as compared to the premenopausal and postmenopausal stages, the perimenopausal stages were found to have deficiencies in processing speed and a lack of progress in verbal memory with repeated testing [56]. These findings imply that the negative impact of menopause on cognitive function is only present during the perimenopausal phase. Given that anatomical studies have shown that the hippocampus and prefrontal cortex, which govern episodic and working memory, display high amounts of ERs, it is thought that estradiol plays a significant role in cognitive performance [57]. Thus, the transitory cognitive abnormalities reported clinically at this time may be caused by fluctuating levels of estrogen during perimenopause [57].

There are strengths of this meta-analysis which included the largest population-based study to-date, inclusion of nineteen symptoms from multiple domains for a more comprehensive understanding of menopause and use of subgroup analysis to pool estimates of risk factors with improved accuracy compared with findings from a single study. However, several limitations should be noted. First, the heterogeneity between studies remains unexplained by the variables studied. Variations in study sample size and representativeness contribute significantly to the heterogeneity of the prevalence. Second, data based on participants’ self-reports can result in reporting bias. Third, most research focused on cross-sectional studies creates recall bias. Fourth, significant lack of articles from countries with low-income level. Finally, GRADE approach indicated our results with a suboptimal quality of evidence. Therefore, higher-quality research is needed in the future to clarify the conclusions.

## Conclusions

Women typically spend about 30% of their lifespan around the menopause. Our study indicated that most menopause-related symptoms affected 50% middle-aged women. Thus, it is important to ensure women and health professionals understand the perimenopause transition, its symptoms and treatments and create a more positive view to the menopause. Health-care providers caring for women at all levels of the healthcare system must be well prepared to guide women through this transition and provide advice to improve quality of life.

### Electronic supplementary material

Below is the link to the electronic supplementary material.

Supplementary Material

## Data Availability

Original data generated and analyzed during this study are included in this published article or in the data repositories listed in References.
