# Supplementary material for: Mapping global prevalence of menopausal symptoms among middle-aged women: a systematic review and meta-analysis
Source: BMC Public Health. 2024 Jul 2;24:1767. doi: 10.1186/s12889-024-19280-5 (PMC11220992; doi:10.1186/s12889-024-19280-5)
Supplement: Supplementary file 1 — Supplementary Material [file 12889_2024_19280_MOESM1_ESM.pdf]

Supplementary file to

Mapping global prevalence of menopausal symptoms among middle-aged women: a systematic review and meta-analysis

Supplementary method. Searching strategy for global prevalence of menopausal symptoms.

**Database searched**	**Years of coverage**	**References**
Embase	2000-2023	39799
Medline		30530
Web of Science		26873
Cochrane		5061
Total		102263

Search strategy—Embase

1	('middle aged'/de OR ('middle age' OR 'middle aged'):ab,ti,kw)
2	('premenopause'/de OR ('menopause, pre' OR 'pre menopause' OR 'premenopausal' OR 'premenopause'):ab,ti,kw)
3	('climacterium'/de OR ('climacteric' OR 'menopausal transition' OR 'perimenopausal' OR 'perimenopause' OR 'climacterium'):ab,ti,kw)
4	('postmenopause '/de OR ('post menopause' OR 'postmenopausal' OR 'postmenopause'):ab,ti,kw)
5	('female'/de OR ('females' OR 'woman' OR 'women' OR 'female'):ab,ti,kw)
6	(1 OR 2 OR 3 OR 4)
7	(5 AND 6)
8	('epidemiological data'/de OR 'epidemiology'/de OR 'geographic distribution'/de OR 'patient volume'/de OR prevalence/exp OR 'incidence'/de OR geography/de OR 'geographic names'/exp OR 'cross-sectional study'/de OR (epidemiolog* OR ((geograph* OR global*) NEAR/3 (distribut*)) OR (patient* NEAR/3 volume*) OR prevalen* OR incidenc* OR population-based* OR cross-sectional*):ab,ti,kw) NOT ((animal/exp OR animal*:de OR nonhuman/de) NOT ('human'/exp)) NOT ('case report'/de OR 'case report*':ti) NOT ([Conference Abstract]/lim) AND [english]/lim
9	('menopausal syndrome'/de OR ((menopausal* OR climacterial* OR climacteric* OR climacterium* OR menopause* OR perimenopausal* OR postmenopausal* OR postmenopause*) NEAR/6 (disorder* OR syndrome* OR complaint* OR symptom* OR distress*)):ab,ti,kw)
10	('hot flush'/de OR ('flush, hot' OR 'hot flash' OR 'hot flashes' OR 'hot flushing'):ab,ti,kw)
11	(('vasomotor symptoms' OR 'vasomotor symptom'):ab,ti,kw)
12	((((sleep* OR sleeping*) NEAR/3 ('disorder' OR 'problem' OR 'disturbance' OR 'disruption')) OR 'sleeplessness' OR 'insomnia' OR 'difficulty sleeping'):ab,ti,kw)
13	(('heart discomfort' OR 'palpation' OR 'palpations'):ab,ti,kw)
14	('headache'/de OR ('headaches' OR 'migraine'):ab,ti,kw)
15	((((muscle* OR muscular* OR joint* OR osteomuscular* OR shoulder* OR limb*) NEAR/3 (pain OR ache OR discomfort OR aching OR stiff)) OR 'arthralgia' OR 'myalgia' OR 'backache'):ab,ti,kw)

16	((((physical* OR mental*) NEAR/3 ('exhaustion')) OR 'fatigue' OR tired* OR 'lack of energy'):ab,ti,kw)
17	('depression'/de OR ('depressive disease' OR 'depressive disorder' OR 'depressive episode' OR 'depressive illness' OR 'depressive symptom' OR 'depressive syndrome' OR 'depressive mood' OR 'depressed'):ab,ti,kw)
18	('anxiety'/de OR 'anxious':ab,ti,kw)
19	('irritability'/de OR 'irritable mood':ab,ti,kw)
20	('mood swings':ab,ti,kw)
21	(((reduce* OR loss* OR decrease* OR change*) NEAR/3 ('libido' OR 'libido sexualis' OR 'sex drive' OR 'sexual drive' OR 'sexual libido')):ab,ti,kw)
22	('vaginal dryness'/de OR ('dry vagina' OR 'vaginal dryness'):ab,ti,kw)
23	(((bladder* OR urinary*) NEAR/2 (problem* OR symptom*)):ab,ti,kw)
24	(('poor memory' OR 'memory problem' OR 'forgetfulness' OR 'loss of memory'):ab,ti,kw)
25	(('difficulty concentrating' OR 'difficulty concentration' OR 'lake of concentration' OR 'poor concentration' OR 'decrease concentration'):ab,ti,kw)
26	('formication':ab,ti,kw)
27	('change in the appearance, texture, or tone of my skin':ab,ti,kw)
28	('facial hair':ab,ti,kw)
29	('drying skin':ab,ti,kw)
30	(9 OR 10 OR 11 OR 12 OR 13 OR 14 OR 15 OR 16 OR 17 OR 18 OR 19 OR 20 OR 21 OR 22 OR 23 OR 24 OR 25 OR 26 OR 27 OR 28 OR 29)
31	(7 AND 8 AND 30)

Search strategy—Medline

S1	(Epidemiological Monitoring/ OR Epidemiology/ OR Epidemiology.fs. OR exp Incidence/ OR exp Prevalence/ OR Incidence/ OR Geography/ OR exp Geographic Locations/ OR Epidemiologic Studies OR Cross-Sectional Studies/ OR (epidemiolog* OR ((geograph* OR global*) ADJ3 (distribut*)) OR (patient* ADJ3 volume*) OR prevalen* OR incidenc* OR population-based* OR cross-sectional*).ab,ti,kf.) NOT (exp Animals/ NOT Humans/) NOT (news OR congres* OR abstract* OR book* OR chapter* OR dissertation abstract*).pt. AND english.la
S2	(middle aged/ OR ('middle age' OR 'middle aged').ab,ti,kf.)
S3	(Premenopause/ OR ('menopause, pre' OR 'pre menopause' OR 'premenopausal' OR 'premenopause').ab,ti,kf.)
S4	(Perimenopause/ OR ('climacteric' OR 'menopausal transition' OR 'perimenopausal' OR 'climacterium' OR 'climacterium').ab,ti,kf.)
S5	(Postmenopause/ OR ('post menopause' OR 'postmenopausal' OR 'postmenopause').ab,ti,kf.)
S6	(S2 OR S3 OR S4 OR S5)
S7	S6 AND (Female/ OR ('females' OR 'woman' OR 'women' OR 'female').ab,ti,kf.)
S8	(((menopausal* OR climacterial* OR climacteric* OR climacterium* OR menopause* OR perimenopausal* OR postmenopausal* OR postmenopause*) ADJ6 (disorder* OR

	syndrome* OR complaint* OR symptom* OR distress*)).ab,ti,kf.)
S9	(Hot Flashes/ OR ('flush, hot' OR 'hot flash' OR 'hot flush' OR 'hot flushing' OR 'vasomotor symptom').ab,ti,kf.)
S10	(((sleep* OR sleeping*) ADJ3 ('disorder' OR 'problem' OR 'disturbance' OR 'disruption')) OR 'sleeplessness' OR 'insomnia' OR 'difficulty sleeping'.ab,ti,kf.)
S11	('heart discomfort' OR 'palpation' OR 'palpations'.ab,ti,kf.)
S12	(Headache/ OR ('headaches' OR 'migraine').ab,ti,kf.)
S13	(((muscle* OR muscular* OR joint* OR osteomuscular* OR shoulder* OR limb*) ADJ3 (pain OR ache OR discomfort OR aching OR stiff)) OR 'arthralgia' OR 'myalgia' OR 'backache'.ab,ti,kf.)
S14	(((physical* OR mental*) ADJ3 ('exhaustion')) OR 'fatigue' OR tired* OR 'lack of energy'.ab,ti,kf.)
S15	(Depression/ OR ('depressive disease' OR 'depressive disorder' OR 'depressive episode' OR 'depressive illness' OR 'depressive symptom' OR 'depressive syndrome' OR 'depressive mood' OR 'depressed').ab,ti,kf.)
S16	(Anxiety/ OR 'anxious'.ab,ti,kf.)
S17	(Irritable Mood/ OR 'irritability'.ab,ti,kf.)
S18	('mood swings'.ab,ti,kf.)
S19	((reduce* OR loss* OR decrease* OR change*) ADJ3 ('libido').ab,ti,kf.)
S20	(('vaginal dryness' OR 'dry vagina' OR 'vaginal dryness').ab,ti,kf.)
S21	((bladder* OR urinary*) ADJ2 (problem* OR symptom*).ab,ti,kf.)
S22	('poor memory' OR 'memory problem' OR 'forgetfulness' OR 'loss of memory'.ab,ti,kf.)
S23	('difficulty concentrating' OR 'difficulty concentration' OR 'lake of concentration' OR 'poor concentration' OR 'decrease concentration'.ab,ti,kf.)
S24	('formication'.ab,ti,kf.)
S25	('change in the appearance, texture, or tone of my skin'.ab,ti,kf.)
S26	('facial hair'.ab,ti,kf.)
S27	('drying skin'.ab,ti,kf.)
S28	(S8 OR S9 OR S10 OR S11 OR S12 OR S13 OR S14 OR S15 OR S16 OR S17 OR S18 OR S19 OR S20 OR S21 OR S22 OR S23 OR S24 OR S25 OR S26 OR S27)
S29	(S1 AND S7 AND S28)

Search strategy—Web of Science

1	TS=((epidemiolog* OR ((geograph* OR global*) NEAR/2 (distribut*)) OR (patient* NEAR/2 volume*) OR prevalen* OR incidenc* OR population-based* OR cross-sectional*) NOT ((animal* OR rat OR rats OR mouse OR mice OR murine OR dog OR dogs OR canine OR cat OR cats OR feline OR rabbit OR cow OR cows OR bovine OR rodent* OR sheep OR ovine OR pig OR swine OR porcine OR veterinar* OR chick* OR zebrafish* OR baboon* OR nonhuman* OR primate* OR cattle* OR goose OR geese OR duck OR macaque* OR avian* OR bird* OR fish*) NOT (human* OR patient* OR women OR woman OR men OR man)))
2	TS=(((('middle aged' OR 'middle age') OR ('premenopause' OR 'menopause, pre' OR 'pre menopause' OR 'premenopausal') OR ('climacterium' OR 'climacteric' OR

	'menopausal transition' OR 'perimenopausal' OR 'perimenopause') OR ('postmenopause' OR 'post menopause' OR 'postmenopausal')) AND ('female' OR 'females' OR 'woman' OR 'women')))
3	TS=((menopausal* OR climacterial* OR climacteric* OR climacterium* OR menopause* OR perimenopausal* OR postmenopausal* OR postmenopause*) NEAR/2 (disorder* OR syndrome* OR complaint* OR symptom* OR distress*))
4	TS=(('hot flush' OR 'hot flash' OR 'hot flushing' OR 'vasomotor symptom') OR (((sleep* OR sleeping*) NEAR/2 ('disorder' OR 'problem' OR 'disturbance' OR 'disruption')) OR 'sleeplessness' OR 'insomnia' OR 'difficulty sleeping') OR ('heart discomfort' OR palpation) OR (headache OR migraine) OR (((muscle* OR muscular* OR joint* OR osteomuscular* OR shoulder* OR limb*) NEAR/2 (pain OR ache OR discomfort OR aching OR stiff)) OR 'arthralgia' OR 'myalgia' OR 'backache'))
5	TS=((((physical* OR mental*) NEAR/3 ('exhaustion')) OR 'fatigue' OR tired* OR 'lack of energy') OR ('depression' OR 'depressive disease' OR 'depressive disorder' OR 'depressive episode' OR 'depressive illness' OR 'depressive symptom' OR 'depressive syndrome' OR 'depressive mood' OR 'depressed') OR ('anxiety' OR 'anxious') OR ('irritability' OR 'irritable mood') OR ('mood swings'))
6	TS=(((reduce* OR loss* OR decrease* OR change*) NEAR/3 ('libido')) OR ((bladder* OR urinary*) NEAR/2 (problem* OR symptom*)) OR ('vaginal dryness' OR 'dry vagina' OR 'vaginal dryness'))
7	TS=(('poor memory' OR 'memory problem' OR 'forgetfulness' OR 'loss of memory') OR ('difficulty concentrating' OR 'difficulty concentration' OR 'lake of concentration' OR 'poor concentration' OR 'decrease concentration') OR formication OR ('drying skin') OR ('change in the appearance, texture, or tone of my skin') OR ('facial hair'))
8	#3 OR #4 OR #5 OR #6 OR #7
9	#1 AND #2 AND #8

Search strategy—Cochrane

1	((epidemiolog* OR ((geograph* OR global*) NEAR/3 (distribut*)) OR (patient* NEAR/3 volume*) OR prevalen* OR incidenc* OR (population NEXT/1 based*) OR (cross NEXT/1 sectional*)):ab,ti,kw)
2	(((('middle aged' OR 'middle age') OR ('premenopause' OR 'menopause, pre' OR 'pre menopause' OR 'premenopausal') OR ('climacterium' OR 'climacteric' OR 'menopausal transition' OR 'perimenopausal' OR 'perimenopause') OR ('postmenopause' OR 'post menopause' OR 'postmenopausal')) AND ('female' OR 'females' OR 'woman' OR 'women')):ab,ti,kw)
3	(((menopausal* OR climacterial* OR climacteric* OR climacterium* OR menopause* OR perimenopausal* OR postmenopausal* OR postmenopause*) NEAR/4 (disorder* OR syndrome* OR complaint* OR symptom* OR distress*)):ab,ti,kw)
4	((('hot flush' OR 'flush, hot' OR 'hot flash' OR 'hot flashes' OR 'hot flushing') OR ('vasomotor symptoms' OR 'vasomotor symptom') OR (((sleep* OR sleeping*) NEAR/3 ('disorder' OR 'problem' OR 'disturbance' OR 'disruption')) OR 'sleeplessness' OR 'insomnia' OR 'difficulty sleeping') OR ('heart discomfort' OR 'palpation' OR 'palpations') OR ('headache' OR 'headaches' OR 'migraine') OR (((muscle* OR

	muscular* OR joint* OR osteomuscular* OR shoulder* OR limb*) NEAR/2 (pain OR ache OR discomfort OR aching OR stiff)) OR 'arthralgia' OR 'myalgia' OR 'backache')):ab,ti,kw)
5	(((((physical* OR mental*) NEAR/3 ('exhaustion')) OR 'fatigue' OR tired* OR 'lack of energy') OR ('depression' OR 'depressive disease' OR 'depressive disorder' OR 'depressive episode' OR 'depressive illness' OR 'depressive symptom' OR 'depressive syndrome' OR 'depressive mood' OR 'depressed') OR ('anxiety' OR 'anxious') OR ('irritability' OR 'irritable mood') OR ('mood swings')):ab,ti,kw)
6	((((reduce* OR loss* OR decrease* OR change*) NEAR/3 ('libido')) OR ((bladder* OR urinary*) NEAR/2 (problem* OR symptom*)) OR ('vaginal dryness' OR 'dry vagina' OR 'vaginal dryness')):ab,ti,kw)
7	((('poor memory' OR 'memory problem' OR 'forgetfulness' OR 'loss of memory') OR ('difficulty concentrating' OR 'difficulty concentration' OR 'lake of concentration' OR 'poor concentration' OR 'decrease concentration') OR('formication') OR ('drying skin') OR ('change in the appearance, texture, or tone of my skin') OR ('facial hair')):ab,ti,kw)
8	#3 OR #4 OR #5 OR #6 OR #7
9	#1 AND #2 AND #8

R code

```
install.packages(c("metafor", "meta"))
library(metafor)
library(meta)
dat <- read.csv("data.csv", header = T, sep = ",")
ies <- escalc(xi = cases, ni = total, data = dat, measure = "PFT")
pes.da <- rma(yi, vi, data = ies, method="REML")
pes <- predict(pes.da, transf = transf.ipft.hm, targ = list(ni = dat$total))
print(pes)
print(pes.da, digits = 4)
confint(pes.da, digits = 2)
pes.summary <- metaprop(cases, total, authoryear, data = dat, sm = "PFT")
forest(pes.summary)
metarate<-metaprop(cases, total, author, data=dat, sm="PFT", incr=0.5, allincr=TRUE,
addincr=FALSE, title="",byvar=country,print.byvar=TRUE)
forest(metarate)
```

Supplementary Table 1. Characteristics for including studies.

<table>
<tr><th>Author</th><th>Country</th><th>P. year</th><th>Study year</th><th>Quality</th><th>Diagnosis criteria</th><th>Sample Source</th><th>Study design</th></tr>
<tr><td>Lan, et al</td><td>China</td><td>2017</td><td>2014</td><td>8</td><td>KMI</td><td>Community</td><td>cross-sectional</td></tr>
<tr><td>Adhikari, et al</td><td>Nepal</td><td>2022</td><td>2017</td><td>8</td><td>MRS</td><td>Community</td><td>cross-sectional</td></tr>
<tr><td>Wang, et al</td><td>China</td><td>2021</td><td>2018</td><td>7</td><td>KMI</td><td>Community</td><td>cross-sectional</td></tr>
<tr><td>Yang, et al</td><td>China</td><td>2017</td><td>2016-2017</td><td>8</td><td>KMI</td><td>Health check-up</td><td>Cohort</td></tr>
<tr><td>Olaolorun, et al</td><td>Nigeria</td><td>2009</td><td></td><td>7</td><td>MRS</td><td>Community</td><td>cross-sectional</td></tr>
<tr><td>Chedraui, et al</td><td>Ecuador</td><td>2010</td><td>2009</td><td>8</td><td>MRS</td><td>Community</td><td>cross-sectional</td></tr>
<tr><td>Li, et al</td><td>China</td><td>2012</td><td>2010-2011</td><td>7</td><td>KMI</td><td>Community</td><td>cross-sectional</td></tr>
<tr><td>Zagalaz-Anula, et al</td><td>Spain</td><td>2019</td><td>2016-2017</td><td>8</td><td>MRS</td><td>Health check-up</td><td>cross-sectional</td></tr>
<tr><td>Javadivala, et al</td><td>Iran</td><td>2020</td><td>2017</td><td>7</td><td>MRS</td><td>Health check-up</td><td>Cohort</td></tr>
<tr><td>Nagaraj, et al</td><td>India</td><td>2021</td><td>2019</td><td>7</td><td>MRS</td><td>Health check-up</td><td>cross-sectional</td></tr>
<tr><td>Yisma, et al</td><td>Ethiopia</td><td>2017</td><td>2015</td><td>8</td><td>MRS</td><td>Community</td><td>cross-sectional</td></tr>
<tr><td>Korkmaz, et al</td><td>Turkey</td><td>2022</td><td></td><td>8</td><td>MRS</td><td>Health check-up</td><td>Cohort</td></tr>
<tr><td>AlQuaiz, et al</td><td>Saudi</td><td>2013</td><td>2010</td><td>8</td><td>MRS</td><td>Health check-up</td><td>cross-sectional</td></tr>
<tr><td rowspan="3">Yu, et al</td><td>China</td><td rowspan="3">2022</td><td rowspan="3"></td><td>8</td><td>Others</td><td rowspan="3">Community</td><td rowspan="3">cross-sectional</td></tr>
<tr><td>Korea</td><td>8</td><td>Others</td></tr>
<tr><td>Taiwan</td><td>8</td><td>Others</td></tr>
<tr><td rowspan="4">Genazzani, et al</td><td>UK</td><td rowspan="4">2006</td><td rowspan="4">2004-2005</td><td>8</td><td>Face-to-face interview</td><td rowspan="4">Community</td><td rowspan="4">cross-sectional</td></tr>
<tr><td>France</td><td>8</td><td>Face-to-face interview</td></tr>
<tr><td>Germany</td><td>8</td><td>Face-to-face interview</td></tr>
<tr><td>Belgium</td><td>8</td><td>Face-to-face</td></tr>
</table>

					interview		
	Netherlands			8	Face-to-face interview		
	Switzerland			8	Face-to-face interview		
	Spain			8	Face-to-face interview		
Aaron, et al	India	2002		8	SDQ-9 for depression	Community	cross-sectional
Abdel-Salam, et al	Saudi	2021	2020	8	MRS	Health check-up	cross-sectional
Anderson, et al	Australia	2004	1999	6	the Greene Climacteric Scale	electoral roles	cross-sectional
	Japan		1998	6	Others		
Shafie, et al	Oman	2011	2010	8	MRS	Health check-up	cross-sectional
Aloufi, et al	Saudi	2022	2022	8	MRS	Health check-up	cross-sectional
Blümel, et al	12 Latin American countries:Argentina, Bolivia, Chile, Colombia,Cuba, Dominican Republic, Ecuador, Mexico, Panama, Peru, Uruguay, Venezuela	2012	2006-2007	8	MRS	Health check-ups	cross-sectional
Chou, et al	Macau, China	2014	2011	8	MRS	Health check-up	cross-sectional
Fallahzadeh, et al	Iran	2007	2006	8	Face-to-face	Community	cross-sectional

					interview		
Fuh, et al	Taiwan	2003	1998	8	KMI	Community	cross-sectional
Chedraui, et al	Ecuador	2014	2011-2012	8	MRS	newspaper advertising	cross-sectional
Chedraui, et al	Ecuador	2010	2007	8	MRS	Health check-up	cross-sectional
Ojeda, et al	Peru	2014		8	MRS	Health check-up	cross-sectional
Ikeda, et al	Japan	2005	2001-2004	8	The Keio questionnaire	Health check-up	cross-sectional
Ishizuka, et al	Japan	2008	1998	8	the simplified menopause index (SMI)	Community	cross-sectional
Kapur, et al	India	2009		8	the Greene Climacteric Scale	Community	Cohort
Sharma, et al	Nepal	2021	2019	8	MRS	Community	cross-sectional
Qazi, et al	Pakistan	2006		8	Others	Community	cross-sectional
Rahman, et al	Malaysia	2010	2007	8	MRS	Health check-up	cross-sectional
Shea, et al	China	2006		8	Face-to-face interview	community	cross-sectional
Som, et al	India	2012		8	MENQOL	Community	cross-sectional
Alwi, et al	Malaysia	2009	2007-2008	8	MENQOL	Community	cross-sectional
Waidyasekera, et al	Sri Lanka	2009		8	MRS	Community	cross-sectional
Tomida, et al	Japan	2021		8	KMI	Community	cross-sectional
He, et al	Australia	2021	2018-2019	8	MRS	Health check-up	Cohort
Kumari,et al	India	2020	2016-2017	8	MRS	Community	cross-sectional
Malacara, et al	Mexico	2002		8	Face-to-face interview	Community	cross-sectional

Silva, et al	Brazil	2013		7	MRS	Health check-up	cross-sectional
Shringarpure, et al	India	2022		8	MRS	Health check-up	cross-sectional
Martínez, et al	Spain	2013	2006	8	KMI	Community	cross-sectional
Andac, et al	Turkey	2017	2015	7	Menopause Rating Scale (MRS), Female Sexual Function Index (FSFI), and Sexual Satisfaction Scale for Women (SSS-W)	Community	cross-sectional
Yang, et al	China	2008	2003-2004	8	Face-to-face interview	Community	cross-sectional
Yim, et al	Korea	2015	2012-2013	7	MENQOL	Health check-up	cross-sectional
Yokota, et al	Japan	2016	1993-2014	8	The Keio questionnaire	Health check-up	Cohort
Gast, et al	Netherlands	2008	1994-1995	7	Others	population-based	cross-sectional
Du, et al	China	2020		8	KMI	Community	cross-sectional
Buhling, et al	Germany	2014	2012	7	MRS	Community	cross-sectional
Singh, et al	India	2014	2011-2013	8	MRS	population-based	cross-sectional
Kulkarni, et al	India	2016	2015	7	MRS	community	cross-sectional
Borker, et al	India	2013	2009	8	Face-to-face interview	community	cross-sectional
Rathnayake, et al	Sri Lanka	2019	2015-2017	7	MRS	community	cross-sectional
Kwon, et al	Korea	2016	2010-2012	8	MRS	Health check-up	cross-sectional
Baquedano, et al	Spain	2023	2021	8	Others	population-based	cross-sectional
Brzyski, et al	USA	2001		8	MENQOL	Health check-up	cross-sectional

Hwang, et al	Korea	2021	2016	7	PSQI, PHQ-9	population-based	cross-sectional
Ahmadieh, et al	Lebanon	2021	2016-2017	8	Face-to-face interview	population-based	cross-sectional
Namgoung, et al	Korea	2022	2014-2018	6	MENQOL	Health check-up	cross-sectional
Pateliya, et al	India	2022	2022	9	Others		cross-sectional
Srinivasan, et al	India	2021	2020	9	MRS	Health check-up	cross-sectional
Chim, et al	Singapore	2002	2000	8	Face-to-face interview, CES-D8 for depression	community	cross-sectional
Dhillon,et al	Malaysia	2006	2002-2003	8	Others	population-based	cross-sectional
Li, et al	China	2008	2004	8	The Zung Self-rating Depression Scale (SDS),The Zung Self-rating Anxiety Scale (SAS)	community	cross-sectional
Santoro，et al	USA	2009	2008	6	Others	population-based	cross-sectional
Chuni, et al	Nepal	2011	2008	8	MRS	Health check-up	cross-sectional
Valadares, et al	Brazil	2011	2005	7	Short Personal Experiences Questionnaire (SPEQ)	population-based	cross-sectional
Nisar, et al	Pakistan	2015	2007-2008	8	MRS	community	cross-sectional
Zhang, et al	China	2016	2014	8	MRS	community	cross-sectional
Agaba, et al	Nigeria	2017		7	MRS	Health check-up	Cohort
AlQuaiz, et al	Saudi	2017	2016	8	MRS	Health check-up	cross-sectional
Grigoriou, et al	Greek	2013		7	the Greene	Health check-up	cross-sectional

					Climacteric Scale		
Hunter, et al	UK	2012	2001-2005	8	Hot Flush Rating Scale	community	cross-sectional
Haines, et al	China	2005		8	Others	community	Cohort
	the Philippines			8	Others		
	Republic of Indonesia			8	Others		
	Korea			8	Others		
	Malaysia			8	Others		
	Pakistan			8	Others		
	Taiwan			8	Others		
	Thailand			8	Others		
	Vietnam			8	Others		
Melby, et al	Japan	2005	2005	6	Others	community	cross-sectional
Dennerstein, et al	Australia	2000		6	Face-to-face interview	population-based	Cohort
Nappi, et al	UK	2008	2008	8	Face-to-face interview	computer-assisted telephone interviewing.	cross-sectional
	France			8	Face-to-face interview		
	Germany			8	Face-to-face interview		
	Italy			8	Face-to-face interview		
	Netherlands			8	Face-to-face interview		

	Switzerland			8	Face-to-face interview		
Gotmar, et al	Sweden	2008	1995-2000	8	Others	population-based	cross-sectional
Górecka, et al	Poland	2022	2018-2019	8	Others	community	cross-sectional
Saied, et al	Iraq	2021	2020	8	MRS	Health check-up	cross-sectional
Sheereen, et al	India	2022	2021	8	MENQOL	Community	cross-sectional
Kang, et al	India	2021	2019	8	MENQOL	community	cross-sectional
Senthilvel, et al	India	2018	2018	8	MENQOL	Health check-up	cross-sectional
Karmakar, et al	India	2017	2014	8	MENQOL	community	cross-sectional
Ganapathy, et al	India	2018	2016	8	MENQOL	Health check-up	cross-sectional
Sripraser, et al	Thailand	2017	2015	8	Hot Flush Rating Scale	population-based	cross-sectional
Tan, et al	Turkey	2014	2009	8	MRS	Health check-up	cross-sectional
Chopra, et al	India	2022	2021	8	MRS	community	cross-sectional
Vaccaro, et al	Italy	2021		8	Face-to-face interview	community	cross-sectional
Hachul, et al	Brazil	2021	2007	8	Others	community	cross-sectional
Saú, et al	Brazil	2020	2014	8	KMI	community	cross-sectional
Smith, et al	USA	2015	2006-2015	8	Others	community	Cohort
Zhao, et al	China	2023	2017-2019	8	MRS	community	cross-sectional
Han, et al	China	2023	2012-2013	8	KMI	community	cross-sectional
Ryu, et al	Korea	2022	2013-2016	8	MRS	Health check-up	cross-sectional
Salin, et al	Finland	2022	2012	8	Others	population-based	cross-sectional
Thakur, et al	India	2022		8	Others	community	cross-sectional
Tijerina, et al	Mexico	2022	2015-2017	8	Others	telephone	cross-sectional

Koçoğlu, et al	Turkey	2022	2018-2019	8	MRS	Health check-up	cross-sectional
Arakane, et al	Ecuador	2011	2010	8	MRS	Health check-up	cross-sectional
AlDughaither, et al	Saudi	2015	2010	8	MRS	Health check-up	cross-sectional
Ceylan, et al	Turkey	2014	2009	8	MENQOL	Community	cross-sectional
Thakur,et al	India	2019		8	the Greene Climacteric Scale	Community	cross-sectional
Gallicchio, et al	USA	2005	2000-2004	8	Others	population-based	cross-sectional
Kim, et al	Korea	2017	2015	8	Others	population-based	cross-sectional
Sievert, et al	USA	2022	2020-2021	8	Face-to-face interview, PHQ-9 for depression	community	cross-sectional
Gerber, et al	USA	2018	1999-2003	8	Face-to-face interview	fliers and word of mouth	cross-sectional
Silvestrin, et al	USA	2016	2014	7	WHAT Questionaire(patient health questionnaire-9 (PHQ-9), the generalized anxiety disorder-2 (GAD-2) scale, 3 incontinence questions (3IQ), as well as a few questions from the female sexual	community	cross-sectional

					function index-6 item (FSFI-6))		
Ghazanfarpour, et al	Iran	2016		8	MENQOL	Health check-up	cross-sectional
Duffy, et al	UK	2012	2009	8	Others	community	cross-sectional
Loutfy, et al	Egypt	2006	2002	8	Face-to-face interview	community	cross-sectional
Delavar, et al	Iran	2011		8	Face-to-face interview	community	cross-sectional
Chedraui, et al	Ecuador	2007	2005	6	MENQOL	screening program was advertised through a newspaper	cross-sectional
Chedraui, et al	Ecuador	2012	2010-2011	6	MRS	community	cross-sectional
Chedraui, et al	Ecuador	2014	2013	6	CS-10	community	cross-sectional
Suka, et al	Japan	2010	2008	8	the simplified menopause index (SMI)	population-based	cross-sectional
Rahman, et al	Bangladesh	2011	2010	8	MRS	Health check-up	cross-sectional
Singh, et al	India	2012		8	MRS	Health check-up	cross-sectional
Aguilar-Zavala, et al	Mexico	2012		8	Face-to-face interview	population-based	cross-sectional
Agwu, et al	Nigeria	2008		8	Others	population-based	cross-sectional
Ahmed, et al	Bangladesh	2016	2013-2014	8	MENQOL	population-based	cross-sectional
Gartoulla, et al	Australia	2016	2013-2014	8	MENQOL	population-based	cross-sectional
Berecki-Gisolf, et al	Australia	2009	1996	8	Face-to-face interview, 36-item	population-based	Cohort

					Short-Form Health Survey subscale		
Bener, et al	Qatar	2014	2012-2013	8	MENQOL	Health check-up	cross-sectional
Bener, et al	United Arab Emirates	2000	1999	8	MENQOL	Health check-up	cross-sectional
Im, et al	USA	2010		8	the Midlife Women's Symptom Index	Internet survey	cross-sectional
Williams, et al	USA	2008		8	MENQOL	population-based	cross-sectional
Ande, et al	Nigeria	2011	2008	8	Face-to-face interview	Health check-up	cross-sectional
Andenæs, et al	Norway	2020	2006-2008	8	Face-to-face interview and the Insomnia Index	population-based	cross-sectional
Khatoon, et al	Pakistan	2018		8	MRS	Health check-up	cross-sectional
Budakoğlu, et al	Turkey	2007	2005	8	MRS	community	cross-sectional
Sharanya Shre, et al	India	2016	2013	8	MRS	Health check-up	cross-sectional
Damodaran, et al	Malaysia	2000	1996-1997	8	Others	Health check-up	cross-sectional
Zeleke, et al	Australia	2016		8	MENQOL	population-based	cross-sectional
Masoudi, et al	Iran	2021	2018-2019	8	MRS, Insomnia Severity Index (ISI)	Health check-up	cross-sectional
Beura, et al	India	2020	2019	8	MRS	population-based	cross-sectional
Thapa, et al	Cambodia	2020	2017	8	MRS	community	cross-sectional
Ibrahim, et al	Egypt	2015	2009-2013	8	MRS	Health check-up	cross-sectional
Nusrat, et al	Pakistan	2008	2005-2006	8	Face-to-face	Health check-up	cross-sectional

					interview		
Bhavani, et al	India	2022	2018-2019	8	Face-to-face interview	community	cross-sectional
Liu, et al	Australia	2007		8	MENQOL	community	cross-sectional
Fooladi, et al	Iran	2018	2016-2017	7	MENQOL	community	cross-sectional
Lee, et al	Taiwan	2020	2006-2016	8	Others	Health check-up	cross-sectional
Modoodi, et al	Iran	2020	2014	7	Others	Health check-up	cross-sectional
Kalhan, et al	India	2020	2018-2019	8	MRS	community	cross-sectional
Rindner, et al	Sweden	2017	2009-2010	7	MRS	Health check-up	cross-sectional
Al-Musa, et al	Saudi	2017	2016	8	MRS	Health check-up	cross-sectional
Moilanen, et al	Finland	2010	2000-2001	8	Face-to-face interview	population-based	cross-sectional
Bairy, et al	India	2009	2006	8	MENQOL	Health check-up	cross-sectional
Heinemann, et al	Europe(France, Germany, Spain, Sweden)	2008		8	MRS	population-based	cross-sectional
	USA			8	MRS		
	Latin America(Argentina, Brazil, Mexico)			8	MRS		
	Republic of Indonesia			8	MRS		
Uncu, et al	Turkey	2007	2004-2005	6	Others	population-based	cross-sectional
Lawton, et al	New Zealand	2008	1999-2004	6	the Greene Climacteric Scale	Health check-up	cross-sectional
Liu, et al	Australia	2008		8	MENQOL	Community	cross-sectional

Liu, et al	Australia	2007	2005	8	MENQOL	Community	cross-sectional
Saccomani, et al	Brazil	2017	2012-2013	8	MRS	population-based	cross-sectional
Im, et al	USA	2009		8	the Midlife Women's Symptom Index	Community	cross-sectional
Resmi, et al	India	2020	2015	8	Face-to-face interview	community	cross-sectional
Ashrafi, et al	Iran	2009	2004-2005	8	Others	population-based	cross-sectional
Ayranci, et al	Turkey	2010	2008	8	the Greene Climacteric Scale	population-based	cross-sectional
Deveci, et al	Turkey	2010	2006	7	the Beck Depression Inventory (BDI)	population-based	cross-sectional
Sagdeo, et al	India	2011	2007-2009	9	Others	community	cross-sectional
Oğurlu, et al	Turkey	2011	2007-2008	9	The Climacteric Complaint Tool	Health check-up	cross-sectional
Wang, et al	China	2021	2018	8	PHQ-9, GAD-7	Community	cross-sectional
Anolue, et al	Nigeria	2012	2009	6	Others	community	cross-sectional
Mahajan,et al	India	2012	2007-2008	8	twenty eight item General Health Questionnaire	community	cross-sectional
Mustafa, et al	Iraq	2012	2010-2011	8	Others	Health check-up	cross-sectional
Nappi, et al	Great Britain, the United States, Canada, Sweden, Denmark, Finland,	2012	2010	8	Others	email	cross-sectional

	and Norway						
Sassoon, et al	South Africa	2014		7	the Greene Climacteric Scale	telephone screening	cross-sectional
Liu, et al	China	2013	2008	8	KMI	community	cross-sectional
Mohamed, et al	Saudi	2014		9	MENQOL	Health check-up	cross-sectional
Hashemzadeh, et al	Iran	2019	2017-2018	9	Saba questionnaire	Health check-up	cross-sectional
Hestiantoro, et al	Indonesian	2019	2016	8	Face-to-face interview		cross-sectional
Kong, et al	China	2019		8	MRS	Health check-up	cross-sectional
Sharifi, et al	Iran	2019	2013-2014	8	MRS	community	cross-sectional
Taavoni, et al	Iran	2015	2010-2012	8	PSQI	community	cross-sectional
Holm, et al	USA	2000	1999	8	WHAS	community	cross-sectional
Pan, et al	Taiwan	2002		8	Others	population-based	cross-sectional
Kasuga, et al	Japan	2004	1993-1999	8	The Keio questionnaire	menopause clinic	cross-sectional
Kaur, et al	India	2004	1998-2000	8	Face-to-face interview	community	cross-sectional
Yahya, et al	Pakistan	2002		8	Face-to-face interview	population-based	cross-sectional
Gupta, et al	UK	2006		8	WHQ WRQ	community	cross-sectional
	India			8	WHQ WRQ		
Inayat, et al	Pakistan	2017		8	Face-to-face interview	Health check-up	cross-sectional
Mathialagan, et al	Malaysia	2022	2020	8	MRS	community	cross-sectional
Canário, et al	Brazil	2012	2011	8	Batt-Kupperman	population-based	cross-sectional

					Menopausal Index		
Rahman, et al	Bangladesh	2020	2017-2018	8	MRS	Health check-up	cross-sectional
Abedzadeh-Kalahroudi, et al	Iran	2012		8	MENQOL	community	cross-sectional
Diyu, et al	Indonesian	2022		8	MRS	community	cross-sectional
Zhang, et al	China	2016	2012-2013	8	KMI	community	cross-sectional
Ryu, et al	Korea	2020	2010-2012	8	MRS	Health check-up	cross-sectional
Ma, et al	China	2017	2014	8	KMI	community	cross-sectional
Fu, et al	China	2020	2018	8	KMI	community	cross-sectional
Liu, et al	China	2018		8	KMI	community	cross-sectional
Li, et al	China	2016	2014	8	KMI	community	cross-sectional
Joshi, et al	India	2015		8	MRS	community	cross-sectional
Ghimire, et al	Nepal	2015		8	MRS	community	cross-sectional
Joseph, et al	India	2014	2011	8	MRS	community	cross-sectional
Dasgupta, et al	India	2015		8	Face-to-face interview	community	cross-sectional
Sievert, et al	Bangladesh	2008	2007	8	Face-to-face interview	community	cross-sectional
Bindhu, et al	India	2014		8	the Greene Climacteric Scale	community	cross-sectional
Srivastava, et al	India	2014		8	MRS	community	cross-sectional
ROKHADE, et al	India	2013		8	Others	Health check-up	cross-sectional
Sun, et al	China	2014	2012-2013	8	KMI	Health check-up	cross-sectional
Wong, et al	Singapore	2022	2014-2016	7	PSQI, MRS, GAD, Centre for	Health check-up	cross-sectional

					Epidemiological Studies Depression Scale, the Pelvic Floor Disability Index		
Santo, et al	Spain and Portugue	2023	2020-2022	8	MRS, HADS	community	cross-sectional
Xu, et al	China	2016		7	Face-to-face interview , PSQI,HADS	community	cross-sectional
Lerner-Geva, et al	Israel	2010	2004	8	the Greene Climacteric Scale	community	cross-sectional
Sweed, et al	Egypt	2012	2008-2009	8	MRS	community	cross-sectional
Taher, et al	Libya	2012	2008-2009	8	MENQOL	community	cross-sectional
Ryan, et al	Australia	2009	2004	8	the Center for Epidemiological Studies Depression Scale, Validated Questionnaire	population-based	cross-sectional
Beigi, et al	Iran	2012		8	Others	Health check-up	cross-sectional
G K, et al	India	2013	2012	8	MENQOL	Health check-up	cross-sectional
Bernis, et al	Spain	2007	1996-1998,2002-2003	8	Others	Health check-up	cross-sectional
Dibonaventura, et al	France, Germany, Italy, Spain, and the United Kingdom	2013	2010	8	MRS	population-based	cross-sectional
Freeman, et al	USA	2009		8	CES-D	population-based	cross-sectional

Seib, et al	Australia	2014	2011	8	General Sleep Disturbance Scale [GSDS	electoral roll	cross-sectional
Chedraui, et al	Ecuador	2007		8	MRS	Health check-up	cross-sectional
Ahlawat, et al	India	2019		8	HAM-D	community	cross-sectional
Bansal, et al	India	2015		8	The Zung Self-rating Depression Scale (SDS),The Zung Self-rating Anxiety Scale (SAS)	community	cross-sectional
Dutta, et al	India	2018	2017	7	Others	community	cross-sectional
Nayak, et al	India	2019	2017-2018	8	IDS-SR	community	cross-sectional
Ps, et al	India	2017		8	PHQ-9	community	cross-sectional
Alam, et al	Bangladesh	2020		7	PHQ-9	population-based	cross-sectional
Shin, et al	Korea	2005		8	Questionnaire,BDI	population-based	Cohort
Fabbrini, et al	Italy	2015		8	PSQI,BDI	Health check-up	cross-sectional
Luo, et al	China	2020	2005	8	HADS	community	cross-sectional
Geng, et al	China	2018	2015-2016	8	Face-to-face interview	Health check-up	cross-sectional
Moral, et al	Spain	2018	2015	8	Others	Health check-up	cross-sectional
Ahmed, et al	Iraq	2013	2011	8	Face-to-face interview	Health check-up	cross-sectional
Xu, et al	China	2020	2018	8	ICIQ-SF	population-based	cross-sectional
Islam, et al	Bangladesh	2018	2013-2014	7	the Questionnaire for Urinary Incontinence	population-based	cross-sectional

					Diagnosis (QUID)		
Yagmur, et al	Turkey	2021	2017	8	the Incontinence Quality of Life Questionnaire	community	cross-sectional
Chung, et al	Hong Kong	2006		8	the Greene Climacteric Scale	community	cross-sectional
Hsu, et al	Taiwan	2005		8	PSQI, The 18-item Taiwanese Depression Scale	community	cross-sectional
Ward, et al	UK	2010	2006	7	Others	population-based	cross-sectional
Pandey, et al	Nepal	2020	2017-2018	8	MRS	Health check-up	cross-sectional
Abdelaziz, et al	Saudi	2022	2021	8	MRS, PSQI	Health check-up	cross-sectional
Liao, et al	China	2019	2013-2016	8	The Zung Self-rating Depression Scale (SDS)	Health check-up	cross-sectional
Sievert, et al	USA	2007	2004	8	Others	community	cross-sectional
Sievert, et al	Mexico	2003	1999-2000	8	Others	community	cross-sectional
Obermeyer, et al	USA	2004	2001-2002	8	Face-to-face interview	community	cross-sectional
Keenan, et al	USA	2003	1997-1998	8	Face-to-face interview	telephone	cross-sectional
Jansson, et al	Sweden	2003	1999	7	Others	community	cross-sectional
Zhao, et al	China	2000		8	Batt-Kupperman Menopausal Index	community	cross-sectional
Lam, et al	Hong Kong	2003	1999-2000	8	the Greene Climacteric Scale	population-based	cross-sectional

Gollschewski, et al	Australia	2004	2001	8	the Greene Climacteric Scale	community	cross-sectional
Biri, et al	Turkey	2005	2002	7	Face-to-face interview	Health check-up	cross-sectional
Obermeyer, et al	Morocco	2002	1995	8	Face-to-face interview	face to face interview	cross-sectional
Lin, et al	Taiwan	2012	2002	8	Taiwanese Depression Questionnaire	population-based	cross-sectional
Loh, et al	Singapore	2005	2001	8	Face-to-face interview	population-based	cross-sectional
Chaopotong, et al	Thailand	2005	2005	7	Others	community	cross-sectional
Zhu, et al	China	2022	2016	8	Vaginal health index score and urinary distress inventory (UDI-6)	community	cross-sectional
Huang, et al	China	2010	2006	8	Face-to-face interview	community	cross-sectional
	Malaysia			8	Face-to-face interview		
	Taiwan			8	Face-to-face interview		
	Thailand			8	Face-to-face interview		
	Hong Kong			8	Face-to-face interview		

Hunter, et al	Spain	2012	2009-2010	8	Hot Flush Rating Scale	population-based	cross-sectional
	Panama			8	Hot Flush Rating Scale		
	Chile			8	Hot Flush Rating Scale		
	Ecuador			8	Hot Flush Rating Scale		
Anderson, et al	Australia	2004	2001	8	the Greene Climacteric Scale	electoral roles	cross-sectional
	Japan		2002	8	the Greene Climacteric Scale		
Glod, et al	USA	2006	1995-1997	8	Face-to-face interview	Community	cross-sectional
Afshari, et al	Iran	2015	2013-2014	7	Hamilton Depression Scale		
Goncalves, et al	Portugue	2013		8	Questionnaire,CES-D		
Onya, et al	Nigeria	2018	2017	8	CES-D		
Yen, et al	Taiwan	2009		8	CES-D	Community	cross-sectional
Timur, et al	Turkey	2010	2008	8	BDI	Community	cross-sectional
Zang, et al	China	2016		8	Questionnaire,SDS	Community	cross-sectional
Masjoudl, et al	Iran	2017	2013-2014	8	MRS	Community	cross-sectional
Kapoor, et al	USA	2021	2015-2016	7	GAD-7, PHQ-9	Health check-up	cross-sectional
Durairaj, et al	India	2022	2019	8	MRS	Community	cross-sectional
Bala, et al	India	2022		8	Face-to-face	population-based	cross-sectional

					interview		
Stefanopoulou, et al	India	2014	2010-2011	8	Hot Flush Rating Scale	population-based	cross-sectional
Abou-Raya, et al	Egypt	2016	2013-2014	7	MRS	Health check-up	cross-sectional
Krajewska-Ferishah, et al1	Poland	2022		8	BDI,Questionnaire	Health check-up	cross-sectional
Krajewska-Ferishah, et al2	Belarus	2022		8	BDI,Questionnaire	Health check-up	cross-sectional
Krajewska-Ferishah, et al3	Belgium	2022		8	BDI,Questionnaire	Health check-up	cross-sectional
Krajewska-Ferishah, et al4	Greece	2022		8	BDI,Questionnaire	Health check-up	cross-sectional
Ogwumike, et al	Nigeria	2012	2011	8	Others	Community	cross-sectional
Ornat, et al	Spain	2013	2012	8	CSFQ-14	Health check-up	cross-sectional
Wang, et al	Taiwan	2013	2008-2009	8	CES-D	Community	cross-sectional
Gao, et al	China	2013	2009-2010	8	Others	Community	cross-sectional
Sierra, et al	Ecuador	2004	2000	8	the Greene Climacteric Scale	Health check-up	cross-sectional
Castelo-Branco, et al	Bolivia	2005	2002	8	Face-to-face interview	Community	cross-sectional
Ahn, et al	Korea	2009	2006	8	IOF Quality of Life Questionnaire	newspaper advertising	cross-sectional
Jin, et al	China	2014	2011-2012	8	KMI	Health check-up	cross-sectional
Pérez-López, et al	Colombia	2013	2009-2011	8	CS-10	population-based	cross-sectional
Huang, et al	China	2020	2018-2019	8	KMI	Health check-up	cross-sectional
Dasgupta, et al	India	2012	2009-2010	8	Others	Community	cross-sectional

Huseth-Zosel, et al	China	2014	2008-2016	8	Face-to-face interview	Community	cross-sectional
Ornat, et al	Spain	2014	2012	8	Jenkins Sleep Scale	Health check-up	cross-sectional
Legorreta, et al	Mexico	2013	2010-2011	8	MRS	Health check-up	cross-sectional
Aydin, et al	Turkey	2013	2005-2012	8	Others	Health check-up	cross-sectional
Zhang, et al	China	2020		7	Others	Health check-up	cross-sectional
Wu, et al	China	2023	2022	8	KMI	Community	cross-sectional
Shea, et al	Canada	2020		8	Others	population-based	cross-sectional
Abdullah, et al	Malaysia	2016		8	MRS	Community	cross-sectional
Chu, et al	China	2022		7	KMI, HAMD	Health check-up	cross-sectional
Barghandan, et al	Iran	2021	2018	8	KMI, BDI, STAI	Health check-up	cross-sectional
Hickey, et al	Australia	2017	2015-2016	8	MRS	online and paper-based	cross-sectional
Blell, et al	UK	2014		8	Face-to-face interview	Community	cross-sectional
Dienye, et al	Nigeria	2013	2010	9	MRS	Health check-up	cross-sectional
Whiteley, et al	USA	2013	2005	7	Face-to-face interview	population-based	cross-sectional
Bener, et al	Qatar	2017	2012-2014	8	DASS	Health check-up	cross-sectional
Olowokere, et al	Nigeria	2020		8	MRS	Community	cross-sectional
Zhu, et al	China	2021	2013	8	Female Sexual Function Index (FSFI), the Greene Climacteric Scale	Health check-up	cross-sectional
Zhang, et al	China	2009		8	Face-to-face interview	Community	cross-sectional

Salazar-Pousada, et al1	Colombia	2017		8	CESD-10, Questionnaire	population-based	cross-sectional
Salazar-Pousada, et al2	Ecuador	2017		8	CESD-10, Questionnaire	population-based	cross-sectional
Salazar-Pousada, et al3	Peru	2017		8	CESD-10, Questionnaire	population-based	cross-sectional
Salazar-Pousada, et al4	Paraguay	2017		8	CESD-10, Questionnaire	population-based	cross-sectional
Whiteman, et al	USA	2003	2001	8	Others	email	cross-sectional
Ruan, et al	China	2017	2008-2015	8	KMI	Health check-up	cross-sectional
An, et al	Korea	2022	2015-2018	9	CESD,PSQI	Health check-up	cross-sectional
Devi, et al	USA	2005		8	Face-to-face interview	Community	cross-sectional
Li, et al	China	2019	2014-2015	8	PHQ-9	population-based	Cohort
Xu, et al	USA	2005	2000-2001	9	the Greene Climacteric Scale	Health check-up	cross-sectional
Yılmaz, et al	Turkey	2020	2019	8	BDI	Community	cross-sectional
Islam, et al	Bangladesh	2016	2013-2014	7	MENQOL	population-based	cross-sectional
Shakhatreh, et al	Jordan	2006	2003	8	Others	Community	cross-sectional
Obermeyer, et al	Lebanon	2007		8	Face-to-face interview	Community	cross-sectional
Al-Qutob, et al	Jordan	2000	1997	8	Face-to-face interview	Community	cross-sectional
Chae, et al	Korea	2014	2010	7	Others	Health check-up	cross-sectional
Veigh, et al	Australia	2005		8	WHAS	population-based	cross-sectional
Lampio, et al	Finland	2014		8	Basic Nordic Sleep	newspaper advertising	Cohort

					Questionnaire,BDI, Questionnaire		
Oppermann, et al	Brazil	2012	2001-2002	8	Face-to-face interview, SRQ-20	population-based	cross-sectional
An, et al	China	2023		9	KMI	population-based	cross-sectional
Kamal, et al	Egypt	2017	2015	9	MRS	Community	cross-sectional
Rajbhandari, et al	Nepal	2017	2014-2015	9	Others	Community	cross-sectional
Cortes, et al	Spain	2023	2020	7	Others	newspaper advertising	cross-sectional
Baig, et al	Pakistan	2006		8	Face-to-face interview	population-based	cross-sectional
Chalise, et al	Nepal	2022	2021	6	Face-to-face interview	Health check-up	cross-sectional

Supplementary Figure 1. Forest plot of prevalence of hot flashes among middle-aged women

Study	Events	Total	Proportion	95%−CI	Weight (common)	Weight (random)
Lan, et al 2017	391	985	0.40	[0.37; 0.43]	0.3%	0.3%
Adhikari, et al 2022	152	252	0.60	[0.54; 0.66]	0.1%	0.3%
Wang, et al 2021	1832	6364	0.29	[0.28; 0.30]	1.8%	0.3%
Yang, et al 2017	247	589	0.42	[0.38; 0.46]	0.2%	0.3%
Olaolorun, et al 2009	464	1189	0.39	[0.36; 0.42]	0.3%	0.3%
Chedraui, et al 2010	646	1154	0.56	[0.53; 0.59]	0.3%	0.3%
Li, et al 2012	6000	20275	0.30	[0.29; 0.30]	5.8%	0.3%
Zagalaz−Anula, et al 2019	128	278	0.46	[0.40; 0.52]	0.1%	0.3%
Javadivala, et al 2020	105	161	0.65	[0.57; 0.73]	0.0%	0.3%
Nagaraj, et al 2021	76	200	0.38	[0.31; 0.45]	0.1%	0.3%
Yisma, et al 2017	149	226	0.66	[0.59; 0.72]	0.1%	0.3%
Korkmaz, et al 2022	101	108	0.94	[0.87; 0.97]	0.0%	0.3%
AlQuaiz, et al 2013	348	490	0.71	[0.67; 0.75]	0.1%	0.3%
Yu, et al1 2022	1384	1588	0.87	[0.85; 0.89]	0.5%	0.3%
Yu, et al2 2022	744	1000	0.74	[0.72; 0.77]	0.3%	0.3%
Yu, et al3 2022	581	773	0.75	[0.72; 0.78]	0.2%	0.3%
Genazzani, et al1 2006	501	611	0.82	[0.79; 0.85]	0.2%	0.3%
Genazzani, et al2 2006	456	600	0.76	[0.72; 0.79]	0.2%	0.3%
Genazzani, et al3 2006	399	596	0.67	[0.63; 0.71]	0.2%	0.3%
Genazzani, et al4 2006	487	594	0.82	[0.79; 0.85]	0.2%	0.3%
Genazzani, et al5 2006	456	600	0.76	[0.72; 0.79]	0.2%	0.3%
Genazzani, et al6 2006	450	600	0.75	[0.71; 0.78]	0.2%	0.3%
Genazzani, et al7 2006	426	600	0.71	[0.67; 0.75]	0.2%	0.3%
Aaron, et al 2002	69	200	0.34	[0.28; 0.42]	0.1%	0.3%
Abdel−Salam, et al 2021	600	845	0.71	[0.68; 0.74]	0.2%	0.3%
Anderson, et al1 2004	323	712	0.45	[0.42; 0.49]	0.2%	0.3%
Anderson, et al2 2004	658	1430	0.46	[0.43; 0.49]	0.4%	0.3%
Shafie, et al 2011	202	472	0.43	[0.38; 0.47]	0.1%	0.3%
Aloufi, et al 2022	248	361	0.69	[0.64; 0.73]	0.1%	0.3%
Bl..mel, et al 2012	3374	6079	0.56	[0.54; 0.57]	1.7%	0.3%
Chou, et al 2014	251	442	0.57	[0.52; 0.61]	0.1%	0.3%
Fallahzadeh, et al 2007	255	346	0.74	[0.69; 0.78]	0.1%	0.3%
Fuh, et al 2003	158	1360	0.12	[0.10; 0.13]	0.4%	0.3%
Chedraui, et al 2014	117	204	0.57	[0.50; 0.64]	0.1%	0.3%
Chedraui, et al 2010	268	404	0.66	[0.61; 0.71]	0.1%	0.3%
Ojeda, et al 2014	638	771	0.83	[0.80; 0.85]	0.2%	0.3%
Ikeda, et al 2005	151	390	0.39	[0.34; 0.44]	0.1%	0.3%
Ishizuka, et al 2008	533	1169	0.46	[0.43; 0.49]	0.3%	0.3%
Kapur, et al 2009	43	129	0.33	[0.25; 0.42]	0.0%	0.3%
Sharma, et al 2021	131	203	0.65	[0.58; 0.71]	0.1%	0.3%
Qazi, et al 2006	444	800	0.56	[0.52; 0.59]	0.2%	0.3%
Rahman, et al 2010	148	356	0.42	[0.36; 0.47]	0.1%	0.3%
Shea, et al 2006	42	399	0.11	[0.08; 0.14]	0.1%	0.3%
Som, et al 2012	223	250	0.89	[0.85; 0.93]	0.1%	0.3%
Alwi, et al 2009	117	276	0.42	[0.36; 0.48]	0.1%	0.3%
Waidyasekera, et al 2009	267	683	0.39	[0.35; 0.43]	0.2%	0.3%
Tomida, et al 2021	573	1152	0.50	[0.47; 0.53]	0.3%	0.3%
He, et al 2021	123	334	0.37	[0.32; 0.42]	0.1%	0.3%
Kumari,et al 2020	144	198	0.73	[0.66; 0.79]	0.1%	0.3%
Malacara, et al 2002	2640	7635	0.35	[0.34; 0.36]	2.2%	0.3%
Silva, et al 2013	886	1415	0.63	[0.60; 0.65]	0.4%	0.3%
Shringarpure, et al 2022	120	290	0.41	[0.36; 0.47]	0.1%	0.3%
Mart..nez, et al 2013	5409	10514	0.51	[0.50; 0.52]	3.0%	0.3%
Andac, et al 2017	86	282	0.30	[0.25; 0.36]	0.1%	0.3%
Yang, et al 2008	1691	9617	0.18	[0.17; 0.18]	2.7%	0.3%
Yim, et al 2015	748	1774	0.42	[0.40; 0.45]	0.5%	0.3%
Yokota, et al 2016	1384	1961	0.71	[0.69; 0.73]	0.6%	0.3%
Gast, et al 2008	1983	5113	0.39	[0.37; 0.40]	1.5%	0.3%
Du, et al 2020	1057	3141	0.34	[0.32; 0.35]	0.9%	0.3%
Buhling, et al 2014	1347	1893	0.71	[0.69; 0.73]	0.5%	0.3%
Singh, et al 2014	117	252	0.46	[0.40; 0.53]	0.1%	0.3%
Kulkarni, et al 2016	65	100	0.65	[0.55; 0.74]	0.0%	0.3%
Rathnayake, et al 2019	162	350	0.46	[0.41; 0.52]	0.1%	0.3%
Kwon, et al 2016	885	1547	0.57	[0.55; 0.60]	0.4%	0.3%
Baquedano, et al 2023	560	1059	0.53	[0.50; 0.56]	0.3%	0.3%
Brzyski, et al 2001	84	160	0.52	[0.44; 0.60]	0.0%	0.3%
Ahmadieh, et al 2021	392	627	0.63	[0.59; 0.66]	0.2%	0.3%
Namgoung, et al 2022	880	4672	0.19	[0.18; 0.20]	1.3%	0.3%
Pateliya, et al 2022	63	100	0.63	[0.53; 0.72]	0.0%	0.3%
Srinivasan, et al 2021	46	100	0.46	[0.36; 0.56]	0.0%	0.3%
Chim, et al 2002	87	495	0.18	[0.14; 0.21]	0.1%	0.3%
Dhillon,et al 2006	173	326	0.53	[0.47; 0.59]	0.1%	0.3%
Chuni, et al 2011	508	729	0.70	[0.66; 0.73]	0.2%	0.3%
Nisar, et al 2015	2224	3062	0.73	[0.71; 0.74]	0.9%	0.3%
Zhang, et al 2016	33	106	0.31	[0.22; 0.41]	0.0%	0.3%
Agaba, et al 2017	70	107	0.65	[0.56; 0.74]	0.0%	0.3%
AlQuaiz, et al 2017	184	228	0.81	[0.75; 0.86]	0.1%	0.3%
Grigoriou, et al 2013	704	1025	0.69	[0.66; 0.72]	0.3%	0.3%
Hunter, et al 2012	5057	9324	0.54	[0.53; 0.55]	2.7%	0.3%
Haines, et al1 2005	179	248	0.72	[0.66; 0.78]	0.1%	0.3%
Haines, et al2 2005	145	195	0.74	[0.68; 0.80]	0.1%	0.3%
Haines, et al3 2005	3	59	0.05	[0.01; 0.14]	0.0%	0.3%
Haines, et al4 2005	46	97	0.47	[0.37; 0.58]	0.0%	0.3%
Haines, et al5 2005	11	24	0.46	[0.26; 0.67]	0.0%	0.3%
Haines, et al6 2005	22	58	0.38	[0.26; 0.52]	0.0%	0.3%
Haines, et al7 2005	37	81	0.46	[0.35; 0.57]	0.0%	0.3%
Haines, et al8 2005	92	150	0.61	[0.53; 0.69]	0.0%	0.3%
Haines, et al9 2005	100	100	1.00	[0.96; 1.00]	0.0%	0.3%
Melby, et al 2005	36	117	0.31	[0.23; 0.40]	0.0%	0.3%
Dennerstein, et al 2000	10	172	0.06	[0.03; 0.10]	0.0%	0.3%
Nappi, et al1 2008	183	300	0.61	[0.55; 0.67]	0.1%	0.3%
Nappi, et al2 2008	153	300	0.51	[0.45; 0.57]	0.1%	0.3%
Nappi, et al3 2008	132	300	0.44	[0.38; 0.50]	0.1%	0.3%
Nappi, et al4 2008	154	301	0.51	[0.45; 0.57]	0.1%	0.3%
Nappi, et al5 2008	169	301	0.56	[0.50; 0.62]	0.1%	0.3%
Nappi, et al6 2008	144	301	0.48	[0.42; 0.54]	0.1%	0.3%
Gotmar, et al 2008	3413	6908	0.49	[0.48; 0.51]	2.0%	0.3%
G..recka, et al 2022	226	349	0.65	[0.59; 0.70]	0.1%	0.3%
Saied, et al 2021	255	342	0.75	[0.70; 0.79]	0.1%	0.3%
Sheereen, et al 2022	70	100	0.70	[0.60; 0.79]	0.0%	0.3%
Kang, et al 2021	112	150	0.75	[0.67; 0.81]	0.0%	0.3%
Senthilvel, et al 2018	113	150	0.75	[0.68; 0.82]	0.0%	0.3%
Karmakar, et al 2017	60	100	0.60	[0.50; 0.70]	0.0%	0.3%
Ganapathy, et al 2018	94	140	0.67	[0.59; 0.75]	0.0%	0.3%
Sripraser, et al 2017	410	632	0.65	[0.61; 0.69]	0.2%	0.3%
Tan, et al 2014	215	305	0.70	[0.65; 0.76]	0.1%	0.3%
Chopra, et al 2022	345	504	0.68	[0.64; 0.72]	0.1%	0.3%
Vaccaro, et al 2021	390	1028	0.38	[0.35; 0.41]	0.3%	0.3%
Hachul, et al 2021	85	235	0.36	[0.30; 0.43]	0.1%	0.3%
Sa.., et al 2020	361	647	0.56	[0.52; 0.60]	0.2%	0.3%
Smith, et al 2015	347	761	0.46	[0.42; 0.49]	0.2%	0.3%
Zhao, et al 2023	953	1829	0.52	[0.50; 0.54]	0.5%	0.3%
Han, et al 2023	24	66	0.36	[0.25; 0.49]	0.0%	0.3%
Ryu, et al 2022	401	918	0.44	[0.40; 0.47]	0.3%	0.3%
Salin, et al 2022	332	2661	0.12	[0.11; 0.14]	0.8%	0.3%
Thakur, et al 2022	293	400	0.73	[0.69; 0.78]	0.1%	0.3%
Tijerina, et al 2022	120	406	0.30	[0.25; 0.34]	0.1%	0.3%
Ko?o?lu, et al 2022	278	383	0.73	[0.68; 0.77]	0.1%	0.3%
Arakane, et al 2011	207	340	0.61	[0.55; 0.66]	0.1%	0.3%
AlDughaither, et al 2015	56	119	0.47	[0.38; 0.56]	0.0%	0.3%
Ceylan, et al 2014	486	874	0.56	[0.52; 0.59]	0.3%	0.3%
Thakur,et al 2019	130	351	0.37	[0.32; 0.42]	0.1%	0.3%
Gallicchio, et al 2005	354	612	0.58	[0.54; 0.62]	0.2%	0.3%
Kim, et al 2017	560	743	0.75	[0.72; 0.78]	0.2%	0.3%
Sievert, et al 2022	116	162	0.72	[0.64; 0.78]	0.0%	0.3%
Gerber, et al 2018	49	139	0.35	[0.27; 0.44]	0.0%	0.3%
Silvestrin, et al 2016	68	110	0.62	[0.52; 0.71]	0.0%	0.3%
Ghazanfarpour, et al 2016	93	202	0.46	[0.39; 0.53]	0.1%	0.3%
Duffy, et al 2012	2006	4295	0.47	[0.45; 0.48]	1.2%	0.3%
Loutfy, et al 2006	408	450	0.91	[0.88; 0.93]	0.1%	0.3%
Delavar, et al 2011	689	1397	0.49	[0.47; 0.52]	0.4%	0.3%
Chedraui, et al 2007	170	325	0.52	[0.47; 0.58]	0.1%	0.3%
Chedraui, et al 2012	529	904	0.59	[0.55; 0.62]	0.3%	0.3%
Chedraui, et al 2014	350	451	0.78	[0.73; 0.81]	0.1%	0.3%
Suka, et al 2010	387	1169	0.33	[0.30; 0.36]	0.3%	0.3%
Rahman, et al 2011	182	509	0.36	[0.32; 0.40]	0.1%	0.3%
Singh, et al 2012	1329	1765	0.75	[0.73; 0.77]	0.5%	0.3%
Aguilar−Zavala, et al 2012	219	290	0.76	[0.70; 0.80]	0.1%	0.3%
Agwu, et al 2008	108	186	0.58	[0.51; 0.65]	0.1%	0.3%
Ahmed, et al 2016	96	150	0.64	[0.56; 0.72]	0.0%	0.3%
Gartoulla, et al 2016	553	1057	0.52	[0.49; 0.55]	0.3%	0.3%
Berecki−Gisolf, et al 2009	2424	8648	0.28	[0.27; 0.29]	2.5%	0.3%
Bener, et al 2014	431	1158	0.37	[0.34; 0.40]	0.3%	0.3%
Bener, et al 2000	129	390	0.33	[0.28; 0.38]	0.1%	0.3%
Im, et al 2010	277	512	0.54	[0.50; 0.58]	0.1%	0.3%
Williams, et al 2008	2099	3135	0.67	[0.65; 0.69]	0.9%	0.3%
Ande, et al 2011	272	533	0.51	[0.47; 0.55]	0.2%	0.3%
Anden?s, et al 2020	8333	12985	0.64	[0.63; 0.65]	3.7%	0.3%
Khatoon, et al 2018	54	121	0.45	[0.36; 0.54]	0.0%	0.3%
Budako?lu, et al 2007	204	338	0.60	[0.55; 0.66]	0.1%	0.3%
Sharanya Shre, et al 2016	36	150	0.24	[0.17; 0.32]	0.0%	0.3%
Damodaran, et al 2000	122	164	0.74	[0.67; 0.81]	0.0%	0.3%
Zeleke, et al 2016	303	1452	0.21	[0.19; 0.23]	0.4%	0.3%
Beura, et al 2020	66	104	0.63	[0.53; 0.73]	0.0%	0.3%
Thapa, et al 2020	118	177	0.67	[0.59; 0.74]	0.1%	0.3%
Ibrahim, et al 2015	880	1214	0.72	[0.70; 0.75]	0.3%	0.3%
Nusrat, et al 2008	513	863	0.59	[0.56; 0.63]	0.2%	0.3%
Bhavani, et al 2022	176	300	0.59	[0.53; 0.64]	0.1%	0.3%
Liu, et al 2007	95	267	0.36	[0.30; 0.42]	0.1%	0.3%
Fooladi, et al 2018	453	1309	0.35	[0.32; 0.37]	0.4%	0.3%
Lee, et al 2020	10332	20882	0.49	[0.49; 0.50]	6.0%	0.3%
Modoodi, et al 2020	289	350	0.83	[0.78; 0.86]	0.1%	0.3%
Kalhan, et al 2020	147	400	0.37	[0.32; 0.42]	0.1%	0.3%
Rindner, et al 2017	72	109	0.66	[0.56; 0.75]	0.0%	0.3%
Al−Musa, et al 2017	184	228	0.81	[0.75; 0.86]	0.1%	0.3%
Moilanen, et al 2010	93	1184	0.08	[0.06; 0.10]	0.3%	0.3%
Bairy, et al 2009	158	352	0.45	[0.40; 0.50]	0.1%	0.3%
Heinemann, et al1 2008	1940	4791	0.40	[0.39; 0.42]	1.4%	0.3%
Heinemann, et al2 2008	662	1500	0.44	[0.42; 0.47]	0.4%	0.3%
Heinemann, et al3 2008	1353	3006	0.45	[0.43; 0.47]	0.9%	0.3%
Heinemann, et al4 2008	505	1000	0.50	[0.47; 0.54]	0.3%	0.3%
Uncu, et al 2007	610	1007	0.61	[0.57; 0.64]	0.3%	0.3%
Lawton, et al 2008	1030	3616	0.28	[0.27; 0.30]	1.0%	0.3%
Liu, et al 2008	94	217	0.43	[0.37; 0.50]	0.1%	0.3%
Liu, et al 2007	136	188	0.72	[0.65; 0.79]	0.1%	0.3%
Resmi, et al 2020	118	400	0.29	[0.25; 0.34]	0.1%	0.3%
Ashrafi, et al 2009	1850	2462	0.75	[0.73; 0.77]	0.7%	0.3%
Ayranci, et al 2010	1497	1551	0.97	[0.95; 0.97]	0.4%	0.3%
Deveci, et al 2010	450	519	0.87	[0.83; 0.90]	0.1%	0.3%
Sagdeo, et al 2011	243	500	0.49	[0.44; 0.53]	0.1%	0.3%
O?urlu, et al 2011	95	132	0.72	[0.63; 0.79]	0.0%	0.3%
Anolue, et al 2012	216	349	0.62	[0.57; 0.67]	0.1%	0.3%
Mahajan,et al 2012	56	100	0.56	[0.46; 0.66]	0.0%	0.3%
Mustafa, et al 2012	357	500	0.71	[0.67; 0.75]	0.1%	0.3%
Nappi, et al 2012	2604	3520	0.74	[0.72; 0.75]	1.0%	0.3%
Sassoon, et al 2014	46	63	0.73	[0.60; 0.83]	0.0%	0.3%
Liu, et al 2013	520	1686	0.31	[0.29; 0.33]	0.5%	0.3%
Mohamed, et al 2014	48	90	0.53	[0.43; 0.64]	0.0%	0.3%
Hashemzadeh, et al 2019	168	302	0.56	[0.50; 0.61]	0.1%	0.3%
Hestiantoro, et al 2019	70	245	0.29	[0.23; 0.35]	0.1%	0.3%
Kong, et al 2019	66	120	0.55	[0.46; 0.64]	0.0%	0.3%
Sharifi, et al 2019	595	600	0.99	[0.98; 1.00]	0.2%	0.3%
Holm, et al 2000	98	214	0.46	[0.39; 0.53]	0.1%	0.3%
Pan, et al 2002	61	161	0.38	[0.30; 0.46]	0.0%	0.3%
Kasuga, et al 2004	796	1069	0.74	[0.72; 0.77]	0.3%	0.3%
Kaur, et al 2004	46	245	0.19	[0.14; 0.24]	0.1%	0.3%
Yahya, et al 2002	47	130	0.36	[0.28; 0.45]	0.0%	0.3%
Gupta, et al1 2006	81	103	0.79	[0.69; 0.86]	0.0%	0.3%
Gupta, et al2 2006	15	50	0.30	[0.18; 0.45]	0.0%	0.3%
Inayat, et al 2017	62	308	0.20	[0.16; 0.25]	0.1%	0.3%
Can..rio, et al 2012	184	370	0.50	[0.45; 0.55]	0.1%	0.3%
Rahman, et al 2020	234	390	0.60	[0.55; 0.65]	0.1%	0.3%
Abedzadeh−Kalahroudi, et al 2012	603	700	0.86	[0.83; 0.89]	0.2%	0.3%
Diyu, et al 2022	230	318	0.72	[0.67; 0.77]	0.1%	0.3%
Zhang, et al 2016	785	2308	0.34	[0.32; 0.36]	0.7%	0.3%
Ryu, et al 2020	1705	2481	0.69	[0.67; 0.71]	0.7%	0.3%
Ma, et al 2017	772	2336	0.33	[0.31; 0.35]	0.7%	0.3%
Fu, et al 2020	899	3199	0.28	[0.27; 0.30]	0.9%	0.3%
Liu, et al 2018	2243	3382	0.66	[0.65; 0.68]	1.0%	0.3%
Li, et al 2016	340	1062	0.32	[0.29; 0.35]	0.3%	0.3%
Joshi, et al 2015	220	1000	0.22	[0.19; 0.25]	0.3%	0.3%
Ghimire, et al 2015	313	833	0.38	[0.34; 0.41]	0.2%	0.3%
Joseph, et al 2014	90	110	0.82	[0.73; 0.89]	0.0%	0.3%
Dasgupta, et al 2015	647	1400	0.46	[0.44; 0.49]	0.4%	0.3%
Bindhu, et al 2014	131	320	0.41	[0.35; 0.47]	0.1%	0.3%
Srivastava, et al 2014	63	117	0.54	[0.44; 0.63]	0.0%	0.3%
ROKHADE, et al 2013	14	115	0.12	[0.07; 0.20]	0.0%	0.3%
Sun, et al 2014	597	2046	0.29	[0.27; 0.31]	0.6%	0.3%
Lerner−Geva, et al 2010	208	612	0.34	[0.30; 0.38]	0.2%	0.3%
Sweed, et al 2012	307	400	0.77	[0.72; 0.81]	0.1%	0.3%
Taher, et al 2012	64	86	0.74	[0.64; 0.83]	0.0%	0.3%
Ryan, et al 2009	51	138	0.37	[0.29; 0.46]	0.0%	0.3%
G K, et al 2013	270	500	0.54	[0.50; 0.58]	0.1%	0.3%
Bernis, et al 2007	479	1069	0.45	[0.42; 0.48]	0.3%	0.3%
Dibonaventura, et al 2013	1912	3801	0.50	[0.49; 0.52]	1.1%	0.3%
Freeman, et al 2009	114	170	0.67	[0.59; 0.74]	0.0%	0.3%
Chedraui, et al 2007	197	300	0.66	[0.60; 0.71]	0.1%	0.3%
Nayak, et al 2019	247	290	0.85	[0.81; 0.89]	0.1%	0.3%
Alam, et al 2020	66	326	0.20	[0.16; 0.25]	0.1%	0.3%
Chung, et al 2006	40	305	0.13	[0.10; 0.17]	0.1%	0.3%
Hsu, et al 2005	35	197	0.18	[0.13; 0.24]	0.1%	0.3%
Ward, et al 2010	564	1115	0.51	[0.48; 0.54]	0.3%	0.3%
Pandey, et al 2020	93	189	0.49	[0.42; 0.57]	0.1%	0.3%
Abdelaziz, et al 2022	219	410	0.53	[0.48; 0.58]	0.1%	0.3%
Sievert, et al 2007	295	869	0.34	[0.31; 0.37]	0.2%	0.3%
Sievert, et al 2003	375	755	0.50	[0.46; 0.53]	0.2%	0.3%
Obermeyer, et al 2004	167	293	0.57	[0.51; 0.63]	0.1%	0.3%
Keenan, et al 2003	1637	2602	0.63	[0.61; 0.65]	0.7%	0.3%
Zhao, et al 2000	283	806	0.35	[0.32; 0.39]	0.2%	0.3%
Lam, et al 2003	228	978	0.23	[0.21; 0.26]	0.3%	0.3%
Gollschewski, et al 2004	388	868	0.45	[0.41; 0.48]	0.2%	0.3%
Biri, et al 2005	389	1049	0.37	[0.34; 0.40]	0.3%	0.3%
Obermeyer, et al 2002	182	299	0.61	[0.55; 0.66]	0.1%	0.3%
Loh, et al 2005	105	656	0.16	[0.13; 0.19]	0.2%	0.3%
Chaopotong, et al 2005	92	148	0.62	[0.54; 0.70]	0.0%	0.3%
Huang, et al1 2010	170	300	0.57	[0.51; 0.62]	0.1%	0.3%
Huang, et al2 2010	58	100	0.58	[0.48; 0.68]	0.0%	0.3%
Huang, et al3 2010	89	300	0.30	[0.25; 0.35]	0.1%	0.3%
Huang, et al4 2010	74	150	0.49	[0.41; 0.58]	0.0%	0.3%
Huang, et al5 2010	105	150	0.70	[0.62; 0.77]	0.0%	0.3%
Hunter, et al1 2012	88	171	0.51	[0.44; 0.59]	0.0%	0.3%
Hunter, et al2 2012	93	129	0.72	[0.64; 0.80]	0.0%	0.3%
Hunter, et al3 2012	120	198	0.61	[0.53; 0.67]	0.1%	0.3%
Hunter, et al4 2012	223	398	0.56	[0.51; 0.61]	0.1%	0.3%
Anderson, et al1 2004	388	868	0.45	[0.41; 0.48]	0.2%	0.3%
Anderson, et al2 2004	386	829	0.47	[0.43; 0.50]	0.2%	0.3%
Glod, et al 2006	1327	3302	0.40	[0.39; 0.42]	0.9%	0.3%
Fagulha, et al 2011	251	728	0.34	[0.31; 0.38]	0.2%	0.3%
Zang, et al 2016	168	743	0.23	[0.20; 0.26]	0.2%	0.3%
Masjoudl, et al 2017	384	645	0.60	[0.56; 0.63]	0.2%	0.3%
Durairaj, et al 2022	38	74	0.51	[0.39; 0.63]	0.0%	0.3%
Bala, et al 2022	152	245	0.62	[0.56; 0.68]	0.1%	0.3%
Stefanopoulou, et al 2014	247	717	0.34	[0.31; 0.38]	0.2%	0.3%
Abou−Raya, et al 2016	371	540	0.69	[0.65; 0.73]	0.2%	0.3%
Ogwumike, et al 2012	166	547	0.30	[0.27; 0.34]	0.2%	0.3%
Sierra, et al 2004	316	385	0.82	[0.78; 0.86]	0.1%	0.3%
Castelo−Branco, et al 2005	58	125	0.46	[0.37; 0.56]	0.0%	0.3%
Jin, et al 2014	614	1215	0.51	[0.48; 0.53]	0.3%	0.3%
Pe_rez−L.pez, et al 2013	1077	1739	0.62	[0.60; 0.64]	0.5%	0.3%
Huang, et al 2020	1134	1501	0.76	[0.73; 0.78]	0.4%	0.3%
Dasgupta, et al 2012	196	480	0.41	[0.36; 0.45]	0.1%	0.3%
Huseth−Zosel, et al 2014	188	296	0.64	[0.58; 0.69]	0.1%	0.3%
Zhang, et al 2020	2244	4595	0.49	[0.47; 0.50]	1.3%	0.3%
Wu, et al 2023	298	468	0.64	[0.59; 0.68]	0.1%	0.3%
Abdullah, et al 2016	142	258	0.55	[0.49; 0.61]	0.1%	0.3%
Chu, et al 2022	1164	1748	0.67	[0.64; 0.69]	0.5%	0.3%
Hickey, et al 2017	456	1151	0.40	[0.37; 0.43]	0.3%	0.3%
Blell, et al 2014	146	254	0.57	[0.51; 0.64]	0.1%	0.3%
Dienye, et al 2013	297	385	0.77	[0.73; 0.81]	0.1%	0.3%
Whiteley, et al 2013	3767	8811	0.43	[0.42; 0.44]	2.5%	0.3%
Olowokere, et al 2020	197	271	0.73	[0.67; 0.78]	0.1%	0.3%
Zhang, et al 2009	416	1399	0.30	[0.27; 0.32]	0.4%	0.3%
Salazar−Pousada, et al1 2017	202	215	0.94	[0.90; 0.97]	0.1%	0.3%
Salazar−Pousada, et al2 2017	109	202	0.54	[0.47; 0.61]	0.1%	0.3%
Salazar−Pousada, et al3 2017	227	231	0.98	[0.96; 1.00]	0.1%	0.3%
Salazar−Pousada, et al4 2017	117	216	0.54	[0.47; 0.61]	0.1%	0.3%
Whiteman, et al 2003	609	1087	0.56	[0.53; 0.59]	0.3%	0.3%
Ruan, et al 2017	533	1225	0.44	[0.41; 0.46]	0.4%	0.3%
Devi, et al 2005	61	151	0.40	[0.32; 0.49]	0.0%	0.3%
Xu, et al 2005	102	237	0.43	[0.37; 0.50]	0.1%	0.3%
Shakhatreh, et al 2006	87	143	0.61	[0.52; 0.69]	0.0%	0.3%
Obermeyer, et al 2007	130	271	0.48	[0.42; 0.54]	0.1%	0.3%
Al−Qutob, et al 2000	6	137	0.04	[0.02; 0.09]	0.0%	0.3%
Chae, et al 2014	346	570	0.61	[0.57; 0.65]	0.2%	0.3%
Veigh, et al 2005	121	160	0.76	[0.68; 0.82]	0.0%	0.3%
Lampio, et al 2014	42	158	0.27	[0.20; 0.34]	0.0%	0.3%
Oppermann, et al 2012	94	211	0.45	[0.38; 0.52]	0.1%	0.3%
An, et al 2023	3755	7309	0.51	[0.50; 0.53]	2.1%	0.3%
Kamal, et al 2017	541	1100	0.49	[0.46; 0.52]	0.3%	0.3%
Rajbhandari, et al 2017	587	2000	0.29	[0.27; 0.31]	0.6%	0.3%
Cortes, et al 2023	124	358	0.35	[0.30; 0.40]	0.1%	0.3%
Baig, et al 2006	54	925	0.06	[0.04; 0.08]	0.3%	0.3%
Chalise, et al 2022	26	180	0.14	[0.10; 0.20]	0.1%	0.3%
**Common effect model**		**349608**	**0.47**	**[0.47; 0.47]**	**100.0%**	**--**
**Random effects model**			**0.53**	**[0.50; 0.55]**	**--**	**100.0%**

Heterogeneity: $I^2 = 99\%$, $\tau^2 = 0.0448$, $p = 0$

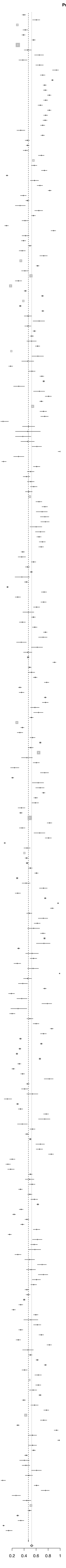

Supplementary Figure 2. Forest plot of prevalence of sleep problems among middle-aged women

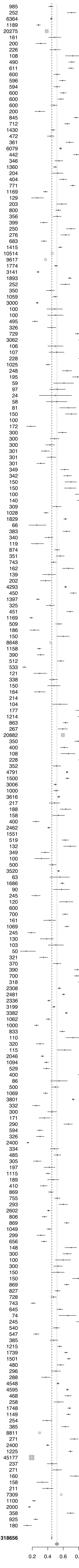

Study	Events	Total	Proportion	95%–CI	Weight (common)	Weight (random)
Lan, et al 2017	433	985	0.44	[0.41; 0.47]	0.3%	0.4%
Adhikari, et al 2022	179	252	0.71	[0.65; 0.77]	0.1%	0.4%
Wang, et al 2021	2846	6364	0.45	[0.43; 0.46]	2.0%	0.4%
Olaolorun, et al 2009	334	1189	0.28	[0.26; 0.31]	0.4%	0.4%
Li, et al 2012	7844	20275	0.39	[0.38; 0.39]	6.4%	0.4%
Javadivala, et al 2020	79	161	0.49	[0.41; 0.57]	0.1%	0.4%
Nagaraj, et al 2021	62	200	0.31	[0.25; 0.38]	0.1%	0.4%
Yisma, et al 2017	112	226	0.50	[0.43; 0.56]	0.1%	0.4%
Korkmaz, et al 2022	78	108	0.72	[0.63; 0.80]	0.0%	0.4%
AlQuaiz, et al 2013	349	490	0.71	[0.67; 0.75]	0.2%	0.4%
Genazzani, et al1 2006	428	611	0.70	[0.66; 0.74]	0.2%	0.4%
Genazzani, et al2 2006	312	600	0.52	[0.48; 0.56]	0.2%	0.4%
Genazzani, et al3 2006	352	596	0.59	[0.55; 0.63]	0.2%	0.4%
Genazzani, et al4 2006	309	594	0.52	[0.48; 0.56]	0.2%	0.4%
Genazzani, et al5 2006	360	600	0.60	[0.56; 0.64]	0.2%	0.4%
Genazzani, et al6 2006	342	600	0.57	[0.53; 0.61]	0.2%	0.4%
Genazzani, et al7 2006	288	600	0.48	[0.44; 0.52]	0.2%	0.4%
Aaron, et al 2002	59	200	0.29	[0.23; 0.36]	0.1%	0.4%
Abdel–Salam, et al 2021	649	845	0.77	[0.74; 0.80]	0.3%	0.4%
Anderson, et al1 2004	464	712	0.65	[0.62; 0.69]	0.2%	0.4%
Anderson, et al2 2004	671	1430	0.47	[0.44; 0.50]	0.4%	0.4%
Shafie, et al 2011	207	472	0.44	[0.39; 0.48]	0.1%	0.4%
Aloufi, et al 2022	247	361	0.68	[0.63; 0.73]	0.1%	0.4%
Bl..mel, et al 2012	3440	6079	0.57	[0.55; 0.58]	1.9%	0.4%
Chou, et al 2014	341	442	0.77	[0.73; 0.81]	0.1%	0.4%
Fallahzadeh, et al 2007	188	346	0.54	[0.49; 0.60]	0.1%	0.4%
Fuh, et al 2003	674	1360	0.50	[0.47; 0.52]	0.4%	0.4%
Chedraui, et al 2014	108	204	0.53	[0.46; 0.60]	0.1%	0.4%
Chedraui, et al 2010	242	404	0.60	[0.55; 0.65]	0.1%	0.4%
Ojeda, et al 2014	558	771	0.72	[0.69; 0.76]	0.2%	0.4%
Ishizuka, et al 2008	432	1169	0.37	[0.34; 0.40]	0.4%	0.4%
Kapur, et al 2009	34	129	0.26	[0.19; 0.35]	0.0%	0.4%
Sharma, et al 2021	118	203	0.58	[0.51; 0.65]	0.1%	0.4%
Qazi, et al 2006	430	800	0.54	[0.50; 0.57]	0.3%	0.4%
Rahman, et al 2010	186	356	0.52	[0.47; 0.58]	0.1%	0.4%
Shea, et al 2006	139	399	0.35	[0.30; 0.40]	0.1%	0.4%
Som, et al 2012	175	250	0.70	[0.64; 0.76]	0.1%	0.4%
Alwi, et al 2009	174	276	0.63	[0.57; 0.69]	0.1%	0.4%
Waidyasekera, et al 2009	239	683	0.35	[0.31; 0.39]	0.2%	0.4%
Silva, et al 2013	794	1415	0.56	[0.53; 0.59]	0.4%	0.4%
Mart..nez, et al 2013	4801	10514	0.46	[0.45; 0.47]	3.3%	0.4%
Yang, et al 2008	3576	9617	0.37	[0.36; 0.38]	3.0%	0.4%
Yim, et al 2015	897	1774	0.51	[0.48; 0.53]	0.6%	0.4%
Du, et al 2020	863	3141	0.27	[0.26; 0.29]	1.0%	0.4%
Buhling, et al 2014	1297	1893	0.69	[0.66; 0.71]	0.6%	0.4%
Singh, et al 2014	158	252	0.63	[0.56; 0.69]	0.1%	0.4%
Rathnayake, et al 2019	152	350	0.43	[0.38; 0.49]	0.1%	0.4%
Baquedano, et al 2023	524	1059	0.49	[0.46; 0.53]	0.3%	0.4%
Hwang, et al 2021	794	3000	0.26	[0.25; 0.28]	0.9%	0.4%
Pateliya, et al 2022	49	100	0.49	[0.39; 0.59]	0.0%	0.4%
Srinivasan, et al 2021	50	100	0.50	[0.40; 0.60]	0.0%	0.4%
Chim, et al 2002	116	495	0.23	[0.20; 0.27]	0.2%	0.4%
Dhillon,et al 2006	147	326	0.45	[0.40; 0.51]	0.1%	0.4%
Chuni, et al 2011	574	729	0.79	[0.76; 0.82]	0.2%	0.4%
Nisar, et al 2015	2489	3062	0.81	[0.80; 0.83]	1.0%	0.4%
Zhang, et al 2016	53	106	0.50	[0.40; 0.60]	0.0%	0.4%
Agaba, et al 2017	55	107	0.51	[0.42; 0.61]	0.0%	0.4%
AlQuaiz, et al 2017	194	228	0.85	[0.80; 0.89]	0.1%	0.4%
Grigoriou, et al 2013	413	1025	0.40	[0.37; 0.43]	0.3%	0.4%
Haines, et al1 2005	161	248	0.65	[0.59; 0.71]	0.1%	0.4%
Haines, et al2 2005	153	195	0.78	[0.72; 0.84]	0.1%	0.4%
Haines, et al3 2005	36	59	0.61	[0.47; 0.73]	0.0%	0.4%
Haines, et al4 2005	51	97	0.53	[0.42; 0.63]	0.0%	0.4%
Haines, et al5 2005	12	24	0.50	[0.29; 0.71]	0.0%	0.3%
Haines, et al6 2005	30	58	0.52	[0.38; 0.65]	0.0%	0.4%
Haines, et al7 2005	54	81	0.67	[0.55; 0.77]	0.0%	0.4%
Haines, et al8 2005	106	150	0.71	[0.63; 0.78]	0.0%	0.4%
Haines, et al9 2005	92	100	0.92	[0.85; 0.96]	0.0%	0.4%
Dennerstein, et al 2000	31	172	0.18	[0.13; 0.25]	0.1%	0.4%
Nappi, et al1 2008	177	300	0.59	[0.53; 0.65]	0.1%	0.4%
Nappi, et al2 2008	147	300	0.49	[0.43; 0.55]	0.1%	0.4%
Nappi, et al3 2008	129	300	0.43	[0.37; 0.49]	0.1%	0.4%
Nappi, et al4 2008	111	301	0.37	[0.31; 0.43]	0.1%	0.4%
Nappi, et al5 2008	135	301	0.45	[0.39; 0.51]	0.1%	0.4%
Nappi, et al6 2008	93	301	0.31	[0.26; 0.36]	0.1%	0.4%
G..recka, et al 2022	216	349	0.62	[0.57; 0.67]	0.1%	0.4%
Saied, et al 2021	225	342	0.66	[0.60; 0.71]	0.1%	0.4%
Kang, et al 2021	105	150	0.70	[0.62; 0.77]	0.0%	0.4%
Senthilvel, et al 2018	104	150	0.69	[0.61; 0.77]	0.0%	0.4%
Karmakar, et al 2017	84	100	0.84	[0.75; 0.91]	0.0%	0.4%
Ganapathy, et al 2018	119	140	0.85	[0.78; 0.90]	0.0%	0.4%
Tan, et al 2014	160	309	0.52	[0.46; 0.57]	0.1%	0.4%
Vaccaro, et al 2021	388	1028	0.38	[0.35; 0.41]	0.3%	0.4%
Zhao, et al 2023	1149	1829	0.63	[0.61; 0.65]	0.6%	0.4%
Han, et al 2023	17	66	0.26	[0.16; 0.38]	0.0%	0.4%
Ko?o?lu, et al 2022	247	383	0.64	[0.59; 0.69]	0.1%	0.4%
Arakane, et al 2011	141	340	0.41	[0.36; 0.47]	0.1%	0.4%
AlDughaither, et al 2015	31	119	0.26	[0.18; 0.35]	0.0%	0.4%
Ceylan, et al 2014	457	874	0.52	[0.49; 0.56]	0.3%	0.4%
Thakur,et al 2019	197	351	0.56	[0.51; 0.61]	0.1%	0.4%
Kim, et al 2017	354	743	0.48	[0.44; 0.51]	0.2%	0.4%
Sievert, et al 2022	108	162	0.67	[0.59; 0.74]	0.1%	0.4%
Gerber, et al 2018	58	139	0.42	[0.33; 0.50]	0.0%	0.4%
Ghazanfarpour, et al 2016	75	202	0.37	[0.30; 0.44]	0.1%	0.4%
Duffy, et al 2012	2708	4293	0.63	[0.62; 0.65]	1.3%	0.4%
Loutfy, et al 2006	329	450	0.73	[0.69; 0.77]	0.1%	0.4%
Delavar, et al 2011	381	1397	0.27	[0.25; 0.30]	0.4%	0.4%
Chedraui, et al 2007	141	325	0.43	[0.38; 0.49]	0.1%	0.4%
Chedraui, et al 2014	310	451	0.69	[0.64; 0.73]	0.1%	0.4%
Suka, et al 2010	218	1169	0.19	[0.16; 0.21]	0.4%	0.4%
Rahman, et al 2011	277	509	0.54	[0.50; 0.59]	0.2%	0.4%
Agwu, et al 2008	42	186	0.23	[0.17; 0.29]	0.1%	0.4%
Ahmed, et al 2016	90	150	0.60	[0.52; 0.68]	0.0%	0.4%
Berecki–Gisolf, et al 2009	3806	8648	0.44	[0.43; 0.45]	2.7%	0.4%
Bener, et al 2014	335	1158	0.29	[0.26; 0.32]	0.4%	0.4%
Bener, et al 2000	120	390	0.31	[0.26; 0.36]	0.1%	0.4%
Im, et al 2010	291	512	0.57	[0.52; 0.61]	0.2%	0.4%
Ande, et al 2011	51	533	0.10	[0.07; 0.12]	0.2%	0.4%
Khatoon, et al 2018	36	121	0.30	[0.22; 0.39]	0.0%	0.4%
Budako?lu, et al 2007	160	338	0.47	[0.42; 0.53]	0.1%	0.4%
Sharanya Shre, et al 2016	72	150	0.48	[0.40; 0.56]	0.0%	0.4%
Damodaran, et al 2000	48	164	0.29	[0.22; 0.37]	0.1%	0.4%
Zeleke, et al 2021	192	214	0.90	[0.85; 0.93]	0.1%	0.4%
Beura, et al 2020	58	104	0.56	[0.46; 0.66]	0.0%	0.4%
Thapa, et al 2020	146	177	0.82	[0.76; 0.88]	0.1%	0.4%
Ibrahim, et al 2015	985	1214	0.81	[0.79; 0.83]	0.4%	0.4%
Nusrat, et al 2008	544	863	0.63	[0.60; 0.66]	0.3%	0.4%
Liu, et al 2007	159	267	0.60	[0.53; 0.65]	0.1%	0.4%
Lee, et al 2020	12440	20882	0.60	[0.59; 0.60]	6.6%	0.4%
Modoodi, et al 2020	277	350	0.79	[0.75; 0.83]	0.1%	0.4%
Kalhan, et al 2020	245	400	0.61	[0.56; 0.66]	0.1%	0.4%
Rindner, et al 2017	72	108	0.67	[0.57; 0.75]	0.0%	0.4%
Al–Musa, et al 2017	194	228	0.85	[0.80; 0.89]	0.1%	0.4%
Bairy, et al 2009	182	352	0.52	[0.46; 0.57]	0.1%	0.4%
Heinemann, et al1 2008	3112	4791	0.65	[0.64; 0.66]	1.5%	0.4%
Heinemann, et al2 2008	981	1500	0.65	[0.63; 0.68]	0.5%	0.4%
Heinemann, et al3 2008	1497	3006	0.50	[0.48; 0.52]	0.9%	0.4%
Heinemann, et al4 2008	508	1000	0.51	[0.48; 0.54]	0.3%	0.4%
Lawton, et al 2008	1962	3616	0.54	[0.53; 0.56]	1.1%	0.4%
Liu, et al 2008	106	217	0.49	[0.42; 0.56]	0.1%	0.4%
Liu, et al 2007	119	188	0.63	[0.56; 0.70]	0.1%	0.4%
Im, et al 2009	85	158	0.54	[0.46; 0.62]	0.0%	0.4%
Resmi, et al 2020	99	400	0.25	[0.21; 0.29]	0.1%	0.4%
Ashrafi, et al 2009	1022	2462	0.42	[0.40; 0.43]	0.8%	0.4%
Ayranci, et al 2010	1308	1551	0.84	[0.82; 0.86]	0.5%	0.4%
Deveci, et al 2010	325	519	0.63	[0.58; 0.67]	0.2%	0.4%
O?urlu, et al 2011	91	132	0.69	[0.60; 0.77]	0.0%	0.4%
Anolue, et al 2012	128	349	0.37	[0.32; 0.42]	0.1%	0.4%
Mahajan,et al 2012	32	100	0.32	[0.23; 0.42]	0.0%	0.4%
Mustafa, et al 2012	232	500	0.46	[0.42; 0.51]	0.2%	0.4%
Nappi, et al 2012	1901	3520	0.54	[0.52; 0.56]	1.1%	0.4%
Sassoon, et al 2014	35	63	0.56	[0.42; 0.68]	0.0%	0.4%
Liu, et al 2013	993	1686	0.59	[0.57; 0.61]	0.5%	0.4%
Mohamed, et al 2014	52	90	0.58	[0.47; 0.68]	0.0%	0.4%
Hestiantoro, et al 2019	60	245	0.24	[0.19; 0.30]	0.1%	0.4%
Kong, et al 2019	77	120	0.64	[0.55; 0.73]	0.0%	0.4%
Sharifi, et al 2019	592	600	0.99	[0.97; 0.99]	0.2%	0.4%
Taavoni, et al 2015	438	700	0.63	[0.59; 0.66]	0.2%	0.4%
Pan, et al 2002	68	161	0.42	[0.35; 0.50]	0.1%	0.4%
Kasuga, et al 2004	693	1069	0.65	[0.62; 0.68]	0.3%	0.4%
Kaur, et al 2004	46	245	0.19	[0.14; 0.24]	0.1%	0.4%
Yahya, et al 2002	50	130	0.38	[0.30; 0.47]	0.0%	0.4%
Gupta, et al1 2006	52	103	0.50	[0.40; 0.60]	0.0%	0.4%
Gupta, et al2 2006	6	50	0.12	[0.05; 0.24]	0.0%	0.4%
Mathialagan, et al 2022	111	321	0.35	[0.29; 0.40]	0.1%	0.4%
Can..rio, et al 2012	193	370	0.52	[0.47; 0.57]	0.1%	0.4%
Rahman, et al 2020	307	390	0.79	[0.74; 0.83]	0.1%	0.4%
Abedzadeh–Kalahroudi, et al 2012	543	700	0.78	[0.74; 0.81]	0.2%	0.4%
Diyu, et al 2022	267	318	0.84	[0.79; 0.88]	0.1%	0.4%
Zhang, et al 2016	1270	2308	0.55	[0.53; 0.57]	0.7%	0.4%
Ryu, et al 2020	1501	2481	0.60	[0.59; 0.62]	0.8%	0.4%
Ma, et al 2017	971	2336	0.42	[0.40; 0.44]	0.7%	0.4%
Fu, et al 2020	1535	3199	0.48	[0.46; 0.50]	1.0%	0.4%
Liu, et al 2018	2165	3382	0.64	[0.62; 0.66]	1.1%	0.4%
Li, et al 2016	432	1062	0.41	[0.38; 0.44]	0.3%	0.4%
Joshi, et al 2015	254	1000	0.25	[0.23; 0.28]	0.3%	0.4%
Ghimire, et al 2015	490	833	0.59	[0.55; 0.62]	0.3%	0.4%
Joseph, et al 2014	80	110	0.73	[0.63; 0.81]	0.0%	0.4%
Bindhu, et al 2014	96	320	0.30	[0.25; 0.35]	0.1%	0.4%
ROKHADE, et al 2013	71	115	0.62	[0.52; 0.71]	0.0%	0.4%
Sun, et al 2014	679	2046	0.33	[0.31; 0.35]	0.6%	0.4%
Wong, et al 2022	418	1094	0.38	[0.35; 0.41]	0.3%	0.4%
Xu, et al 2016	203	529	0.38	[0.34; 0.43]	0.2%	0.4%
Sweed, et al 2012	333	400	0.83	[0.79; 0.87]	0.1%	0.4%
Taher, et al 2012	47	86	0.55	[0.44; 0.65]	0.0%	0.4%
G K, et al 2013	250	500	0.50	[0.46; 0.54]	0.2%	0.4%
Bernis, et al 2007	398	1069	0.37	[0.34; 0.40]	0.3%	0.4%
Dibonaventura, et al 2013	2771	3801	0.73	[0.71; 0.74]	1.2%	0.4%
Seib, et al 2014	83	332	0.25	[0.20; 0.30]	0.1%	0.4%
Chedraui, et al 2007	137	300	0.46	[0.40; 0.51]	0.1%	0.4%
Dutta, et al 2018	61	171	0.36	[0.29; 0.43]	0.1%	0.4%
Nayak, et al 2019	193	290	0.67	[0.61; 0.72]	0.1%	0.4%
Ps, et al 2017	177	594	0.30	[0.26; 0.34]	0.2%	0.4%
Alam, et al 2020	98	326	0.30	[0.25; 0.35]	0.1%	0.4%
Shin, et al 2005	343	2400	0.14	[0.13; 0.16]	0.8%	0.4%
Fabbrini, et al 2015	163	334	0.49	[0.43; 0.54]	0.1%	0.4%
Luo, et al 2020	255	485	0.53	[0.48; 0.57]	0.2%	0.4%
Chung, et al 2006	80	305	0.26	[0.21; 0.32]	0.1%	0.4%
Hsu, et al 2005	83	197	0.42	[0.35; 0.49]	0.1%	0.4%
Ward, et al 2010	450	1115	0.40	[0.37; 0.43]	0.3%	0.4%
Pandey, et al 2020	89	189	0.47	[0.40; 0.54]	0.1%	0.4%
Abdelaziz, et al 2022	142	410	0.35	[0.30; 0.39]	0.1%	0.4%
Sievert, et al 2007	382	869	0.44	[0.41; 0.47]	0.3%	0.4%
Sievert, et al 2003	395	755	0.52	[0.49; 0.56]	0.2%	0.4%
Obermeyer, et al 2004	176	293	0.60	[0.54; 0.66]	0.1%	0.4%
Keenan, et al 2003	1069	2602	0.41	[0.39; 0.43]	0.8%	0.4%
Zhao, et al 2000	287	806	0.36	[0.32; 0.39]	0.3%	0.4%
Gollschewski, et al 2004	561	869	0.65	[0.61; 0.68]	0.3%	0.4%
Biri, et al 2005	438	1049	0.42	[0.39; 0.45]	0.3%	0.4%
Obermeyer, et al 2002	93	299	0.31	[0.26; 0.37]	0.1%	0.4%
Loh, et al 2005	230	656	0.35	[0.31; 0.39]	0.2%	0.4%
Chaopotong, et al 2005	104	148	0.70	[0.62; 0.77]	0.0%	0.4%
Huang, et al1 2010	196	300	0.65	[0.60; 0.71]	0.1%	0.4%
Huang, et al2 2010	60	100	0.60	[0.50; 0.70]	0.0%	0.4%
Huang, et al3 2010	160	300	0.53	[0.48; 0.59]	0.1%	0.4%
Huang, et al4 2010	80	150	0.53	[0.45; 0.62]	0.0%	0.4%
Huang, et al5 2010	119	150	0.79	[0.72; 0.86]	0.0%	0.4%
Anderson, et al1 2004	561	869	0.65	[0.61; 0.68]	0.3%	0.4%
Anderson, et al2 2004	436	827	0.53	[0.49; 0.56]	0.3%	0.4%
Fagulha, et al 2011	345	728	0.47	[0.44; 0.51]	0.2%	0.4%
Zang, et al 2016	157	743	0.21	[0.18; 0.24]	0.2%	0.4%
Masjoudl, et al 2017	359	645	0.56	[0.52; 0.60]	0.2%	0.4%
Durairaj, et al 2022	49	74	0.66	[0.54; 0.77]	0.0%	0.4%
Bala, et al 2022	202	245	0.82	[0.77; 0.87]	0.1%	0.4%
Abou–Raya, et al 2016	275	540	0.51	[0.47; 0.55]	0.2%	0.4%
Ogwumike, et al 2012	137	547	0.25	[0.21; 0.29]	0.2%	0.4%
Sierra, et al 2004	184	385	0.48	[0.43; 0.53]	0.1%	0.4%
Jin, et al 2014	675	1215	0.56	[0.53; 0.58]	0.4%	0.4%
Pe_rez–L.pez, et al 2013	1107	1739	0.64	[0.61; 0.66]	0.5%	0.4%
Huang, et al 2020	1010	1501	0.67	[0.65; 0.70]	0.5%	0.4%
Dasgupta, et al 2012	272	480	0.57	[0.52; 0.61]	0.2%	0.4%
Huseth–Zosel, et al 2014	150	296	0.51	[0.45; 0.57]	0.1%	0.4%
Ornat, et al 2014	108	288	0.38	[0.32; 0.43]	0.1%	0.4%
Legorreta, et al 2013	2424	4548	0.53	[0.52; 0.55]	1.4%	0.4%
Zhang, et al 2020	3006	4595	0.65	[0.64; 0.67]	1.4%	0.4%
Wu, et al 2023	311	468	0.66	[0.62; 0.71]	0.1%	0.4%
Abdullah, et al 2016	140	258	0.54	[0.48; 0.60]	0.1%	0.4%
Chu, et al 2022	1189	1748	0.68	[0.66; 0.70]	0.5%	0.4%
Hickey, et al 2017	768	1149	0.67	[0.64; 0.70]	0.4%	0.4%
Blell, et al 2014	99	254	0.39	[0.33; 0.45]	0.1%	0.4%
Dienye, et al 2013	241	385	0.63	[0.58; 0.67]	0.1%	0.4%
Whiteley, et al 2013	2591	8811	0.29	[0.28; 0.30]	2.8%	0.4%
Olowokere, et al 2020	201	271	0.74	[0.69; 0.79]	0.1%	0.4%
Zhu, et al 2021	960	2400	0.40	[0.38; 0.42]	0.8%	0.4%
Ruan, et al 2017	850	1225	0.69	[0.67; 0.72]	0.4%	0.4%
An, et al 2022	8674	45177	0.19	[0.19; 0.20]	14.2%	0.4%
Xu, et al 2005	108	237	0.46	[0.39; 0.52]	0.1%	0.4%
Obermeyer, et al 2007	141	271	0.52	[0.46; 0.58]	0.1%	0.4%
Veigh, et al 2005	122	160	0.76	[0.69; 0.83]	0.1%	0.4%
Lampio, et al 2014	52	158	0.33	[0.26; 0.41]	0.0%	0.4%
Oppermann, et al 2012	84	211	0.40	[0.33; 0.47]	0.1%	0.4%
An, et al 2023	4200	7309	0.57	[0.56; 0.59]	2.3%	0.4%
Kamal, et al 2017	243	1100	0.22	[0.20; 0.25]	0.3%	0.4%
Rajbhandari, et al 2017	328	2000	0.16	[0.15; 0.18]	0.6%	0.4%
Cortes, et al 2023	249	358	0.70	[0.64; 0.74]	0.1%	0.4%
Baig, et al 2006	227	925	0.25	[0.22; 0.27]	0.3%	0.4%
Chalise, et al 2022	24	180	0.13	[0.09; 0.19]	0.1%	0.4%
**Common effect model**		**318656**	**0.45**	**[0.45; 0.45]**	**100.0%**	**--**
**Random effects model**			**0.52**	**[0.50; 0.54]**	**--**	**100.0%**

Heterogeneity: $I^2 = 99\%$, $\tau^2 = 0.0340$, $p = 0$

Supplementary Figure 3. Forest plot of prevalence of heart discomfort among middle-aged women

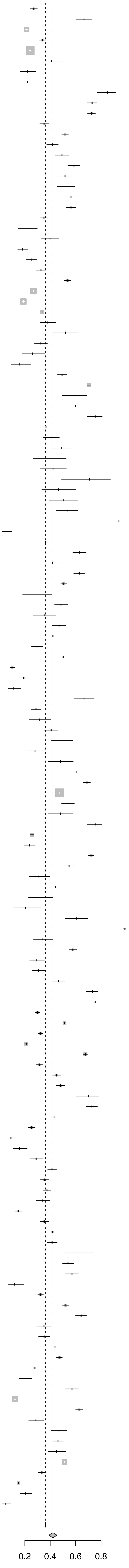

Study	Events	Total	Proportion	95%−CI	Weight (common)	Weight (random)
Lan, et al 2017	266	985	0.27	[0.24; 0.30]	0.6%	0.7%
Adhikari, et al 2022	168	252	0.67	[0.60; 0.72]	0.1%	0.7%
Wang, et al 2021	1373	6364	0.22	[0.21; 0.23]	3.7%	0.7%
Olaolorun, et al 2009	402	1189	0.34	[0.31; 0.37]	0.7%	0.7%
Li, et al 2012	4907	20275	0.24	[0.24; 0.25]	11.9%	0.7%
Javadivala, et al 2020	66	161	0.41	[0.33; 0.49]	0.1%	0.7%
Nagaraj, et al 2021	44	200	0.22	[0.16; 0.28]	0.1%	0.7%
Yisma, et al 2017	50	226	0.22	[0.17; 0.28]	0.1%	0.7%
Korkmaz, et al 2022	92	108	0.85	[0.77; 0.91]	0.1%	0.7%
AlQuaiz, et al 2013	358	490	0.73	[0.69; 0.77]	0.3%	0.7%
Abdel−Salam, et al 2021	613	845	0.73	[0.69; 0.76]	0.5%	0.7%
Anderson, et al1 2004	251	712	0.35	[0.32; 0.39]	0.4%	0.7%
Anderson, et al2 2004	739	1430	0.52	[0.49; 0.54]	0.8%	0.7%
Shafie, et al 2011	197	472	0.42	[0.37; 0.46]	0.3%	0.7%
Aloufi, et al 2022	178	361	0.49	[0.44; 0.55]	0.2%	0.7%
Chou, et al 2014	259	442	0.59	[0.54; 0.63]	0.3%	0.7%
Fallahzadeh, et al 2007	179	346	0.52	[0.46; 0.57]	0.2%	0.7%
Chedraui, et al 2014	107	204	0.52	[0.45; 0.59]	0.1%	0.7%
Chedraui, et al 2010	228	404	0.56	[0.51; 0.61]	0.2%	0.7%
Ojeda, et al 2014	434	771	0.56	[0.53; 0.60]	0.5%	0.7%
Ishizuka, et al 2008	409	1169	0.35	[0.32; 0.38]	0.7%	0.7%
Kapur, et al 2009	28	129	0.22	[0.15; 0.30]	0.1%	0.7%
Sharma, et al 2021	81	203	0.40	[0.33; 0.47]	0.1%	0.7%
Rahman, et al 2010	65	356	0.18	[0.14; 0.23]	0.2%	0.7%
Shea, et al 2006	100	399	0.25	[0.21; 0.30]	0.2%	0.7%
Waidyasekera, et al 2009	223	683	0.33	[0.29; 0.36]	0.4%	0.7%
Silva, et al 2013	761	1415	0.54	[0.51; 0.56]	0.8%	0.7%
Mart..nez, et al 2013	2826	10514	0.27	[0.26; 0.28]	6.2%	0.7%
Yang, et al 2008	1822	9617	0.19	[0.18; 0.20]	5.6%	0.7%
Du, et al 2020	1057	3141	0.34	[0.32; 0.35]	1.8%	0.7%
Singh, et al 2014	96	252	0.38	[0.32; 0.44]	0.1%	0.7%
Kulkarni, et al 2016	52	100	0.52	[0.42; 0.62]	0.1%	0.7%
Rathnayake, et al 2019	114	350	0.33	[0.28; 0.38]	0.2%	0.7%
Pateliya, et al 2022	26	100	0.26	[0.18; 0.36]	0.1%	0.7%
Srinivasan, et al 2021	16	100	0.16	[0.09; 0.25]	0.1%	0.7%
Chuni, et al 2011	360	729	0.49	[0.46; 0.53]	0.4%	0.7%
Nisar, et al 2015	2162	3062	0.71	[0.69; 0.72]	1.8%	0.7%
Zhang, et al 2016	63	106	0.59	[0.49; 0.69]	0.1%	0.7%
Agaba, et al 2017	64	107	0.60	[0.50; 0.69]	0.1%	0.7%
AlQuaiz, et al 2017	172	228	0.75	[0.69; 0.81]	0.1%	0.7%
Grigoriou, et al 2013	377	1025	0.37	[0.34; 0.40]	0.6%	0.7%
Haines, et al1 2005	101	248	0.41	[0.35; 0.47]	0.1%	0.7%
Haines, et al2 2005	95	195	0.49	[0.42; 0.56]	0.1%	0.7%
Haines, et al3 2005	23	59	0.39	[0.27; 0.53]	0.0%	0.6%
Haines, et al4 2005	41	97	0.42	[0.32; 0.53]	0.1%	0.7%
Haines, et al5 2005	17	24	0.71	[0.49; 0.87]	0.0%	0.6%
Haines, et al6 2005	27	58	0.47	[0.33; 0.60]	0.0%	0.6%
Haines, et al7 2005	41	81	0.51	[0.39; 0.62]	0.0%	0.7%
Haines, et al8 2005	80	150	0.53	[0.45; 0.62]	0.1%	0.7%
Haines, et al9 2005	94	100	0.94	[0.87; 0.98]	0.1%	0.7%
Dennerstein, et al 2000	9	172	0.05	[0.02; 0.10]	0.1%	0.7%
G..recka, et al 2022	127	349	0.36	[0.31; 0.42]	0.2%	0.7%
Saied, et al 2021	216	342	0.63	[0.58; 0.68]	0.2%	0.7%
Tan, et al 2014	129	309	0.42	[0.36; 0.47]	0.2%	0.7%
Chopra, et al 2022	317	504	0.63	[0.59; 0.67]	0.3%	0.7%
Zhao, et al 2023	925	1829	0.51	[0.48; 0.53]	1.1%	0.7%
Han, et al 2023	19	66	0.29	[0.18; 0.41]	0.0%	0.7%
Ko?o?lu, et al 2022	186	383	0.49	[0.43; 0.54]	0.2%	0.7%
AlDughaither, et al 2015	42	119	0.35	[0.27; 0.45]	0.1%	0.7%
Thakur,et al 2019	165	351	0.47	[0.42; 0.52]	0.2%	0.7%
Kim, et al 2017	312	743	0.42	[0.38; 0.46]	0.4%	0.7%
Loutfy, et al 2006	133	450	0.30	[0.25; 0.34]	0.3%	0.7%
Chedraui, et al 2014	227	451	0.50	[0.46; 0.55]	0.3%	0.7%
Suka, et al 2010	117	1169	0.10	[0.08; 0.12]	0.7%	0.7%
Rahman, et al 2011	97	509	0.19	[0.16; 0.23]	0.3%	0.7%
Agwu, et al 2008	21	186	0.11	[0.07; 0.17]	0.1%	0.7%
Ahmed, et al 2016	100	150	0.67	[0.59; 0.74]	0.1%	0.7%
Im, et al 2010	147	512	0.29	[0.25; 0.33]	0.3%	0.7%
Khatoon, et al 2018	38	121	0.31	[0.23; 0.40]	0.1%	0.7%
Budako?lu, et al 2007	138	338	0.41	[0.36; 0.46]	0.2%	0.7%
Sharanya Shre, et al 2016	74	150	0.49	[0.41; 0.58]	0.1%	0.7%
Damodaran, et al 2000	46	164	0.28	[0.21; 0.36]	0.1%	0.7%
Beura, et al 2020	50	104	0.48	[0.38; 0.58]	0.1%	0.7%
Thapa, et al 2020	107	177	0.60	[0.53; 0.68]	0.1%	0.7%
Ibrahim, et al 2015	837	1214	0.69	[0.66; 0.72]	0.7%	0.7%
Lee, et al 2020	9918	20882	0.47	[0.47; 0.48]	12.2%	0.7%
Kalhan, et al 2020	216	400	0.54	[0.49; 0.59]	0.2%	0.7%
Rindner, et al 2017	52	108	0.48	[0.38; 0.58]	0.1%	0.7%
Al−Musa, et al 2017	172	228	0.75	[0.69; 0.81]	0.1%	0.7%
Lawton, et al 2008	932	3616	0.26	[0.24; 0.27]	2.1%	0.7%
Resmi, et al 2020	95	400	0.24	[0.20; 0.28]	0.2%	0.7%
Ayranci, et al 2010	1119	1551	0.72	[0.70; 0.74]	0.9%	0.7%
Deveci, et al 2010	285	519	0.55	[0.51; 0.59]	0.3%	0.7%
O?urlu, et al 2011	41	132	0.31	[0.23; 0.40]	0.1%	0.7%
Anolue, et al 2012	154	349	0.44	[0.39; 0.50]	0.2%	0.7%
Mahajan,et al 2012	32	100	0.32	[0.23; 0.42]	0.1%	0.7%
Sassoon, et al 2014	13	63	0.21	[0.11; 0.33]	0.0%	0.6%
Kong, et al 2019	73	120	0.61	[0.52; 0.70]	0.1%	0.7%
Sharifi, et al 2019	592	600	0.99	[0.97; 0.99]	0.4%	0.7%
Pan, et al 2002	55	161	0.34	[0.27; 0.42]	0.1%	0.7%
Kasuga, et al 2004	617	1069	0.58	[0.55; 0.61]	0.6%	0.7%
Kaur, et al 2004	72	245	0.29	[0.24; 0.36]	0.1%	0.7%
Inayat, et al 2017	95	308	0.31	[0.26; 0.36]	0.2%	0.7%
Can..rio, et al 2012	172	370	0.46	[0.41; 0.52]	0.2%	0.7%
Rahman, et al 2020	286	390	0.73	[0.69; 0.78]	0.2%	0.7%
Diyu, et al 2022	240	318	0.75	[0.70; 0.80]	0.2%	0.7%
Zhang, et al 2016	692	2308	0.30	[0.28; 0.32]	1.4%	0.7%
Ryu, et al 2020	1271	2481	0.51	[0.49; 0.53]	1.5%	0.7%
Ma, et al 2017	753	2336	0.32	[0.30; 0.34]	1.4%	0.7%
Fu, et al 2020	678	3199	0.21	[0.20; 0.23]	1.9%	0.7%
Liu, et al 2018	2289	3382	0.68	[0.66; 0.69]	2.0%	0.7%
Li, et al 2016	334	1062	0.31	[0.29; 0.34]	0.6%	0.7%
Joshi, et al 2015	450	1000	0.45	[0.42; 0.48]	0.6%	0.7%
Ghimire, et al 2015	401	833	0.48	[0.45; 0.52]	0.5%	0.7%
Joseph, et al 2014	77	110	0.70	[0.61; 0.78]	0.1%	0.7%
Sweed, et al 2012	291	400	0.73	[0.68; 0.77]	0.2%	0.7%
Taher, et al 2012	37	86	0.43	[0.32; 0.54]	0.1%	0.7%
Bernis, et al 2007	271	1069	0.25	[0.23; 0.28]	0.6%	0.7%
Chedraui, et al 2007	27	300	0.09	[0.06; 0.13]	0.2%	0.7%
Pandey, et al 2020	30	189	0.16	[0.11; 0.22]	0.1%	0.7%
Obermeyer, et al 2004	85	293	0.29	[0.24; 0.35]	0.2%	0.7%
Zhao, et al 2000	334	806	0.41	[0.38; 0.45]	0.5%	0.7%
Gollschewski, et al 2004	301	851	0.35	[0.32; 0.39]	0.5%	0.7%
Biri, et al 2005	393	1049	0.37	[0.35; 0.40]	0.6%	0.7%
Obermeyer, et al 2002	102	299	0.34	[0.29; 0.40]	0.2%	0.7%
Loh, et al 2005	98	656	0.15	[0.12; 0.18]	0.4%	0.7%
Anderson, et al1 2004	301	851	0.35	[0.32; 0.39]	0.5%	0.7%
Anderson, et al2 2004	343	820	0.42	[0.38; 0.45]	0.5%	0.7%
Masjoudl, et al 2017	268	645	0.42	[0.38; 0.45]	0.4%	0.7%
Durairaj, et al 2022	47	74	0.64	[0.52; 0.74]	0.0%	0.7%
Abou−Raya, et al 2016	292	540	0.54	[0.50; 0.58]	0.3%	0.7%
Sierra, et al 2004	220	385	0.57	[0.52; 0.62]	0.2%	0.7%
Castelo−Branco, et al 2005	15	125	0.12	[0.07; 0.19]	0.1%	0.7%
Pe_rez−L.pez, et al 2013	563	1739	0.32	[0.30; 0.35]	1.0%	0.7%
Huang, et al 2020	784	1501	0.52	[0.50; 0.55]	0.9%	0.7%
Dasgupta, et al 2012	309	480	0.64	[0.60; 0.69]	0.3%	0.7%
Huseth−Zosel, et al 2014	104	296	0.35	[0.30; 0.41]	0.2%	0.7%
Wu, et al 2023	165	468	0.35	[0.31; 0.40]	0.3%	0.7%
Abdullah, et al 2016	113	258	0.44	[0.38; 0.50]	0.2%	0.7%
Chu, et al 2022	823	1748	0.47	[0.45; 0.49]	1.0%	0.7%
Hickey, et al 2017	321	1152	0.28	[0.25; 0.31]	0.7%	0.7%
Blell, et al 2014	51	253	0.20	[0.15; 0.26]	0.1%	0.7%
Dienye, et al 2013	220	385	0.57	[0.52; 0.62]	0.2%	0.7%
Whiteley, et al 2013	1075	8811	0.12	[0.12; 0.13]	5.2%	0.7%
Ruan, et al 2017	769	1225	0.63	[0.60; 0.65]	0.7%	0.7%
Xu, et al 2005	68	237	0.29	[0.23; 0.35]	0.1%	0.7%
Obermeyer, et al 2007	127	271	0.47	[0.41; 0.53]	0.2%	0.7%
Chae, et al 2014	263	570	0.46	[0.42; 0.50]	0.3%	0.7%
Oppermann, et al 2012	95	211	0.45	[0.38; 0.52]	0.1%	0.7%
An, et al 2023	3748	7309	0.51	[0.50; 0.52]	4.3%	0.7%
Kamal, et al 2017	367	1100	0.33	[0.31; 0.36]	0.6%	0.7%
Rajbhandari, et al 2017	303	2000	0.15	[0.14; 0.17]	1.2%	0.7%
Cortes, et al 2023	74	358	0.21	[0.17; 0.25]	0.2%	0.7%
Chalise, et al 2022	9	180	0.05	[0.02; 0.09]	0.1%	0.7%
**Common effect model**		**170526**	**0.36**	**[0.36; 0.36]**	**100.0%**	**--**
**Random effects model**			**0.42**	**[0.39; 0.45]**	**--**	**100.0%**

Heterogeneity: $I^2 = 99\%$, $\tau^2 = 0.0401$, $p = 0$

Supplementary Figure 4. Forest plot of prevalence of headaches among middle-aged women

Study	Events	Total	Proportion	95%−CI	Weight (common)	Weight (random)
Lan, et al 2017	363	985	0.37	[0.34; 0.40]	0.6%	0.8%
Wang, et al 2021	2294	6364	0.36	[0.35; 0.37]	3.9%	0.8%
Li, et al 2012	5962	20275	0.29	[0.29; 0.30]	12.4%	0.8%
Genazzani, et al1 2006	299	611	0.49	[0.45; 0.53]	0.4%	0.8%
Genazzani, et al2 2006	180	600	0.30	[0.26; 0.34]	0.4%	0.8%
Genazzani, et al3 2006	262	596	0.44	[0.40; 0.48]	0.4%	0.8%
Genazzani, et al4 2006	184	594	0.31	[0.27; 0.35]	0.4%	0.8%
Genazzani, et al5 2006	228	600	0.38	[0.34; 0.42]	0.4%	0.8%
Genazzani, et al6 2006	210	600	0.35	[0.31; 0.39]	0.4%	0.8%
Genazzani, et al7 2006	192	600	0.32	[0.28; 0.36]	0.4%	0.8%
Aaron, et al 2002	63	200	0.32	[0.25; 0.38]	0.1%	0.8%
Anderson, et al1 2004	382	712	0.54	[0.50; 0.57]	0.4%	0.8%
Anderson, et al2 2004	867	1430	0.61	[0.58; 0.63]	0.9%	0.8%
Fallahzadeh, et al 2007	19	346	0.05	[0.03; 0.08]	0.2%	0.8%
Fuh, et al 2003	432	1360	0.32	[0.29; 0.34]	0.8%	0.8%
Ishizuka, et al 2008	446	1169	0.38	[0.35; 0.41]	0.7%	0.8%
Kapur, et al 2009	56	129	0.43	[0.35; 0.52]	0.1%	0.8%
Qazi, et al 2006	562	800	0.70	[0.67; 0.73]	0.5%	0.8%
Shea, et al 2006	87	399	0.22	[0.18; 0.26]	0.2%	0.8%
Mart..nez, et al 2013	3186	10514	0.30	[0.29; 0.31]	6.5%	0.8%
Yang, et al 2008	2797	9617	0.29	[0.28; 0.30]	5.9%	0.8%
Singh, et al 2014	72	252	0.29	[0.23; 0.35]	0.2%	0.8%
Borker, et al 2013	77	106	0.73	[0.63; 0.81]	0.1%	0.8%
Pateliya, et al 2022	39	100	0.39	[0.29; 0.49]	0.1%	0.8%
Chim, et al 2002	149	495	0.30	[0.26; 0.34]	0.3%	0.8%
Dhillon,et al 2006	161	326	0.49	[0.44; 0.55]	0.2%	0.8%
Grigoriou, et al 2013	375	1025	0.37	[0.34; 0.40]	0.6%	0.8%
Dennerstein, et al 2000	38	172	0.22	[0.16; 0.29]	0.1%	0.8%
Nappi, et al1 2008	111	300	0.37	[0.32; 0.43]	0.2%	0.8%
Nappi, et al2 2008	109	300	0.36	[0.31; 0.42]	0.2%	0.8%
Nappi, et al3 2008	84	300	0.28	[0.23; 0.33]	0.2%	0.8%
Nappi, et al4 2008	69	301	0.23	[0.18; 0.28]	0.2%	0.8%
Nappi, et al5 2008	81	301	0.27	[0.22; 0.32]	0.2%	0.8%
Nappi, et al6 2008	63	301	0.21	[0.16; 0.26]	0.2%	0.8%
G..recka, et al 2022	173	349	0.50	[0.44; 0.55]	0.2%	0.8%
Han, et al 2023	38	66	0.58	[0.45; 0.70]	0.0%	0.7%
Thakur,et al 2019	154	351	0.44	[0.39; 0.49]	0.2%	0.8%
Kim, et al 2017	125	743	0.17	[0.14; 0.20]	0.5%	0.8%
Gerber, et al 2018	66	139	0.47	[0.39; 0.56]	0.1%	0.8%
Ghazanfarpour, et al 2016	63	202	0.31	[0.25; 0.38]	0.1%	0.8%
Duffy, et al 2012	1751	4279	0.41	[0.39; 0.42]	2.6%	0.8%
Loutfy, et al 2006	428	450	0.95	[0.93; 0.97]	0.3%	0.8%
Delavar, et al 2011	688	1397	0.49	[0.47; 0.52]	0.9%	0.8%
Chedraui, et al 2007	206	325	0.63	[0.58; 0.69]	0.2%	0.8%
Suka, et al 2010	175	1169	0.15	[0.13; 0.17]	0.7%	0.8%
Rahman, et al 2011	452	509	0.89	[0.86; 0.91]	0.3%	0.8%
Ahmed, et al 2016	99	150	0.66	[0.58; 0.74]	0.1%	0.8%
Berecki−Gisolf, et al 2009	1282	8648	0.15	[0.14; 0.16]	5.3%	0.8%
Bener, et al 2014	570	1158	0.49	[0.46; 0.52]	0.7%	0.8%
Bener, et al 2000	181	390	0.46	[0.41; 0.51]	0.2%	0.8%
Im, et al 2010	124	512	0.24	[0.21; 0.28]	0.3%	0.8%
Ande, et al 2011	118	533	0.22	[0.19; 0.26]	0.3%	0.8%
Damodaran, et al 2000	42	164	0.26	[0.19; 0.33]	0.1%	0.8%
Liu, et al 2007	144	267	0.54	[0.48; 0.60]	0.2%	0.8%
Lee, et al 2020	9086	20882	0.44	[0.43; 0.44]	12.8%	0.8%
Modoodi, et al 2020	276	350	0.79	[0.74; 0.83]	0.2%	0.8%
Moilanen, et al 2010	99	1184	0.08	[0.07; 0.10]	0.7%	0.8%
Bairy, et al 2009	129	352	0.37	[0.32; 0.42]	0.2%	0.8%
Lawton, et al 2008	1225	3616	0.34	[0.32; 0.35]	2.2%	0.8%
Liu, et al 2008	94	217	0.43	[0.37; 0.50]	0.1%	0.8%
Liu, et al 2007	130	188	0.69	[0.62; 0.76]	0.1%	0.8%
Resmi, et al 2020	101	400	0.25	[0.21; 0.30]	0.2%	0.8%
Deveci, et al 2010	355	519	0.68	[0.64; 0.72]	0.3%	0.8%
O?urlu, et al 2011	46	132	0.35	[0.27; 0.44]	0.1%	0.8%
Anolue, et al 2012	148	349	0.42	[0.37; 0.48]	0.2%	0.8%
Mahajan,et al 2012	46	100	0.46	[0.36; 0.56]	0.1%	0.8%
Nappi, et al 2012	810	3520	0.23	[0.22; 0.24]	2.2%	0.8%
Sassoon, et al 2014	31	63	0.49	[0.36; 0.62]	0.0%	0.7%
Liu, et al 2013	1093	1686	0.65	[0.62; 0.67]	1.0%	0.8%
Mohamed, et al 2014	71	90	0.79	[0.69; 0.87]	0.1%	0.8%
Holm, et al 2000	116	214	0.54	[0.47; 0.61]	0.1%	0.8%
Pan, et al 2002	45	161	0.28	[0.21; 0.36]	0.1%	0.8%
Kasuga, et al 2004	673	1069	0.63	[0.60; 0.66]	0.7%	0.8%
Kaur, et al 2004	60	245	0.24	[0.19; 0.30]	0.2%	0.8%
Gupta, et al1 2006	28	103	0.27	[0.19; 0.37]	0.1%	0.8%
Gupta, et al2 2006	6	50	0.12	[0.05; 0.24]	0.0%	0.7%
Inayat, et al 2017	99	308	0.32	[0.27; 0.38]	0.2%	0.8%
Can..rio, et al 2012	170	370	0.46	[0.41; 0.51]	0.2%	0.8%
Abedzadeh−Kalahroudi, et al 2012	581	700	0.83	[0.80; 0.86]	0.4%	0.8%
Zhang, et al 2016	831	2308	0.36	[0.34; 0.38]	1.4%	0.8%
Ma, et al 2017	923	2336	0.40	[0.38; 0.42]	1.4%	0.8%
Fu, et al 2020	1263	3199	0.39	[0.38; 0.41]	2.0%	0.8%
Liu, et al 2018	2132	3382	0.63	[0.61; 0.65]	2.1%	0.8%
Li, et al 2016	414	1062	0.39	[0.36; 0.42]	0.7%	0.8%
Srivastava, et al 2014	65	117	0.56	[0.46; 0.65]	0.1%	0.8%
ROKHADE, et al 2013	42	115	0.37	[0.28; 0.46]	0.1%	0.8%
G K, et al 2013	355	500	0.71	[0.67; 0.75]	0.3%	0.8%
Bernis, et al 2007	450	1069	0.42	[0.39; 0.45]	0.7%	0.8%
Fabbrini, et al 2015	159	334	0.48	[0.42; 0.53]	0.2%	0.8%
Ward, et al 2010	486	1115	0.44	[0.41; 0.47]	0.7%	0.8%
Sievert, et al 2007	435	869	0.50	[0.47; 0.53]	0.5%	0.8%
Sievert, et al 2003	401	755	0.53	[0.49; 0.57]	0.5%	0.8%
Obermeyer, et al 2004	170	293	0.58	[0.52; 0.64]	0.2%	0.8%
Zhao, et al 2000	266	806	0.33	[0.30; 0.36]	0.5%	0.8%
Lam, et al 2003	419	978	0.43	[0.40; 0.46]	0.6%	0.8%
Gollschewski, et al 2004	452	862	0.52	[0.49; 0.56]	0.5%	0.8%
Biri, et al 2005	490	1049	0.47	[0.44; 0.50]	0.6%	0.8%
Obermeyer, et al 2002	173	299	0.58	[0.52; 0.64]	0.2%	0.8%
Loh, et al 2005	269	656	0.41	[0.37; 0.45]	0.4%	0.8%
Chaopotong, et al 2005	110	148	0.74	[0.67; 0.81]	0.1%	0.8%
Huang, et al1 2010	170	300	0.57	[0.51; 0.62]	0.2%	0.8%
Huang, et al2 2010	63	100	0.63	[0.53; 0.72]	0.1%	0.8%
Huang, et al3 2010	163	300	0.54	[0.49; 0.60]	0.2%	0.8%
Huang, et al4 2010	78	150	0.52	[0.44; 0.60]	0.1%	0.8%
Huang, et al5 2010	86	150	0.57	[0.49; 0.65]	0.1%	0.8%
Anderson, et al1 2004	452	862	0.52	[0.49; 0.56]	0.5%	0.8%
Anderson, et al2 2004	464	827	0.56	[0.53; 0.60]	0.5%	0.8%
Sierra, et al 2004	323	385	0.84	[0.80; 0.87]	0.2%	0.8%
Castelo−Branco, et al 2005	28	125	0.22	[0.15; 0.31]	0.1%	0.8%
Jin, et al 2014	348	1215	0.29	[0.26; 0.31]	0.7%	0.8%
Huang, et al 2020	659	1501	0.44	[0.41; 0.46]	0.9%	0.8%
Dasgupta, et al 2012	229	480	0.48	[0.43; 0.52]	0.3%	0.8%
Wu, et al 2023	271	468	0.58	[0.53; 0.62]	0.3%	0.8%
Chu, et al 2022	723	1748	0.41	[0.39; 0.44]	1.1%	0.8%
Blell, et al 2014	96	253	0.38	[0.32; 0.44]	0.2%	0.8%
Olowokere, et al 2020	205	271	0.76	[0.70; 0.81]	0.2%	0.8%
Ruan, et al 2017	696	1225	0.57	[0.54; 0.60]	0.8%	0.8%
Xu, et al 2005	129	237	0.54	[0.48; 0.61]	0.1%	0.8%
Shakhatreh, et al 2006	66	143	0.46	[0.38; 0.55]	0.1%	0.8%
Obermeyer, et al 2007	141	271	0.52	[0.46; 0.58]	0.2%	0.8%
Al−Qutob, et al 2000	30	137	0.22	[0.15; 0.30]	0.1%	0.8%
Oppermann, et al 2012	127	211	0.60	[0.53; 0.67]	0.1%	0.8%
An, et al 2023	4326	7309	0.59	[0.58; 0.60]	4.5%	0.8%
Cortes, et al 2023	169	358	0.47	[0.42; 0.53]	0.2%	0.8%
Chalise, et al 2022	44	180	0.24	[0.18; 0.31]	0.1%	0.8%
**Common effect model**		**162907**	**0.38**	**[0.38; 0.39]**	**100.0%**	**--**
**Random effects model**			**0.44**	**[0.41; 0.47]**	**--**	**100.0%**

Heterogeneity: $I^2 = 99\%$, $\tau^2 = 0.0346$, $p = 0$

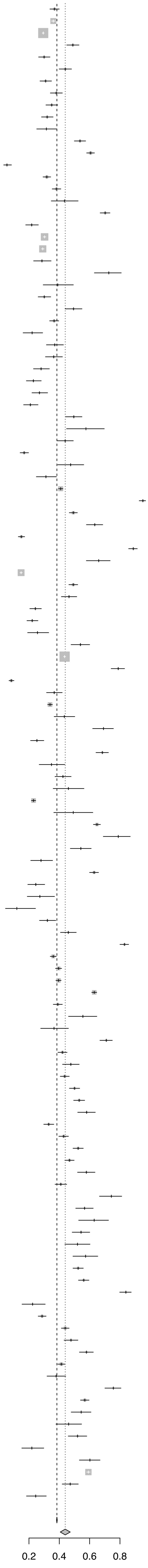

Supplementary Figure 5. Forest plot of prevalence of joint and muscular discomfort among middle-aged women

Study	Events	Total	Proportion	95%–CI	Weight (common)	Weight (random)
Lan, et al 2017	537	985	0.55	[0.51; 0.58]	0.4%	0.5%
Adhikari, et al 2022	214	252	0.85	[0.80; 0.89]	0.1%	0.5%
Wang, et al 2021	2009	6364	0.32	[0.30; 0.33]	2.7%	0.5%
Olaolorun, et al 2009	702	1189	0.59	[0.56; 0.62]	0.5%	0.5%
Li, et al 2012	7847	20275	0.39	[0.38; 0.39]	8.6%	0.5%
Javadivala, et al 2020	114	161	0.71	[0.63; 0.78]	0.1%	0.5%
Nagaraj, et al 2021	127	200	0.64	[0.56; 0.70]	0.1%	0.5%
Yisma, et al 2017	73	226	0.32	[0.26; 0.39]	0.1%	0.5%
Korkmaz, et al 2022	69	108	0.64	[0.54; 0.73]	0.0%	0.5%
AlQuaiz, et al 2013	411	490	0.84	[0.80; 0.87]	0.2%	0.5%
Abdel–Salam, et al 2021	678	845	0.80	[0.77; 0.83]	0.4%	0.5%
Anderson, et al1 2004	555	712	0.78	[0.75; 0.81]	0.3%	0.5%
Anderson, et al2 2004	1007	1430	0.70	[0.68; 0.73]	0.6%	0.5%
Shafie, et al 2011	351	472	0.74	[0.70; 0.78]	0.2%	0.5%
Aloufi, et al 2022	286	361	0.79	[0.75; 0.83]	0.2%	0.5%
Chou, et al 2014	391	442	0.88	[0.85; 0.91]	0.2%	0.5%
Fallahzadeh, et al 2007	286	346	0.83	[0.78; 0.86]	0.1%	0.5%
Fuh, et al 2003	448	1360	0.33	[0.30; 0.36]	0.6%	0.5%
Chedraui, et al 2014	178	204	0.87	[0.82; 0.92]	0.1%	0.5%
Chedraui, et al 2010	323	404	0.80	[0.76; 0.84]	0.2%	0.5%
Ojeda, et al 2014	600	771	0.78	[0.75; 0.81]	0.3%	0.5%
Ishizuka, et al 2008	881	1169	0.75	[0.73; 0.78]	0.5%	0.5%
Kapur, et al 2009	72	129	0.56	[0.47; 0.65]	0.1%	0.5%
Sharma, et al 2021	158	203	0.78	[0.71; 0.83]	0.1%	0.5%
Qazi, et al 2006	474	800	0.59	[0.56; 0.63]	0.3%	0.5%
Rahman, et al 2010	285	356	0.80	[0.76; 0.84]	0.2%	0.5%
Shea, et al 2006	176	399	0.44	[0.39; 0.49]	0.2%	0.5%
Som, et al 2012	170	250	0.68	[0.62; 0.74]	0.1%	0.5%
Alwi, et al 2009	228	276	0.83	[0.78; 0.87]	0.1%	0.5%
Waidyasekera, et al 2009	510	683	0.75	[0.71; 0.78]	0.3%	0.5%
Silva, et al 2013	1058	1415	0.75	[0.72; 0.77]	0.6%	0.5%
Mart..nez, et al 2013	4115	10514	0.39	[0.38; 0.40]	4.4%	0.5%
Yang, et al 2008	3436	9617	0.36	[0.35; 0.37]	4.1%	0.5%
Yim, et al 2015	1248	1774	0.70	[0.68; 0.72]	0.7%	0.5%
Yokota, et al 2016	1104	1961	0.56	[0.54; 0.59]	0.8%	0.5%
Du, et al 2020	905	3141	0.29	[0.27; 0.30]	1.3%	0.5%
Singh, et al 2014	149	252	0.59	[0.53; 0.65]	0.1%	0.5%
Kulkarni, et al 2016	92	100	0.92	[0.85; 0.96]	0.0%	0.5%
Borker, et al 2013	56	106	0.53	[0.43; 0.63]	0.0%	0.5%
Rathnayake, et al 2019	290	350	0.83	[0.78; 0.87]	0.1%	0.5%
Pateliya, et al 2022	78	100	0.78	[0.69; 0.86]	0.0%	0.5%
Srinivasan, et al 2021	80	100	0.80	[0.71; 0.87]	0.0%	0.5%
Chim, et al 2002	256	495	0.52	[0.47; 0.56]	0.2%	0.5%
Dhillon,et al 2006	230	326	0.71	[0.65; 0.75]	0.1%	0.5%
Chuni, et al 2011	500	729	0.69	[0.65; 0.72]	0.3%	0.5%
Nisar, et al 2015	2567	3062	0.84	[0.82; 0.85]	1.3%	0.5%
Zhang, et al 2016	85	106	0.80	[0.71; 0.87]	0.0%	0.5%
Agaba, et al 2017	66	107	0.62	[0.52; 0.71]	0.0%	0.5%
AlQuaiz, et al 2017	219	228	0.96	[0.93; 0.98]	0.1%	0.5%
Grigoriou, et al 2013	485	1025	0.47	[0.44; 0.50]	0.4%	0.5%
Haines, et al1 2005	202	248	0.81	[0.76; 0.86]	0.1%	0.5%
Haines, et al2 2005	175	195	0.90	[0.85; 0.94]	0.1%	0.5%
Haines, et al3 2005	55	59	0.93	[0.84; 0.98]	0.0%	0.5%
Haines, et al4 2005	74	97	0.76	[0.67; 0.84]	0.0%	0.5%
Haines, et al5 2005	23	24	0.96	[0.79; 1.00]	0.0%	0.4%
Haines, et al6 2005	47	58	0.81	[0.69; 0.90]	0.0%	0.5%
Haines, et al7 2005	67	81	0.83	[0.73; 0.90]	0.0%	0.5%
Haines, et al8 2005	135	150	0.90	[0.84; 0.94]	0.1%	0.5%
Haines, et al9 2005	96	100	0.96	[0.90; 0.99]	0.0%	0.5%
Dennerstein, et al 2000	41	172	0.24	[0.18; 0.31]	0.1%	0.5%
G..recka, et al 2022	191	349	0.55	[0.49; 0.60]	0.1%	0.5%
Saied, et al 2021	318	342	0.93	[0.90; 0.95]	0.1%	0.5%
Sheereen, et al 2022	94	100	0.94	[0.87; 0.98]	0.0%	0.5%
Kang, et al 2021	133	150	0.89	[0.82; 0.93]	0.1%	0.5%
Senthilvel, et al 2018	136	150	0.91	[0.85; 0.95]	0.1%	0.5%
Karmakar, et al 2017	84	100	0.84	[0.75; 0.91]	0.0%	0.5%
Ganapathy, et al 2018	135	140	0.96	[0.92; 0.99]	0.1%	0.5%
Tan, et al 2014	201	309	0.65	[0.59; 0.70]	0.1%	0.5%
Chopra, et al 2022	395	504	0.78	[0.75; 0.82]	0.2%	0.5%
Vaccaro, et al 2021	325	1028	0.32	[0.29; 0.35]	0.4%	0.5%
Zhao, et al 2023	1203	1829	0.66	[0.64; 0.68]	0.8%	0.5%
Han, et al 2023	40	66	0.61	[0.48; 0.72]	0.0%	0.5%
Ko?o?lu, et al 2022	298	383	0.78	[0.73; 0.82]	0.2%	0.5%
AlDughaither, et al 2015	96	119	0.81	[0.72; 0.87]	0.1%	0.5%
Ceylan, et al 2014	691	874	0.79	[0.76; 0.82]	0.4%	0.5%
Thakur,et al 2019	176	351	0.50	[0.45; 0.55]	0.1%	0.5%
Kim, et al 2017	280	743	0.38	[0.34; 0.41]	0.3%	0.5%
Sievert, et al 2022	132	162	0.81	[0.75; 0.87]	0.1%	0.5%
Gerber, et al 2018	71	139	0.51	[0.42; 0.60]	0.1%	0.5%
Ghazanfarpour, et al 2016	73	202	0.36	[0.30; 0.43]	0.1%	0.5%
Duffy, et al 2012	2470	4288	0.58	[0.56; 0.59]	1.8%	0.5%
Loutfy, et al 2006	325	450	0.72	[0.68; 0.76]	0.2%	0.5%
Delavar, et al 2011	988	1397	0.71	[0.68; 0.73]	0.6%	0.5%
Chedraui, et al 2007	274	325	0.84	[0.80; 0.88]	0.1%	0.5%
Chedraui, et al 2014	399	451	0.88	[0.85; 0.91]	0.2%	0.5%
Suka, et al 2010	541	1169	0.46	[0.43; 0.49]	0.5%	0.5%
Rahman, et al 2011	388	509	0.76	[0.72; 0.80]	0.2%	0.5%
Agwu, et al 2008	48	186	0.26	[0.20; 0.33]	0.1%	0.5%
Ahmed, et al 2016	96	150	0.64	[0.56; 0.72]	0.1%	0.5%
Berecki–Gisolf, et al 2009	1399	8648	0.16	[0.15; 0.17]	3.7%	0.5%
Bener, et al 2014	413	1158	0.36	[0.33; 0.39]	0.5%	0.5%
Bener, et al 2000	135	390	0.35	[0.30; 0.40]	0.2%	0.5%
Im, et al 2010	266	512	0.52	[0.48; 0.56]	0.2%	0.5%
Ande, et al 2011	287	533	0.54	[0.50; 0.58]	0.2%	0.5%
Khatoon, et al 2018	83	121	0.69	[0.60; 0.77]	0.1%	0.5%
Budako?lu, et al 2007	174	338	0.51	[0.46; 0.57]	0.1%	0.5%
Sharanya Shre, et al 2016	116	150	0.77	[0.70; 0.84]	0.1%	0.5%
Damodaran, et al 2000	110	164	0.67	[0.59; 0.74]	0.1%	0.5%
Beura, et al 2020	98	104	0.94	[0.88; 0.98]	0.0%	0.5%
Thapa, et al 2020	141	177	0.80	[0.73; 0.85]	0.1%	0.5%
Ibrahim, et al 2015	1030	1214	0.85	[0.83; 0.87]	0.5%	0.5%
Nusrat, et al 2008	653	863	0.76	[0.73; 0.78]	0.4%	0.5%
Liu, et al 2007	182	267	0.68	[0.62; 0.74]	0.1%	0.5%
Lee, et al 2020	12152	20882	0.58	[0.58; 0.59]	8.8%	0.5%
Mododi, et al 2020	189	350	0.54	[0.49; 0.59]	0.1%	0.5%
Kalhan, et al 2020	224	400	0.56	[0.51; 0.61]	0.2%	0.5%
Rindner, et al 2017	66	108	0.61	[0.51; 0.70]	0.0%	0.5%
Al–Musa, et al 2017	219	228	0.96	[0.93; 0.98]	0.1%	0.5%
Moilanen, et al 2010	265	1184	0.22	[0.20; 0.25]	0.5%	0.5%
Bairy, et al 2009	236	352	0.67	[0.62; 0.72]	0.1%	0.5%
Heinemann, et al1 2008	3186	4791	0.66	[0.65; 0.68]	2.0%	0.5%
Heinemann, et al2 2008	1010	1500	0.67	[0.65; 0.70]	0.6%	0.5%
Heinemann, et al3 2008	1849	3006	0.62	[0.60; 0.63]	1.3%	0.5%
Heinemann, et al4 2008	751	1000	0.75	[0.72; 0.78]	0.4%	0.5%
Lawton, et al 2008	2317	3616	0.64	[0.62; 0.66]	1.5%	0.5%
Liu, et al 2008	129	217	0.59	[0.53; 0.66]	0.1%	0.5%
Liu, et al 2007	159	188	0.85	[0.79; 0.89]	0.1%	0.5%
Im, et al 2009	82	158	0.52	[0.44; 0.60]	0.1%	0.5%
Resmi, et al 2020	143	400	0.36	[0.31; 0.41]	0.2%	0.5%
Ashrafi, et al 2009	1755	2462	0.71	[0.69; 0.73]	1.0%	0.5%
Deveci, et al 2010	269	519	0.52	[0.47; 0.56]	0.2%	0.5%
O?urlu, et al 2011	85	132	0.64	[0.56; 0.73]	0.1%	0.5%
Anolue, et al 2012	272	349	0.78	[0.73; 0.82]	0.1%	0.5%
Mahajan,et al 2012	51	100	0.51	[0.41; 0.61]	0.0%	0.5%
Nappi, et al 2012	1056	3520	0.30	[0.28; 0.32]	1.5%	0.5%
Sassoon, et al 2014	38	63	0.60	[0.47; 0.72]	0.0%	0.5%
Liu, et al 2013	1173	1686	0.70	[0.67; 0.72]	0.7%	0.5%
Mohamed, et al 2014	74	90	0.82	[0.73; 0.89]	0.0%	0.5%
Kong, et al 2019	88	120	0.73	[0.64; 0.81]	0.1%	0.5%
Sharifi, et al 2019	591	600	0.98	[0.97; 0.99]	0.3%	0.5%
Holm, et al 2000	133	214	0.62	[0.55; 0.69]	0.1%	0.5%
Kasuga, et al 2004	913	1069	0.85	[0.83; 0.87]	0.5%	0.5%
Kaur, et al 2004	92	245	0.38	[0.31; 0.44]	0.1%	0.5%
Gupta, et al1 2006	44	103	0.43	[0.33; 0.53]	0.0%	0.5%
Gupta, et al2 2006	7	50	0.14	[0.06; 0.27]	0.0%	0.4%
Can..rio, et al 2012	267	370	0.72	[0.67; 0.77]	0.2%	0.5%
Rahman, et al 2020	330	390	0.85	[0.81; 0.88]	0.2%	0.5%
Abedzadeh–Kalahroudi, et al 2012	619	700	0.88	[0.86; 0.91]	0.3%	0.5%
Diyu, et al 2022	274	318	0.86	[0.82; 0.90]	0.1%	0.5%
Zhang, et al 2016	1062	2308	0.46	[0.44; 0.48]	1.0%	0.5%
Ryu, et al 2020	1506	2481	0.61	[0.59; 0.63]	1.0%	0.5%
Ma, et al 2017	890	2336	0.38	[0.36; 0.40]	1.0%	0.5%
Fu, et al 2020	1074	3199	0.34	[0.32; 0.35]	1.4%	0.5%
Liu, et al 2018	2218	3382	0.66	[0.64; 0.67]	1.4%	0.5%
Li, et al 2016	398	1062	0.37	[0.35; 0.40]	0.5%	0.5%
Joshi, et al 2015	736	1000	0.74	[0.71; 0.76]	0.4%	0.5%
Ghimire, et al 2015	594	833	0.71	[0.68; 0.74]	0.4%	0.5%
Joseph, et al 2014	94	110	0.85	[0.77; 0.91]	0.0%	0.5%
Bindhu, et al 2014	115	320	0.36	[0.31; 0.41]	0.1%	0.5%
ROKHADE, et al 2013	59	115	0.51	[0.42; 0.61]	0.0%	0.5%
Sweed, et al 2012	361	400	0.90	[0.87; 0.93]	0.2%	0.5%
Taher, et al 2012	64	86	0.74	[0.64; 0.83]	0.0%	0.5%
G K, et al 2013	395	500	0.79	[0.75; 0.82]	0.2%	0.5%
Bernis, et al 2007	684	1069	0.64	[0.61; 0.67]	0.5%	0.5%
Chedraui, et al 2007	231	300	0.77	[0.72; 0.82]	0.1%	0.5%
Nayak, et al 2019	254	290	0.88	[0.83; 0.91]	0.1%	0.5%
Hsu, et al 2005	72	197	0.37	[0.30; 0.44]	0.1%	0.5%
Ward, et al 2010	601	1115	0.54	[0.51; 0.57]	0.5%	0.5%
Pandey, et al 2020	97	189	0.51	[0.44; 0.59]	0.1%	0.5%
Sievert, et al 2007	556	869	0.64	[0.61; 0.67]	0.4%	0.5%
Sievert, et al 2003	420	755	0.56	[0.52; 0.59]	0.3%	0.5%
Obermeyer, et al 2004	158	293	0.54	[0.48; 0.60]	0.1%	0.5%
Jansson, et al 2003	573	1125	0.51	[0.48; 0.54]	0.5%	0.5%
Zhao, et al 2000	309	806	0.38	[0.35; 0.42]	0.3%	0.5%
Lam, et al 2003	553	978	0.57	[0.53; 0.60]	0.4%	0.5%
Gollschewski, et al 2004	673	863	0.78	[0.75; 0.81]	0.4%	0.5%
Biri, et al 2005	572	1049	0.55	[0.51; 0.58]	0.4%	0.5%
Obermeyer, et al 2002	161	299	0.54	[0.48; 0.60]	0.1%	0.5%
Loh, et al 2005	345	656	0.53	[0.49; 0.56]	0.3%	0.5%
Chaopotong, et al 2005	125	148	0.84	[0.78; 0.90]	0.1%	0.5%
Anderson, et al1 2004	673	863	0.78	[0.75; 0.81]	0.4%	0.5%
Anderson, et al2 2004	596	830	0.72	[0.69; 0.75]	0.4%	0.5%
Masjoudi, et al 2017	473	645	0.73	[0.70; 0.77]	0.3%	0.5%
Durairaj, et al 2022	66	74	0.89	[0.80; 0.95]	0.0%	0.5%
Bala, et al 2022	218	245	0.89	[0.84; 0.93]	0.1%	0.5%
Abou–Raya, et al 2016	501	540	0.93	[0.90; 0.95]	0.2%	0.5%
Ogwumike, et al 2012	250	547	0.46	[0.41; 0.50]	0.2%	0.5%
Gao, et al 2013	248	743	0.33	[0.30; 0.37]	0.3%	0.5%
Sierra, et al 2004	306	385	0.79	[0.75; 0.83]	0.2%	0.5%
Castelo–Branco, et al 2005	18	125	0.14	[0.09; 0.22]	0.1%	0.5%
Ahn, et al 2009	99	134	0.74	[0.66; 0.81]	0.1%	0.5%
Jin, et al 2014	697	1215	0.57	[0.55; 0.60]	0.5%	0.5%
Pe_rez–L.pez, et al 2013	1239	1739	0.71	[0.69; 0.73]	0.7%	0.5%
Huang, et al 2020	869	1501	0.58	[0.55; 0.60]	0.6%	0.5%
Dasgupta, et al 2012	355	480	0.74	[0.70; 0.78]	0.2%	0.5%
Huseth–Zosel, et al 2014	14	296	0.05	[0.03; 0.08]	0.1%	0.5%
Legorreta, et al 2013	2401	4548	0.53	[0.51; 0.54]	1.9%	0.5%
Wu, et al 2023	284	468	0.61	[0.56; 0.65]	0.2%	0.5%
Abdullah, et al 2016	189	258	0.73	[0.67; 0.79]	0.1%	0.5%
Chu, et al 2022	1002	1748	0.57	[0.55; 0.60]	0.7%	0.5%
Hickey, et al 2017	720	1145	0.63	[0.60; 0.66]	0.5%	0.5%
Blell, et al 2014	106	254	0.42	[0.36; 0.48]	0.1%	0.5%
Dienye, et al 2013	337	385	0.88	[0.84; 0.91]	0.2%	0.5%
Whiteley, et al 2013	1703	8811	0.19	[0.19; 0.20]	3.7%	0.5%
Olowokere, et al 2020	228	271	0.84	[0.79; 0.88]	0.1%	0.5%
Zhu, et al 2021	1099	2400	0.46	[0.44; 0.48]	1.0%	0.5%
Ruan, et al 2017	672	1225	0.55	[0.52; 0.58]	0.5%	0.5%
Xu, et al 2005	135	237	0.57	[0.50; 0.63]	0.1%	0.5%
Islam, et al 2016	888	1590	0.56	[0.53; 0.58]	0.7%	0.5%
Shakhatreh, et al 2006	126	143	0.88	[0.82; 0.93]	0.1%	0.5%
Obermeyer, et al 2007	119	271	0.44	[0.38; 0.50]	0.1%	0.5%
Chae, et al 2014	279	570	0.49	[0.45; 0.53]	0.2%	0.5%
Oppermann, et al 2012	111	211	0.53	[0.46; 0.60]	0.1%	0.5%
An, et al 2023	4432	7309	0.61	[0.60; 0.62]	3.1%	0.5%
Kamal, et al 2017	903	1100	0.82	[0.80; 0.84]	0.5%	0.5%
Rajbhandari, et al 2017	736	2000	0.37	[0.35; 0.39]	0.8%	0.5%
Cortes, et al 2023	187	358	0.52	[0.47; 0.58]	0.2%	0.5%
Chalise, et al 2022	31	180	0.17	[0.12; 0.24]	0.1%	0.5%
**Common effect model**		**236615**	**0.53**	**[0.53; 0.53]**	**100.0%**	**--**
**Random effects model**			**0.65**	**[0.63; 0.68]**	**--**	**100.0%**

Heterogeneity: $I^2 = 99\%$, $\tau^2 = 0.0485$, $p = 0$

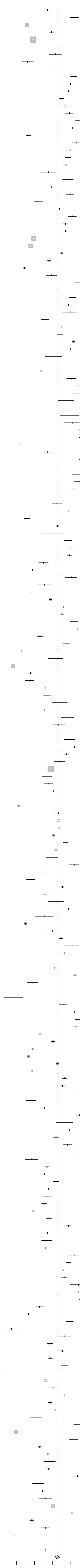

Supplementary Figure 6. Forest plot of prevalence of depression among middle-aged women

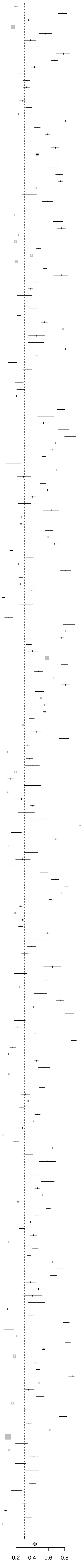

Study	Events	Total	Proportion	95%−CI	Weight (common)	Weight (random)
Lan, et al 2017	197	985	0.20	[0.18; 0.23]	0.3%	0.4%
Adhikari, et al 2022	195	252	0.77	[0.72; 0.82]	0.1%	0.4%
Olaolorun, et al 2009	424	1189	0.36	[0.33; 0.38]	0.4%	0.4%
Li, et al 2012	3284	20275	0.16	[0.16; 0.17]	6.7%	0.4%
Javadivala, et al 2020	91	161	0.57	[0.48; 0.64]	0.1%	0.4%
Nagaraj, et al 2021	76	200	0.38	[0.31; 0.45]	0.1%	0.4%
Yisma, et al 2017	104	226	0.46	[0.39; 0.53]	0.1%	0.4%
Korkmaz, et al 2022	85	108	0.79	[0.70; 0.86]	0.0%	0.4%
AlQuaiz, et al 2013	331	490	0.68	[0.63; 0.72]	0.2%	0.4%
Genazzani, et al1 2006	263	611	0.43	[0.39; 0.47]	0.2%	0.4%
Genazzani, et al2 2006	150	600	0.25	[0.22; 0.29]	0.2%	0.4%
Genazzani, et al3 2006	197	596	0.33	[0.29; 0.37]	0.2%	0.4%
Genazzani, et al4 2006	196	594	0.33	[0.29; 0.37]	0.2%	0.4%
Genazzani, et al5 2006	180	600	0.30	[0.26; 0.34]	0.2%	0.4%
Genazzani, et al6 2006	168	600	0.28	[0.24; 0.32]	0.2%	0.4%
Genazzani, et al7 2006	216	600	0.36	[0.32; 0.40]	0.2%	0.4%
Aaron, et al 2002	47	200	0.23	[0.18; 0.30]	0.1%	0.4%
Abdel−Salam, et al 2021	686	845	0.81	[0.78; 0.84]	0.3%	0.4%
Anderson, et al1 2004	330	712	0.46	[0.43; 0.50]	0.2%	0.4%
Anderson, et al2 2004	841	1430	0.59	[0.56; 0.61]	0.5%	0.4%
Shafie, et al 2011	182	472	0.39	[0.34; 0.43]	0.2%	0.4%
Aloufi, et al 2022	248	361	0.69	[0.64; 0.73]	0.1%	0.4%
Bl..mel, et al 2012	2827	6079	0.47	[0.45; 0.48]	2.0%	0.4%
Chou, et al 2014	317	442	0.72	[0.67; 0.76]	0.1%	0.4%
Chedraui, et al 2014	132	204	0.65	[0.58; 0.71]	0.1%	0.4%
Chedraui, et al 2010	297	404	0.74	[0.69; 0.78]	0.1%	0.4%
Ojeda, et al 2014	578	771	0.75	[0.72; 0.78]	0.3%	0.4%
Ishizuka, et al 2008	526	1169	0.45	[0.42; 0.48]	0.4%	0.4%
Kapur, et al 2009	47	129	0.36	[0.28; 0.45]	0.0%	0.4%
Sharma, et al 2021	120	203	0.59	[0.52; 0.66]	0.1%	0.4%
Rahman, et al 2010	116	356	0.33	[0.28; 0.38]	0.1%	0.4%
Shea, et al 2006	72	399	0.18	[0.14; 0.22]	0.1%	0.4%
Som, et al 2012	180	250	0.72	[0.66; 0.77]	0.1%	0.4%
Alwi, et al 2009	210	276	0.76	[0.71; 0.81]	0.1%	0.4%
Waidyasekera, et al 2009	161	683	0.24	[0.20; 0.27]	0.2%	0.4%
Malacara, et al 2002	1485	7635	0.19	[0.19; 0.20]	2.5%	0.4%
Silva, et al 2013	680	1415	0.48	[0.45; 0.51]	0.5%	0.4%
Mart..nez, et al 2013	4117	10514	0.39	[0.38; 0.40]	3.5%	0.4%
Yang, et al 2008	2027	9617	0.21	[0.20; 0.22]	3.2%	0.4%
Yim, et al 2015	991	1774	0.56	[0.54; 0.58]	0.6%	0.4%
Kulkarni, et al 2016	76	100	0.76	[0.66; 0.84]	0.0%	0.4%
Rathnayake, et al 2019	166	350	0.47	[0.42; 0.53]	0.1%	0.4%
Baquedano, et al 2023	401	1059	0.38	[0.35; 0.41]	0.4%	0.4%
Pateliya, et al 2022	30	100	0.30	[0.21; 0.40]	0.0%	0.4%
Srinivasan, et al 2021	34	100	0.34	[0.25; 0.44]	0.0%	0.4%
Dhillon,et al 2006	134	326	0.41	[0.36; 0.47]	0.1%	0.4%
Li, et al 2008	306	1280	0.24	[0.22; 0.26]	0.4%	0.4%
Chuni, et al 2011	402	729	0.55	[0.51; 0.59]	0.2%	0.4%
Nisar, et al 2015	2389	3062	0.78	[0.77; 0.79]	1.0%	0.4%
Zhang, et al 2016	48	106	0.45	[0.36; 0.55]	0.0%	0.4%
Agaba, et al 2017	48	107	0.45	[0.35; 0.55]	0.0%	0.4%
AlQuaiz, et al 2017	185	228	0.81	[0.75; 0.86]	0.1%	0.4%
Grigoriou, et al 2013	470	1025	0.46	[0.43; 0.49]	0.3%	0.4%
Dennerstein, et al 2000	26	172	0.15	[0.10; 0.21]	0.1%	0.4%
Nappi, et al1 2008	102	300	0.34	[0.29; 0.40]	0.1%	0.4%
Nappi, et al2 2008	75	300	0.25	[0.20; 0.30]	0.1%	0.4%
Nappi, et al3 2008	72	300	0.24	[0.19; 0.29]	0.1%	0.4%
Nappi, et al4 2008	78	301	0.26	[0.21; 0.31]	0.1%	0.4%
Nappi, et al5 2008	63	301	0.21	[0.16; 0.26]	0.1%	0.4%
Nappi, et al6 2008	57	301	0.19	[0.15; 0.24]	0.1%	0.4%
Saied, et al 2021	259	342	0.76	[0.71; 0.80]	0.1%	0.4%
Sheereen, et al 2022	57	100	0.57	[0.47; 0.67]	0.0%	0.4%
Kang, et al 2021	81	150	0.54	[0.46; 0.62]	0.1%	0.4%
Senthilvel, et al 2018	119	150	0.79	[0.72; 0.86]	0.1%	0.4%
Karmakar, et al 2017	88	100	0.88	[0.80; 0.94]	0.0%	0.4%
Ganapathy, et al 2018	96	140	0.69	[0.60; 0.76]	0.0%	0.4%
Tan, et al 2014	202	309	0.65	[0.60; 0.71]	0.1%	0.4%
Zhao, et al 2023	991	1829	0.54	[0.52; 0.56]	0.6%	0.4%
Han, et al 2023	10	66	0.15	[0.08; 0.26]	0.0%	0.4%
Ko?o?lu, et al 2022	267	383	0.70	[0.65; 0.74]	0.1%	0.4%
AlDughaither, et al 2015	35	119	0.29	[0.21; 0.38]	0.0%	0.4%
Ceylan, et al 2014	467	874	0.53	[0.50; 0.57]	0.3%	0.4%
Thakur,et al 2019	207	351	0.59	[0.54; 0.64]	0.1%	0.4%
Kim, et al 2017	302	743	0.41	[0.37; 0.44]	0.2%	0.4%
Gerber, et al 2018	42	139	0.30	[0.23; 0.39]	0.0%	0.4%
Silvestrin, et al 2016	70	110	0.64	[0.54; 0.73]	0.0%	0.4%
Ghazanfarpour, et al 2016	55	202	0.27	[0.21; 0.34]	0.1%	0.4%
Duffy, et al 2012	1131	4264	0.27	[0.25; 0.28]	1.4%	0.4%
Loutfy, et al 2006	273	450	0.61	[0.56; 0.65]	0.1%	0.4%
Delavar, et al 2011	834	1397	0.60	[0.57; 0.62]	0.5%	0.4%
Chedraui, et al 2007	219	325	0.67	[0.62; 0.72]	0.1%	0.4%
Suka, et al 2010	169	1169	0.14	[0.12; 0.17]	0.4%	0.4%
Rahman, et al 2011	190	509	0.37	[0.33; 0.42]	0.2%	0.4%
Agwu, et al 2008	43	186	0.23	[0.17; 0.30]	0.1%	0.4%
Ahmed, et al 2016	122	150	0.81	[0.74; 0.87]	0.1%	0.4%
Bener, et al 2014	302	1158	0.26	[0.24; 0.29]	0.4%	0.4%
Bener, et al 2000	101	390	0.26	[0.22; 0.31]	0.1%	0.4%
Im, et al 2010	199	512	0.39	[0.35; 0.43]	0.2%	0.4%
Ande, et al 2011	23	533	0.04	[0.03; 0.06]	0.2%	0.4%
Khatoon, et al 2018	39	121	0.32	[0.24; 0.41]	0.0%	0.4%
Budako?lu, et al 2007	264	338	0.78	[0.73; 0.82]	0.1%	0.4%
Sharanya Shre, et al 2016	16	150	0.11	[0.06; 0.17]	0.1%	0.4%
Beura, et al 2020	90	104	0.87	[0.78; 0.92]	0.0%	0.4%
Thapa, et al 2020	144	177	0.81	[0.75; 0.87]	0.1%	0.4%
Ibrahim, et al 2015	927	1214	0.76	[0.74; 0.79]	0.4%	0.4%
Nusrat, et al 2008	310	863	0.36	[0.33; 0.39]	0.3%	0.4%
Liu, et al 2007	108	267	0.40	[0.35; 0.47]	0.1%	0.4%
Lee, et al 2020	12209	20882	0.58	[0.58; 0.59]	6.9%	0.4%
Modoodi, et al 2020	281	350	0.80	[0.76; 0.84]	0.1%	0.4%
Kalhan, et al 2020	192	400	0.48	[0.43; 0.53]	0.1%	0.4%
Rindner, et al 2017	72	108	0.67	[0.57; 0.75]	0.0%	0.4%
Al−Musa, et al 2017	185	228	0.81	[0.75; 0.86]	0.1%	0.4%
Bairy, et al 2009	174	352	0.49	[0.44; 0.55]	0.1%	0.4%
Heinemann, et al1 2008	2429	4791	0.51	[0.49; 0.52]	1.6%	0.4%
Heinemann, et al2 2008	833	1500	0.56	[0.53; 0.58]	0.5%	0.4%
Heinemann, et al3 2008	1671	3006	0.56	[0.54; 0.57]	1.0%	0.4%
Heinemann, et al4 2008	397	1000	0.40	[0.37; 0.43]	0.3%	0.4%
Lawton, et al 2008	1045	3616	0.29	[0.27; 0.30]	1.2%	0.4%
Liu, et al 2008	99	217	0.46	[0.39; 0.53]	0.1%	0.4%
Liu, et al 2007	150	188	0.80	[0.73; 0.85]	0.1%	0.4%
Saccomani, et al 2017	255	749	0.34	[0.31; 0.38]	0.2%	0.4%
Resmi, et al 2020	38	400	0.10	[0.07; 0.13]	0.1%	0.4%
Deveci, et al 2010	192	519	0.37	[0.33; 0.41]	0.2%	0.4%
O?urlu, et al 2011	53	132	0.40	[0.32; 0.49]	0.0%	0.4%
Wang, et al 2021	1422	7275	0.20	[0.19; 0.20]	2.4%	0.4%
Anolue, et al 2012	46	349	0.13	[0.10; 0.17]	0.1%	0.4%
Mahajan,et al 2012	40	100	0.40	[0.30; 0.50]	0.0%	0.4%
Mustafa, et al 2012	47	500	0.09	[0.07; 0.12]	0.2%	0.4%
Sassoon, et al 2014	17	63	0.27	[0.17; 0.40]	0.0%	0.4%
Liu, et al 2013	681	1686	0.40	[0.38; 0.43]	0.6%	0.4%
Mohamed, et al 2014	29	90	0.32	[0.23; 0.43]	0.0%	0.4%
Kong, et al 2019	64	120	0.53	[0.44; 0.62]	0.0%	0.4%
Sharifi, et al 2019	594	600	0.99	[0.98; 1.00]	0.2%	0.4%
Pan, et al 2002	32	161	0.20	[0.14; 0.27]	0.1%	0.4%
Kasuga, et al 2004	732	1069	0.68	[0.66; 0.71]	0.4%	0.4%
Kaur, et al 2004	26	245	0.11	[0.07; 0.15]	0.1%	0.4%
Yahya, et al 2002	50	130	0.38	[0.30; 0.47]	0.0%	0.4%
Gupta, et al1 2006	29	103	0.28	[0.20; 0.38]	0.0%	0.4%
Gupta, et al2 2006	7	50	0.14	[0.06; 0.27]	0.0%	0.4%
Can..rio, et al 2012	202	370	0.55	[0.49; 0.60]	0.1%	0.4%
Rahman, et al 2020	268	390	0.69	[0.64; 0.73]	0.1%	0.4%
Abedzadeh−Kalahroudi, et al 2012	579	700	0.83	[0.80; 0.85]	0.2%	0.4%
Diyu, et al 2022	241	318	0.76	[0.71; 0.80]	0.1%	0.4%
Ryu, et al 2020	1548	2481	0.62	[0.60; 0.64]	0.8%	0.4%
Ma, et al 2017	607	2336	0.26	[0.24; 0.28]	0.8%	0.4%
Fu, et al 2020	617	3199	0.19	[0.18; 0.21]	1.1%	0.4%
Liu, et al 2018	957	3382	0.28	[0.27; 0.30]	1.1%	0.4%
Li, et al 2016	276	1062	0.26	[0.23; 0.29]	0.4%	0.4%
Ghimire, et al 2015	489	833	0.59	[0.55; 0.62]	0.3%	0.4%
Joseph, et al 2014	56	110	0.51	[0.41; 0.61]	0.0%	0.4%
Bindhu, et al 2014	125	320	0.39	[0.34; 0.45]	0.1%	0.4%
Xu, et al 2016	159	511	0.31	[0.27; 0.35]	0.2%	0.4%
Sweed, et al 2012	298	400	0.74	[0.70; 0.79]	0.1%	0.4%
Taher, et al 2012	56	86	0.65	[0.54; 0.75]	0.0%	0.4%
Ryan, et al 2009	35	138	0.25	[0.18; 0.33]	0.0%	0.4%
G K, et al 2013	286	500	0.57	[0.53; 0.62]	0.2%	0.4%
Bernis, et al 2007	259	1069	0.24	[0.22; 0.27]	0.4%	0.4%
Freeman, et al 2009	85	170	0.50	[0.42; 0.58]	0.1%	0.4%
Chedraui, et al 2007	224	300	0.75	[0.69; 0.79]	0.1%	0.4%
Ahlawat, et al 2019	241	580	0.42	[0.38; 0.46]	0.2%	0.4%
Bansal, et al 2015	156	180	0.87	[0.81; 0.91]	0.1%	0.4%
Dutta, et al 2018	42	171	0.25	[0.18; 0.32]	0.1%	0.4%
Nayak, et al 2019	65	290	0.22	[0.18; 0.28]	0.1%	0.4%
Ps, et al 2017	260	594	0.44	[0.40; 0.48]	0.2%	0.4%
Alam, et al 2020	299	326	0.92	[0.88; 0.94]	0.1%	0.4%
Fabbrini, et al 2015	54	334	0.16	[0.12; 0.21]	0.1%	0.4%
Hsu, et al 2005	22	197	0.11	[0.07; 0.16]	0.1%	0.4%
Ward, et al 2010	507	1115	0.45	[0.43; 0.48]	0.4%	0.4%
Pandey, et al 2020	104	189	0.55	[0.48; 0.62]	0.1%	0.4%
Liao, et al 2019	232	2051	0.11	[0.10; 0.13]	0.7%	0.4%
Sievert, et al 2007	269	869	0.31	[0.28; 0.34]	0.3%	0.4%
Sievert, et al 2003	395	755	0.52	[0.49; 0.56]	0.3%	0.4%
Obermeyer, et al 2004	94	293	0.32	[0.27; 0.38]	0.1%	0.4%
Keenan, et al 2003	919	2602	0.35	[0.33; 0.37]	0.9%	0.4%
Zhao, et al 2000	214	806	0.27	[0.24; 0.30]	0.3%	0.4%
Gollschewski, et al 2004	401	860	0.47	[0.43; 0.50]	0.3%	0.4%
Biri, et al 2005	441	1049	0.42	[0.39; 0.45]	0.3%	0.4%
Obermeyer, et al 2002	84	299	0.28	[0.23; 0.34]	0.1%	0.4%
Lin, et al 2012	145	3359	0.04	[0.04; 0.05]	1.1%	0.4%
Loh, et al 2005	132	656	0.20	[0.17; 0.23]	0.2%	0.4%
Chaopotong, et al 2005	96	148	0.65	[0.57; 0.73]	0.0%	0.4%
Huang, et al1 2010	105	300	0.35	[0.30; 0.41]	0.1%	0.4%
Huang, et al2 2010	59	100	0.59	[0.49; 0.69]	0.0%	0.4%
Huang, et al3 2010	57	300	0.19	[0.15; 0.24]	0.1%	0.4%
Huang, et al4 2010	67	150	0.45	[0.37; 0.53]	0.1%	0.4%
Huang, et al5 2010	89	150	0.59	[0.51; 0.67]	0.1%	0.4%
Anderson, et al1 2004	401	860	0.47	[0.43; 0.50]	0.3%	0.4%
Anderson, et al2 2004	438	825	0.53	[0.50; 0.57]	0.3%	0.4%
Glod, et al 2006	750	3302	0.23	[0.21; 0.24]	1.1%	0.4%
Afshari, et al 2015	766	1280	0.60	[0.57; 0.63]	0.4%	0.4%
Goncalves, et al 2013	266	579	0.46	[0.42; 0.50]	0.2%	0.4%
Onya, et al 2018	145	380	0.38	[0.33; 0.43]	0.1%	0.4%
Yen, et al 2009	182	672	0.27	[0.24; 0.31]	0.2%	0.4%
Timur, et al 2010	286	685	0.42	[0.38; 0.46]	0.2%	0.4%
Zang, et al 2016	84	743	0.11	[0.09; 0.14]	0.2%	0.4%
Masjoudl, et al 2017	282	645	0.44	[0.40; 0.48]	0.2%	0.4%
Kapoor, et al 2021	604	1670	0.36	[0.34; 0.39]	0.6%	0.4%
Durairaj, et al 2022	48	74	0.65	[0.53; 0.76]	0.0%	0.4%
Bala, et al 2022	182	245	0.74	[0.68; 0.80]	0.1%	0.4%
Abou−Raya, et al 2016	360	540	0.67	[0.63; 0.71]	0.2%	0.4%
Krajewska−Ferishah, et al1 2022	92	241	0.38	[0.32; 0.45]	0.1%	0.4%
Krajewska−Ferishah, et al2 2022	57	119	0.48	[0.39; 0.57]	0.0%	0.4%
Krajewska−Ferishah, et al3 2022	32	79	0.41	[0.30; 0.52]	0.0%	0.4%
Krajewska−Ferishah, et al4 2022	45	100	0.45	[0.35; 0.55]	0.0%	0.4%
Ogwumike, et al 2012	54	547	0.10	[0.08; 0.13]	0.2%	0.4%
Wang, et al 2013	219	566	0.39	[0.35; 0.43]	0.2%	0.4%
Sierra, et al 2004	316	385	0.82	[0.78; 0.86]	0.1%	0.4%
Castelo−Branco, et al 2005	13	125	0.10	[0.06; 0.17]	0.0%	0.4%
Jin, et al 2014	257	1215	0.21	[0.19; 0.24]	0.4%	0.4%
Dasgupta, et al 2012	403	480	0.84	[0.80; 0.87]	0.2%	0.4%
Legorreta, et al 2013	2465	4548	0.54	[0.53; 0.56]	1.5%	0.4%
Shea, et al 2020	2436	13216	0.18	[0.18; 0.19]	4.4%	0.4%
Abdullah, et al 2016	115	258	0.45	[0.38; 0.51]	0.1%	0.4%
Chu, et al 2022	827	1748	0.47	[0.45; 0.50]	0.6%	0.4%
Barghandan, et al 2021	219	245	0.89	[0.85; 0.93]	0.1%	0.4%
Hickey, et al 2017	558	1149	0.49	[0.46; 0.51]	0.4%	0.4%
Blell, et al 2014	90	253	0.36	[0.30; 0.42]	0.1%	0.4%
Dienye, et al 2013	192	385	0.50	[0.45; 0.55]	0.1%	0.4%
Whiteley, et al 2013	1400	8811	0.16	[0.15; 0.17]	2.9%	0.4%
Bener, et al 2017	343	1101	0.31	[0.28; 0.34]	0.4%	0.4%
Olowokere, et al 2020	212	271	0.78	[0.73; 0.83]	0.1%	0.4%
Salazar−Pousada, et al 2017	311	864	0.36	[0.33; 0.39]	0.3%	0.4%
Ruan, et al 2017	758	1225	0.62	[0.59; 0.65]	0.4%	0.4%
An, et al 2022	4691	45177	0.10	[0.10; 0.11]	15.0%	0.4%
Devi, et al 2005	40	151	0.26	[0.20; 0.34]	0.1%	0.4%
Li, et al 2019	665	5537	0.12	[0.11; 0.13]	1.8%	0.4%
Xu, et al 2005	98	237	0.41	[0.35; 0.48]	0.1%	0.4%
Y_lmaz, et al 2020	48	190	0.25	[0.19; 0.32]	0.1%	0.4%
Shakhatreh, et al 2006	57	143	0.40	[0.32; 0.48]	0.0%	0.4%
Obermeyer, et al 2007	111	271	0.41	[0.35; 0.47]	0.1%	0.4%
Chae, et al 2014	233	570	0.41	[0.37; 0.45]	0.2%	0.4%
Lampio, et al 2014	32	158	0.20	[0.14; 0.27]	0.1%	0.4%
Oppermann, et al 2012	82	211	0.39	[0.32; 0.46]	0.1%	0.4%
Kamal, et al 2017	334	1100	0.30	[0.28; 0.33]	0.4%	0.4%
Rajbhandari, et al 2017	146	2000	0.07	[0.06; 0.09]	0.7%	0.4%
Cortes, et al 2023	125	358	0.35	[0.30; 0.40]	0.1%	0.4%
Chalise, et al 2022	7	180	0.04	[0.02; 0.08]	0.1%	0.4%
**Common effect model**		**300449**	**0.31**	**[0.31; 0.31]**	**100.0%**	**--**
**Random effects model**			**0.43**	**[0.40; 0.46]**	**--**	**100.0%**

Heterogeneity: $I^2 = 100\%$, $\tau^2 = 0.0553$, $p = 0$

Supplementary Figure 7. Forest plot of prevalence of physical and mental exhaustion among middle-aged women

Study	Events	Total	Proportion	95%−CI	Weight (common)	Weight (random)
Lan, et al 2017	454	985	0.46	[0.43; 0.49]	0.5%	0.6%
Adhikari, et al 2022	196	252	0.78	[0.72; 0.83]	0.1%	0.6%
Wang, et al 2021	2569	6364	0.40	[0.39; 0.42]	3.2%	0.6%
Olaolorun, et al 2009	511	1189	0.43	[0.40; 0.46]	0.6%	0.6%
Li, et al 2012	8281	20275	0.41	[0.40; 0.42]	10.2%	0.6%
Javadivala, et al 2020	129	161	0.80	[0.73; 0.86]	0.1%	0.6%
Nagaraj, et al 2021	73	200	0.36	[0.30; 0.44]	0.1%	0.6%
Yisma, et al 2017	79	226	0.35	[0.29; 0.42]	0.1%	0.6%
Korkmaz, et al 2022	99	108	0.92	[0.85; 0.96]	0.1%	0.5%
AlQuaiz, et al 2013	393	490	0.80	[0.76; 0.84]	0.2%	0.6%
Aaron, et al 2002	73	200	0.36	[0.30; 0.44]	0.1%	0.6%
Abdel−Salam, et al 2021	702	845	0.83	[0.80; 0.86]	0.4%	0.6%
Shafie, et al 2011	226	472	0.48	[0.43; 0.52]	0.2%	0.6%
Aloufi, et al 2022	290	361	0.80	[0.76; 0.84]	0.2%	0.6%
Chou, et al 2014	399	442	0.90	[0.87; 0.93]	0.2%	0.6%
Fuh, et al 2003	441	1360	0.32	[0.30; 0.35]	0.7%	0.6%
Chedraui, et al 2014	147	204	0.72	[0.65; 0.78]	0.1%	0.6%
Chedraui, et al 2010	288	404	0.71	[0.67; 0.76]	0.2%	0.6%
Ojeda, et al 2014	565	771	0.73	[0.70; 0.76]	0.4%	0.6%
Ishizuka, et al 2008	756	1169	0.65	[0.62; 0.67]	0.6%	0.6%
Kapur, et al 2009	66	129	0.51	[0.42; 0.60]	0.1%	0.6%
Sharma, et al 2021	175	203	0.86	[0.81; 0.91]	0.1%	0.6%
Qazi, et al 2006	542	800	0.68	[0.64; 0.71]	0.4%	0.6%
Rahman, et al 2010	239	356	0.67	[0.62; 0.72]	0.2%	0.6%
Shea, et al 2006	143	399	0.36	[0.31; 0.41]	0.2%	0.6%
Som, et al 2012	238	250	0.95	[0.92; 0.97]	0.1%	0.6%
Alwi, et al 2009	209	276	0.76	[0.70; 0.81]	0.1%	0.6%
Waidyasekera, et al 2009	368	683	0.54	[0.50; 0.58]	0.3%	0.6%
Silva, et al 2013	886	1415	0.63	[0.60; 0.65]	0.7%	0.6%
Mart..nez, et al 2013	3187	10514	0.30	[0.29; 0.31]	5.3%	0.6%
Yang, et al 2008	2587	9617	0.27	[0.26; 0.28]	4.8%	0.6%
Yim, et al 2015	1461	1774	0.82	[0.81; 0.84]	0.9%	0.6%
Du, et al 2020	1196	3141	0.38	[0.36; 0.40]	1.6%	0.6%
Buhling, et al 2014	1183	1893	0.62	[0.60; 0.65]	1.0%	0.6%
Singh, et al 2014	101	252	0.40	[0.34; 0.46]	0.1%	0.6%
Kulkarni, et al 2016	84	100	0.84	[0.75; 0.91]	0.1%	0.5%
Rathnayake, et al 2019	223	350	0.64	[0.58; 0.69]	0.2%	0.6%
Baquedano, et al 2023	487	1059	0.46	[0.43; 0.49]	0.5%	0.6%
Pateliya, et al 2022	81	100	0.81	[0.72; 0.88]	0.1%	0.5%
Srinivasan, et al 2021	65	100	0.65	[0.55; 0.74]	0.1%	0.5%
Chim, et al 2002	192	495	0.39	[0.34; 0.43]	0.2%	0.6%
Dhillon,et al 2006	258	326	0.79	[0.74; 0.83]	0.2%	0.6%
Chuni, et al 2011	536	729	0.74	[0.70; 0.77]	0.4%	0.6%
Nisar, et al 2015	2620	3062	0.86	[0.84; 0.87]	1.5%	0.6%
Zhang, et al 2016	62	106	0.58	[0.49; 0.68]	0.1%	0.5%
Agaba, et al 2017	76	107	0.71	[0.61; 0.79]	0.1%	0.5%
AlQuaiz, et al 2017	187	228	0.82	[0.76; 0.87]	0.1%	0.6%
Grigoriou, et al 2013	508	1025	0.50	[0.46; 0.53]	0.5%	0.6%
G..recka, et al 2022	268	349	0.77	[0.72; 0.81]	0.2%	0.6%
Saied, et al 2021	321	342	0.94	[0.91; 0.96]	0.2%	0.6%
Sheereen, et al 2022	92	100	0.92	[0.85; 0.96]	0.1%	0.5%
Kang, et al 2021	131	150	0.87	[0.81; 0.92]	0.1%	0.6%
Senthilvel, et al 2018	119	150	0.79	[0.72; 0.86]	0.1%	0.6%
Karmakar, et al 2017	93	100	0.93	[0.86; 0.97]	0.1%	0.5%
Ganapathy, et al 2018	125	140	0.89	[0.83; 0.94]	0.1%	0.6%
Tan, et al 2014	232	309	0.75	[0.70; 0.80]	0.2%	0.6%
Chopra, et al 2022	418	504	0.83	[0.79; 0.86]	0.3%	0.6%
Zhao, et al 2023	1483	1829	0.81	[0.79; 0.83]	0.9%	0.6%
Han, et al 2023	42	66	0.64	[0.51; 0.75]	0.0%	0.5%
Ko?o?lu, et al 2022	297	383	0.78	[0.73; 0.82]	0.2%	0.6%
AlDughaither, et al 2015	77	119	0.65	[0.55; 0.73]	0.1%	0.5%
Ceylan, et al 2014	692	874	0.79	[0.76; 0.82]	0.4%	0.6%
Thakur,et al 2019	242	351	0.69	[0.64; 0.74]	0.2%	0.6%
Kim, et al 2017	374	743	0.50	[0.47; 0.54]	0.4%	0.6%
Gerber, et al 2018	66	139	0.47	[0.39; 0.56]	0.1%	0.6%
Ghazanfarpour, et al 2016	61	202	0.30	[0.24; 0.37]	0.1%	0.6%
Duffy, et al 2012	3624	4309	0.84	[0.83; 0.85]	2.2%	0.6%
Loutfy, et al 2006	432	450	0.96	[0.94; 0.98]	0.2%	0.6%
Delavar, et al 2011	374	1397	0.27	[0.24; 0.29]	0.7%	0.6%
Chedraui, et al 2007	258	325	0.79	[0.75; 0.84]	0.2%	0.6%
Chedraui, et al 2014	299	451	0.66	[0.62; 0.71]	0.2%	0.6%
Suka, et al 2010	409	1169	0.35	[0.32; 0.38]	0.6%	0.6%
Rahman, et al 2011	310	509	0.61	[0.57; 0.65]	0.3%	0.6%
Ahmed, et al 2016	124	150	0.83	[0.76; 0.88]	0.1%	0.6%
Bener, et al 2014	311	1158	0.27	[0.24; 0.30]	0.6%	0.6%
Bener, et al 2000	104	390	0.27	[0.22; 0.31]	0.2%	0.6%
Im, et al 2010	275	512	0.54	[0.49; 0.58]	0.3%	0.6%
Ande, et al 2011	104	533	0.20	[0.16; 0.23]	0.3%	0.6%
Khatoon, et al 2018	88	121	0.73	[0.64; 0.80]	0.1%	0.5%
Budako?lu, et al 2007	183	338	0.54	[0.49; 0.60]	0.2%	0.6%
Sharanya Shre, et al 2016	61	150	0.41	[0.33; 0.49]	0.1%	0.6%
Damodaran, et al 2000	125	164	0.76	[0.69; 0.83]	0.1%	0.6%
Beura, et al 2020	96	104	0.92	[0.85; 0.97]	0.1%	0.5%
Thapa, et al 2020	156	177	0.88	[0.82; 0.93]	0.1%	0.6%
Ibrahim, et al 2015	1032	1214	0.85	[0.83; 0.87]	0.6%	0.6%
Liu, et al 2007	179	267	0.67	[0.61; 0.73]	0.1%	0.6%
Lee, et al 2020	13880	20882	0.66	[0.66; 0.67]	10.5%	0.6%
Modoodi, et al 2020	261	350	0.75	[0.70; 0.79]	0.2%	0.6%
Kalhan, et al 2020	286	400	0.71	[0.67; 0.76]	0.2%	0.6%
Rindner, et al 2017	80	109	0.73	[0.64; 0.81]	0.1%	0.5%
Al−Musa, et al 2017	187	228	0.82	[0.76; 0.87]	0.1%	0.6%
Moilanen, et al 2010	60	1184	0.05	[0.04; 0.06]	0.6%	0.6%
Bairy, et al 2009	155	352	0.44	[0.39; 0.49]	0.2%	0.6%
Lawton, et al 2008	2412	3616	0.67	[0.65; 0.68]	1.8%	0.6%
Liu, et al 2008	144	217	0.66	[0.60; 0.73]	0.1%	0.6%
Liu, et al 2007	160	188	0.85	[0.79; 0.90]	0.1%	0.6%
Resmi, et al 2020	129	400	0.32	[0.28; 0.37]	0.2%	0.6%
Ashrafi, et al 2009	1378	2462	0.56	[0.54; 0.58]	1.2%	0.6%
Deveci, et al 2010	349	519	0.67	[0.63; 0.71]	0.3%	0.6%
O?urlu, et al 2011	63	132	0.48	[0.39; 0.57]	0.1%	0.6%
Anolue, et al 2012	93	349	0.27	[0.22; 0.32]	0.2%	0.6%
Mahajan,et al 2012	62	100	0.62	[0.52; 0.72]	0.1%	0.5%
Mustafa, et al 2012	416	500	0.83	[0.80; 0.86]	0.3%	0.6%
Nappi, et al 2012	1619	3520	0.46	[0.44; 0.48]	1.8%	0.6%
Sassoon, et al 2014	48	63	0.76	[0.64; 0.86]	0.0%	0.5%
Liu, et al 2013	1378	1686	0.82	[0.80; 0.84]	0.8%	0.6%
Mohamed, et al 2014	72	90	0.80	[0.70; 0.88]	0.0%	0.5%
Kong, et al 2019	111	120	0.92	[0.86; 0.97]	0.1%	0.5%
Holm, et al 2000	160	214	0.75	[0.68; 0.80]	0.1%	0.6%
Pan, et al 2002	42	161	0.26	[0.19; 0.34]	0.1%	0.6%
Kasuga, et al 2004	943	1069	0.88	[0.86; 0.90]	0.5%	0.6%
Kaur, et al 2004	100	245	0.41	[0.35; 0.47]	0.1%	0.6%
Gupta, et al1 2006	54	103	0.52	[0.42; 0.62]	0.1%	0.5%
Gupta, et al2 2006	9	50	0.18	[0.09; 0.31]	0.0%	0.5%
Mathialagan, et al 2022	110	321	0.34	[0.29; 0.40]	0.2%	0.6%
Can..rio, et al 2012	241	370	0.65	[0.60; 0.70]	0.2%	0.6%
Rahman, et al 2020	291	390	0.75	[0.70; 0.79]	0.2%	0.6%
Abedzadeh−Kalahroudi, et al 2012	647	700	0.92	[0.90; 0.94]	0.4%	0.6%
Diyu, et al 2022	275	318	0.86	[0.82; 0.90]	0.2%	0.6%
Zhang, et al 2016	1131	2308	0.49	[0.47; 0.51]	1.2%	0.6%
Ryu, et al 2020	2147	2481	0.87	[0.85; 0.88]	1.2%	0.6%
Ma, et al 2017	1284	2336	0.55	[0.53; 0.57]	1.2%	0.6%
Fu, et al 2020	1366	3199	0.43	[0.41; 0.44]	1.6%	0.6%
Liu, et al 2018	2409	3382	0.71	[0.70; 0.73]	1.7%	0.6%
Li, et al 2016	576	1062	0.54	[0.51; 0.57]	0.5%	0.6%
Joshi, et al 2015	668	1000	0.67	[0.64; 0.70]	0.5%	0.6%
Ghimire, et al 2015	673	833	0.81	[0.78; 0.83]	0.4%	0.6%
Joseph, et al 2014	94	110	0.85	[0.77; 0.91]	0.1%	0.5%
Bindhu, et al 2014	159	320	0.50	[0.44; 0.55]	0.2%	0.6%
Srivastava, et al 2014	85	117	0.73	[0.64; 0.80]	0.1%	0.5%
Sweed, et al 2012	320	400	0.80	[0.76; 0.84]	0.2%	0.6%
Taher, et al 2012	48	86	0.56	[0.45; 0.67]	0.0%	0.5%
G K, et al 2013	360	500	0.72	[0.68; 0.76]	0.3%	0.6%
Bernis, et al 2007	300	1069	0.28	[0.25; 0.31]	0.5%	0.6%
Chedraui, et al 2007	120	300	0.40	[0.34; 0.46]	0.2%	0.6%
Nayak, et al 2019	230	290	0.79	[0.74; 0.84]	0.1%	0.6%
Hsu, et al 2005	35	197	0.18	[0.13; 0.24]	0.1%	0.6%
Ward, et al 2010	720	1115	0.65	[0.62; 0.67]	0.6%	0.6%
Pandey, et al 2020	97	189	0.51	[0.44; 0.59]	0.1%	0.6%
Sievert, et al 2007	417	869	0.48	[0.45; 0.51]	0.4%	0.6%
Sievert, et al 2003	525	755	0.70	[0.66; 0.73]	0.4%	0.6%
Obermeyer, et al 2004	132	293	0.45	[0.39; 0.51]	0.1%	0.6%
Zhao, et al 2000	376	806	0.47	[0.43; 0.50]	0.4%	0.6%
Lam, et al 2003	528	978	0.54	[0.51; 0.57]	0.5%	0.6%
Gollschewski, et al 2004	710	863	0.82	[0.80; 0.85]	0.4%	0.6%
Biri, et al 2005	617	1049	0.59	[0.56; 0.62]	0.5%	0.6%
Obermeyer, et al 2002	185	299	0.62	[0.56; 0.67]	0.2%	0.6%
Chaopotong, et al 2005	101	148	0.68	[0.60; 0.76]	0.1%	0.6%
Anderson, et al1 2004	710	863	0.82	[0.80; 0.85]	0.4%	0.6%
Anderson, et al2 2004	704	827	0.85	[0.83; 0.87]	0.4%	0.6%
Masjoudl, et al 2017	453	645	0.70	[0.67; 0.74]	0.3%	0.6%
Durairaj, et al 2022	51	74	0.69	[0.57; 0.79]	0.0%	0.5%
Bala, et al 2022	214	245	0.87	[0.83; 0.91]	0.1%	0.6%
Abou−Raya, et al 2016	416	540	0.77	[0.73; 0.81]	0.3%	0.6%
Sierra, et al 2004	297	385	0.77	[0.73; 0.81]	0.2%	0.6%
Jin, et al 2014	609	1215	0.50	[0.47; 0.53]	0.6%	0.6%
Pe_rez−L.pez, et al 2013	933	1739	0.54	[0.51; 0.56]	0.9%	0.6%
Huang, et al 2020	984	1501	0.66	[0.63; 0.68]	0.8%	0.6%
Dasgupta, et al 2012	371	480	0.77	[0.73; 0.81]	0.2%	0.6%
Legorreta, et al 2013	2774	4548	0.61	[0.60; 0.62]	2.3%	0.6%
Wu, et al 2023	319	468	0.68	[0.64; 0.72]	0.2%	0.6%
Abdullah, et al 2016	153	258	0.59	[0.53; 0.65]	0.1%	0.6%
Chu, et al 2022	1143	1748	0.65	[0.63; 0.68]	0.9%	0.6%
Hickey, et al 2017	653	1143	0.57	[0.54; 0.60]	0.6%	0.6%
Blell, et al 2014	105	254	0.41	[0.35; 0.48]	0.1%	0.6%
Dienye, et al 2013	309	385	0.80	[0.76; 0.84]	0.2%	0.6%
Olowokere, et al 2020	214	271	0.79	[0.74; 0.84]	0.1%	0.6%
Zhu, et al 2021	1073	2400	0.45	[0.43; 0.47]	1.2%	0.6%
Ruan, et al 2017	929	1225	0.76	[0.73; 0.78]	0.6%	0.6%
Xu, et al 2005	83	237	0.35	[0.29; 0.41]	0.1%	0.6%
Obermeyer, et al 2007	214	271	0.79	[0.74; 0.84]	0.1%	0.6%
Veigh, et al 2005	144	160	0.90	[0.84; 0.94]	0.1%	0.6%
Oppermann, et al 2012	160	211	0.76	[0.69; 0.81]	0.1%	0.6%
An, et al 2023	5045	7309	0.69	[0.68; 0.70]	3.7%	0.6%
Kamal, et al 2017	766	1100	0.70	[0.67; 0.72]	0.6%	0.6%
Rajbhandari, et al 2017	498	2000	0.25	[0.23; 0.27]	1.0%	0.6%
Cortes, et al 2023	133	358	0.37	[0.32; 0.42]	0.2%	0.6%
Chalise, et al 2022	17	180	0.09	[0.06; 0.15]	0.1%	0.6%
**Common effect model**		**198507**	**0.57**	**[0.56; 0.57]**	**100.0%**	**--**
**Random effects model**			**0.64**	**[0.61; 0.67]**	**--**	**100.0%**

Heterogeneity: $I^2 = 99\%$, $\tau^2 = 0.0494$, $p = 0$

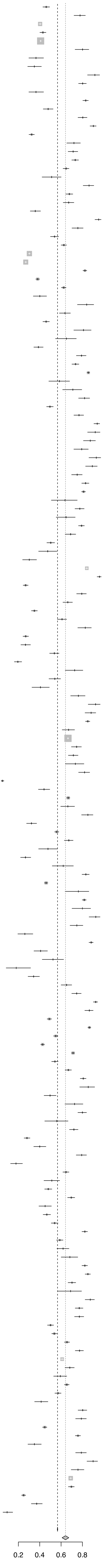

Supplementary Figure 8. Forest plot of prevalence of irritability among middle-aged women

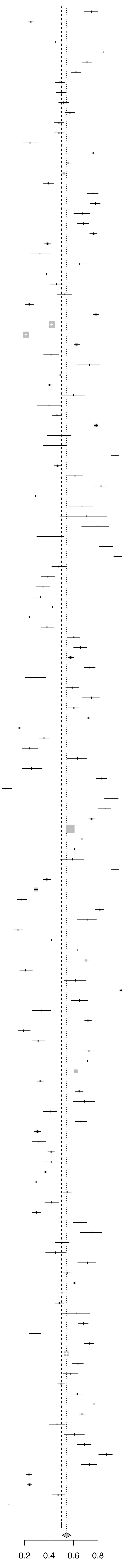

Study	Events	Total	Proportion	95%−CI	Weight (common)	Weight (random)
Adhikari, et al 2022	188	252	0.75	[0.69; 0.80]	0.2%	0.7%
Olaolorun, et al 2009	298	1189	0.25	[0.23; 0.28]	1.0%	0.7%
Javadivala, et al 2020	87	161	0.54	[0.46; 0.62]	0.1%	0.7%
Yisma, et al 2017	102	226	0.45	[0.39; 0.52]	0.2%	0.7%
Korkmaz, et al 2022	91	108	0.84	[0.76; 0.91]	0.1%	0.7%
AlQuaiz, et al 2013	348	490	0.71	[0.67; 0.75]	0.4%	0.7%
Genazzani, et al1 2006	379	611	0.62	[0.58; 0.66]	0.5%	0.7%
Genazzani, et al2 2006	294	600	0.49	[0.45; 0.53]	0.5%	0.7%
Genazzani, et al3 2006	298	596	0.50	[0.46; 0.54]	0.5%	0.7%
Genazzani, et al4 2006	309	594	0.52	[0.48; 0.56]	0.5%	0.7%
Genazzani, et al5 2006	342	600	0.57	[0.53; 0.61]	0.5%	0.7%
Genazzani, et al6 2006	288	600	0.48	[0.44; 0.52]	0.5%	0.7%
Genazzani, et al7 2006	288	600	0.48	[0.44; 0.52]	0.5%	0.7%
Aaron, et al 2002	49	200	0.24	[0.19; 0.31]	0.2%	0.7%
Abdel−Salam, et al 2021	644	845	0.76	[0.73; 0.79]	0.7%	0.7%
Anderson, et al1 2004	396	712	0.56	[0.52; 0.59]	0.6%	0.7%
Anderson, et al2 2004	746	1430	0.52	[0.50; 0.55]	1.2%	0.7%
Shafie, et al 2011	186	472	0.39	[0.35; 0.44]	0.4%	0.7%
Aloufi, et al 2022	274	361	0.76	[0.71; 0.80]	0.3%	0.7%
Chou, et al 2014	345	442	0.78	[0.74; 0.82]	0.4%	0.7%
Chedraui, et al 2014	137	204	0.67	[0.60; 0.74]	0.2%	0.7%
Chedraui, et al 2010	275	404	0.68	[0.63; 0.73]	0.3%	0.7%
Ojeda, et al 2014	589	771	0.76	[0.73; 0.79]	0.6%	0.7%
Ishizuka, et al 2008	453	1169	0.39	[0.36; 0.42]	1.0%	0.7%
Kapur, et al 2009	42	129	0.33	[0.25; 0.41]	0.1%	0.7%
Sharma, et al 2021	132	203	0.65	[0.58; 0.72]	0.2%	0.7%
Rahman, et al 2010	135	356	0.38	[0.33; 0.43]	0.3%	0.7%
Shea, et al 2006	184	399	0.46	[0.41; 0.51]	0.3%	0.7%
Alwi, et al 2009	146	276	0.53	[0.47; 0.59]	0.2%	0.7%
Waidyasekera, et al 2009	163	683	0.24	[0.21; 0.27]	0.6%	0.7%
Silva, et al 2013	1108	1415	0.78	[0.76; 0.80]	1.2%	0.7%
Mart..nez, et al 2013	4440	10514	0.42	[0.41; 0.43]	8.6%	0.7%
Yang, et al 2008	2020	9617	0.21	[0.20; 0.22]	7.9%	0.7%
Buhling, et al 2014	1187	1893	0.63	[0.60; 0.65]	1.5%	0.7%
Singh, et al 2014	105	252	0.42	[0.36; 0.48]	0.2%	0.7%
Kulkarni, et al 2016	73	100	0.73	[0.63; 0.81]	0.1%	0.7%
Rathnayake, et al 2019	172	350	0.49	[0.44; 0.55]	0.3%	0.7%
Baquedano, et al 2023	428	1059	0.40	[0.37; 0.43]	0.9%	0.7%
Pateliya, et al 2022	60	100	0.60	[0.50; 0.70]	0.1%	0.7%
Srinivasan, et al 2021	40	100	0.40	[0.30; 0.50]	0.1%	0.7%
Chuni, et al 2011	339	729	0.47	[0.43; 0.50]	0.6%	0.7%
Nisar, et al 2015	2408	3062	0.79	[0.77; 0.80]	2.5%	0.7%
Zhang, et al 2016	51	106	0.48	[0.38; 0.58]	0.1%	0.7%
Agaba, et al 2017	48	107	0.45	[0.35; 0.55]	0.1%	0.7%
AlQuaiz, et al 2017	216	228	0.95	[0.91; 0.97]	0.2%	0.7%
Grigoriou, et al 2013	482	1025	0.47	[0.44; 0.50]	0.8%	0.7%
Haines, et al1 2005	152	248	0.61	[0.55; 0.67]	0.2%	0.7%
Haines, et al2 2005	161	195	0.83	[0.76; 0.88]	0.2%	0.7%
Haines, et al3 2005	17	59	0.29	[0.18; 0.42]	0.0%	0.6%
Haines, et al4 2005	65	97	0.67	[0.57; 0.76]	0.1%	0.7%
Haines, et al5 2005	17	24	0.71	[0.49; 0.87]	0.0%	0.6%
Haines, et al6 2005	46	58	0.79	[0.67; 0.89]	0.0%	0.6%
Haines, et al7 2005	33	81	0.41	[0.30; 0.52]	0.1%	0.6%
Haines, et al8 2005	131	150	0.87	[0.81; 0.92]	0.1%	0.7%
Haines, et al9 2005	98	100	0.98	[0.93; 1.00]	0.1%	0.7%
Nappi, et al1 2008	144	300	0.48	[0.42; 0.54]	0.2%	0.7%
Nappi, et al2 2008	117	300	0.39	[0.33; 0.45]	0.2%	0.7%
Nappi, et al3 2008	105	300	0.35	[0.30; 0.41]	0.2%	0.7%
Nappi, et al4 2008	99	301	0.33	[0.28; 0.39]	0.2%	0.7%
Nappi, et al5 2008	129	301	0.43	[0.37; 0.49]	0.2%	0.7%
Nappi, et al6 2008	72	301	0.24	[0.19; 0.29]	0.2%	0.7%
G..recka, et al 2022	134	349	0.38	[0.33; 0.44]	0.3%	0.7%
Saied, et al 2021	206	342	0.60	[0.55; 0.65]	0.3%	0.7%
Tan, et al 2014	203	309	0.66	[0.60; 0.71]	0.3%	0.7%
Zhao, et al 2023	1055	1829	0.58	[0.55; 0.60]	1.5%	0.7%
Ko?o?lu, et al 2022	281	383	0.73	[0.69; 0.78]	0.3%	0.7%
AlDughaither, et al 2015	34	119	0.29	[0.21; 0.38]	0.1%	0.7%
Thakur,et al 2019	207	351	0.59	[0.54; 0.64]	0.3%	0.7%
Sievert, et al 2022	121	162	0.75	[0.67; 0.81]	0.1%	0.7%
Loutfy, et al 2006	271	450	0.60	[0.56; 0.65]	0.4%	0.7%
Delavar, et al 2011	1007	1397	0.72	[0.70; 0.74]	1.1%	0.7%
Suka, et al 2010	183	1169	0.16	[0.14; 0.18]	1.0%	0.7%
Rahman, et al 2011	183	509	0.36	[0.32; 0.40]	0.4%	0.7%
Agwu, et al 2008	45	186	0.24	[0.18; 0.31]	0.2%	0.7%
Ahmed, et al 2016	95	150	0.63	[0.55; 0.71]	0.1%	0.7%
Khatoon, et al 2018	31	121	0.26	[0.18; 0.34]	0.1%	0.7%
Budako?lu, et al 2007	281	338	0.83	[0.79; 0.87]	0.3%	0.7%
Sharanya Shre, et al 2016	7	150	0.05	[0.02; 0.09]	0.1%	0.7%
Beura, et al 2020	96	104	0.92	[0.85; 0.97]	0.1%	0.7%
Thapa, et al 2020	152	177	0.86	[0.80; 0.91]	0.1%	0.7%
Ibrahim, et al 2015	909	1214	0.75	[0.72; 0.77]	1.0%	0.7%
Lee, et al 2020	11963	20882	0.57	[0.57; 0.58]	17.1%	0.7%
Modoodi, et al 2020	234	350	0.67	[0.62; 0.72]	0.3%	0.7%
Kalhan, et al 2020	243	400	0.61	[0.56; 0.66]	0.3%	0.7%
Rindner, et al 2017	64	108	0.59	[0.49; 0.69]	0.1%	0.7%
Al−Musa, et al 2017	216	228	0.95	[0.91; 0.97]	0.2%	0.7%
Uncu, et al 2007	384	1007	0.38	[0.35; 0.41]	0.8%	0.7%
Lawton, et al 2008	1060	3616	0.29	[0.28; 0.31]	3.0%	0.7%
Resmi, et al 2020	71	400	0.18	[0.14; 0.22]	0.3%	0.7%
Deveci, et al 2010	423	519	0.82	[0.78; 0.85]	0.4%	0.7%
O?urlu, et al 2011	94	132	0.71	[0.63; 0.79]	0.1%	0.7%
Anolue, et al 2012	51	349	0.15	[0.11; 0.19]	0.3%	0.7%
Mahajan,et al 2012	42	100	0.42	[0.32; 0.52]	0.1%	0.7%
Sassoon, et al 2014	40	63	0.63	[0.50; 0.75]	0.1%	0.6%
Liu, et al 2013	1184	1686	0.70	[0.68; 0.72]	1.4%	0.7%
Hestiantoro, et al 2019	51	245	0.21	[0.16; 0.26]	0.2%	0.7%
Kong, et al 2019	74	120	0.62	[0.52; 0.70]	0.1%	0.7%
Sharifi, et al 2019	594	600	0.99	[0.98; 1.00]	0.5%	0.7%
Holm, et al 2000	139	214	0.65	[0.58; 0.71]	0.2%	0.7%
Pan, et al 2002	54	161	0.34	[0.26; 0.41]	0.1%	0.7%
Kasuga, et al 2004	769	1069	0.72	[0.69; 0.75]	0.9%	0.7%
Kaur, et al 2004	47	245	0.19	[0.14; 0.25]	0.2%	0.7%
Inayat, et al 2017	96	308	0.31	[0.26; 0.37]	0.3%	0.7%
Rahman, et al 2020	283	390	0.73	[0.68; 0.77]	0.3%	0.7%
Diyu, et al 2022	227	318	0.71	[0.66; 0.76]	0.3%	0.7%
Ryu, et al 2020	1538	2481	0.62	[0.60; 0.64]	2.0%	0.7%
Joshi, et al 2015	328	1000	0.33	[0.30; 0.36]	0.8%	0.7%
Ghimire, et al 2015	539	833	0.65	[0.61; 0.68]	0.7%	0.7%
Joseph, et al 2014	76	110	0.69	[0.60; 0.78]	0.1%	0.7%
Sievert, et al 2008	131	320	0.41	[0.35; 0.47]	0.3%	0.7%
Sweed, et al 2012	264	400	0.66	[0.61; 0.71]	0.3%	0.7%
Bernis, et al 2007	326	1069	0.30	[0.28; 0.33]	0.9%	0.7%
Chedraui, et al 2007	95	300	0.32	[0.26; 0.37]	0.2%	0.7%
Ward, et al 2010	466	1115	0.42	[0.39; 0.45]	0.9%	0.7%
Pandey, et al 2020	79	189	0.42	[0.35; 0.49]	0.2%	0.7%
Sievert, et al 2007	322	869	0.37	[0.34; 0.40]	0.7%	0.7%
Zhao, et al 2000	238	806	0.30	[0.26; 0.33]	0.7%	0.7%
Gollschewski, et al 2004	467	851	0.55	[0.51; 0.58]	0.7%	0.7%
Obermeyer, et al 2002	126	299	0.42	[0.36; 0.48]	0.2%	0.7%
Loh, et al 2005	195	656	0.30	[0.26; 0.33]	0.5%	0.7%
Huang, et al1 2010	196	300	0.65	[0.60; 0.71]	0.2%	0.7%
Huang, et al2 2010	75	100	0.75	[0.65; 0.83]	0.1%	0.7%
Huang, et al3 2010	152	300	0.51	[0.45; 0.56]	0.2%	0.7%
Huang, et al4 2010	68	150	0.45	[0.37; 0.54]	0.1%	0.7%
Huang, et al5 2010	107	150	0.71	[0.63; 0.78]	0.1%	0.7%
Anderson, et al1 2004	467	851	0.55	[0.51; 0.58]	0.7%	0.7%
Anderson, et al2 2004	504	829	0.61	[0.57; 0.64]	0.7%	0.7%
Fagulha, et al 2011	368	728	0.51	[0.47; 0.54]	0.6%	0.7%
Masjoudl, et al 2017	313	645	0.49	[0.45; 0.52]	0.5%	0.7%
Durairaj, et al 2022	46	74	0.62	[0.50; 0.73]	0.1%	0.6%
Abou−Raya, et al 2016	368	540	0.68	[0.64; 0.72]	0.4%	0.7%
Sierra, et al 2004	110	385	0.29	[0.24; 0.33]	0.3%	0.7%
Dasgupta, et al 2012	350	480	0.73	[0.69; 0.77]	0.4%	0.7%
Legorreta, et al 2013	2465	4548	0.54	[0.53; 0.56]	3.7%	0.7%
Wu, et al 2023	298	468	0.64	[0.59; 0.68]	0.4%	0.7%
Abdullah, et al 2016	149	258	0.58	[0.51; 0.64]	0.2%	0.7%
Hickey, et al 2017	572	1146	0.50	[0.47; 0.53]	0.9%	0.7%
Dienye, et al 2013	243	385	0.63	[0.58; 0.68]	0.3%	0.7%
Olowokere, et al 2020	208	271	0.77	[0.71; 0.82]	0.2%	0.7%
Ruan, et al 2017	821	1225	0.67	[0.64; 0.70]	1.0%	0.7%
Xu, et al 2005	110	237	0.46	[0.40; 0.53]	0.2%	0.7%
Shakhatreh, et al 2006	87	143	0.61	[0.52; 0.69]	0.1%	0.7%
Obermeyer, et al 2007	187	271	0.69	[0.63; 0.74]	0.2%	0.7%
Veigh, et al 2005	139	160	0.87	[0.81; 0.92]	0.1%	0.7%
Oppermann, et al 2012	154	211	0.73	[0.66; 0.79]	0.2%	0.7%
Kamal, et al 2017	259	1100	0.24	[0.21; 0.26]	0.9%	0.7%
Rajbhandari, et al 2017	482	2000	0.24	[0.22; 0.26]	1.6%	0.7%
Cortes, et al 2023	170	358	0.47	[0.42; 0.53]	0.3%	0.7%
Chalise, et al 2022	13	180	0.07	[0.04; 0.12]	0.1%	0.7%
**Common effect model**		**122265**	**0.50**	**[0.50; 0.50]**	**100.0%**	**--**
**Random effects model**			**0.54**	**[0.51; 0.58]**	**--**	**100.0%**

Heterogeneity: $I^2 = 99\%$, $\tau^2 = 0.0483$, $p = 0$

Supplementary Figure 9. Forest plot of prevalence of anxiety among middle-aged women

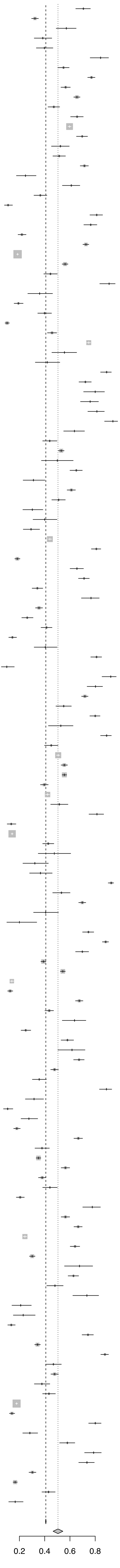

Study	Events	Total	Proportion	95%-CI	Weight (common)	Weight (random)
Adhikari, et al 2022	178	252	0.71	[0.65; 0.76]	0.2%	0.7%
Olaolorun, et al 2009	384	1189	0.32	[0.30; 0.35]	0.9%	0.7%
Javadivala, et al 2020	92	161	0.57	[0.49; 0.65]	0.1%	0.6%
Nagaraj, et al 2021	77	200	0.38	[0.32; 0.46]	0.2%	0.7%
Yisma, et al 2017	90	226	0.40	[0.33; 0.47]	0.2%	0.7%
Korkmaz, et al 2022	91	108	0.84	[0.76; 0.91]	0.1%	0.6%
AlQuaiz, et al 2013	269	490	0.55	[0.50; 0.59]	0.4%	0.7%
Abdel−Salam, et al 2021	651	845	0.77	[0.74; 0.80]	0.6%	0.7%
Anderson, et al1 2004	403	712	0.57	[0.53; 0.60]	0.5%	0.7%
Anderson, et al2 2004	937	1430	0.66	[0.63; 0.68]	1.1%	0.7%
Shafie, et al 2011	223	472	0.47	[0.43; 0.52]	0.4%	0.7%
Aloufi, et al 2022	237	361	0.66	[0.61; 0.71]	0.3%	0.7%
Bl..mel, et al 2012	3629	6079	0.60	[0.58; 0.61]	4.6%	0.7%
Chou, et al 2014	308	442	0.70	[0.65; 0.74]	0.3%	0.7%
Chedraui, et al 2014	107	204	0.52	[0.45; 0.59]	0.2%	0.7%
Chedraui, et al 2010	208	404	0.51	[0.46; 0.56]	0.3%	0.7%
Ojeda, et al 2014	551	771	0.71	[0.68; 0.75]	0.6%	0.7%
Kapur, et al 2009	32	129	0.25	[0.18; 0.33]	0.1%	0.6%
Sharma, et al 2021	124	203	0.61	[0.54; 0.68]	0.2%	0.7%
Rahman, et al 2010	130	356	0.37	[0.32; 0.42]	0.3%	0.7%
Shea, et al 2006	44	399	0.11	[0.08; 0.15]	0.3%	0.7%
Som, et al 2012	203	250	0.81	[0.76; 0.86]	0.2%	0.7%
Alwi, et al 2009	211	276	0.76	[0.71; 0.81]	0.2%	0.7%
Waidyasekera, et al 2009	150	683	0.22	[0.19; 0.25]	0.5%	0.7%
Silva, et al 2013	1028	1415	0.73	[0.70; 0.75]	1.1%	0.7%
Yang, et al 2008	1784	9617	0.19	[0.18; 0.19]	7.3%	0.7%
Yim, et al 2015	996	1774	0.56	[0.54; 0.58]	1.4%	0.7%
Rathnayake, et al 2019	156	350	0.45	[0.39; 0.50]	0.3%	0.7%
Pateliya, et al 2022	91	100	0.91	[0.84; 0.96]	0.1%	0.6%
Srinivasan, et al 2021	36	100	0.36	[0.27; 0.46]	0.1%	0.6%
Chim, et al 2002	95	495	0.19	[0.16; 0.23]	0.4%	0.7%
Dhillon,et al 2006	130	326	0.40	[0.35; 0.45]	0.2%	0.7%
Li, et al 2008	131	1280	0.10	[0.09; 0.12]	1.0%	0.7%
Chuni, et al 2011	334	729	0.46	[0.42; 0.50]	0.6%	0.7%
Nisar, et al 2015	2293	3062	0.75	[0.73; 0.76]	2.3%	0.7%
Zhang, et al 2016	59	106	0.56	[0.46; 0.65]	0.1%	0.6%
Agaba, et al 2017	45	107	0.42	[0.33; 0.52]	0.1%	0.6%
AlQuaiz, et al 2017	203	228	0.89	[0.84; 0.93]	0.2%	0.7%
Saied, et al 2021	247	342	0.72	[0.67; 0.77]	0.3%	0.7%
Sheereen, et al 2022	80	100	0.80	[0.71; 0.87]	0.1%	0.6%
Kang, et al 2021	114	150	0.76	[0.68; 0.83]	0.1%	0.6%
Senthilvel, et al 2018	122	150	0.81	[0.74; 0.87]	0.1%	0.6%
Karmakar, et al 2017	94	100	0.94	[0.87; 0.98]	0.1%	0.6%
Ganapathy, et al 2018	89	140	0.64	[0.55; 0.72]	0.1%	0.6%
Tan, et al 2014	136	309	0.44	[0.38; 0.50]	0.2%	0.7%
Zhao, et al 2023	971	1829	0.53	[0.51; 0.55]	1.4%	0.7%
Han, et al 2023	33	66	0.50	[0.37; 0.63]	0.1%	0.6%
Ko?o?lu, et al 2022	249	383	0.65	[0.60; 0.70]	0.3%	0.7%
AlDughaither, et al 2015	37	119	0.31	[0.23; 0.40]	0.1%	0.6%
Ceylan, et al 2014	534	874	0.61	[0.58; 0.64]	0.7%	0.7%
Thakur,et al 2019	179	351	0.51	[0.46; 0.56]	0.3%	0.7%
Gerber, et al 2018	42	139	0.30	[0.23; 0.39]	0.1%	0.6%
Silvestrin, et al 2016	44	110	0.40	[0.31; 0.50]	0.1%	0.6%
Ghazanfarpour, et al 2016	59	202	0.29	[0.23; 0.36]	0.2%	0.7%
Duffy, et al 2012	1891	4286	0.44	[0.43; 0.46]	3.3%	0.7%
Loutfy, et al 2006	364	450	0.81	[0.77; 0.84]	0.3%	0.7%
Delavar, et al 2011	256	1397	0.18	[0.16; 0.20]	1.1%	0.7%
Chedraui, et al 2007	213	325	0.66	[0.60; 0.71]	0.2%	0.7%
Chedraui, et al 2014	321	451	0.71	[0.67; 0.75]	0.3%	0.7%
Rahman, et al 2011	174	509	0.34	[0.30; 0.38]	0.4%	0.7%
Ahmed, et al 2016	115	150	0.77	[0.69; 0.83]	0.1%	0.6%
Bener, et al 2014	411	1158	0.35	[0.33; 0.38]	0.9%	0.7%
Bener, et al 2000	102	390	0.26	[0.22; 0.31]	0.3%	0.7%
Im, et al 2010	212	512	0.41	[0.37; 0.46]	0.4%	0.7%
Ande, et al 2011	77	533	0.14	[0.12; 0.18]	0.4%	0.7%
Khatoon, et al 2018	49	121	0.40	[0.32; 0.50]	0.1%	0.6%
Budako?lu, et al 2007	274	338	0.81	[0.76; 0.85]	0.3%	0.7%
Sharanya Shre, et al 2016	15	150	0.10	[0.06; 0.16]	0.1%	0.6%
Beura, et al 2020	96	104	0.92	[0.85; 0.97]	0.1%	0.6%
Thapa, et al 2020	142	177	0.80	[0.74; 0.86]	0.1%	0.6%
Ibrahim, et al 2015	872	1214	0.72	[0.69; 0.74]	0.9%	0.7%
Liu, et al 2007	147	267	0.55	[0.49; 0.61]	0.2%	0.7%
Kalhan, et al 2020	320	400	0.80	[0.76; 0.84]	0.3%	0.7%
Rindner, et al 2017	57	108	0.53	[0.43; 0.62]	0.1%	0.6%
Al−Musa, et al 2017	203	228	0.89	[0.84; 0.93]	0.2%	0.7%
Bairy, et al 2009	159	352	0.45	[0.40; 0.51]	0.3%	0.7%
Heinemann, et al1 2008	2429	4791	0.51	[0.49; 0.52]	3.6%	0.7%
Heinemann, et al2 2008	833	1500	0.56	[0.53; 0.58]	1.1%	0.7%
Heinemann, et al3 2008	1671	3006	0.56	[0.54; 0.57]	2.3%	0.7%
Heinemann, et al4 2008	397	1000	0.40	[0.37; 0.43]	0.8%	0.7%
Lawton, et al 2008	1529	3616	0.42	[0.41; 0.44]	2.8%	0.7%
Liu, et al 2008	112	217	0.52	[0.45; 0.58]	0.2%	0.7%
Liu, et al 2007	153	188	0.81	[0.75; 0.87]	0.1%	0.6%
Resmi, et al 2020	54	400	0.14	[0.10; 0.17]	0.3%	0.7%
Wang, et al 2021	1035	7275	0.14	[0.13; 0.15]	5.5%	0.7%
Mustafa, et al 2012	214	500	0.43	[0.38; 0.47]	0.4%	0.7%
Sassoon, et al 2014	30	63	0.48	[0.35; 0.61]	0.0%	0.6%
Mohamed, et al 2014	29	90	0.32	[0.23; 0.43]	0.1%	0.6%
Kong, et al 2019	44	120	0.37	[0.28; 0.46]	0.1%	0.6%
Sharifi, et al 2019	556	600	0.93	[0.90; 0.95]	0.5%	0.7%
Holm, et al 2000	114	214	0.53	[0.46; 0.60]	0.2%	0.7%
Kasuga, et al 2004	746	1069	0.70	[0.67; 0.73]	0.8%	0.7%
Gupta, et al1 2006	42	103	0.41	[0.31; 0.51]	0.1%	0.6%
Gupta, et al2 2006	10	50	0.20	[0.10; 0.34]	0.0%	0.6%
Rahman, et al 2020	291	390	0.75	[0.70; 0.79]	0.3%	0.7%
Abedzadeh−Kalahroudi, et al 2012	618	700	0.88	[0.86; 0.91]	0.5%	0.7%
Diyu, et al 2022	222	318	0.70	[0.64; 0.75]	0.2%	0.7%
Zhang, et al 2016	900	2308	0.39	[0.37; 0.41]	1.8%	0.7%
Ryu, et al 2020	1349	2481	0.54	[0.52; 0.56]	1.9%	0.7%
Ma, et al 2017	327	2336	0.14	[0.13; 0.15]	1.8%	0.7%
Li, et al 2016	134	1062	0.13	[0.11; 0.15]	0.8%	0.7%
Joshi, et al 2015	674	1000	0.67	[0.64; 0.70]	0.8%	0.7%
Ghimire, et al 2015	363	833	0.44	[0.40; 0.47]	0.6%	0.7%
Joseph, et al 2014	70	110	0.64	[0.54; 0.73]	0.1%	0.6%
Xu, et al 2016	128	512	0.25	[0.21; 0.29]	0.4%	0.7%
Sweed, et al 2012	232	400	0.58	[0.53; 0.63]	0.3%	0.7%
Taher, et al 2012	53	86	0.62	[0.51; 0.72]	0.1%	0.6%
G K, et al 2013	336	500	0.67	[0.63; 0.71]	0.4%	0.7%
Bernis, et al 2007	511	1069	0.48	[0.45; 0.51]	0.8%	0.7%
Chedraui, et al 2007	107	300	0.36	[0.30; 0.41]	0.2%	0.7%
Bansal, et al 2015	160	180	0.89	[0.83; 0.93]	0.1%	0.6%
Dutta, et al 2018	54	171	0.32	[0.25; 0.39]	0.1%	0.6%
Nayak, et al 2019	31	290	0.11	[0.07; 0.15]	0.2%	0.7%
Pandey, et al 2020	52	189	0.28	[0.21; 0.34]	0.1%	0.6%
Sievert, et al 2007	156	869	0.18	[0.15; 0.21]	0.7%	0.7%
Sievert, et al 2003	503	755	0.67	[0.63; 0.70]	0.6%	0.7%
Obermeyer, et al 2004	111	293	0.38	[0.32; 0.44]	0.2%	0.7%
Keenan, et al 2003	913	2602	0.35	[0.33; 0.37]	2.0%	0.7%
Gollschewski, et al 2004	487	863	0.56	[0.53; 0.60]	0.7%	0.7%
Biri, et al 2005	399	1049	0.38	[0.35; 0.41]	0.8%	0.7%
Obermeyer, et al 2002	132	299	0.44	[0.38; 0.50]	0.2%	0.7%
Loh, et al 2005	135	656	0.21	[0.18; 0.24]	0.5%	0.7%
Chaopotong, et al 2005	115	148	0.78	[0.70; 0.84]	0.1%	0.6%
Anderson, et al1 2004	487	863	0.56	[0.53; 0.60]	0.7%	0.7%
Anderson, et al2 2004	548	824	0.67	[0.63; 0.70]	0.6%	0.7%
Glod, et al 2006	804	3302	0.24	[0.23; 0.26]	2.5%	0.7%
Masjoudl, et al 2017	413	645	0.64	[0.60; 0.68]	0.5%	0.7%
Kapoor, et al 2021	503	1670	0.30	[0.28; 0.32]	1.3%	0.7%
Durairaj, et al 2022	50	74	0.68	[0.56; 0.78]	0.1%	0.6%
Abou−Raya, et al 2016	339	540	0.63	[0.59; 0.67]	0.4%	0.7%
Krajewska−Ferishah, et al1 2022	116	241	0.48	[0.42; 0.55]	0.2%	0.7%
Krajewska−Ferishah, et al2 2022	58	79	0.73	[0.62; 0.83]	0.1%	0.6%
Krajewska−Ferishah, et al3 2022	25	119	0.21	[0.14; 0.29]	0.1%	0.6%
Krajewska−Ferishah, et al4 2022	23	100	0.23	[0.15; 0.32]	0.1%	0.6%
Ogwumike, et al 2012	74	547	0.14	[0.11; 0.17]	0.4%	0.7%
Sierra, et al 2004	286	385	0.74	[0.70; 0.79]	0.3%	0.7%
Pe_rez−L.pez, et al 2013	597	1739	0.34	[0.32; 0.37]	1.3%	0.7%
Dasgupta, et al 2012	421	480	0.88	[0.84; 0.91]	0.4%	0.7%
Abdullah, et al 2016	121	258	0.47	[0.41; 0.53]	0.2%	0.7%
Hickey, et al 2017	548	1145	0.48	[0.45; 0.51]	0.9%	0.7%
Blell, et al 2014	96	254	0.38	[0.32; 0.44]	0.2%	0.7%
Dienye, et al 2013	167	385	0.43	[0.38; 0.48]	0.3%	0.7%
Whiteley, et al 2013	1566	8811	0.18	[0.17; 0.19]	6.7%	0.7%
Bener, et al 2017	155	1101	0.14	[0.12; 0.16]	0.8%	0.7%
Olowokere, et al 2020	217	271	0.80	[0.75; 0.85]	0.2%	0.7%
Xu, et al 2005	67	237	0.28	[0.23; 0.34]	0.2%	0.7%
Obermeyer, et al 2007	157	271	0.58	[0.52; 0.64]	0.2%	0.7%
Veigh, et al 2005	126	160	0.79	[0.72; 0.85]	0.1%	0.6%
Oppermann, et al 2012	155	211	0.73	[0.67; 0.79]	0.2%	0.7%
Kamal, et al 2017	333	1100	0.30	[0.28; 0.33]	0.8%	0.7%
Rajbhandari, et al 2017	332	2000	0.17	[0.15; 0.18]	1.5%	0.7%
Cortes, et al 2023	154	358	0.43	[0.38; 0.48]	0.3%	0.7%
Chalise, et al 2022	30	180	0.17	[0.12; 0.23]	0.1%	0.6%
**Common effect model**		**131236**	**0.41**	**[0.41; 0.41]**	**100.0%**	**--**
**Random effects model**			**0.51**	**[0.47; 0.54]**	**--**	**100.0%**

Heterogeneity: $I^2 = 99\%$, $\tau^2 = 0.0585$, $p = 0$

Supplementary Figure 10. Forest plot of prevalence of mood swings among middle-aged women

Study	Events	Total	Proportion	95%−CI	Weight (common)	Weight (random)
Lan, et al 2017	418	985	0.42	[0.39; 0.46]	1.2%	1.7%
Wang, et al 2021	2367	6364	0.37	[0.36; 0.38]	7.8%	1.7%
Li, et al 2012	6858	20275	0.34	[0.33; 0.34]	24.7%	1.7%
Genazzani, et al1 2006	372	611	0.61	[0.57; 0.65]	0.7%	1.7%
Genazzani, et al2 2006	312	600	0.52	[0.48; 0.56]	0.7%	1.7%
Genazzani, et al3 2006	340	596	0.57	[0.53; 0.61]	0.7%	1.7%
Genazzani, et al4 2006	339	594	0.57	[0.53; 0.61]	0.7%	1.7%
Genazzani, et al5 2006	342	600	0.57	[0.53; 0.61]	0.7%	1.7%
Genazzani, et al6 2006	336	600	0.56	[0.52; 0.60]	0.7%	1.7%
Genazzani, et al7 2006	342	600	0.57	[0.53; 0.61]	0.7%	1.7%
Du, et al 2020	574	3141	0.18	[0.17; 0.20]	3.8%	1.7%
Borker, et al 2013	96	106	0.91	[0.83; 0.95]	0.1%	1.6%
Pateliya, et al 2022	44	100	0.44	[0.34; 0.54]	0.1%	1.6%
Dhillon,et al 2006	168	326	0.52	[0.46; 0.57]	0.4%	1.7%
Haines, et al1 2005	152	248	0.61	[0.55; 0.67]	0.3%	1.6%
Haines, et al2 2005	138	195	0.71	[0.64; 0.77]	0.2%	1.6%
Haines, et al3 2005	23	59	0.39	[0.27; 0.53]	0.1%	1.5%
Haines, et al4 2005	61	97	0.63	[0.52; 0.72]	0.1%	1.6%
Haines, et al5 2005	15	24	0.62	[0.41; 0.81]	0.0%	1.4%
Haines, et al6 2005	36	58	0.62	[0.48; 0.74]	0.1%	1.5%
Haines, et al7 2005	35	81	0.43	[0.32; 0.55]	0.1%	1.6%
Haines, et al8 2005	99	150	0.66	[0.58; 0.74]	0.2%	1.6%
Haines, et al9 2005	96	100	0.96	[0.90; 0.99]	0.1%	1.6%
Nappi, et al1 2008	117	300	0.39	[0.33; 0.45]	0.4%	1.7%
Nappi, et al2 2008	99	300	0.33	[0.28; 0.39]	0.4%	1.7%
Nappi, et al3 2008	108	300	0.36	[0.31; 0.42]	0.4%	1.7%
Nappi, et al4 2008	96	301	0.32	[0.27; 0.37]	0.4%	1.7%
Nappi, et al5 2008	132	301	0.44	[0.38; 0.50]	0.4%	1.7%
Nappi, et al6 2008	108	301	0.36	[0.30; 0.42]	0.4%	1.7%
G..recka, et al 2022	193	349	0.55	[0.50; 0.61]	0.4%	1.7%
Chopra, et al 2022	386	504	0.77	[0.73; 0.80]	0.6%	1.7%
Vaccaro, et al 2021	336	1028	0.33	[0.30; 0.36]	1.3%	1.7%
Duffy, et al 2012	1957	4288	0.46	[0.44; 0.47]	5.2%	1.7%
Delavar, et al 2011	91	1397	0.07	[0.05; 0.08]	1.7%	1.7%
Im, et al 2010	249	512	0.49	[0.44; 0.53]	0.6%	1.7%
Nusrat, et al 2008	492	863	0.57	[0.54; 0.60]	1.1%	1.7%
Resmi, et al 2020	118	400	0.29	[0.25; 0.34]	0.5%	1.7%
Mustafa, et al 2012	207	500	0.41	[0.37; 0.46]	0.6%	1.7%
Nappi, et al 2012	1619	3520	0.46	[0.44; 0.48]	4.3%	1.7%
Gupta, et al1 2006	49	103	0.48	[0.38; 0.58]	0.1%	1.6%
Gupta, et al2 2006	6	50	0.12	[0.05; 0.24]	0.1%	1.5%
Fu, et al 2020	1273	3199	0.40	[0.38; 0.42]	3.9%	1.7%
Joshi, et al 2015	355	1000	0.35	[0.33; 0.39]	1.2%	1.7%
Sievert, et al 2007	235	869	0.27	[0.24; 0.30]	1.1%	1.7%
Huang, et al1 2010	178	300	0.59	[0.54; 0.65]	0.4%	1.7%
Huang, et al2 2010	67	100	0.67	[0.57; 0.76]	0.1%	1.6%
Huang, et al3 2010	131	300	0.44	[0.38; 0.49]	0.4%	1.7%
Huang, et al4 2010	64	150	0.43	[0.35; 0.51]	0.2%	1.6%
Huang, et al5 2010	117	150	0.78	[0.71; 0.84]	0.2%	1.6%
Fagulha, et al 2011	381	728	0.52	[0.49; 0.56]	0.9%	1.7%
Krajewska−Ferishah, et al1 2022	200	241	0.83	[0.78; 0.88]	0.3%	1.6%
Krajewska−Ferishah, et al2 2022	53	79	0.67	[0.56; 0.77]	0.1%	1.6%
Krajewska−Ferishah, et al3 2022	100	119	0.84	[0.76; 0.90]	0.1%	1.6%
Krajewska−Ferishah, et al4 2022	60	100	0.60	[0.50; 0.70]	0.1%	1.6%
Jin, et al 2014	529	1215	0.44	[0.41; 0.46]	1.5%	1.7%
Huang, et al 2020	929	1501	0.62	[0.59; 0.64]	1.8%	1.7%
Chu, et al 2022	1145	1748	0.66	[0.63; 0.68]	2.1%	1.7%
Whiteley, et al 2013	2083	8811	0.24	[0.23; 0.25]	10.8%	1.7%
An, et al 2023	4248	7309	0.58	[0.57; 0.59]	8.9%	1.7%
Rajbhandari, et al 2017	333	2000	0.17	[0.15; 0.18]	2.4%	1.7%
Chalise, et al 2022	6	180	0.03	[0.01; 0.07]	0.2%	1.6%
**Common effect model**		**81926**	**0.39**	**[0.39; 0.39]**	**100.0%**	**--**
**Random effects model**			**0.49**	**[0.44; 0.54]**	**--**	**100.0%**

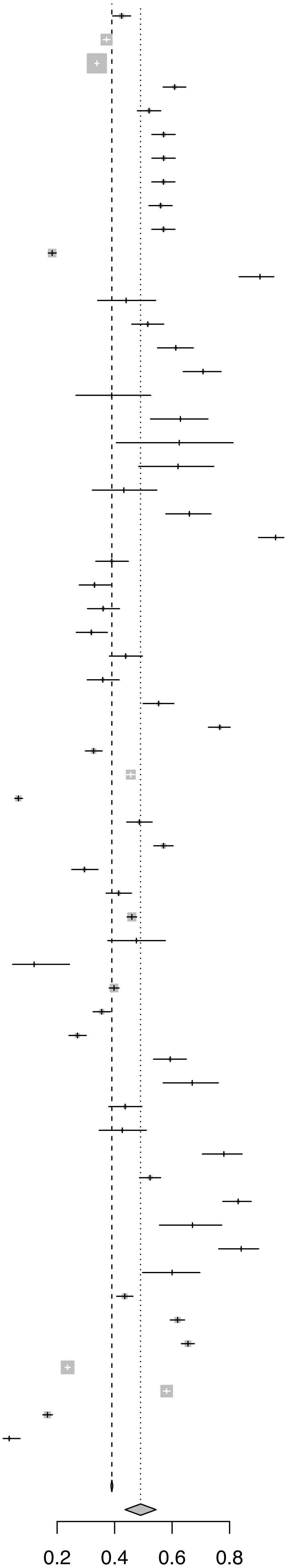

Heterogeneity: $I^2 = 99\%$, $\tau^2 = 0.0447$, $p = 0$

Supplementary Figure 11. Forest plot of prevalence of sexual problems among middle-aged women

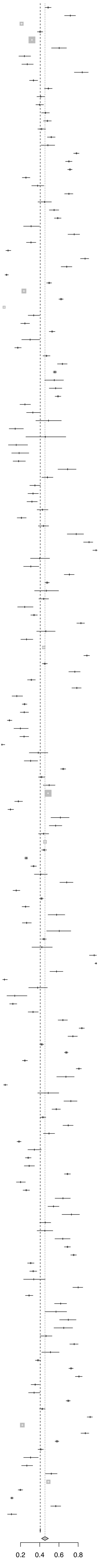

Study	Events	Total	Proportion	95%–CI	Weight (common)	Weight (random)
Lan, et al 2017	480	985	0.49	[0.46; 0.52]	0.5%	0.5%
Adhikari, et al 2022	181	252	0.72	[0.66; 0.77]	0.1%	0.5%
Wang, et al 2021	1343	6364	0.21	[0.20; 0.22]	3.5%	0.5%
Olaolorun, et al 2009	480	1189	0.40	[0.38; 0.43]	0.7%	0.5%
Li, et al 2012	6468	20275	0.32	[0.31; 0.33]	11.2%	0.5%
Javadivala, et al 2020	97	161	0.60	[0.52; 0.68]	0.1%	0.5%
Nagaraj, et al 2021	48	200	0.24	[0.18; 0.31]	0.1%	0.5%
Yisma, et al 2017	61	226	0.27	[0.21; 0.33]	0.1%	0.5%
Korkmaz, et al 2022	91	108	0.84	[0.76; 0.91]	0.1%	0.5%
AlQuaiz, et al 2013	164	490	0.33	[0.29; 0.38]	0.3%	0.5%
Genazzani, et al1 2006	299	611	0.49	[0.45; 0.53]	0.3%	0.5%
Genazzani, et al2 2006	246	600	0.41	[0.37; 0.45]	0.3%	0.5%
Genazzani, et al3 2006	238	596	0.40	[0.36; 0.44]	0.3%	0.5%
Genazzani, et al4 2006	273	594	0.46	[0.42; 0.50]	0.3%	0.5%
Genazzani, et al5 2006	288	600	0.48	[0.44; 0.52]	0.3%	0.5%
Genazzani, et al6 2006	252	600	0.42	[0.38; 0.46]	0.3%	0.5%
Genazzani, et al7 2006	312	600	0.52	[0.48; 0.56]	0.3%	0.5%
Aaron, et al 2002	97	200	0.48	[0.41; 0.56]	0.1%	0.5%
Abdel–Salam, et al 2021	661	845	0.78	[0.75; 0.81]	0.5%	0.5%
Anderson, et al1 2004	501	712	0.70	[0.67; 0.74]	0.4%	0.5%
Anderson, et al2 2004	1022	1430	0.71	[0.69; 0.74]	0.8%	0.5%
Shafie, et al 2011	121	472	0.26	[0.22; 0.30]	0.3%	0.5%
Aloufi, et al 2022	90	238	0.38	[0.32; 0.44]	0.1%	0.5%
Chou, et al 2014	311	442	0.70	[0.66; 0.75]	0.2%	0.5%
Chedraui, et al 2014	92	204	0.45	[0.38; 0.52]	0.1%	0.5%
Chedraui, et al 2010	222	404	0.55	[0.50; 0.60]	0.2%	0.5%
Ojeda, et al 2014	453	771	0.59	[0.55; 0.62]	0.4%	0.5%
Kapur, et al 2009	40	129	0.31	[0.23; 0.40]	0.1%	0.5%
Sharma, et al 2021	154	203	0.76	[0.69; 0.82]	0.1%	0.5%
Rahman, et al 2010	110	356	0.31	[0.26; 0.36]	0.2%	0.5%
Shea, et al 2006	28	399	0.07	[0.05; 0.10]	0.2%	0.5%
Som, et al 2012	218	250	0.87	[0.82; 0.91]	0.1%	0.5%
Alwi, et al 2009	188	276	0.68	[0.62; 0.74]	0.2%	0.5%
Waidyasekera, et al 2009	37	683	0.05	[0.04; 0.07]	0.4%	0.5%
Silva, et al 2013	705	1415	0.50	[0.47; 0.52]	0.8%	0.5%
Yang, et al 2008	2264	9617	0.24	[0.23; 0.24]	5.3%	0.5%
Yim, et al 2015	1104	1774	0.62	[0.60; 0.64]	1.0%	0.5%
Du, et al 2020	91	3141	0.03	[0.02; 0.04]	1.7%	0.5%
Singh, et al 2014	85	252	0.34	[0.28; 0.40]	0.1%	0.5%
Rathnayake, et al 2019	86	350	0.25	[0.20; 0.29]	0.2%	0.5%
Baquedano, et al 2023	560	1059	0.53	[0.50; 0.56]	0.6%	0.5%
Srinivasan, et al 2021	30	100	0.30	[0.21; 0.40]	0.1%	0.5%
Chim, et al 2002	85	495	0.17	[0.14; 0.21]	0.3%	0.5%
Chuni, et al 2011	343	729	0.47	[0.43; 0.51]	0.4%	0.5%
Valadares, et al 2011	229	360	0.64	[0.58; 0.69]	0.2%	0.5%
Nisar, et al 2015	1707	3062	0.56	[0.54; 0.58]	1.7%	0.5%
Agaba, et al 2017	59	107	0.55	[0.45; 0.65]	0.1%	0.5%
AlQuaiz, et al 2017	129	228	0.57	[0.50; 0.63]	0.1%	0.5%
Grigoriou, et al 2013	605	1025	0.59	[0.56; 0.62]	0.6%	0.5%
Haines, et al1 2005	61	248	0.25	[0.19; 0.30]	0.1%	0.5%
Haines, et al2 2005	64	195	0.33	[0.26; 0.40]	0.1%	0.5%
Haines, et al3 2005	29	59	0.49	[0.36; 0.63]	0.0%	0.5%
Haines, et al4 2005	14	97	0.14	[0.08; 0.23]	0.1%	0.5%
Haines, et al5 2005	11	24	0.46	[0.26; 0.67]	0.0%	0.5%
Haines, et al6 2005	9	58	0.16	[0.07; 0.27]	0.0%	0.5%
Haines, et al7 2005	15	81	0.19	[0.11; 0.29]	0.0%	0.5%
Haines, et al8 2005	27	150	0.18	[0.12; 0.25]	0.1%	0.5%
Haines, et al9 2005	69	100	0.69	[0.59; 0.78]	0.1%	0.5%
Nappi, et al1 2008	144	300	0.48	[0.42; 0.54]	0.2%	0.5%
Nappi, et al2 2008	105	300	0.35	[0.30; 0.41]	0.2%	0.5%
Nappi, et al3 2008	99	300	0.33	[0.28; 0.39]	0.2%	0.5%
Nappi, et al4 2008	96	301	0.32	[0.27; 0.37]	0.2%	0.5%
Nappi, et al5 2008	129	301	0.43	[0.37; 0.49]	0.2%	0.5%
Nappi, et al6 2008	63	301	0.21	[0.16; 0.26]	0.2%	0.5%
Saied, et al 2021	150	342	0.44	[0.39; 0.49]	0.2%	0.5%
Sheereen, et al 2022	78	100	0.78	[0.69; 0.86]	0.1%	0.5%
Kang, et al 2021	137	150	0.91	[0.86; 0.95]	0.1%	0.5%
Senthilvel, et al 2018	148	150	0.99	[0.95; 1.00]	0.1%	0.5%
Karmakar, et al 2017	40	100	0.40	[0.30; 0.50]	0.1%	0.5%
Ganapathy, et al 2018	43	140	0.31	[0.23; 0.39]	0.1%	0.5%
Tan, et al 2014	219	309	0.71	[0.65; 0.76]	0.2%	0.5%
Zhao, et al 2023	872	1829	0.48	[0.45; 0.50]	1.0%	0.5%
Han, et al 2023	31	66	0.47	[0.35; 0.60]	0.0%	0.5%
Ko?o?lu, et al 2022	169	383	0.44	[0.39; 0.49]	0.2%	0.5%
AlDughaither, et al 2015	29	119	0.24	[0.17; 0.33]	0.1%	0.5%
Ceylan, et al 2014	252	740	0.34	[0.31; 0.38]	0.4%	0.5%
Thakur,et al 2019	291	351	0.83	[0.79; 0.87]	0.2%	0.5%
Silvestrin, et al 2016	51	110	0.46	[0.37; 0.56]	0.1%	0.5%
Ghazanfarpour, et al 2016	53	202	0.26	[0.20; 0.33]	0.1%	0.5%
Duffy, et al 2012	1886	4268	0.44	[0.43; 0.46]	2.4%	0.5%
Loutfy, et al 2006	401	450	0.89	[0.86; 0.92]	0.2%	0.5%
Delavar, et al 2011	633	1397	0.45	[0.43; 0.48]	0.8%	0.5%
Chedraui, et al 2007	160	209	0.77	[0.70; 0.82]	0.1%	0.5%
Rahman, et al 2011	159	509	0.31	[0.27; 0.35]	0.3%	0.5%
Aguilar–Zavala, et al 2012	228	290	0.79	[0.73; 0.83]	0.2%	0.5%
Agwu, et al 2008	30	186	0.16	[0.11; 0.22]	0.1%	0.5%
Bener, et al 2014	280	1158	0.24	[0.22; 0.27]	0.6%	0.5%
Bener, et al 2000	93	390	0.24	[0.20; 0.28]	0.2%	0.5%
Ande, et al 2011	45	533	0.08	[0.06; 0.11]	0.3%	0.5%
Khatoon, et al 2018	24	121	0.20	[0.13; 0.28]	0.1%	0.5%
Budako?lu, et al 2007	80	338	0.24	[0.19; 0.29]	0.2%	0.5%
Sharanya Shre, et al 2016	1	150	0.01	[0.00; 0.04]	0.1%	0.5%
Beura, et al 2020	40	104	0.38	[0.29; 0.49]	0.1%	0.5%
Thapa, et al 2020	54	177	0.31	[0.24; 0.38]	0.1%	0.5%
Ibrahim, et al 2015	782	1214	0.64	[0.62; 0.67]	0.7%	0.5%
Nusrat, et al 2008	362	863	0.42	[0.39; 0.45]	0.5%	0.5%
Liu, et al 2007	133	267	0.50	[0.44; 0.56]	0.1%	0.5%
Lee, et al 2020	10161	20882	0.49	[0.48; 0.49]	11.6%	0.5%
Modoodi, et al 2020	62	350	0.18	[0.14; 0.22]	0.2%	0.5%
Kalhan, et al 2020	38	400	0.10	[0.07; 0.13]	0.2%	0.5%
Rindner, et al 2017	67	109	0.61	[0.52; 0.71]	0.1%	0.5%
Al–Musa, et al 2017	129	228	0.57	[0.50; 0.63]	0.1%	0.5%
Bairy, et al 2009	155	352	0.44	[0.39; 0.49]	0.2%	0.5%
Heinemann, et al1 2008	2180	4791	0.46	[0.44; 0.47]	2.7%	0.5%
Heinemann, et al2 2008	669	1500	0.45	[0.42; 0.47]	0.8%	0.5%
Heinemann, et al3 2008	779	3006	0.26	[0.24; 0.28]	1.7%	0.5%
Heinemann, et al4 2008	336	1000	0.34	[0.31; 0.37]	0.6%	0.5%
Liu, et al 2008	89	217	0.41	[0.34; 0.48]	0.1%	0.5%
Liu, et al 2007	128	188	0.68	[0.61; 0.75]	0.1%	0.5%
Resmi, et al 2020	62	400	0.15	[0.12; 0.19]	0.2%	0.5%
Ashrafi, et al 2009	1027	2462	0.42	[0.40; 0.44]	1.4%	0.5%
Deveci, et al 2010	131	519	0.25	[0.22; 0.29]	0.3%	0.5%
O?urlu, et al 2011	76	132	0.58	[0.49; 0.66]	0.1%	0.5%
Anolue, et al 2012	92	349	0.26	[0.22; 0.31]	0.2%	0.5%
Sassoon, et al 2014	38	63	0.60	[0.47; 0.72]	0.0%	0.5%
Liu, et al 2013	749	1686	0.44	[0.42; 0.47]	0.9%	0.5%
Mohamed, et al 2014	38	90	0.42	[0.32; 0.53]	0.1%	0.5%
Kong, et al 2019	116	120	0.97	[0.92; 0.99]	0.1%	0.5%
Sharifi, et al 2019	592	600	0.99	[0.97; 0.99]	0.3%	0.5%
Holm, et al 2000	123	214	0.57	[0.51; 0.64]	0.1%	0.5%
Kaur, et al 2004	8	245	0.03	[0.01; 0.06]	0.1%	0.5%
Gupta, et al1 2006	39	103	0.38	[0.28; 0.48]	0.1%	0.5%
Gupta, et al2 2006	7	50	0.14	[0.06; 0.27]	0.0%	0.5%
Inayat, et al 2017	37	308	0.12	[0.09; 0.16]	0.2%	0.5%
Mathialagan, et al 2022	106	321	0.33	[0.28; 0.38]	0.2%	0.5%
Rahman, et al 2020	250	390	0.64	[0.59; 0.69]	0.2%	0.5%
Abedzadeh–Kalahroudi, et al 2012	587	700	0.84	[0.81; 0.87]	0.4%	0.5%
Diyu, et al 2022	237	318	0.75	[0.69; 0.79]	0.2%	0.5%
Zhang, et al 2016	969	2308	0.42	[0.40; 0.44]	1.3%	0.5%
Ryu, et al 2020	1679	2481	0.68	[0.66; 0.70]	1.4%	0.5%
Joshi, et al 2015	245	1000	0.24	[0.22; 0.27]	0.6%	0.5%
Ghimire, et al 2015	673	833	0.81	[0.78; 0.83]	0.5%	0.5%
Joseph, et al 2014	74	110	0.67	[0.58; 0.76]	0.1%	0.5%
Sweed, et al 2012	16	400	0.04	[0.02; 0.06]	0.2%	0.5%
Taher, et al 2012	42	86	0.49	[0.38; 0.60]	0.0%	0.5%
Ryan, et al 2012	126	174	0.72	[0.65; 0.79]	0.1%	0.5%
G K, et al 2013	286	500	0.57	[0.53; 0.62]	0.3%	0.5%
Bernis, et al 2007	463	1069	0.43	[0.40; 0.46]	0.6%	0.5%
Chedraui, et al 2007	209	300	0.70	[0.64; 0.75]	0.2%	0.5%
Nayak, et al 2019	144	290	0.50	[0.44; 0.56]	0.2%	0.5%
Geng, et al 2018	238	1294	0.18	[0.16; 0.21]	0.7%	0.5%
Pandey, et al 2020	65	189	0.34	[0.28; 0.42]	0.1%	0.5%
Sievert, et al 2007	243	869	0.28	[0.25; 0.31]	0.5%	0.5%
Obermeyer, et al 2004	85	293	0.29	[0.24; 0.35]	0.2%	0.5%
Gollschewski, et al 2004	569	826	0.69	[0.66; 0.72]	0.5%	0.5%
Obermeyer, et al 2002	60	299	0.20	[0.16; 0.25]	0.2%	0.5%
Loh, et al 2005	170	656	0.26	[0.23; 0.29]	0.4%	0.5%
Chaopotong, et al 2005	95	148	0.64	[0.56; 0.72]	0.1%	0.5%
Huang, et al1 2010	163	300	0.54	[0.49; 0.60]	0.2%	0.5%
Huang, et al2 2010	73	100	0.73	[0.63; 0.81]	0.1%	0.5%
Huang, et al3 2010	137	300	0.46	[0.40; 0.51]	0.2%	0.5%
Huang, et al4 2010	68	150	0.45	[0.37; 0.54]	0.1%	0.5%
Huang, et al5 2010	96	150	0.64	[0.56; 0.72]	0.1%	0.5%
Anderson, et al1 2004	569	826	0.69	[0.66; 0.72]	0.5%	0.5%
Anderson, et al2 2004	617	819	0.75	[0.72; 0.78]	0.5%	0.5%
Fagulha, et al 2011	223	728	0.31	[0.27; 0.34]	0.4%	0.5%
Masjoudl, et al 2017	214	645	0.33	[0.30; 0.37]	0.4%	0.5%
Durairaj, et al 2022	25	74	0.34	[0.23; 0.46]	0.0%	0.5%
Bala, et al 2022	196	245	0.80	[0.74; 0.85]	0.1%	0.5%
Abou–Raya, et al 2016	156	540	0.29	[0.25; 0.33]	0.3%	0.5%
Krajewska–Ferishah, et al1 2022	149	241	0.62	[0.55; 0.68]	0.1%	0.5%
Krajewska–Ferishah, et al2 2022	45	79	0.57	[0.45; 0.68]	0.0%	0.5%
Krajewska–Ferishah, et al3 2022	83	119	0.70	[0.61; 0.78]	0.1%	0.5%
Krajewska–Ferishah, et al4 2022	65	100	0.65	[0.55; 0.74]	0.1%	0.5%
Ornat, et al 2013	121	260	0.47	[0.40; 0.53]	0.1%	0.5%
Sierra, et al 2004	292	385	0.76	[0.71; 0.80]	0.2%	0.5%
Castelo–Branco, et al 2005	64	125	0.51	[0.42; 0.60]	0.1%	0.5%
Jin, et al 2014	464	1215	0.38	[0.35; 0.41]	0.7%	0.5%
Huang, et al 2020	1090	1501	0.73	[0.70; 0.75]	0.8%	0.5%
Dasgupta, et al 2012	388	480	0.81	[0.77; 0.84]	0.3%	0.5%
Wu, et al 2023	165	468	0.35	[0.31; 0.40]	0.3%	0.5%
Abdullah, et al 2016	88	258	0.34	[0.28; 0.40]	0.1%	0.5%
Chu, et al 2022	1218	1748	0.70	[0.67; 0.72]	1.0%	0.5%
Hickey, et al 2017	485	1137	0.43	[0.40; 0.46]	0.6%	0.5%
Dienye, et al 2013	356	385	0.92	[0.89; 0.95]	0.2%	0.5%
Whiteley, et al 2013	1927	8811	0.22	[0.21; 0.23]	4.9%	0.5%
Olowokere, et al 2020	237	271	0.87	[0.83; 0.91]	0.2%	0.5%
Zhu, et al 2021	1391	2400	0.58	[0.56; 0.60]	1.3%	0.5%
Ruan, et al 2017	502	1225	0.41	[0.38; 0.44]	0.7%	0.5%
Devi, et al 2005	46	151	0.30	[0.23; 0.38]	0.1%	0.5%
Xu, et al 2005	63	237	0.27	[0.21; 0.33]	0.1%	0.5%
Obermeyer, et al 2007	141	271	0.52	[0.46; 0.58]	0.2%	0.5%
An, et al 2023	3581	7309	0.49	[0.48; 0.50]	4.0%	0.5%
Kamal, et al 2017	218	1100	0.20	[0.18; 0.22]	0.6%	0.5%
Rajbhandari, et al 2017	220	2000	0.11	[0.10; 0.12]	1.1%	0.5%
Cortes, et al 2023	203	358	0.57	[0.51; 0.62]	0.2%	0.5%
Chalise, et al 2022	19	180	0.11	[0.06; 0.16]	0.1%	0.5%
**Common effect model**		**180618**	**0.41**	**[0.40; 0.41]**	**100.0%**	**--**
**Random effects model**			**0.45**	**[0.42; 0.49]**	**--**	**100.0%**

Heterogeneity: $I^2 = 99\%$, $\tau^2 = 0.0611$, $p = 0$

Supplementary Figure 12. Forest plot of prevalence of vagina dryness among middle-aged women

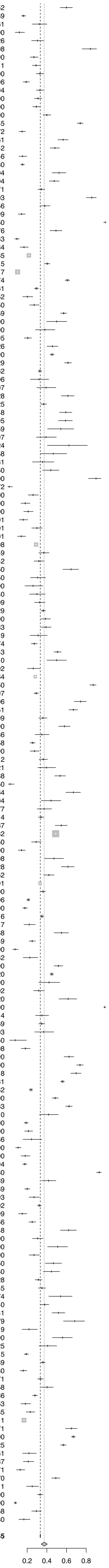

Study	Events	Total	Proportion	95%−CI	Weight (common)	Weight (random)
Adhikari, et al 2022	151	252	0.60	[0.54; 0.66]	0.2%	0.5%
Olaolorun, et al 2009	193	1189	0.16	[0.14; 0.18]	0.7%	0.6%
Javadivala, et al 2020	52	161	0.32	[0.25; 0.40]	0.1%	0.5%
Nagaraj, et al 2021	24	200	0.12	[0.08; 0.17]	0.1%	0.5%
Yisma, et al 2017	69	226	0.31	[0.25; 0.37]	0.1%	0.5%
Korkmaz, et al 2022	91	108	0.84	[0.76; 0.91]	0.1%	0.5%
AlQuaiz, et al 2013	132	490	0.27	[0.23; 0.31]	0.3%	0.5%
Genazzani, et al1 2006	177	611	0.29	[0.25; 0.33]	0.4%	0.6%
Genazzani, et al2 2006	198	600	0.33	[0.29; 0.37]	0.4%	0.6%
Genazzani, et al3 2006	113	596	0.19	[0.16; 0.22]	0.4%	0.6%
Genazzani, et al4 2006	196	594	0.33	[0.29; 0.37]	0.4%	0.6%
Genazzani, et al5 2006	186	600	0.31	[0.27; 0.35]	0.4%	0.6%
Genazzani, et al6 2006	176	600	0.29	[0.26; 0.33]	0.4%	0.6%
Genazzani, et al7 2006	240	600	0.40	[0.36; 0.44]	0.4%	0.6%
Abdel−Salam, et al 2021	625	845	0.74	[0.71; 0.77]	0.5%	0.6%
Shafie, et al 2011	70	472	0.15	[0.12; 0.18]	0.3%	0.5%
Aloufi, et al 2022	204	361	0.57	[0.51; 0.62]	0.2%	0.5%
Chou, et al 2014	213	442	0.48	[0.43; 0.53]	0.3%	0.5%
Fallahzadeh, et al 2007	53	346	0.15	[0.12; 0.20]	0.2%	0.5%
Fuh, et al 2003	207	1360	0.15	[0.13; 0.17]	0.8%	0.6%
Chedraui, et al 2014	107	204	0.52	[0.45; 0.59]	0.1%	0.5%
Chedraui, et al 2010	192	404	0.48	[0.43; 0.53]	0.2%	0.5%
Ojeda, et al 2014	265	771	0.34	[0.31; 0.38]	0.5%	0.6%
Sharma, et al 2021	174	203	0.86	[0.80; 0.90]	0.1%	0.5%
Rahman, et al 2010	135	356	0.38	[0.33; 0.43]	0.2%	0.5%
Shea, et al 2006	57	399	0.14	[0.11; 0.18]	0.2%	0.5%
Som, et al 2012	249	250	1.00	[0.98; 1.00]	0.1%	0.5%
Alwi, et al 2009	136	276	0.49	[0.43; 0.55]	0.2%	0.5%
Waidyasekera, et al 2009	65	683	0.10	[0.07; 0.12]	0.4%	0.6%
He, et al 2021	56	334	0.17	[0.13; 0.21]	0.2%	0.5%
Malacara, et al 2002	1661	7635	0.22	[0.21; 0.23]	4.6%	0.6%
Silva, et al 2013	572	1415	0.40	[0.38; 0.43]	0.8%	0.6%
Yang, et al 2008	987	9617	0.10	[0.10; 0.11]	5.8%	0.6%
Yim, et al 2015	1081	1774	0.61	[0.59; 0.63]	1.1%	0.6%
Yokota, et al 2016	577	1961	0.29	[0.27; 0.31]	1.2%	0.6%
Singh, et al 2014	51	252	0.20	[0.15; 0.26]	0.2%	0.5%
Rathnayake, et al 2019	95	350	0.27	[0.23; 0.32]	0.2%	0.5%
Baquedano, et al 2023	604	1059	0.57	[0.54; 0.60]	0.6%	0.6%
Pateliya, et al 2022	50	100	0.50	[0.40; 0.60]	0.1%	0.5%
Srinivasan, et al 2021	38	100	0.38	[0.28; 0.48]	0.1%	0.5%
Chim, et al 2002	102	495	0.21	[0.17; 0.24]	0.3%	0.5%
Dhillon,et al 2006	149	326	0.46	[0.40; 0.51]	0.2%	0.5%
Santoro,.et al 2009	1038	2290	0.45	[0.43; 0.47]	1.4%	0.6%
Chuni, et al 2011	449	729	0.62	[0.58; 0.65]	0.4%	0.6%
Nisar, et al 2015	1008	3062	0.33	[0.31; 0.35]	1.8%	0.6%
Zhang, et al 2016	34	106	0.32	[0.23; 0.42]	0.1%	0.5%
Agaba, et al 2017	42	107	0.39	[0.30; 0.49]	0.1%	0.5%
AlQuaiz, et al 2017	140	228	0.61	[0.55; 0.68]	0.1%	0.5%
Grigoriou, et al 2013	377	1025	0.37	[0.34; 0.40]	0.6%	0.6%
Haines, et al1 2005	147	248	0.59	[0.53; 0.65]	0.1%	0.5%
Haines, et al2 2005	115	195	0.59	[0.52; 0.66]	0.1%	0.5%
Haines, et al3 2005	32	59	0.54	[0.41; 0.67]	0.0%	0.5%
Haines, et al4 2005	38	97	0.39	[0.29; 0.50]	0.1%	0.5%
Haines, et al5 2005	15	24	0.62	[0.41; 0.81]	0.0%	0.5%
Haines, et al6 2005	27	58	0.47	[0.33; 0.60]	0.0%	0.5%
Haines, et al7 2005	29	81	0.36	[0.25; 0.47]	0.0%	0.5%
Haines, et al8 2005	66	150	0.44	[0.36; 0.52]	0.1%	0.5%
Haines, et al9 2005	90	100	0.90	[0.82; 0.95]	0.1%	0.5%
Dennerstein, et al 2000	3	172	0.02	[0.00; 0.05]	0.1%	0.5%
Nappi, et al1 2008	78	300	0.26	[0.21; 0.31]	0.2%	0.5%
Nappi, et al2 2008	54	300	0.18	[0.14; 0.23]	0.2%	0.5%
Nappi, et al3 2008	63	300	0.21	[0.17; 0.26]	0.2%	0.5%
Nappi, et al4 2008	48	301	0.16	[0.12; 0.21]	0.2%	0.5%
Nappi, et al5 2008	90	301	0.30	[0.25; 0.35]	0.2%	0.5%
Nappi, et al6 2008	42	301	0.14	[0.10; 0.18]	0.2%	0.5%
Gotmar, et al 2008	2010	6908	0.29	[0.28; 0.30]	4.1%	0.6%
G..recka, et al 2022	129	349	0.37	[0.32; 0.42]	0.2%	0.5%
Saied, et al 2021	109	342	0.32	[0.27; 0.37]	0.2%	0.5%
Kang, et al 2021	97	150	0.65	[0.56; 0.72]	0.1%	0.5%
Senthilvel, et al 2018	46	150	0.31	[0.23; 0.39]	0.1%	0.5%
Karmakar, et al 2017	26	100	0.26	[0.18; 0.36]	0.1%	0.5%
Ganapathy, et al 2018	42	140	0.30	[0.23; 0.38]	0.1%	0.5%
Tan, et al 2014	101	309	0.33	[0.27; 0.38]	0.2%	0.5%
Zhao, et al 2023	666	1829	0.36	[0.34; 0.39]	1.1%	0.6%
Thakur, et al 2022	155	400	0.39	[0.34; 0.44]	0.2%	0.5%
Ko?o?lu, et al 2022	150	383	0.39	[0.34; 0.44]	0.2%	0.5%
AlDughaither, et al 2015	37	119	0.31	[0.23; 0.40]	0.1%	0.5%
Ceylan, et al 2014	238	874	0.27	[0.24; 0.30]	0.5%	0.6%
Kim, et al 2017	378	743	0.51	[0.47; 0.55]	0.4%	0.6%
Silvestrin, et al 2016	55	110	0.50	[0.40; 0.60]	0.1%	0.5%
Ghazanfarpour, et al 2016	53	202	0.26	[0.20; 0.33]	0.1%	0.5%
Duffy, et al 2012	1204	4264	0.28	[0.27; 0.30]	2.6%	0.6%
Loutfy, et al 2006	392	450	0.87	[0.84; 0.90]	0.3%	0.5%
Delavar, et al 2011	408	1397	0.29	[0.27; 0.32]	0.8%	0.6%
Chedraui, et al 2007	153	206	0.74	[0.68; 0.80]	0.1%	0.5%
Chedraui, et al 2014	302	451	0.67	[0.62; 0.71]	0.3%	0.5%
Rahman, et al 2011	183	509	0.36	[0.32; 0.40]	0.3%	0.5%
Aguilar−Zavala, et al 2012	168	290	0.58	[0.52; 0.64]	0.2%	0.5%
Agwu, et al 2008	65	186	0.35	[0.28; 0.42]	0.1%	0.5%
Bener, et al 2014	296	1158	0.26	[0.23; 0.28]	0.7%	0.6%
Bener, et al 2000	107	390	0.27	[0.23; 0.32]	0.2%	0.5%
Im, et al 2010	186	512	0.36	[0.32; 0.41]	0.3%	0.5%
Khatoon, et al 2018	48	121	0.40	[0.31; 0.49]	0.1%	0.5%
Budako?lu, et al 2007	181	338	0.54	[0.48; 0.59]	0.2%	0.5%
Sharanya Shre, et al 2016	4	150	0.03	[0.01; 0.07]	0.1%	0.5%
Damodaran, et al 2000	110	164	0.67	[0.59; 0.74]	0.1%	0.5%
Beura, et al 2020	46	104	0.44	[0.34; 0.54]	0.1%	0.5%
Thapa, et al 2020	66	177	0.37	[0.30; 0.45]	0.1%	0.5%
Ibrahim, et al 2015	413	1214	0.34	[0.31; 0.37]	0.7%	0.6%
Liu, et al 2007	146	267	0.55	[0.48; 0.61]	0.2%	0.5%
Lee, et al 2020	10228	20882	0.49	[0.48; 0.50]	12.5%	0.6%
Modoodi, et al 2020	102	350	0.29	[0.24; 0.34]	0.2%	0.5%
Kalhan, et al 2020	57	400	0.14	[0.11; 0.18]	0.2%	0.5%
Rindner, et al 2017	51	108	0.47	[0.38; 0.57]	0.1%	0.5%
Al−Musa, et al 2017	140	228	0.61	[0.55; 0.68]	0.1%	0.5%
Bairy, et al 2009	148	352	0.42	[0.37; 0.47]	0.2%	0.5%
Heinemann, et al1 2008	1586	4791	0.33	[0.32; 0.34]	2.9%	0.6%
Heinemann, et al2 2008	543	1500	0.36	[0.34; 0.39]	0.9%	0.6%
Heinemann, et al3 2008	637	3006	0.21	[0.20; 0.23]	1.8%	0.6%
Heinemann, et al4 2008	177	1000	0.18	[0.15; 0.20]	0.6%	0.6%
Lawton, et al 2008	1263	3616	0.35	[0.33; 0.37]	2.2%	0.6%
Liu, et al 2008	48	217	0.22	[0.17; 0.28]	0.1%	0.5%
Liu, et al 2007	103	188	0.55	[0.47; 0.62]	0.1%	0.5%
Saccomani, et al 2017	188	749	0.25	[0.22; 0.28]	0.4%	0.6%
Resmi, et al 2020	31	400	0.08	[0.05; 0.11]	0.2%	0.5%
O?urlu, et al 2011	30	132	0.23	[0.16; 0.31]	0.1%	0.5%
Mustafa, et al 2012	259	500	0.52	[0.47; 0.56]	0.3%	0.5%
Nappi, et al 2012	1584	3520	0.45	[0.43; 0.47]	2.1%	0.6%
Mohamed, et al 2014	38	90	0.42	[0.32; 0.53]	0.1%	0.5%
Hashemzadeh, et al 2019	96	302	0.32	[0.27; 0.37]	0.2%	0.5%
Kong, et al 2019	74	120	0.62	[0.52; 0.70]	0.1%	0.5%
Sharifi, et al 2019	594	600	0.99	[0.98; 1.00]	0.4%	0.6%
Holm, et al 2000	75	214	0.35	[0.29; 0.42]	0.1%	0.5%
Kasuga, et al 2004	371	1069	0.35	[0.32; 0.38]	0.6%	0.6%
Gupta, et al1 2006	38	103	0.37	[0.28; 0.47]	0.1%	0.5%
Gupta, et al2 2006	4	50	0.08	[0.02; 0.19]	0.0%	0.5%
Inayat, et al 2017	56	308	0.18	[0.14; 0.23]	0.2%	0.5%
Rahman, et al 2020	244	390	0.63	[0.58; 0.67]	0.2%	0.5%
Abedzadeh−Kalahroudi, et al 2012	516	700	0.74	[0.70; 0.77]	0.4%	0.6%
Diyu, et al 2022	222	318	0.70	[0.64; 0.75]	0.2%	0.5%
Ryu, et al 2020	1388	2481	0.56	[0.54; 0.58]	1.5%	0.6%
Liu, et al 2018	807	3382	0.24	[0.22; 0.25]	2.0%	0.6%
Joshi, et al 2015	485	1000	0.48	[0.45; 0.52]	0.6%	0.6%
Ghimire, et al 2015	521	833	0.63	[0.59; 0.66]	0.5%	0.6%
Joseph, et al 2014	46	110	0.42	[0.32; 0.52]	0.1%	0.5%
Dasgupta, et al 2015	265	1400	0.19	[0.17; 0.21]	0.8%	0.6%
Sweed, et al 2012	85	400	0.21	[0.17; 0.26]	0.2%	0.5%
Taher, et al 2012	21	86	0.24	[0.16; 0.35]	0.1%	0.5%
G K, et al 2013	54	500	0.11	[0.08; 0.14]	0.3%	0.5%
Chedraui, et al 2007	54	300	0.18	[0.14; 0.23]	0.2%	0.5%
Geng, et al 2018	227	1294	0.18	[0.16; 0.20]	0.8%	0.6%
Moral, et al 2018	401	430	0.93	[0.90; 0.95]	0.3%	0.5%
Pandey, et al 2020	79	189	0.42	[0.35; 0.49]	0.1%	0.5%
Sievert, et al 2007	174	869	0.20	[0.17; 0.23]	0.5%	0.6%
Obermeyer, et al 2004	79	293	0.27	[0.22; 0.32]	0.2%	0.5%
Keenan, et al 2003	846	2602	0.33	[0.31; 0.34]	1.6%	0.6%
Obermeyer, et al 2002	45	299	0.15	[0.11; 0.20]	0.2%	0.5%
Loh, et al 2005	165	656	0.25	[0.22; 0.29]	0.4%	0.6%
Chaopotong, et al 2005	92	148	0.62	[0.54; 0.70]	0.1%	0.5%
Huang, et al1 2010	92	300	0.31	[0.25; 0.36]	0.2%	0.5%
Huang, et al2 2010	51	100	0.51	[0.41; 0.61]	0.1%	0.5%
Huang, et al3 2010	81	300	0.27	[0.22; 0.32]	0.2%	0.5%
Huang, et al4 2010	70	150	0.47	[0.38; 0.55]	0.1%	0.5%
Huang, et al5 2010	67	150	0.45	[0.37; 0.53]	0.1%	0.5%
Fagulha, et al 2011	229	728	0.31	[0.28; 0.35]	0.4%	0.6%
Masjoudl, et al 2017	226	645	0.35	[0.31; 0.39]	0.4%	0.6%
Durairaj, et al 2022	40	74	0.54	[0.42; 0.66]	0.0%	0.5%
Abou−Raya, et al 2016	204	540	0.38	[0.34; 0.42]	0.3%	0.5%
Krajewska−Ferishah, et al1 2022	125	241	0.52	[0.45; 0.58]	0.1%	0.5%
Krajewska−Ferishah, et al2 2022	54	79	0.68	[0.57; 0.78]	0.0%	0.5%
Krajewska−Ferishah, et al3 2022	26	119	0.22	[0.15; 0.30]	0.1%	0.5%
Krajewska−Ferishah, et al4 2022	56	100	0.56	[0.46; 0.66]	0.1%	0.5%
Castelo−Branco, et al 2005	51	125	0.41	[0.32; 0.50]	0.1%	0.5%
Jin, et al 2014	232	1215	0.19	[0.17; 0.21]	0.7%	0.6%
Pe_rez−L.pez, et al 2013	626	1739	0.36	[0.34; 0.38]	1.0%	0.6%
Dasgupta, et al 2012	77	480	0.16	[0.13; 0.20]	0.3%	0.5%
Aydin, et al 2013	358	1071	0.33	[0.31; 0.36]	0.6%	0.6%
Abdullah, et al 2016	104	258	0.40	[0.34; 0.47]	0.2%	0.5%
Hickey, et al 2017	318	1136	0.28	[0.25; 0.31]	0.7%	0.6%
Blell, et al 2014	46	253	0.18	[0.14; 0.23]	0.2%	0.5%
Dienye, et al 2013	89	385	0.23	[0.19; 0.28]	0.2%	0.5%
Whiteley, et al 2013	1476	8811	0.17	[0.16; 0.18]	5.3%	0.6%
Olowokere, et al 2020	176	271	0.65	[0.59; 0.71]	0.2%	0.5%
Zhu, et al 2021	1607	2400	0.67	[0.65; 0.69]	1.4%	0.6%
Ruan, et al 2017	696	1225	0.57	[0.54; 0.60]	0.7%	0.6%
Devi, et al 2005	33	151	0.22	[0.16; 0.29]	0.1%	0.5%
Xu, et al 2005	50	237	0.21	[0.16; 0.27]	0.1%	0.5%
Obermeyer, et al 2007	35	271	0.13	[0.09; 0.18]	0.2%	0.5%
Chae, et al 2014	280	570	0.49	[0.45; 0.53]	0.3%	0.5%
Oppermann, et al 2012	53	211	0.25	[0.19; 0.32]	0.1%	0.5%
Kamal, et al 2017	365	1100	0.33	[0.30; 0.36]	0.7%	0.6%
Rajbhandari, et al 2017	160	2000	0.08	[0.07; 0.09]	1.2%	0.6%
Cortes, et al 2023	105	358	0.29	[0.25; 0.34]	0.2%	0.5%
Chalise, et al 2022	30	180	0.17	[0.12; 0.23]	0.1%	0.5%
**Common effect model**		**167055**	**0.33**	**[0.33; 0.34]**	**100.0%**	**---**
**Random effects model**			**0.37**	**[0.34; 0.40]**	**---**	**100.0%**

Heterogeneity: I^2 = 99%, τ^2 = 0.0469, p = 0

Supplementary Figure 13. Forest plot of prevalence of urinary problems among middle-aged women

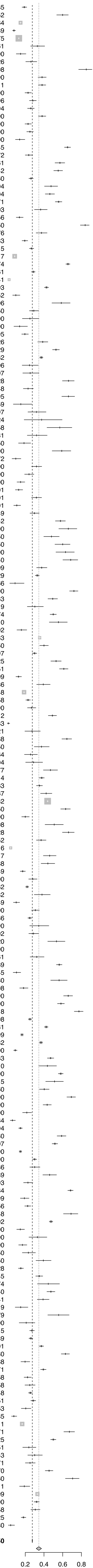

Study	Events		Proportion	95%−CI	Weight (common)	Weight (random)
Lan, et al 2017	187	985	0.19	[0.17; 0.22]	0.5%	0.5%
Adhikari, et al 2022	151	252	0.60	[0.54; 0.66]	0.1%	0.5%
Wang, et al 2021	952	6364	0.15	[0.14; 0.16]	3.2%	0.5%
Olaolorun, et al 2009	94	1189	0.08	[0.06; 0.10]	0.6%	0.5%
Li, et al 2012	2661	20275	0.13	[0.13; 0.14]	10.1%	0.5%
Javadivala, et al 2020	53	161	0.33	[0.26; 0.41]	0.1%	0.5%
Nagaraj, et al 2021	30	200	0.15	[0.10; 0.21]	0.1%	0.5%
Yisma, et al 2017	59	226	0.26	[0.21; 0.32]	0.1%	0.5%
Korkmaz, et al 2022	92	108	0.85	[0.77; 0.91]	0.1%	0.5%
AlQuaiz, et al 2013	186	490	0.38	[0.34; 0.42]	0.2%	0.5%
Genazzani, et al1 2006	232	611	0.38	[0.34; 0.42]	0.3%	0.5%
Genazzani, et al2 2006	138	600	0.23	[0.20; 0.27]	0.3%	0.5%
Genazzani, et al3 2006	167	596	0.28	[0.24; 0.32]	0.3%	0.5%
Genazzani, et al4 2006	154	594	0.26	[0.22; 0.30]	0.3%	0.5%
Genazzani, et al5 2006	228	600	0.38	[0.34; 0.42]	0.3%	0.5%
Genazzani, et al6 2006	138	600	0.23	[0.20; 0.27]	0.3%	0.5%
Genazzani, et al7 2006	150	600	0.25	[0.22; 0.29]	0.3%	0.5%
Aaron, et al 2002	28	200	0.14	[0.10; 0.20]	0.1%	0.5%
Abdel−Salam, et al 2021	551	845	0.65	[0.62; 0.68]	0.4%	0.5%
Shafie, et al 2011	112	472	0.24	[0.20; 0.28]	0.2%	0.5%
Aloufi, et al 2022	206	361	0.57	[0.52; 0.62]	0.2%	0.5%
Chou, et al 2014	244	442	0.55	[0.50; 0.60]	0.2%	0.5%
Fuh, et al 2003	356	1360	0.26	[0.24; 0.29]	0.7%	0.5%
Chedraui, et al 2014	97	204	0.48	[0.41; 0.55]	0.1%	0.5%
Chedraui, et al 2010	187	404	0.46	[0.41; 0.51]	0.2%	0.5%
Ojeda, et al 2014	429	771	0.56	[0.52; 0.59]	0.4%	0.5%
Sharma, et al 2021	74	203	0.36	[0.30; 0.43]	0.1%	0.5%
Rahman, et al 2010	49	356	0.14	[0.10; 0.18]	0.2%	0.5%
Som, et al 2012	210	250	0.84	[0.79; 0.88]	0.1%	0.5%
Alwi, et al 2009	103	276	0.37	[0.32; 0.43]	0.1%	0.5%
Waidyasekera, et al 2009	133	683	0.19	[0.17; 0.23]	0.3%	0.5%
Silva, et al 2013	378	1415	0.27	[0.24; 0.29]	0.7%	0.5%
Yang, et al 2008	836	9617	0.09	[0.08; 0.09]	4.8%	0.5%
Yim, et al 2015	1164	1774	0.66	[0.63; 0.68]	0.9%	0.5%
Yokota, et al 2016	559	1961	0.29	[0.27; 0.31]	1.0%	0.5%
Du, et al 2020	86	3141	0.03	[0.02; 0.03]	1.6%	0.5%
Buhling, et al 2014	808	1893	0.43	[0.40; 0.45]	0.9%	0.5%
Singh, et al 2014	25	252	0.10	[0.07; 0.14]	0.1%	0.5%
Borker, et al 2013	62	106	0.58	[0.49; 0.68]	0.1%	0.5%
Rathnayake, et al 2019	102	350	0.29	[0.24; 0.34]	0.2%	0.5%
Pateliya, et al 2022	25	100	0.25	[0.17; 0.35]	0.1%	0.5%
Srinivasan, et al 2021	14	100	0.14	[0.08; 0.22]	0.1%	0.5%
Chim, et al 2002	97	495	0.20	[0.16; 0.23]	0.2%	0.5%
Dhillon,et al 2006	126	326	0.39	[0.33; 0.44]	0.2%	0.5%
Chuni, et al 2011	385	729	0.53	[0.49; 0.56]	0.4%	0.5%
Nisar, et al 2015	1138	3062	0.37	[0.35; 0.39]	1.5%	0.5%
Zhang, et al 2016	26	106	0.25	[0.17; 0.34]	0.1%	0.5%
Agaba, et al 2017	27	107	0.25	[0.17; 0.35]	0.1%	0.5%
AlQuaiz, et al 2017	151	228	0.66	[0.60; 0.72]	0.1%	0.5%
Haines, et al1 2005	57	248	0.23	[0.18; 0.29]	0.1%	0.5%
Haines, et al2 2005	129	195	0.66	[0.59; 0.73]	0.1%	0.5%
Haines, et al3 2005	9	59	0.15	[0.07; 0.27]	0.0%	0.5%
Haines, et al4 2005	31	97	0.32	[0.23; 0.42]	0.0%	0.5%
Haines, et al5 2005	9	24	0.38	[0.19; 0.59]	0.0%	0.4%
Haines, et al6 2005	33	58	0.57	[0.43; 0.70]	0.0%	0.5%
Haines, et al7 2005	26	81	0.32	[0.22; 0.43]	0.0%	0.5%
Haines, et al8 2005	28	150	0.19	[0.13; 0.26]	0.1%	0.5%
Haines, et al9 2005	59	100	0.59	[0.49; 0.69]	0.1%	0.5%
Dennerstein, et al 2000	17	172	0.10	[0.06; 0.15]	0.1%	0.5%
Nappi, et al1 2008	96	300	0.32	[0.27; 0.38]	0.1%	0.5%
Nappi, et al2 2008	72	300	0.24	[0.19; 0.29]	0.1%	0.5%
Nappi, et al3 2008	45	300	0.15	[0.11; 0.20]	0.1%	0.5%
Nappi, et al4 2008	39	301	0.13	[0.09; 0.17]	0.2%	0.5%
Nappi, et al5 2008	96	301	0.32	[0.27; 0.37]	0.2%	0.5%
Nappi, et al6 2008	33	301	0.11	[0.08; 0.15]	0.2%	0.5%
G..recka, et al 2022	104	349	0.30	[0.25; 0.35]	0.2%	0.5%
Saied, et al 2021	197	342	0.58	[0.52; 0.63]	0.2%	0.5%
Sheereen, et al 2022	66	100	0.66	[0.56; 0.75]	0.1%	0.5%
Kang, et al 2021	72	150	0.48	[0.40; 0.56]	0.1%	0.5%
Senthilvel, et al 2018	90	150	0.60	[0.52; 0.68]	0.1%	0.5%
Karmakar, et al 2017	63	100	0.63	[0.53; 0.72]	0.1%	0.5%
Ganapathy, et al 2018	96	140	0.69	[0.60; 0.76]	0.1%	0.5%
Tan, et al 2014	116	309	0.38	[0.32; 0.43]	0.2%	0.5%
Zhao, et al 2023	600	1829	0.33	[0.31; 0.35]	0.9%	0.5%
Han, et al 2023	6	66	0.09	[0.03; 0.19]	0.0%	0.5%
Thakur, et al 2022	288	400	0.72	[0.67; 0.76]	0.2%	0.5%
Ko?o?lu, et al 2022	189	383	0.49	[0.44; 0.54]	0.2%	0.5%
AlDughaither, et al 2015	36	119	0.30	[0.22; 0.39]	0.1%	0.5%
Ceylan, et al 2014	436	874	0.50	[0.47; 0.53]	0.4%	0.5%
Silvestrin, et al 2016	61	110	0.55	[0.46; 0.65]	0.1%	0.5%
Ghazanfarpour, et al 2016	32	202	0.16	[0.11; 0.22]	0.1%	0.5%
Duffy, et al 2012	1515	4283	0.35	[0.34; 0.37]	2.1%	0.5%
Loutfy, et al 2006	180	450	0.40	[0.35; 0.45]	0.2%	0.5%
Delavar, et al 2011	418	1397	0.30	[0.28; 0.32]	0.7%	0.5%
Chedraui, et al 2007	172	325	0.53	[0.47; 0.58]	0.2%	0.5%
Chedraui, et al 2014	276	451	0.61	[0.57; 0.66]	0.2%	0.5%
Rahman, et al 2011	65	509	0.13	[0.10; 0.16]	0.3%	0.5%
Agwu, et al 2008	73	186	0.39	[0.32; 0.47]	0.1%	0.5%
Berecki−Gisolf, et al 2009	1619	8648	0.19	[0.18; 0.20]	4.3%	0.5%
Bener, et al 2014	266	1158	0.23	[0.21; 0.26]	0.6%	0.5%
Bener, et al 2000	104	390	0.27	[0.22; 0.31]	0.2%	0.5%
Im, et al 2010	251	512	0.49	[0.45; 0.53]	0.3%	0.5%
Ande, et al 2011	10	533	0.02	[0.01; 0.03]	0.3%	0.5%
Khatoon, et al 2018	33	121	0.27	[0.20; 0.36]	0.1%	0.5%
Budako?lu, et al 2007	218	338	0.64	[0.59; 0.70]	0.2%	0.5%
Sharanya Shre, et al 2016	56	150	0.37	[0.30; 0.46]	0.1%	0.5%
Damodaran, et al 2000	42	164	0.26	[0.19; 0.33]	0.1%	0.5%
Beura, et al 2020	30	104	0.29	[0.20; 0.39]	0.1%	0.5%
Thapa, et al 2020	83	177	0.47	[0.39; 0.55]	0.1%	0.5%
Ibrahim, et al 2015	456	1214	0.38	[0.35; 0.40]	0.6%	0.5%
Nusrat, et al 2008	303	863	0.35	[0.32; 0.38]	0.4%	0.5%
Liu, et al 2007	113	267	0.42	[0.36; 0.48]	0.1%	0.5%
Lee, et al 2020	9169	20882	0.44	[0.43; 0.45]	10.4%	0.5%
Modoodi, et al 2020	221	350	0.63	[0.58; 0.68]	0.2%	0.5%
Kalhan, et al 2020	80	400	0.20	[0.16; 0.24]	0.2%	0.5%
Rindner, et al 2017	55	108	0.51	[0.41; 0.61]	0.1%	0.5%
Al−Musa, et al 2017	151	228	0.66	[0.60; 0.72]	0.1%	0.5%
Bairy, et al 2009	130	352	0.37	[0.32; 0.42]	0.2%	0.5%
Lawton, et al 2008	160	3616	0.04	[0.04; 0.05]	1.8%	0.5%
Liu, et al 2008	100	217	0.46	[0.39; 0.53]	0.1%	0.5%
Liu, et al 2007	83	188	0.44	[0.37; 0.52]	0.1%	0.5%
Saccomani, et al 2017	129	749	0.17	[0.15; 0.20]	0.4%	0.5%
Resmi, et al 2020	112	400	0.28	[0.24; 0.33]	0.2%	0.5%
Ashrafi, et al 2009	542	2462	0.22	[0.20; 0.24]	1.2%	0.5%
O?urlu, et al 2011	50	132	0.38	[0.30; 0.47]	0.1%	0.5%
Anolue, et al 2012	36	349	0.10	[0.07; 0.14]	0.2%	0.5%
Mustafa, et al 2012	154	500	0.31	[0.27; 0.35]	0.2%	0.5%
Liu, et al 2013	418	1686	0.25	[0.23; 0.27]	0.8%	0.5%
Mohamed, et al 2014	31	90	0.34	[0.25; 0.45]	0.0%	0.5%
Hashemzadeh, et al 2019	87	302	0.29	[0.24; 0.34]	0.2%	0.5%
Kong, et al 2019	64	120	0.53	[0.44; 0.62]	0.1%	0.5%
Sharifi, et al 2019	593	600	0.99	[0.98; 1.00]	0.3%	0.5%
Pan, et al 2002	52	161	0.32	[0.25; 0.40]	0.1%	0.5%
Kasuga, et al 2004	602	1069	0.56	[0.53; 0.59]	0.5%	0.5%
Kaur, et al 2004	26	245	0.11	[0.07; 0.15]	0.1%	0.5%
Yahya, et al 2002	73	130	0.56	[0.47; 0.65]	0.1%	0.5%
Inayat, et al 2017	56	308	0.18	[0.14; 0.23]	0.2%	0.5%
Rahman, et al 2020	257	390	0.66	[0.61; 0.71]	0.2%	0.5%
Abedzadeh−Kalahroudi, et al 2012	407	700	0.58	[0.54; 0.62]	0.3%	0.5%
Diyu, et al 2022	246	318	0.77	[0.72; 0.82]	0.2%	0.5%
Zhang, et al 2016	577	2308	0.25	[0.23; 0.27]	1.2%	0.5%
Ryu, et al 2020	1052	2481	0.42	[0.40; 0.44]	1.2%	0.5%
Fu, et al 2020	528	3199	0.17	[0.15; 0.18]	1.6%	0.5%
Liu, et al 2018	1245	3382	0.37	[0.35; 0.38]	1.7%	0.5%
Joshi, et al 2015	93	1000	0.09	[0.08; 0.11]	0.5%	0.5%
Ghimire, et al 2015	392	833	0.47	[0.44; 0.51]	0.4%	0.5%
Joseph, et al 2014	48	110	0.44	[0.34; 0.53]	0.1%	0.5%
Dasgupta, et al 2015	811	1400	0.58	[0.55; 0.61]	0.7%	0.5%
ROKHADE, et al 2013	59	115	0.51	[0.42; 0.61]	0.1%	0.5%
Santo, et al 2023	145	360	0.40	[0.35; 0.46]	0.2%	0.5%
Sweed, et al 2012	277	400	0.69	[0.64; 0.74]	0.2%	0.5%
G K, et al 2013	217	500	0.43	[0.39; 0.48]	0.2%	0.5%
Chedraui, et al 2007	65	300	0.22	[0.17; 0.27]	0.1%	0.5%
Fabbrini, et al 2015	21	334	0.06	[0.04; 0.09]	0.2%	0.5%
Geng, et al 2018	192	1294	0.15	[0.13; 0.17]	0.6%	0.5%
Moral, et al 2018	253	430	0.59	[0.54; 0.64]	0.2%	0.5%
Ahmed, et al 2013	572	1107	0.52	[0.49; 0.55]	0.6%	0.5%
Xu, et al 2020	414	2790	0.15	[0.14; 0.16]	1.4%	0.5%
Islam, et al 2018	476	1590	0.30	[0.28; 0.32]	0.8%	0.5%
Yagmur, et al 2021	86	286	0.30	[0.25; 0.36]	0.1%	0.5%
Pandey, et al 2020	87	189	0.46	[0.39; 0.53]	0.1%	0.5%
Obermeyer, et al 2004	67	293	0.23	[0.18; 0.28]	0.1%	0.5%
Biri, et al 2005	707	1034	0.68	[0.65; 0.71]	0.5%	0.5%
Obermeyer, et al 2002	57	299	0.19	[0.15; 0.24]	0.1%	0.5%
Loh, et al 2005	148	656	0.23	[0.19; 0.26]	0.3%	0.5%
Chaopotong, et al 2005	102	148	0.69	[0.61; 0.76]	0.1%	0.5%
Zhu, et al 2022	1284	2702	0.48	[0.46; 0.49]	1.3%	0.5%
Huang, et al1 2010	44	300	0.15	[0.11; 0.19]	0.1%	0.5%
Huang, et al2 2010	33	100	0.33	[0.24; 0.43]	0.1%	0.5%
Huang, et al3 2010	51	300	0.17	[0.13; 0.22]	0.1%	0.5%
Huang, et al4 2010	35	150	0.23	[0.17; 0.31]	0.1%	0.5%
Huang, et al5 2010	59	150	0.39	[0.31; 0.48]	0.1%	0.5%
Fagulha, et al 2011	111	728	0.15	[0.13; 0.18]	0.4%	0.5%
Masjoudi, et al 2017	225	645	0.35	[0.31; 0.39]	0.3%	0.5%
Durairaj, et al 2022	33	74	0.45	[0.33; 0.57]	0.0%	0.5%
Abou−Raya, et al 2016	256	540	0.47	[0.43; 0.52]	0.3%	0.5%
Krajewska−Ferishah, et al1 2022	94	241	0.39	[0.33; 0.45]	0.1%	0.5%
Krajewska−Ferishah, et al2 2022	18	119	0.15	[0.09; 0.23]	0.1%	0.5%
Krajewska−Ferishah, et al3 2022	44	79	0.56	[0.44; 0.67]	0.0%	0.5%
Krajewska−Ferishah, et al4 2022	21	100	0.21	[0.13; 0.30]	0.1%	0.5%
Jin, et al 2014	330	1215	0.27	[0.25; 0.30]	0.6%	0.5%
Pe_rez−L.pez, et al 2013	452	1739	0.26	[0.24; 0.28]	0.9%	0.5%
Huang, et al 2020	558	1501	0.37	[0.35; 0.40]	0.7%	0.5%
Dasgupta, et al 2012	302	480	0.63	[0.58; 0.67]	0.2%	0.5%
Ornat, et al 2014	57	288	0.20	[0.15; 0.25]	0.1%	0.5%
Aydin, et al 2013	421	1071	0.39	[0.36; 0.42]	0.5%	0.5%
Wu, et al 2023	105	468	0.22	[0.19; 0.26]	0.2%	0.5%
Abdullah, et al 2016	64	258	0.25	[0.20; 0.31]	0.1%	0.5%
Chu, et al 2022	445	1748	0.25	[0.23; 0.28]	0.9%	0.5%
Hickey, et al 2017	328	1151	0.28	[0.26; 0.31]	0.6%	0.5%
Blell, et al 2014	52	253	0.21	[0.16; 0.26]	0.1%	0.5%
Dienye, et al 2013	30	385	0.08	[0.05; 0.11]	0.2%	0.5%
Whiteley, et al 2013	1471	8811	0.17	[0.16; 0.17]	4.4%	0.5%
Olowokere, et al 2020	182	271	0.67	[0.61; 0.73]	0.1%	0.5%
Ruan, et al 2017	612	1225	0.50	[0.47; 0.53]	0.6%	0.5%
Devi, et al 2005	36	151	0.24	[0.17; 0.31]	0.1%	0.5%
Shakhatreh, et al 2006	43	143	0.30	[0.23; 0.38]	0.1%	0.5%
Obermeyer, et al 2007	68	271	0.25	[0.20; 0.31]	0.1%	0.5%
Chae, et al 2014	259	570	0.45	[0.41; 0.50]	0.3%	0.5%
Veigh, et al 2005	113	160	0.71	[0.63; 0.78]	0.1%	0.5%
Oppermann, et al 2012	40	211	0.19	[0.14; 0.25]	0.1%	0.5%
An, et al 2023	2413	7309	0.33	[0.32; 0.34]	3.6%	0.5%
Kamal, et al 2017	353	1100	0.32	[0.29; 0.35]	0.5%	0.5%
Cortes, et al 2023	110	358	0.31	[0.26; 0.36]	0.2%	0.5%
Baig, et al 2006	167	925	0.18	[0.16; 0.21]	0.5%	0.5%
Chalise, et al 2022	8	180	0.04	[0.02; 0.09]	0.1%	0.5%
**Common effect model**		200250	**0.28**	**[0.27; 0.28]**	**100.0%**	**--**
**Random effects model**			**0.34**	**[0.32; 0.37]**	**--**	**100.0%**

Heterogeneity: $I^2 = 99\%$, $\tau^2 = 0.0435$, $p = 0$

Supplementary Figure 15. Forest plot of prevalence of difficult concentrating among middle-aged women

Study	Events	Total	Proportion	95%−CI	Weight (common)	Weight (random)
Aaron, et al 2002	9	200	0.04	[0.02; 0.08]	0.7%	1.8%
Anderson, et al1 2004	421	712	0.59	[0.55; 0.63]	2.3%	1.8%
Anderson, et al2 2004	1090	1430	0.76	[0.74; 0.78]	4.7%	1.8%
Kapur, et al 2009	36	129	0.28	[0.20; 0.36]	0.4%	1.8%
Qazi, et al 2006	396	800	0.49	[0.46; 0.53]	2.6%	1.8%
Shea, et al 2006	102	399	0.26	[0.21; 0.30]	1.3%	1.8%
Yang, et al 2008	2007	9617	0.21	[0.20; 0.22]	31.5%	1.8%
Singh, et al 2014	113	252	0.45	[0.39; 0.51]	0.8%	1.8%
Pateliya, et al 2022	16	100	0.16	[0.09; 0.25]	0.3%	1.8%
Dhillon,et al 2006	253	326	0.78	[0.73; 0.82]	1.1%	1.8%
Grigoriou, et al 2013	388	1025	0.38	[0.35; 0.41]	3.4%	1.8%
Haines, et al1 2005	121	248	0.49	[0.42; 0.55]	0.8%	1.8%
Haines, et al2 2005	133	195	0.68	[0.61; 0.75]	0.6%	1.8%
Haines, et al3 2005	29	59	0.49	[0.36; 0.63]	0.2%	1.7%
Haines, et al4 2005	72	97	0.74	[0.64; 0.83]	0.3%	1.8%
Haines, et al5 2005	14	24	0.58	[0.37; 0.78]	0.1%	1.6%
Haines, et al6 2005	33	58	0.57	[0.43; 0.70]	0.2%	1.7%
Haines, et al7 2005	40	81	0.49	[0.38; 0.61]	0.3%	1.7%
Haines, et al8 2005	99	150	0.66	[0.58; 0.74]	0.5%	1.8%
Haines, et al9 2005	97	100	0.97	[0.91; 0.99]	0.3%	1.8%
Dennerstein, et al 2000	21	172	0.12	[0.08; 0.18]	0.6%	1.8%
G..recka, et al 2022	97	349	0.28	[0.23; 0.33]	1.1%	1.8%
Thakur,et al 2019	172	351	0.49	[0.44; 0.54]	1.2%	1.8%
Sievert, et al 2022	118	162	0.73	[0.65; 0.80]	0.5%	1.8%
Loutfy, et al 2006	371	450	0.82	[0.79; 0.86]	1.5%	1.8%
Agwu, et al 2008	51	186	0.27	[0.21; 0.34]	0.6%	1.8%
Ahmed, et al 2016	113	150	0.75	[0.68; 0.82]	0.5%	1.8%
Im, et al 2010	170	512	0.33	[0.29; 0.37]	1.7%	1.8%
Resmi, et al 2020	9	400	0.02	[0.01; 0.04]	1.3%	1.8%
Mahajan,et al 2012	12	100	0.12	[0.06; 0.20]	0.3%	1.8%
Mustafa, et al 2012	269	500	0.54	[0.49; 0.58]	1.6%	1.8%
Sassoon, et al 2014	38	63	0.60	[0.47; 0.72]	0.2%	1.7%
Holm, et al 2000	123	214	0.57	[0.51; 0.64]	0.7%	1.8%
Zhang, et al 2016	36	86	0.42	[0.31; 0.53]	0.3%	1.8%
Nayak, et al 2019	87	290	0.30	[0.25; 0.36]	1.0%	1.8%
Ps, et al 2017	57	594	0.10	[0.07; 0.12]	1.9%	1.8%
Sievert, et al 2007	191	869	0.22	[0.19; 0.25]	2.9%	1.8%
Obermeyer, et al 2004	111	293	0.38	[0.32; 0.44]	1.0%	1.8%
Zhao, et al 2000	272	806	0.34	[0.30; 0.37]	2.6%	1.8%
Gollschewski, et al 2004	503	858	0.59	[0.55; 0.62]	2.8%	1.8%
Obermeyer, et al 2002	54	299	0.18	[0.14; 0.23]	1.0%	1.8%
Loh, et al 2005	190	656	0.29	[0.26; 0.33]	2.2%	1.8%
Chaopotong, et al 2005	104	148	0.70	[0.62; 0.77]	0.5%	1.8%
Anderson, et al1 2004	503	858	0.59	[0.55; 0.62]	2.8%	1.8%
Anderson, et al2 2004	511	828	0.62	[0.58; 0.65]	2.7%	1.8%
Krajewska−Ferishah, et al1 2022	152	241	0.63	[0.57; 0.69]	0.8%	1.8%
Krajewska−Ferishah, et al2 2022	56	79	0.71	[0.60; 0.81]	0.3%	1.7%
Krajewska−Ferishah, et al3 2022	48	119	0.40	[0.31; 0.50]	0.4%	1.8%
Krajewska−Ferishah, et al4 2022	42	100	0.42	[0.32; 0.52]	0.3%	1.8%
Sierra, et al 2004	335	385	0.87	[0.83; 0.90]	1.3%	1.8%
Dasgupta, et al 2012	276	480	0.57	[0.53; 0.62]	1.6%	1.8%
Blell, et al 2014	71	254	0.28	[0.23; 0.34]	0.8%	1.8%
Obermeyer, et al 2007	117	271	0.43	[0.37; 0.49]	0.9%	1.8%
Veigh, et al 2005	135	160	0.84	[0.78; 0.90]	0.5%	1.8%
Rajbhandari, et al 2017	131	2000	0.07	[0.06; 0.08]	6.6%	1.8%
Chalise, et al 2022	3	180	0.02	[0.00; 0.05]	0.6%	1.8%
**Common effect model**		**30465**	**0.35**	**[0.34; 0.35]**	**100.0%**	**--**
**Random effects model**			**0.45**	**[0.38; 0.52]**	**--**	**100.0%**

Heterogeneity: $I^2 = 99\%$, $\tau^2 = 0.0744$, $p = 0$

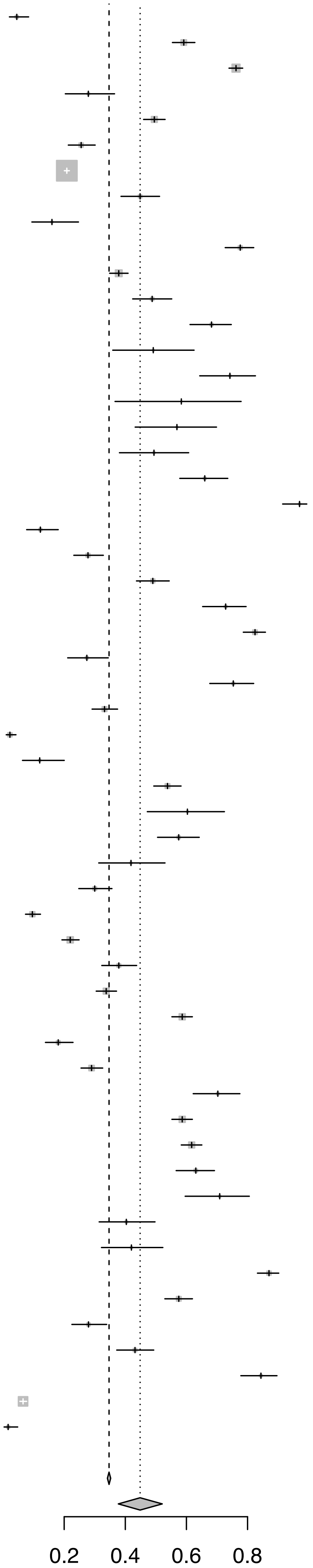

Supplementary Figure 14. Forest plot of prevalence of forgetfulness among middle-aged women

Study	Events	Total	Proportion	95%−CI	Weight (common)	Weight (random)
Aaron, et al 2002	16	200	0.08	[0.05; 0.13]	0.3%	1.4%
Fallahzadeh, et al 2007	183	346	0.53	[0.47; 0.58]	0.5%	1.4%
Shea, et al 2006	231	399	0.58	[0.53; 0.63]	0.6%	1.4%
Alwi, et al 2009	198	276	0.72	[0.66; 0.77]	0.4%	1.4%
Yim, et al 2015	1416	1774	0.80	[0.78; 0.82]	2.8%	1.4%
Yokota, et al 2016	1454	1961	0.74	[0.72; 0.76]	3.1%	1.4%
Borker, et al 2013	60	106	0.57	[0.47; 0.66]	0.2%	1.3%
Chim, et al 2002	223	495	0.45	[0.41; 0.50]	0.8%	1.4%
Haines, et al1 2005	196	248	0.79	[0.73; 0.84]	0.4%	1.4%
Haines, et al2 2005	166	195	0.85	[0.79; 0.90]	0.3%	1.4%
Haines, et al3 2005	43	59	0.73	[0.60; 0.84]	0.1%	1.3%
Haines, et al4 2005	76	97	0.78	[0.69; 0.86]	0.2%	1.3%
Haines, et al5 2005	18	24	0.75	[0.53; 0.90]	0.0%	1.2%
Haines, et al6 2005	47	58	0.81	[0.69; 0.90]	0.1%	1.3%
Haines, et al7 2005	54	81	0.67	[0.55; 0.77]	0.1%	1.3%
Haines, et al8 2005	123	150	0.82	[0.75; 0.88]	0.2%	1.4%
Haines, et al9 2005	99	100	0.99	[0.95; 1.00]	0.2%	1.3%
Sheereen, et al 2022	37	100	0.37	[0.28; 0.47]	0.2%	1.3%
Kang, et al 2021	104	150	0.69	[0.61; 0.77]	0.2%	1.4%
Senthilvel, et al 2018	104	150	0.69	[0.61; 0.77]	0.2%	1.4%
Karmakar, et al 2017	57	100	0.57	[0.47; 0.67]	0.2%	1.3%
Ganapathy, et al 2018	59	140	0.42	[0.34; 0.51]	0.2%	1.4%
Ghazanfarpour, et al 2016	65	202	0.32	[0.26; 0.39]	0.3%	1.4%
Duffy, et al 2012	1856	4274	0.43	[0.42; 0.45]	6.7%	1.4%
Chedraui, et al 2007	262	325	0.81	[0.76; 0.85]	0.5%	1.4%
Bener, et al 2014	308	1158	0.27	[0.24; 0.29]	1.8%	1.4%
Bener, et al 2000	98	390	0.25	[0.21; 0.30]	0.6%	1.4%
Im, et al 2010	295	512	0.58	[0.53; 0.62]	0.8%	1.4%
Nusrat, et al 2008	536	863	0.62	[0.59; 0.65]	1.3%	1.4%
Liu, et al 2007	202	267	0.76	[0.70; 0.81]	0.4%	1.4%
Lee, et al 2020	13967	20882	0.67	[0.66; 0.68]	32.6%	1.4%
Modoodi, et al 2020	73	350	0.21	[0.17; 0.25]	0.5%	1.4%
Bairy, et al 2009	213	352	0.61	[0.55; 0.66]	0.6%	1.4%
Liu, et al 2008	108	217	0.50	[0.43; 0.57]	0.3%	1.4%
Liu, et al 2007	138	188	0.73	[0.66; 0.80]	0.3%	1.4%
Im, et al 2009	96	158	0.61	[0.53; 0.68]	0.2%	1.4%
Resmi, et al 2020	29	400	0.07	[0.05; 0.10]	0.6%	1.4%
Ashrafi, et al 2009	733	2462	0.30	[0.28; 0.32]	3.8%	1.4%
Deveci, et al 2010	301	519	0.58	[0.54; 0.62]	0.8%	1.4%
Anolue, et al 2012	156	349	0.45	[0.39; 0.50]	0.5%	1.4%
Mahajan,et al 2012	44	100	0.44	[0.34; 0.54]	0.2%	1.3%
Mustafa, et al 2012	293	500	0.59	[0.54; 0.63]	0.8%	1.4%
Mohamed, et al 2014	29	90	0.32	[0.23; 0.43]	0.1%	1.3%
Holm, et al 2000	143	214	0.67	[0.60; 0.73]	0.3%	1.4%
Kasuga, et al 2004	865	1069	0.81	[0.78; 0.83]	1.7%	1.4%
Gupta, et al1 2006	44	103	0.43	[0.33; 0.53]	0.2%	1.3%
Gupta, et al2 2006	7	50	0.14	[0.06; 0.27]	0.1%	1.3%
Inayat, et al 2017	74	308	0.24	[0.19; 0.29]	0.5%	1.4%
Abedzadeh−Kalahroudi, et al 2012	602	700	0.86	[0.83; 0.88]	1.1%	1.4%
Zhang, et al 2016	34	86	0.40	[0.29; 0.51]	0.1%	1.3%
G K, et al 2013	316	500	0.63	[0.59; 0.67]	0.8%	1.4%
Nayak, et al 2019	90	290	0.31	[0.26; 0.37]	0.5%	1.4%
Obermeyer, et al 2004	135	293	0.46	[0.40; 0.52]	0.5%	1.4%
Keenan, et al 2003	877	2602	0.34	[0.32; 0.36]	4.1%	1.4%
Zhao, et al 2000	465	806	0.58	[0.54; 0.61]	1.3%	1.4%
Obermeyer, et al 2002	102	299	0.34	[0.29; 0.40]	0.5%	1.4%
Fagulha, et al 2011	355	728	0.49	[0.45; 0.52]	1.1%	1.4%
Krajewska−Ferishah, et al1 2022	161	241	0.67	[0.60; 0.73]	0.4%	1.4%
Krajewska−Ferishah, et al2 2022	70	79	0.89	[0.79; 0.95]	0.1%	1.3%
Krajewska−Ferishah, et al3 2022	63	119	0.53	[0.44; 0.62]	0.2%	1.4%
Krajewska−Ferishah, et al4 2022	43	100	0.43	[0.33; 0.53]	0.2%	1.3%
Dasgupta, et al 2012	341	480	0.71	[0.67; 0.75]	0.8%	1.4%
Blell, et al 2014	79	254	0.31	[0.25; 0.37]	0.4%	1.4%
Whiteley, et al 2013	2135	8811	0.24	[0.23; 0.25]	13.8%	1.4%
Olowokere, et al 2020	184	271	0.68	[0.62; 0.73]	0.4%	1.4%
Devi, et al 2005	78	151	0.52	[0.43; 0.60]	0.2%	1.4%
Xu, et al 2005	74	237	0.31	[0.25; 0.38]	0.4%	1.4%
Obermeyer, et al 2007	146	271	0.54	[0.48; 0.60]	0.4%	1.4%
Chae, et al 2014	361	570	0.63	[0.59; 0.67]	0.9%	1.4%
Veigh, et al 2005	145	160	0.91	[0.85; 0.95]	0.3%	1.4%
Oppermann, et al 2012	148	211	0.70	[0.63; 0.76]	0.3%	1.4%
Rajbhandari, et al 2017	450	2000	0.22	[0.21; 0.24]	3.1%	1.4%
Chalise, et al 2022	16	180	0.09	[0.05; 0.14]	0.3%	1.4%
**Common effect model**		**63950**	**0.52**	**[0.52; 0.53]**	**100.0%**	**--**
**Random effects model**			**0.54**	**[0.49; 0.60]**	**--**	**100.0%**

Heterogeneity: $I^2 = 99\%$, $\tau^2 = 0.0569$, $p = 0$

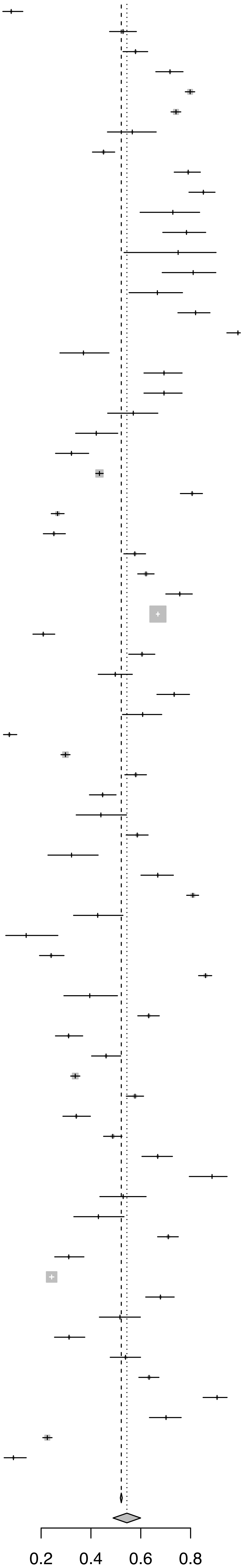

Supplementary Figure 16. Forest plot of prevalence of formication among middle-aged women

Study	Events	Total	Proportion	95%–CI	Weight (common)	Weight (random)
Lan, et al 2017	134	985	0.14	[0.12; 0.16]	1.9%	6.3%
Wang, et al 2021	664	6364	0.10	[0.10; 0.11]	12.2%	6.3%
Li, et al 2012	1543	20275	0.08	[0.07; 0.08]	38.8%	6.3%
Han, et al 2023	15	66	0.23	[0.13; 0.35]	0.1%	5.7%
Gerber, et al 2018	30	139	0.22	[0.15; 0.29]	0.3%	6.0%
Loutfy, et al 2006	340	450	0.76	[0.71; 0.79]	0.9%	6.2%
Liu, et al 2013	345	1686	0.20	[0.19; 0.22]	3.2%	6.3%
Ma, et al 2017	220	2336	0.09	[0.08; 0.11]	4.5%	6.3%
Fu, et al 2020	383	3199	0.12	[0.11; 0.13]	6.1%	6.3%
Liu, et al 2018	1515	3382	0.45	[0.43; 0.46]	6.5%	6.3%
Li, et al 2016	94	1062	0.09	[0.07; 0.11]	2.0%	6.3%
Huang, et al 2020	223	1501	0.15	[0.13; 0.17]	2.9%	6.3%
Wu, et al 2023	110	468	0.24	[0.20; 0.28]	0.9%	6.2%
Chu, et al 2022	342	1748	0.20	[0.18; 0.22]	3.3%	6.3%
Ruan, et al 2017	244	1225	0.20	[0.18; 0.22]	2.3%	6.3%
An, et al 2023	1787	7309	0.24	[0.23; 0.25]	14.0%	6.3%
**Common effect model**		**52195**	**0.14**	**[0.14; 0.14]**	**100.0%**	**--**
**Random effects model**			**0.20**	**[0.13; 0.29]**	**--**	**100.0%**

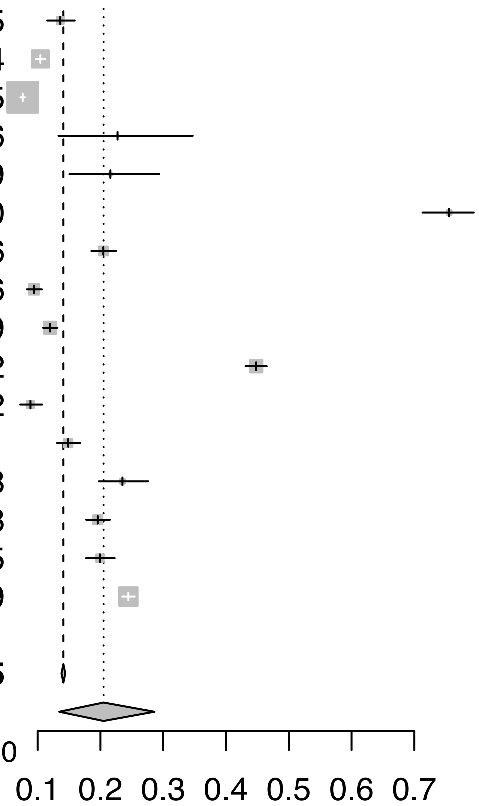

Heterogeneity: $I^2 = 100\%$, $\tau^2 = 0.0364$, $p = 0$

Supplementary Figure 17. Forest plot of prevalence of change in the appearance, texture, or tone of skin among middle-aged women

Study	Events	Total	Proportion	95%–CI	Weight (common)	Weight (random)
Yim, et al 2015	1438	1774	0.81	[0.79; 0.83]	23.1%	5.7%
Sheereen, et al 2022	64	100	0.64	[0.54; 0.73]	1.3%	5.4%
Kang, et al 2021	100	150	0.67	[0.59; 0.74]	2.0%	5.5%
Senthilvel, et al 2018	60	150	0.40	[0.32; 0.48]	2.0%	5.5%
Karmakar, et al 2017	40	100	0.40	[0.30; 0.50]	1.3%	5.4%
Ganapathy, et al 2018	105	140	0.75	[0.67; 0.82]	1.8%	5.5%
Ceylan, et al 2014	287	874	0.33	[0.30; 0.36]	11.4%	5.7%
Ghazanfarpour, et al 2016	22	202	0.11	[0.07; 0.16]	2.6%	5.5%
Chedraui, et al 2007	232	325	0.71	[0.66; 0.76]	4.2%	5.6%
Bener, et al 2014	286	1158	0.25	[0.22; 0.27]	15.1%	5.7%
Bener, et al 2000	96	390	0.25	[0.20; 0.29]	5.1%	5.6%
Liu, et al 2007	133	267	0.50	[0.44; 0.56]	3.5%	5.6%
Bairy, et al 2009	127	352	0.36	[0.31; 0.41]	4.6%	5.6%
Liu, et al 2008	64	217	0.29	[0.24; 0.36]	2.8%	5.6%
Liu, et al 2007	110	188	0.59	[0.51; 0.66]	2.5%	5.5%
Mohamed, et al 2014	31	90	0.34	[0.25; 0.45]	1.2%	5.4%
Abedzadeh–Kalahroudi, et al 2012	516	700	0.74	[0.70; 0.77]	9.1%	5.6%
G K, et al 2013	141	500	0.28	[0.24; 0.32]	6.5%	5.6%
**Common effect model**		**7677**	**0.50**	**[0.49; 0.52]**	**100.0%**	**--**
**Random effects model**			**0.46**	**[0.36; 0.57]**	**--**	**100.0%**

Heterogeneity: $I^2 = 99\%$, $\tau^2 = 0.0494$, $p = 0$

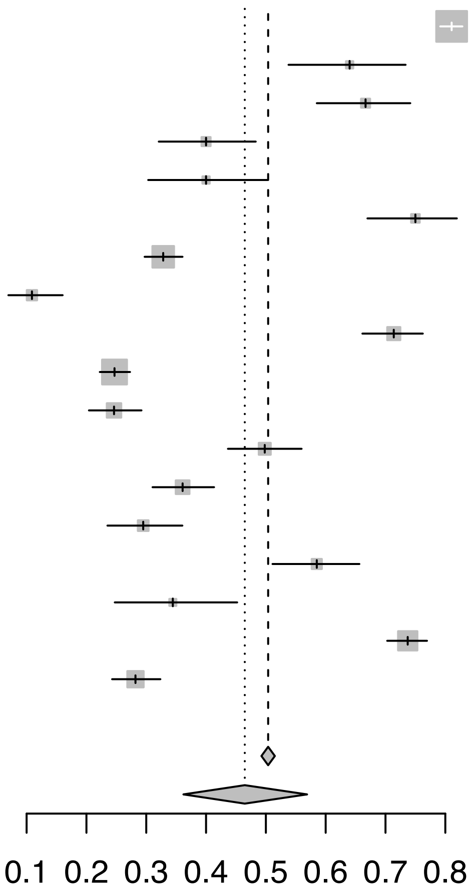

Supplementary Figure 18. Forest plot of prevalence of increased facial hair among middle-aged women

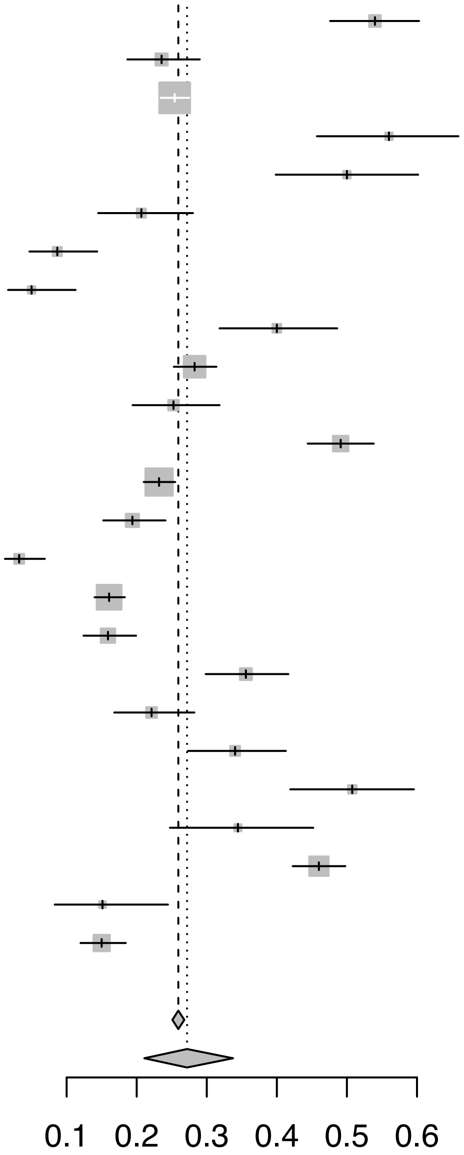

Study	Events	Total	Proportion	95%–CI	Weight (common)	Weight (random)
Som, et al 2012	135	250	0.54	[0.48; 0.60]	2.5%	4.0%
Alwi, et al 2009	65	276	0.24	[0.19; 0.29]	2.7%	4.0%
Yim, et al 2015	451	1774	0.25	[0.23; 0.28]	17.4%	4.1%
Pateliya, et al 2022	56	100	0.56	[0.46; 0.66]	1.0%	3.9%
Sheereen, et al 2022	50	100	0.50	[0.40; 0.60]	1.0%	3.9%
Kang, et al 2021	31	150	0.21	[0.14; 0.28]	1.5%	3.9%
Senthilvel, et al 2018	13	150	0.09	[0.05; 0.14]	1.5%	3.9%
Karmakar, et al 2017	5	100	0.05	[0.02; 0.11]	1.0%	3.9%
Ganapathy, et al 2018	56	140	0.40	[0.32; 0.49]	1.4%	3.9%
Ceylan, et al 2014	247	874	0.28	[0.25; 0.31]	8.6%	4.1%
Ghazanfarpour, et al 2016	51	202	0.25	[0.19; 0.32]	2.0%	4.0%
Loutfy, et al 2006	221	450	0.49	[0.44; 0.54]	4.4%	4.1%
Delavar, et al 2011	324	1397	0.23	[0.21; 0.25]	13.7%	4.1%
Chedraui, et al 2007	63	325	0.19	[0.15; 0.24]	3.2%	4.1%
Agwu, et al 2008	6	186	0.03	[0.01; 0.07]	1.8%	4.0%
Bener, et al 2014	186	1158	0.16	[0.14; 0.18]	11.3%	4.1%
Bener, et al 2000	62	390	0.16	[0.12; 0.20]	3.8%	4.1%
Liu, et al 2007	95	267	0.36	[0.30; 0.42]	2.6%	4.0%
Liu, et al 2008	48	217	0.22	[0.17; 0.28]	2.1%	4.0%
Liu, et al 2007	64	188	0.34	[0.27; 0.41]	1.8%	4.0%
O?urlu, et al 2011	67	132	0.51	[0.42; 0.60]	1.3%	3.9%
Mohamed, et al 2014	31	90	0.34	[0.25; 0.45]	0.9%	3.8%
Abedzadeh–Kalahroudi, et al 2012	322	700	0.46	[0.42; 0.50]	6.9%	4.1%
Taher, et al 2012	13	86	0.15	[0.08; 0.24]	0.8%	3.8%
G K, et al 2013	75	500	0.15	[0.12; 0.18]	4.9%	4.1%
**Common effect model**		**10202**	**0.26**	**[0.25; 0.27]**	**100.0%**	**--**
**Random effects model**			**0.27**	**[0.21; 0.34]**	**--**	**100.0%**

Heterogeneity: $I^2 = 97\%$, $\tau^2 = 0.0315$, $p < 0.01$

Supplementary Figure 19. Forest plot of prevalence of drying skin among middle-aged women

Study	Events	Total	Proportion	95%−CI	Weight (common)	Weight (random)
Kapur, et al 2009	35	129	0.27	[0.20; 0.36]	0.3%	2.5%
Som, et al 2012	151	250	0.60	[0.54; 0.67]	0.6%	2.6%
Alwi, et al 2009	159	276	0.58	[0.52; 0.64]	0.6%	2.6%
Yim, et al 2015	1487	1774	0.84	[0.82; 0.86]	3.9%	2.6%
Singh, et al 2014	31	252	0.12	[0.09; 0.17]	0.6%	2.6%
Baquedano, et al 2023	573	1059	0.54	[0.51; 0.57]	2.4%	2.6%
Chim, et al 2002	137	495	0.28	[0.24; 0.32]	1.1%	2.6%
Sheereen, et al 2022	45	100	0.45	[0.35; 0.55]	0.2%	2.5%
Kang, et al 2021	104	150	0.69	[0.61; 0.77]	0.3%	2.5%
Senthilvel, et al 2018	60	150	0.40	[0.32; 0.48]	0.3%	2.5%
Karmakar, et al 2017	40	100	0.40	[0.30; 0.50]	0.2%	2.5%
Ganapathy, et al 2018	77	140	0.55	[0.46; 0.63]	0.3%	2.5%
Ceylan, et al 2014	378	874	0.43	[0.40; 0.47]	1.9%	2.6%
Ghazanfarpour, et al 2016	42	202	0.21	[0.15; 0.27]	0.4%	2.5%
Duffy, et al 2012	872	4273	0.20	[0.19; 0.22]	9.5%	2.6%
Loutfy, et al 2006	408	450	0.91	[0.88; 0.93]	1.0%	2.6%
Delavar, et al 2011	344	1397	0.25	[0.22; 0.27]	3.1%	2.6%
Chedraui, et al 2007	278	325	0.86	[0.81; 0.89]	0.7%	2.6%
Chedraui, et al 2014	322	451	0.71	[0.67; 0.76]	1.0%	2.6%
Bener, et al 2014	338	1158	0.29	[0.27; 0.32]	2.6%	2.6%
Bener, et al 2000	113	390	0.29	[0.25; 0.34]	0.9%	2.6%
Liu, et al 2007	188	267	0.70	[0.65; 0.76]	0.6%	2.6%
Lee, et al 2020	11766	20882	0.56	[0.56; 0.57]	46.4%	2.6%
Modoodi, et al 2020	206	350	0.59	[0.54; 0.64]	0.8%	2.6%
Bairy, et al 2009	123	352	0.35	[0.30; 0.40]	0.8%	2.6%
Liu, et al 2008	75	217	0.35	[0.28; 0.41]	0.5%	2.6%
Liu, et al 2007	102	188	0.54	[0.47; 0.62]	0.4%	2.5%
O?urlu, et al 2011	50	132	0.38	[0.30; 0.47]	0.3%	2.5%
Mohamed, et al 2014	31	90	0.34	[0.25; 0.45]	0.2%	2.5%
Kasuga, et al 2004	624	1069	0.58	[0.55; 0.61]	2.4%	2.6%
Inayat, et al 2017	74	308	0.24	[0.19; 0.29]	0.7%	2.6%
Abedzadeh−Kalahroudi, et al 2012	472	700	0.67	[0.64; 0.71]	1.6%	2.6%
G K, et al 2013	136	500	0.27	[0.23; 0.31]	1.1%	2.6%
Keenan, et al 2003	305	1143	0.27	[0.24; 0.29]	2.5%	2.6%
Chaopotong, et al 2005	120	148	0.81	[0.74; 0.87]	0.3%	2.5%
Pe_rez−L.pez, et al 2013	1074	1739	0.62	[0.59; 0.64]	3.9%	2.6%
Huseth−Zosel, et al 2014	152	296	0.51	[0.45; 0.57]	0.7%	2.6%
Blell, et al 2014	66	254	0.26	[0.21; 0.32]	0.6%	2.6%
Rajbhandari, et al 2017	176	2000	0.09	[0.08; 0.10]	4.4%	2.6%
**Common effect model**		**45030**	**0.48**	**[0.47; 0.48]**	**100.0%**	**--**
**Random effects model**			**0.46**	**[0.39; 0.53]**	**--**	**100.0%**

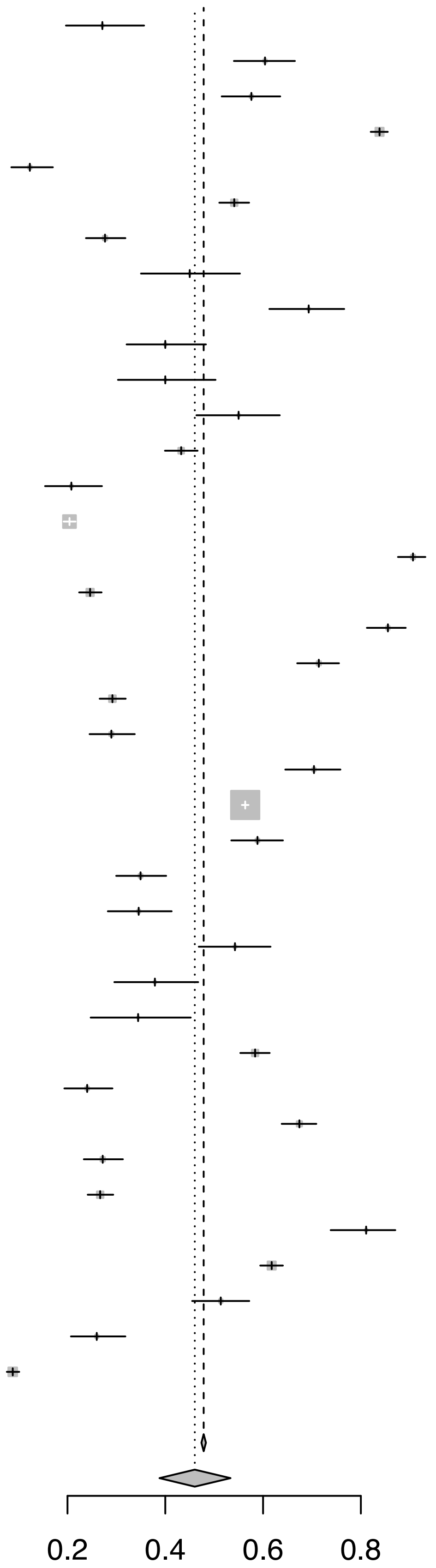

Heterogeneity: $I^2 = 100\%$, $\tau^2 = 0.0530$, $p = 0$

Supplementary Table 2. Subgroup analysis and risk factors for **Sleep problems**

Subgroup	Studies	Event	Total	Prevalence (%)	95% CI (%)	P value
**Country**						**<0.01**
China	32	38652	82789	49.65	44.69-54.61	
Nepal	7	1802	4386	48.38	28.58-68.46	
Nigeria	8	1189	3567	37.66	22.51-54.15	
Iran	10	3954	7563	60.32	44.09-75.47	
India	31	3641	7822	50.46	42.88-58.03	
Ethiopia	1	112	226	49.56	43.04-56.08	
Turkey	9	3264	5263	60.99	51.49-70.08	
Saudi	8	1858	2771	64.2	47.97-78.92	
UK	6	3914	6676	53.9	43.52-64.12	
France	2	459	900	51	47.73-54.27	
Germany	3	1778	2789	57.19	42.45-71.3	
Belgium	1	309	594	52.02	48-56.03	
Netherlands	2	495	901	52.6	37.8-67.17	
Switzerland	2	435	901	43.82	19.91-69.36	
Spain	6	6368	13888	47.9	38.51-57.36	
Australia	12	6972	13797	57.53	45.2-69.41	
Japan	5	2450	5664	43.53	28.14-59.6	
Oman	1	207	472	43.86	39.4-48.36	
Multi	5	12721	21197	59.82	51.48-67.88	
Macau, China	1	341	442	77.15	73.11-80.95	
Taiwan	6	13479	22981	52.08	44.88-59.23	
Ecuador	7	1263	2409	51.54	44.03-59.01	
Peru	1	558	771	72.37	69.16-75.48	
Pakistan	7	3806	5959	49.23	33.76-64.77	
Malaysia	8	878	1825	48.32	39.73-56.97	
Sri Lanka	2	391	1033	38.97	30.89-47.36	
Brazil	3	1071	1996	49.75	40.36-59.14	
Korea	7	12614	55672	37.66	24.12-52.26	
Singapore	3	764	2245	32.15	23.6-41.35	
Greece	1	413	1025	40.29	37.31-43.31	
the Philippines	1	153	195	78.46	72.4-83.97	
Republic of Indonesia	4	871	1622	55.54	29.89-79.73	
Thailand	3	290	448	64.93	53.56-75.52	
Vietnam	1	92	100	92	85.76-96.64	
Italy	3	662	1663	40.98	33.77-48.39	
Poland	1	216	349	61.89	56.73-66.92	
Iraq	2	457	842	56.15	37.05-74.36	
USA	10	5849	15283	50.3	42.61-57.99	
Egypt	5	2165	3704	62.88	38.87-83.93	
Bangladesh	4	772	1375	56.12	35.65-75.57	

Qatar	1	335	1158	28.93	26.35-31.58	
United Arab Emirates	1	120	390	30.77	26.28-35.45	
Cambodia	1	146	177	82.49	76.51-87.76	
Sweden	1	72	108	66.67	57.46-75.28	
New Zealand	1	1962	3616	54.26	52.63-55.88	
South Africa	1	35	63	55.56	43.1-67.68	
Libya	1	47	86	54.65	44.01-65.08	
Hong Kong	2	199	455	53.15	6.9-95.99	
Mexico	2	2819	5303	53.16	51.82-54.5	
Morocco	1	93	299	31.1	25.97-36.48	
Portugal	1	345	728	47.39	43.77-51.02	
Colombia	1	1107	1739	63.66	61.38-65.9	
Lebanon	1	141	271	52.03	46.06-57.97	
Finland	1	52	158	32.91	25.78-40.46	
**Continent**						0.89
Asia	158	91677	213920	51.97	48.84-55.1	
Africa	17	3641	7945	47.4	35.92-59.01	
Europe	32	21401	39272	51.25	47.01-55.48	
Oceania	13	8934	17413	57.27	45.94-68.24	
South America	13	7439	12994	54.2	48.48-59.86	
North America	12	8668	20586	50.71	44.35-57.06	
Multi	2	3398	6526	51.92	47.8-56.03	
**Income level**						0.66
Upper-Middle-Income	72	52279	106982	53.09	49.93-56.24	
Lower-Middle-Income	83	19216	38073	52.27	47.15-57.37	
Low-Income	1	112	226	49.56	43.04-56.08	
High-Income	90	70111	167296	50.55	46.97-54.12	
**Development status**						0.23
Developing	156	83095	164103	52.94	49.88-55.99	
Developed	89	57126	145468	50.03	46.38-53.67	
**Publication date**						**0.02**
Before 2011	105	39681	84755	48.84	45.52-52.17	
After 2011	142	105477	233901	54.13	50.94-57.3	
**Study size**						0.09
<1000	184	34125	65010	53.05	50.3-55.79	
>1000	63	111033	253646	48.6	44.26-52.95	
**Study quality**						0.16
<8	35	28295	63512	52.51	49.95-55.06	
≥8	212	11686	255144	48.15	42.64-53.69	

		3				
**Diagnosis criteria**						**<0.01**
KMI	22	34238	73366	49.66	44.17-55.15	
MRS	70	35349	58489	59.85	55.68-63.95	
Face-to-face interview	49	19390	48691	44.11	39.12-49.16	
Others	55	30419	58043	50.12	44.81-55.43	
The Greene Climacteric Scale	16	8070	15089	50.25	42.18-58.31	
SMI	2	650	2338	27.32	11.58-46.73	
MENQOL	21	4334	8339	57.71	50.73-64.54	
PSQI	8	10915	51441	38.32	29.09-48	
CS-10	2	1417	2190	65.85	60.86-70.67	
the Midlife Women's Symptom Index	2	376	670	56.13	52.35-59.88	

Risk factors	Studies	Event	Total	Prevalence (%)	95% CI (%)	P value
**Menopausal stage**						**<0.01**
Premenopause	60	15269	41415	39.35	34.3-44.52	
Perimenopause	64	15023	30263	52.41	47.3-57.49	
Postmenopause	101	38554	73260	54.91	51.23-58.57	
**Age**						0.27
<50	12	5812	14170	39.61	28.66-51.11	
≥50	26	7359	15916	47.25	39.79-54.76	
**Physical activity**						0.40
Regular	3	602	1279	49.46	35.92-63.03	
Irregular	3	316	633	65.22	31.62-92.04	
**BMI**						**0.01**
Underweight	3	58	144	40.16	32.14-48.44	
Normal weight	3	794	2322	34.79	31.91-37.72	
Overweight	4	390	1017	42.31	31.39-53.63	
Obesity	8	1198	2030	55.62	41.57-69.24	
**Urban or rural**						0.48
Rural	18	7625	14812	45.17	35.01-55.54	
Urban	7	6986	13533	53.98	32.02-75.17	
**Work**						0.57
Working	6	1326	2400	54.12	44.09-63.98	
Non-working	5	309	534	59.33	44.32-73.54	
**Current drinking habit**						0.76
Yes	2	96	246	38.92	32.83-45.18	
No	2	520	1366	38.06	35.5-40.66	
**Current smoking**						0.27
Yes	2	14	29	48.15	29.32-67.21	

No	2	604	1582	38.18	35.8-40.59	
**Marital status**						0.80
Single	3	96	289	31	20.01-43.07	
Married	4	816	3480	32.51	18.09-48.83	
Divorced or Widowed	2	84	341	57.04	0.1-100	
**Education level**						0.71
<12 years	7	2021	4864	45.55	32.75-58.65	
>12 years	6	772	2086	36.59	19.18-55.99	

Supplementary Table 3. Subgroup analysis and risk factors for **Heart discomfort**

Subgroup	Studies	Event	Total	Prevalence (%)	95% CI (%)	P value
**Country**						**<0.01**
China	23	22179	68790	38.56	32.57-44.73	
Nepal	7	1352	4386	32.25	15.98-51.09	
Nigeria	5	861	2216	40.1	22.6-59.01	
Iran	4	1105	1752	62.67	27.88-91.3	
India	18	2166	4799	42.07	34.14-50.21	
Ethiopia	1	50	226	22.12	16.94-27.78	
Turkey	8	2383	4389	51.87	38.56-65.05	
Saudi	6	1535	2271	64.2	50.52-76.81	
Australia	5	1183	3738	26.64	14.68-40.65	
Japan	5	2225	5657	38.04	21.29-56.41	
Oman	1	197	472	41.74	37.32-46.22	
Macau, China	1	259	442	58.6	53.97-63.15	
Ecuador	5	809	1744	43.72	24.41-64.06	
Peru	1	434	771	56.29	52.77-59.78	
Malaysia	4	241	802	38.04	18.15-60.23	
Sri Lanka	2	337	1033	32.62	29.79-35.51	
Brazil	3	1028	1996	49.03	43.36-54.72	
Spain	3	3171	11941	24.85	21.7-28.13	
Pakistan	4	2322	3549	45.02	26.49-64.28	
Greece	1	377	1025	36.78	33.85-39.76	
the Philippines	1	95	195	48.72	41.71-55.75	
Republic of Indonesia	2	263	377	58.36	22.76-89.62	
Korea	4	1887	3891	46.05	41.35-50.79	
Taiwan	3	10014	21124	43.94	34.55-53.55	
Thailand	1	80	150	53.33	45.3-61.28	
Vietnam	1	94	100	94	88.35-97.96	
Poland	1	127	349	36.39	31.41-41.51	
Iraq	1	216	342	63.16	57.97-68.2	
Egypt	5	1920	3704	51.81	34.21-69.19	
Bangladesh	3	483	1049	52.54	19.24-84.61	

USA	4	1375	9853	23.94	15.48-33.58	
Cambodia	1	107	177	60.45	53.13-67.55	
Sweden	1	52	108	48.15	38.75-57.61	
New Zealand	1	932	3616	25.77	24.36-27.21	
South Africa	1	13	63	20.63	11.43-31.61	
Libya	1	37	86	43.02	32.7-53.66	
Morocco	1	102	299	34.11	28.84-39.59	
Singapore	1	98	656	14.94	12.31-17.77	
Bolivia	1	15	125	12	6.82-18.34	
Colombia	1	563	1739	32.37	30.19-34.59	
UK	1	51	253	20.16	15.43-25.34	
Lebanon	1	127	271	46.86	40.94-52.83	
**Continent**						**<0.01**
Asia	102	49765	126674	44.86	40.89-48.86	
Africa	14	2983	6594	41.32	31.33-51.68	
Oceania	6	2115	7354	26.54	16.67-37.76	
South America	11	2849	6375	41.9	30.79-53.43	
Europe	7	3778	13676	29.93	23.13-37.21	
North America	4	1375	9853	23.94	15.48-33.58	
**Income level**						**<0.01**
Upper-Middle-Income	50	28242	81314	42.84	38.18-47.57	
Lower-Middle-Income	56	11349	24032	44.8	38.78-50.89	
Low-Income	1	50	226	22.12	16.94-27.78	
High-Income	37	23224	64954	37.71	31.8-43.81	
**Development status**						0.07
Developing	107	49677	126464	43.91	40.13-47.73	
Developed	37	13188	44062	37.01	30.89-43.34	
**Publication date**						**<0.01**
Before 2011	51	12036	37452	35.98	30.99-41.12	
After 2011	93	50829	133074	45.51	41.45-49.61	
**Study quality**						**0.02**
<8	26	13751	51862	34.84	28.35-41.62	
≥8	118	49114	118664	43.76	40.11-47.45	
**Study size**						0.18
<1000	109	16131	36524	43.34	39.47-47.24	
>1000	35	46734	134002	38.47	32.6-44.52	
**Diagnosis criteria**						**<0.01**
KMI	18	21989	67059	37.54	31.15-44.15	
MRS	61	16387	31546	50.42	45.27-55.56	
The Greene Climacteric Scale	13	4857	12021	39.19	31.26-47.43	

Face-to-face interview	19	4659	24381	25.44	19.38-32.02	
SMI	2	526	2338	21.12	2.94-49.67	
Others	27	13520	30755	41.39	34.3-48.66	
CS-10	2	790	2190	41.09	24.33-58.98	
MENQOL	2	137	236	55.27	32.15-77.27	
**Risk factors**	**Studies**	**Event**	**Total**	**Prevalence (%)**	**95% CI (%)**	**P value**
**Menopausal stage**						**<0.01**
Premenopause	39	9201	34571	29.72	23.86-35.93	
Perimenopause	39	8624	23436	41.83	35.54-48.24	
Postmenopause	58	20431	51148	46.43	40.98-51.92	
**Age**						0.88
<50	6	1006	2292	37.3	22.83-53	
≥50	9	2228	5974	39.04	24.62-54.49	
**Urban or rural**						0.26
Rural	10	5401	11964	37.5	26.16-49.59	
Urban	6	5168	12936	53.77	28.56-77.99	

Supplementary Table 4. Subgroup analysis and risk factors for **Headache**

Subgroup	Studies	Event	Total	Prevalence (%)	95% CI (%)	P value
**Country**						**<0.01**
China	20	25656	66251	43.17	37.41-49.02	
UK	6	2771	6661	40.03	34.92-45.25	
France	2	289	900	32.84	26.8-39.16	
Germany	2	346	896	35.91	21.23-52.08	
Belgium	1	184	594	30.98	27.32-34.76	
Netherlands	2	309	901	32.5	22.19-43.74	
Switzerland	2	273	901	27.81	15.25-42.46	
Spain	4	3997	12541	37.6	29.91-45.61	
India	15	1494	3497	40.69	32.28-49.38	
Australia	8	2974	11928	44.56	31.54-57.98	
Japan	5	2625	5664	45.84	27.62-64.65	
Iran	5	1627	2995	48.45	18.28-79.26	
Taiwan	4	9726	22703	39.29	28.16-51	
Pakistan	2	661	1108	51.38	16.31-85.68	
Singapore	2	418	1151	35.51	25.26-46.49	
Malaysia	3	266	590	45.56	24.8-67.15	
Greece	1	375	1025	36.59	33.66-39.56	
Italy	2	228	635	34.76	13.31-60.12	
Poland	1	173	349	49.57	44.33-54.82	
Korea	1	125	743	16.82	14.22-19.6	
USA	6	1040	2264	47.74	37.45-58.14	
Egypt	1	428	450	95.11	92.91-96.93	
Ecuador	2	529	710	74.34	52.2-91.36	

Bangladesh	2	551	659	78.72	52.9-96.04	
Qatar	1	570	1158	49.22	46.34-52.1	
United Arab Emirates	1	181	390	46.41	41.48-51.38	
Nigeria	3	471	1153	46.52	17.25-77.17	
Finland	1	99	1184	8.36	6.85-10.01	
New Zealand	1	1225	3616	33.88	32.34-35.43	
Turkey	3	891	1700	50.31	31.25-69.32	
Multi	1	810	3520	23.01	21.64-24.42	
South Africa	1	31	63	49.21	36.87-61.59	
Saudi	1	71	90	78.89	69.79-86.76	
Brazil	2	297	581	52.91	38.98-66.62	
Mexico	1	401	755	53.11	49.54-56.66	
Hong Kong	2	505	1128	49.58	35.57-63.63	
Morocco	1	173	299	57.86	52.21-63.41	
Thailand	2	188	298	63.52	40.82-83.46	
Bolivia	1	28	125	22.4	15.48-30.16	
Jordan	2	96	280	33.5	12.53-58.58	
Lebanon	1	141	271	52.03	46.06-57.97	
Nepal	1	44	180	24.44	18.42-31.01	
**Continent**						**<0.01**
Asia	73	45836	110856	44.95	40.63-49.3	
Europe	24	9044	26587	34.7	30.46-39.05	
Oceania	9	4199	15544	43.33	31.65-55.39	
North America	7	1441	3019	48.53	39.67-57.43	
Africa	6	1103	1965	58.85	35.16-80.57	
South America	5	854	1416	55.72	34.67-75.75	
Multi	1	810	3520	23.01	21.64-24.42	
**Income level**						**<0.05**
Upper-Middle-Income	36	28355	71228	47.3	42.16-52.46	
High-Income	57	29314	80942	39.81	35.94-43.74	
Lower-Middle-Income	32	5618	10737	47.39	38.55-56.31	
**Development status**						**0.01**
Developing	72	43801	104750	47.34	42.64-52.07	
Developed	53	19468	58157	39.3	35.27-43.41	
**Publication date**						0.19
Before 2011	50	41996	104448	46.5	41.94-51.08	
After 2011	75	21291	58459	42.17	37.68-46.72	
**Study size**						0.06
<1000	93	15735	34027	45.54	41.54-49.57	
>1000	32	47552	128880	39.35	34.24-44.57	
**Study quality**						0.38

<8	18	14181	39798	47.26	39.45-55.13	
≥8	107	49106	123109	43.35	39.76-46.97	
**Diagnosis criteria**						**<0.01**
KMI	20	26390	68179	43.23	37.98-48.55	
Face-to-face interview	43	10308	34917	37.41	31.69-43.32	
Others	31	17222	40308	43.68	37.79-49.67	
The Greene Climacteric Scale	13	5329	11477	51.26	43.91-58.59	
SMI	2	621	2338	25.69	7.03-50.92	
MRS	4	794	1149	63.61	35.73-87.3	
MENQOL	12	2623	4539	58.04	48.19-67.58	

Risk factors	Studies	Event	Total	Prevalence (%)	95% CI (%)	P value
**Menopausal stage**						0.30
Premenopause	25	10614	31407	41.43	34.06-49.01	
Perimenopause	27	9159	22405	49.13	41.97-56.32	
Postmenopause	48	19019	48500	47.73	42.02-53.47	
**Age**						
<50	6	1938	10314	32.48	22.77-43.02	
≥50	13	3055	10183	33.62	21.91-46.45	
**Urban or rural**						0.22
Rural	7	3209	7393	49.05	34.05-64.15	
Urban	4	5067	12232	35.76	21.99-50.83	

Supplementary Table 5. Subgroup analysis and risk factors for **Joint and muscular discomfort**

Subgroup	Studies	Event	Total	Prevalence (%)	95% CI (%)	P value
**Country**						**<0.01**
China	27	32966	74834	49.81	42.63-56.99	
Nepal	7	2330	4386	58.76	39.55-76.69	
Nigeria	8	2190	3567	62.99	47.44-77.28	
Iran	9	5088	6863	74.01	60.12-85.81	
India	32	5236	7667	71.53	63.88-78.61	
Ethiopia	1	73	226	32.3	26.35-38.55	
Turkey	8	2359	3712	63.86	55.81-71.54	
Saudi	7	1983	2361	86.46	79.69-92.06	
Australia	9	4531	13075	61.42	44.16-77.31	
Japan	6	5042	7628	68.24	56.44-78.96	
Oman	1	351	472	74.36	70.32-78.21	
Macau, China	1	391	442	88.46	85.31-91.28	
Taiwan	4	12739	22520	52.8	29.97-75.03	
Ecuador	6	1711	2069	82.91	79.01-86.49	
Peru	1	600	771	77.82	74.82-80.69	

Pakistan	5	3824	4904	73.95	64.51-82.4	
Malaysia	6	1065	1404	77.39	70.31-83.78	
Sri Lanka	2	800	1033	78.76	70.25-86.18	
Brazil	3	1436	1996	67.07	53.24-79.56	
Spain	3	4986	11941	51.76	37.49-65.88	
Korea	6	3486	5799	61.33	48.79-73.15	
Singapore	2	601	1151	52.22	49.32-55.1	
Greece	1	485	1025	47.32	44.27-50.38	
the Philippines	1	175	195	89.74	85.06-93.65	
Republic of Indonesia	3	1080	1377	84.78	73.65-93.32	
Thailand	2	260	298	87.37	81.47-92.29	
Vietnam	1	96	100	96	91.09-99.13	
Poland	1	191	349	54.73	49.48-59.93	
Iraq	1	318	342	92.98	90.01-95.47	
Italy	1	325	1028	31.61	28.81-34.49	
USA	10	4246	12895	55.95	45.27-66.37	
UK	4	3221	5760	49.77	41.91-57.63	
Egypt	5	3120	3704	85.08	77.68-91.21	
Bangladesh	4	1702	2639	70.82	57.27-82.72	
Qatar	1	413	1158	35.66	32.93-38.45	
United Arab Emirates	1	135	390	34.62	29.97-39.42	
Cambodia	1	141	177	79.66	73.39-85.29	
Sweden	2	639	1233	55.01	45.22-64.61	
Finland	1	265	1184	22.38	20.05-24.8	
Multi	3	6091	11317	52.62	30.25-74.44	
New Zealand	1	2317	3616	64.08	62.5-65.63	
South Africa	1	38	63	60.32	47.9-72.11	
Libya	1	64	86	74.42	64.61-83.14	
Mexico	2	2821	5303	53.73	51.11-56.33	
Hong Kong	1	553	978	56.54	53.42-59.64	
Morocco	1	161	299	53.85	48.17-59.47	
Bolivia	1	18	125	14.4	8.74-21.15	
Colombia	1	1239	1739	71.25	69.1-73.35	
Jordan	1	126	143	88.11	82.24-92.96	
Lebanon	1	119	271	43.91	38.04-49.87	
**Continent**						**<0.01**
Asia	141	83379	153244	67.37	63.83-70.81	
Africa	17	5646	7945	68.54	58.22-78.01	
Oceania	10	6848	16691	61.69	46.27-75.99	
South America	12	5004	6700	72.59	60.87-82.92	

Risk factors	Studies	Event	Total	Prevalence (%)	95% CI (%)	P value
Europe	14	13298	27311	48.84	42.04-55.67	
North America	12	7067	18198	55.63	46.81-64.27	
Multi	2	2905	6526	45.52	17.01-75.76	
**Income level**						**<0.01**
Upper-Middle-Income	62	47243	96208	63.99	58.87-68.94	
Lower-Middle-Income	81	26080	37307	71.3	66.68-75.72	
Low-Income	1	73	226	32.3	26.35-38.55	
High-Income	64	50751	102874	59.66	54.63-64.6	
**Development status**						0.08
Developing	145	83970	153093	67.08	63.55-70.51	
Developed	62	38328	80516	61.57	56.4-66.62	
**Publication date**						0.29
Before 2011	83	38792	72143	63.54	59.13-67.85	
After 2011	125	85355	164472	66.67	62.8-70.43	
**Study size**						**<0.01**
<1000	153	35354	52415	69.42	66.12-72.62	
>1000	55	88793	184200	54.16	49.33-58.95	
**Study quality**						0.21
<8	33	25927	58289	61.22	53.87-68.32	
≥8	175	98220	178326	66.21	63.04-69.32	
**Diagnosis criteria**						**<0.01**
KMI	21	31245	71320	49.15	43.22-55.09	
MRS	67	33142	46655	76.74	73.53-79.8	
The Greene Climacteric Scale	15	8800	14202	62.45	55.18-69.45	
Face-to-face interview	29	12487	38874	45.63	36.86-54.54	
SMI	2	1422	2338	61.34	32.12-86.67	
Others	45	26392	47307	63.58	57.25-69.68	
MENQOL	23	6656	10029	73.99	66.18-81.11	
The Keio questionnaire	2	2017	3030	72.02	40.51-94.64	
CS-10	2	1638	2190	80.5	61.28-94.23	
the Midlife Women's Symptom Index	2	348	670	51.94	48.14-55.73	
**Menopausal stage**						**<0.01**
Premenopause	51	15753	39037	52.83	46.43-59.2	

Perimenopause	55	14575	27650	66.01	59.78-71.98	
Postmenopause	84	37684	65103	71.16	66.99-75.15	
**Age**						**0.04**
<50	8	2190	10759	41.94	28.33-56.19	
≥50	14	5141	10763	61.53	49.74-72.68	
**Urban or rural**						0.64
Rural	18	8438	13282	67.65	56.82-77.61	
Urban	8	6990	13871	72.37	54.22-87.36	
**Work**						0.89
Working	4	1299	1954	49.08	21.41-77.05	
Non-working	3	143	410	44.64	2.81-92.36	

Supplementary Table 6. Subgroup analysis and risk factors for **Physical and mental exhaustion**

**Subgroup**	**Studies**	**Event**	**Total**	**Prevalence (%)**	**95% CI (%)**	**P value**
**Country**						**<0.01**
China	24	35550	73547	57.27	50.11-64.27	
Nepal	7	2192	4386	57.9	33.57-80.36	
Nigeria	6	1307	2834	53.36	30.86-75.17	
Iran	7	3303	5917	62.83	42.59-80.97	
India	32	5131	7763	68.67	60.77-76.08	
Ethiopia	1	79	226	34.96	28.86-41.31	
Turkey	8	2532	3712	69.84	59.07-79.61	
Saudi	7	1908	2361	79.72	75.91-83.28	
Oman	1	226	472	47.88	43.38-52.4	
Macau, China	1	399	442	90.27	87.32-92.87	
Taiwan	4	14398	22600	35.19	16.12-57.11	
Ecuador	6	1409	2069	68.16	56.6-78.69	
Peru	1	565	771	73.28	70.1-76.35	
Japan	4	2812	4234	69.94	44.12-90.34	
Pakistan	3	3250	3983	76	63.96-86.26	
Malaysia	6	1094	1701	65.77	51.89-78.4	
Sri Lanka	2	591	1033	58.67	48.92-68.08	
Brazil	3	1287	1996	67.59	59.6-75.1	
Spain	4	4107	13000	35.16	27.46-43.28	
Korea	3	3982	4998	74.45	50.7-92.29	
Germany	1	1183	1893	62.49	60.3-64.66	
Singapore	1	192	495	38.79	34.54-43.13	
Greece	1	508	1025	49.56	46.5-52.62	
Poland	1	268	349	76.79	72.21-81.08	
Iraq	2	737	842	89.05	76.61-97.18	
USA	6	1133	2264	50.8	39.97-61.58	

UK	4	4503	5781	61.69	42.17-79.44	
Egypt	5	2966	3704	82.63	72.2-91.03	
Bangladesh	3	725	1049	72.9	59.84-84.21	
Qatar	1	311	1158	26.86	24.34-29.45	
United Arab Emirates	1	104	390	26.67	22.39-31.17	
Cambodia	1	156	177	88.14	82.92-92.53	
Australia	7	2700	3701	76.5	67.02-84.82	
Sweden	1	80	109	73.39	64.66-81.31	
Finland	1	60	1184	5.07	3.89-6.39	
New Zealand	1	2412	3616	66.7	65.16-68.23	
Multi	1	1619	3520	45.99	44.35-47.64	
South Africa	1	48	63	76.19	64.81-85.99	
Republic of Indonesia	1	275	318	86.48	82.49-90.03	
Libya	1	48	86	55.81	45.18-66.2	
Mexico	2	3299	5303	65.18	56.62-73.27	
Hong Kong	1	528	978	53.99	50.86-57.1	
Morocco	1	185	299	61.87	56.29-67.31	
Thailand	1	101	148	68.24	60.49-75.52	
Colombia	1	933	1739	53.65	51.3-55.99	
Lebanon	1	214	271	78.97	73.9-83.63	
**Continent**						**<0.01**
Asia	122	80711	142975	65.24	61.33-69.06	
Africa	15	4633	7212	65.01	52.3-76.73	
South America	11	4194	6575	67.25	60.38-73.77	
Europe	13	10709	23341	49.54	36.09-63.02	
North America	8	4432	7567	54.6	45.21-63.83	
Oceania	8	5112	7317	75.28	66.69-82.98	
Multi	1	1619	3520	45.99	44.35-47.64	
**Income level**						**<0.01**
Upper-Middle-Income	57	48002	92419	64.62	60.19-68.94	
Lower-Middle-Income	69	20295	31734	67.79	62.32-73.02	
Low-Income	1	79	226	34.96	28.86-41.31	
High-Income	51	43034	74128	59.07	52.52-65.46	
**Development status**						0.22
Developing	128	82163	145950	65.39	61.67-69.01	
Developed	50	29247	52557	60.89	54.61-66.99	
**Publication date**						**<0.01**
Before 2011	60	23135	46654	56.06	50.16-61.87	

After 2011	118	88275	151853	68.11	64.53-71.58	
**Study quality**						0.30
<8	27	22931	45773	60.46	52.99-67.69	
≥8	151	88479	152734	64.78	61.25-68.23	
**Study size**						**<0.01**
<1000	131	29877	45226	67.14	63.55-70.63	
>1000	47	81533	153281	55.69	49.49-61.81	
**Diagnosis criteria**						**<0.01**
KMI	21	33960	71320	54.99	48.41-61.48	
MRS	65	26989	38088	71.64	67.78-75.35	
Others	33	28606	46040	57.59	49.99-65.02	
SMI	2	1165	2338	49.82	22-77.7	
The Greene Climacteric Scale	13	7540	12057	64.07	54.49-73.12	
Face-to-face interview	18	5831	17661	45.85	32.41-59.62	
MENQOL	22	5783	8439	73.71	64.23-82.2	
CS-10	2	1232	2190	59.9	47.3-71.87	
WHAS	2	304	374	82.97	65.81-95.05	

Risk factors	Studies	Event	Total	Prevalence (%)	95% CI (%)	P value
**Menopausal stage**						**0.02**
Premenopause	47	17115	37222	53.57	46.75-60.32	
Perimenopause	52	14104	26811	62.96	56.37-69.33	
Postmenopause	76	34045	61673	65.75	60.58-70.75	
**Age**						0.17
<50	7	997	1902	51.15	40.83-61.42	
≥50	11	4783	9016	62.79	49.85-74.86	
**Urban or rural**						0.94
Rural	15	7910	12964	66.5	53.37-78.45	
Urban	6	5925	12586	67.6	40.82-89.33	

Supplementary Table 7. Subgroup analysis and risk factors for **Anxiety**

Subgroup	Studies	Event	Total	Prevalence (%)	95% CI (%)	P value
**Country**						**<0.01**
Nepal	7	1413	4386	39.41	24-55.98	
Nigeria	6	964	3032	36.59	17.66-57.96	
Iran	6	1994	3705	60.03	33.12-84.03	
India	25	3567	6031	59.52	47.23-71.23	
Ethiopia	1	90	226	39.82	33.52-46.3	
Turkey	6	1683	3061	62.87	47.02-77.42	
Saudi	7	1629	2361	64.5	44.97-81.82	

Australia	8	2463	4415	60.7	51.71-69.34	
Japan	3	2231	3323	67.25	64.62-69.83	
Oman	1	223	472	47.25	42.75-51.76	
Multi	3	7729	13876	55.35	50.2-60.44	
Macau, China	1	308	442	69.68	65.31-73.89	
Ecuador	6	1242	2069	58.76	46.74-70.26	
Peru	1	551	771	71.47	68.22-74.6	
Malaysia	4	592	1216	50.2	32.06-68.31	
China	12	5590	26910	26.49	17.3-36.85	
Sri Lanka	2	306	1033	32.69	13.06-56.16	
Brazil	2	1183	1626	72.79	70.59-74.93	
Korea	2	2345	4255	55.15	53.42-56.87	
Singapore	2	230	1151	19.98	17.71-22.34	
Pakistan	2	2342	3183	58.55	24.83-88.29	
Iraq	2	461	842	57.82	28.9-84.09	
USA	12	5365	20259	33.72	26.87-40.93	
UK	3	2029	4643	41.79	37.4-46.25	
Egypt	5	2140	3704	61.18	43.6-77.38	
Bangladesh	3	580	1049	62.32	34.49-86.36	
Qatar	2	566	2259	23.96	6.75-47.45	
United Arab Emirates	1	102	390	26.15	21.9-30.64	
Cambodia	1	142	177	80.23	74.01-85.79	
Sweden	1	57	108	52.78	43.3-62.15	
Republic of Indonesia	2	619	1318	54.88	25.83-82.23	
New Zealand	1	1529	3616	42.28	40.68-43.9	
South Africa	1	30	63	47.62	35.34-60.04	
Libya	1	53	86	61.63	51.07-71.67	
Spain	2	665	1427	45.89	41.32-50.49	
Mexico	1	503	755	66.62	63.22-69.94	
Morocco	1	132	299	44.15	38.55-49.82	
Thailand	1	115	148	77.7	70.61-84.08	
Poland	1	116	241	48.13	41.84-54.46	
Belarus	1	25	119	21.01	14.12-28.83	
Belgium	1	58	79	73.42	63.07-82.64	
Greece	1	23	100	23	15.23-31.8	
Colombia	1	597	1739	34.33	32.12-36.58	
Lebanon	1	157	271	57.93	52-63.76	
**Continent**						**<0.01**
Asia	93	27195	67983	51.75	46.16-57.32	
Africa	15	3409	7410	47.87	36.46-59.4	

Oceania	9	3992	8031	58.63	49.7-67.29	
South America	11	7202	12284	60.49	51.49-69.15	
North America	13	5868	21014	36.25	28.28-44.62	
Europe	11	5402	11508	43.64	35.49-51.96	
Multi	1	1671	3006	55.59	53.81-57.36	
**Income level**						**0.03**
Lower-Middle-Income	61	14356	28188	54.29	47.26-61.23	
Low-Income	1	90	226	39.82	33.52-46.3	
Upper-Middle-Income	41	14604	42853	49.04	41.83-56.26	
High-Income	49	22060	53890	47.15	41.47-52.86	
**Development status**						0.08
Developing	100	27174	67598	52.76	47.58-57.91	
Developed	51	22265	54553	45.91	40.42-51.45	
**Publication date**						**0.02**
Before 2011	54	21254	54269	44.85	39.18-50.59	
After 2011	99	33485	76967	53.66	48.64-58.65	
**Study size**						**<0.01**
<1000	119	21542	40474	53.92	49.55-58.27	
>1000	34	33197	90762	38.99	32.14-46.07	
**Study quality**						0.30
<8	24	9600	25450	46.77	39.62-53.98	
≥8	129	45139	105786	51.23	46.84-55.62	
**Diagnosis criteria**						**<0.01**
MRS	64	26973	47330	57.13	52.24-61.96	
The Greene Climacteric Scale	11	4985	9473	52.05	42.75-61.28	
Face-to-face interview	18	7119	30863	31.72	22.59-41.62	
MENQOL	22	4950	8439	63.93	55.11-72.31	
Others	23	6305	16115	42.7	31.82-53.94	
SAS	2	291	1460	49.08	0-100	
KMI	4	1394	5772	26.98	11.17-46.56	
GAD	3	1582	9055	26.97	13.37-43.23	
CS-10	2	918	2190	52.89	18.44-85.83	
BDI	4	222	539	40.73	18.12-65.61	

**Risk factors**	**Studies**	**Event**	**Total**	**Prevalence (%)**	**95% CI (%)**	**P value**
**Menopausal stage**						0.22
Premenopause	38	4112	13768	46.57	38.83-54.38	
Perimenopause	41	3918	10346	53.41	45.39-61.34	

Postmenopause	56	11046	25802	55.7	48.74-62.55	
**Age**						0.53
<50	6	562	2102	44.67	22.17-68.37	
≥50	8	1217	3175	55.11	33.9-75.4	
**BMI**						**0.02**
Underweight	1	118	681	17.33	14.57-20.27	
Normal weight	1	531	3865	13.74	12.67-14.84	
Overweight	3	408	2700	21.81	7.88-40.15	
Obesity	4	376	1264	44.56	13.67-78.01	
**Urban or rural**						0.75
Rural	11	4360	9751	58.38	39.7-75.9	
Urban	6	1492	6079	51.53	16.49-85.73	
**Education level**						0.59
<12 years	4	1423	7954	47.79	10.19-86.96	
>12 years	3	138	878	31.59	3.1-71.35	

Supplementary Table 8. Subgroup analysis and risk factors for **Irritability**

**Subgroup**	**Studies**	**Event**	**Total**	**Prevalence (%)**	**95% CI (%)**	**P value**
**Country**						**<0.01**
Nepal	7	1772	4386	45.14	26.18-64.86	
Nigeria	6	893	2487	40.66	21.39-61.55	
Iran	5	2235	3153	71.53	48.04-90.09	
Ethiopia	1	102	226	45.13	38.68-51.67	
Turkey	7	1757	2796	71.66	59.38-82.51	
Saudi	6	1732	2271	76.22	55.11-92.16	
UK	3	989	2026	50.62	38.74-62.45	
France	2	411	900	44.18	34.55-54.03	
Germany	3	1590	2789	49.38	33.81-65.02	
Belgium	1	309	594	52.02	48-56.03	
Netherlands	2	471	901	50.08	36.35-63.8	
Switzerland	2	360	901	35.59	14.45-60.2	
Spain	5	5652	13600	41.5	35.31-47.83	
India	17	1882	4295	46.71	34.3-59.34	
Australia	5	2041	3720	60.94	46.64-74.35	
Japan	5	2655	5666	47.31	27.73-67.33	
Oman	1	186	472	39.41	35.04-43.86	
Macau, China	1	345	442	78.05	74.07-81.8	
Ecuador	4	617	1293	48.73	27.72-69.97	
Peru	1	589	771	76.39	73.33-79.33	
Malaysia	5	522	1014	58.01	44.53-70.92	
China	11	6273	16804	53.63	43.64-63.47	

Sri Lanka	2	335	1033	35.94	13.85-61.76	
Brazil	2	1262	1626	76.38	71.12-81.28	
Pakistan	4	2581	3549	54.11	25.08-81.72	
Greece	1	482	1025	47.02	43.97-50.09	
the Philippines	1	161	195	82.56	76.9-87.59	
Republic of Indonesia	3	295	622	40	12.25-71.79	
Korea	2	1603	2578	62.24	60.35-64.11	
Taiwan	4	12202	21424	46.13	35.59-56.85	
Thailand	2	199	300	68.27	24.19-98.23	
Vietnam	1	98	100	98	94.07-99.97	
Italy	1	99	301	32.89	27.69-38.31	
Poland	1	134	349	38.4	33.35-43.56	
Iraq	1	206	342	60.23	54.99-65.37	
USA	4	692	1482	55.83	38.64-72.34	
Egypt	5	2071	3704	58.59	40.05-75.95	
Bangladesh	4	692	1369	53.29	35.77-70.4	
Cambodia	1	152	177	85.88	80.32-90.65	
Sweden	1	64	108	59.26	49.82-68.37	
New Zealand	1	1060	3616	29.31	27.84-30.81	
South Africa	1	40	63	63.49	51.16-75.01	
Morocco	1	126	299	42.14	36.59-47.79	
Singapore	1	195	656	29.73	26.29-33.28	
Hong Kong	1	107	150	71.33	63.81-78.31	
Portugue	1	368	728	50.55	46.92-54.18	
Mexico	1	2465	4548	54.2	52.75-55.65	
Jordan	1	87	143	60.84	52.69-68.7	
Lebanon	1	187	271	69	63.36-74.38	
**Continent**						**0.01**
Asia	94	38459	74208	56.72	51.77-61.61	
Africa	14	3232	6779	49.1	37.54-60.71	
Europe	23	10929	24222	45.47	41.43-49.54	
Oceania	6	3101	7336	55.64	39.82-70.89	
South America	7	2468	3690	60.92	44.72-75.98	
North America	5	3157	6030	55.44	42.18-68.31	
**Income level**						**<0.01**
Lower-Middle-Income	58	13480	25640	52.24	45.32-59.12	
Low-Income	1	102	226	45.13	38.68-51.67	
Upper-Middle-Income	37	14362	30142	61.3	55.55-66.9	
High-Income	53	33402	66257	51.98	46.94-57	

**Development status**						0.55
Developing	95	39624	76418	55.13	50.3-59.92	
Developed	54	21722	45847	52.99	47.97-57.99	
**Publication date**						**0.01**
Before 2011	72	18892	46682	49.72	44.99-54.44	
After 2011	77	42454	75583	58.66	53.54-63.69	
**Study size**						0.14
<1000	122	22333	41339	55.58	51.57-59.55	
>1000	27	39013	80926	49.01	41.42-56.63	
**Study quality**						0.32
<8	21	7870	16587	50.02	40.83-59.20	
≥8	128	53476	105678	55.08	51.21-58.93	
**Diagnosis criteria**						**<0.01**
MRS	62	22422	37548	60.59	54.98-66.06	
Face-to-face interview	33	8136	22164	45.57	38.94-52.28	
Others	33	18299	34611	55.06	46.8-63.19	
The Greene Climacteric Scale	12	4631	10479	48.48	41.52-55.48	
SMI	2	636	2338	26.38	7.6-51.38	
MENQOL	2	241	426	57.8	47.49-67.77	
KMI	5	6981	14699	54.61	38.76-70	

**Risk factors**	**Studies**	**Event**	**Total**	**Prevalence (%)**	**95% CI (%)**	**P value**
**Menopausal stage**						0.10
Premenopause	34	6347	15718	45.2	36.83-53.7	
Perimenopause	39	8530	17061	57.3	50.21-64.24	
Postmenopause	63	20095	41526	53.74	47.4-60.03	
**Age**						0.69
<50	5	201	535	39.62	31.42-48.12	
≥50	11	1855	5084	44.28	24.43-65.13	
**Urban or rural**						0.15
Rural	10	4883	9626	43.02	27.9-58.83	
Urban	4	4180	8192	73.63	34.66-98.25	

Supplementary Table 9. Subgroup analysis and risk factors for **Mood Swings**

**Subgroup**	**Studies**	**Event**	**Total**	**Prevalence (%)**	**95% CI (%)**	**P value**
**Country**						**<0.01**
China	11	18671	46285	47.04	37.96-56.23	
UK	4	2495	5302	48.4	39.08-57.76	
France	2	411	900	42.47	24.72-61.29	
Germany	2	448	896	46.55	26.66-67.03	

Belgium	2	392	673	60.8	51.24-69.96	
Netherlands	2	474	901	50.59	37.8-63.35	
Switzerland	2	444	901	45.97	26.95-65.61	
Spain	1	342	600	57	53.02-60.94	
India	6	1005	2160	48.62	23.67-73.94	
Malaysia	3	250	450	59.32	48.08-70.11	
the Philippines	1	138	195	70.77	64.17-76.96	
Republic of Indonesia	1	23	59	38.98	26.86-51.81	
Korea	1	61	97	62.89	53-72.26	
Pakistan	2	528	921	57.38	54.15-60.58	
Taiwan	2	166	381	43.55	38.58-48.59	
Thailand	2	163	300	54.46	31.72-76.26	
Vietnam	1	96	100	96	91.09-99.13	
Italy	2	432	1329	32.49	30-35.04	
Poland	2	393	590	70.02	40.6-92.46	
Iran	1	91	1397	6.51	5.28-7.87	
USA	3	2567	10192	32.61	18.69-48.3	
Iraq	1	207	500	41.4	37.11-45.75	
Multi	1	1619	3520	45.99	44.35-47.64	
Hong Kong	1	117	150	78	70.99-84.3	
Portugal	1	381	728	52.34	48.7-55.96	
Belarus	1	100	119	84.03	76.86-90.12	
Greece	1	60	100	60	50.19-69.42	
Nepal	2	339	2180	9.08	0.54-25.8	
**Continent**						0.09
Asia	35	21855	55175	48.66	40.35-57.01	
Europe	22	6372	13039	52.06	45.76-58.32	
North America	3	2567	10192	32.61	18.69-48.3	
Multi	1	1619	3520	45.99	44.35-47.64	
**Income level**						0.77
Upper-Middle-Income	18	19391	47654	51.63	43.96-59.26	
High-Income	29	10802	27260	49.27	43.91-54.63	
Lower-Middle-Income	14	2220	7012	44.84	26.8-63.61	
**Development status**						0.31
Developing	30	21427	54478	46.08	36.93-55.38	
Developed	31	10986	27448	51.77	46.12-57.4	
**Publication date**						0.5452
Before 2011	35	5926	12864	50.55	44.15-56.94	
After 2011	26	26487	69062	47.06	37.85-56.37	

**Study size**						**<0.01**
<1000	47	8071	16130	52.87	46.92-58.77	
>1000	17	24342	65796	36.76	27.26-46.8	
**Study quality**						0.09
<8	5	12459	37378	30.46	11.58-53.64	
≥8	56	19954	44548	50.76	45.43-56.08	
**Diagnosis criteria**						0.38
KMI	9	18341	45737	44.2	34.17-54.47	
Face-to-face interview	25	6822	19789	45.22	36.5-54.09	
Others	25	6509	14896	54.18	45.68-62.57	
MRS	2	741	1504	56.6	17.58-91.26	

Risk factors	Studies	Event	Total	Prevalence (%)	95% CI (%)	P value
**Menopausal stage**						0.18
Premenopause	6	5862	18272	34.61	23.52-46.61	
Perimenopause	8	3752	8660	42.21	26.61-58.64	
Postmenopause	17	8409	19316	49.55	39.06-60.07	

Supplementary Table 10. Subgroup analysis and risk factors for **Poor memory or forgetfulness**

Subgroup	Studies	Event	Total	Prevalence (%)	95% CI (%)	P value
**Country**						**<0.01**
India	14	1477	3118	43.82	30.91-57.16	
Iran	5	1656	4060	44.64	21.55-69.03	
China	4	926	1539	59.3	43.11-74.53	
Malaysia	2	216	300	72.34	67.02-77.38	
Korea	3	1853	2441	73.95	62.89-83.66	
Japan	2	2319	3030	77.57	70.61-83.83	
Singapore	1	223	495	45.05	40.69-49.45	
the Philippines	1	166	195	85.13	79.76-89.8	
Republic of Indonesia	1	43	59	72.88	60.74-83.56	
Pakistan	3	657	1229	55.65	22.6-86.13	
Taiwan	2	14021	20963	66.99	66.34-67.63	
Thailand	1	123	150	82	75.41-87.77	
Vietnam	1	99	100	99	95.76-100	
UK	3	1979	4631	39.04	31.08-47.29	
Ecuador	1	262	325	80.62	76.13-84.74	
Qatar	1	308	1158	26.6	24.09-29.18	
United Arab Emirates	1	98	390	25.13	20.94-29.56	
USA	8	3833	12978	46.07	35.23-57.11	
Australia	4	593	832	73.56	55.5-88.28	

Turkey	1	301	519	58	53.72-62.21	
Nigeria	2	340	620	56.43	33.62-77.89	
Iraq	1	293	500	58.6	54.25-62.89	
Saudi	1	29	90	32.22	22.92-42.28	
Morocco	1	102	299	34.11	28.84-39.59	
Portugal	1	355	728	48.76	45.14-52.4	
Poland	1	161	241	66.8	60.72-72.62	
Belarus	1	63	119	52.94	43.92-61.87	
Belgium	1	70	79	88.61	80.54-94.81	
Greece	1	43	100	43	33.42-52.85	
Lebanon	1	146	271	53.87	47.91-59.78	
Brazil	1	148	211	70.14	63.78-76.14	
Nepal	2	466	2180	15.34	4.63-30.68	
**Continent**						**<0.01**
Asia	48	25420	42787	53.87	46.38-61.27	
Europe	8	2671	5898	52.53	39.38-65.5	
South America	2	410	536	75.72	64.78-85.2	
North America	8	3833	12978	46.07	35.23-57.11	
Oceania	4	593	832	73.56	55.5-88.28	
Africa	3	442	919	48.92	29.62-68.37	
**Income level**						**0.02**
Lower-Middle-Income	31	5152	12131	48.5	38.54-58.52	
Upper-Middle-Income	12	2332	3663	65.66	58.1-72.85	
High-Income	30	25885	48156	55.98	48.4-63.43	
**Development status**						0.92
Developing	46	22035	37580	54.22	46.79-61.56	
Developed	27	11334	26370	54.77	46.47-62.95	
**Publication date**						0.05
Before 2011	38	8091	15964	59.63	52.09-66.94	
After 2011	35	25278	47986	48.79	40.96-56.64	
**Study size**						0.41
<1000	63	9308	16957	55.44	49.5-61.3	
>1000	10	24061	46993	48.31	32.73-64.06	
**Study quality**						0.44
<8	8	4720	12878	46.92	27.05-67.32	
≥8	65	28649	51072	55.37	49.64-61.02	
**Diagnosis criteria**						**<0.01**
Others	31	20504	35916	56.56	47.26-65.63	
Face-to-face interview	16	5052	15989	40.08	30.37-50.21	

MENQOL	18	4316	7079	57.96	48.08-67.52	
The Keio questionnaire	2	2319	3030	77.57	70.61-83.83	
the Midlife Women's Symptom Index	2	391	670	58.37	54.6-62.09	
WHAS	2	288	374	80	52.66-97.3	
KMI	2	499	892	49.35	31.94-66.85	

Risk factors	Studies	Event	Total	Prevalence (%)	95% CI (%)	P value
**Menopausal stage**						0.16
Premenopause	13	4959	8116	48.18	34.54-61.96	
Perimenopause	13	5068	7854	64.39	55.37-72.94	
Postmenopause	25	12375	20004	59.3	51.83-66.57	
**Urban or rural**						**<0.01**
Rural	8	644	1502	49.3	32.9-65.77	
Urban	1	245	299	81.94	77.36-86.11	

Supplementary Table 11. Subgroup analysis and risk factors for **Difficulty concentrating**

Subgroup	Studies	Event	Total	Prevalence (%)	95% CI (%)	P value
**Country**						**<0.01**
India	10	787	2896	22.68	11.36-36.49	
Australia	5	1583	2760	54.35	29.67-77.95	
Japan	2	1601	2258	69.25	54.28-82.39	
Pakistan	2	429	858	50.44	45.62-55.26	
China	5	2538	11156	33.35	23.59-43.89	
Malaysia	2	267	350	70.68	50.63-87.39	
Greece	2	430	1125	38.17	35.34-41.05	
the Philippines	1	133	195	68.21	61.48-74.57	
Republic of Indonesia	1	29	59	49.15	36.4-61.96	
Korea	1	72	97	74.23	65.01-82.49	
Taiwan	1	40	81	49.38	38.5-60.3	
Thailand	2	203	298	68.14	62.71-73.33	
Vietnam	1	97	100	97	92.53-99.62	
Poland	2	249	590	45.06	13.79-78.71	
USA	5	713	2050	44.21	26.65-62.55	
Egypt	1	371	450	82.44	78.78-85.83	
Nigeria	1	51	186	27.42	21.23-34.07	
Bangladesh	1	113	150	75.33	68.09-81.93	
Iraq	1	269	500	53.8	49.41-58.16	
South Africa	1	38	63	60.32	47.9-72.11	
Morocco	1	54	299	18.06	13.89-22.64	

Singapore	1	190	656	28.96	25.55-32.5	
Belarus	1	48	119	40.34	31.67-49.32	
Belgium	1	56	79	70.89	60.33-80.44	
Ecuador	1	335	385	87.01	83.46-90.2	
UK	1	71	254	27.95	22.59-33.65	
Lebanon	1	117	271	43.17	37.32-49.12	
Nepal	2	134	2180	3.98	0.62-9.88	
**Continent**						**<0.01**
Asia	34	7019	22105	42.09	32.43-52.07	
Oceania	5	1583	2760	54.35	29.67-77.95	
Europe	7	854	2167	43.8	31.69-56.3	
North America	5	713	2050	44.21	26.65-62.55	
Africa	4	514	998	46.84	17.92-76.97	
South America	1	335	385	87.01	83.46-90.2	
**Income level**						0.13
Lower-Middle-Income	22	2315	7644	35.31	22.63-49.14	
High-Income	21	5005	9950	50.1	40.92-59.29	
Upper-Middle-Income	13	3698	12871	52.77	40.92-64.47	
**Development status**						0.12
Developing	33	5738	20127	40.38	30.39-50.79	
Developed	23	5280	10338	51.29	42.42-60.13	
**Publication date**						0.04
Before 2011	34	8714	22590	50.76	41.89-59.6	
After 2011	22	2304	7875	35.86	25.25-47.22	
**Study size**						0.5
<1000	52	7402	16393	45.78	38.45-53.2	
>1000	4	3616	14072	33.22	7.65-65.98	
**Study quality**						0.85
<8	7	2065	3730	42.61	19.24-67.86	
≥8	49	8953	26735	45.16	37.72-52.71	
**Diagnosis criteria**						**<0.01**
Others	30	3281	9407	45.86	36.22-55.66	
The Greene Climacteric Scale	10	3997	6639	58.27	47.05-69.08	
Face-to-face interview	12	3174	13153	28.48	14.67-44.74	
WHAS	2	258	374	71.84	42.92-93.36	
KMI	2	308	892	36.25	28.97-43.86	
**Risk factors**	**Studies**	**Event**	**Total**	**Prevalence (%)**	**95% CI (%)**	**P value**

**Menopausal stage**						0.26
Premenopause	7	797	4242	26.14	8.34-49.34	
Perimenopause	7	998	3041	50.41	32.26-68.51	
Postmenopause	11	1945	5997	43.75	27.07-61.19	

Supplementary Table 12. Subgroup analysis and risk factors for **Formication**

Subgroup	Studies	Event	Total	Prevalence (%)	95% CI (%)	P value
**Country**						**<0.01**
China	14	7619	51606	17.13	12.61-22.19	
USA	1	30	139	21.58	15.11-28.84	
Egypt	1	340	450	75.56	71.47-79.42	
**Continent**						**<0.01**
Asia	14	7619	51606	17.13	12.61-22.19	
North America	1	30	139	21.58	15.11-28.84	
Africa	1	340	450	75.56	71.47-79.42	
**Income level**						**<0.01**
Upper-Middle-Income	14	7619	51606	17.13	12.61-22.19	
High-Income	1	30	139	21.58	15.11-28.84	
Lower-Middle-Income	1	340	450	75.56	71.47-79.42	
**Development status**						0.81
Developing	15	7959	52056	20.43	12.97-29.09	
Developed	1	30	139	21.58	15.11-28.84	
**Publication date**						**<0.01**
Before 2011	1	340	450	75.56	71.47-79.42	
After 2011	15	7649	51745	17.39	13.07-22.18	
**Study size**						0.19
<1000	5	629	2108	30.47	11.53-53.69	
>1000	11	7360	50087	16.58	11.23-22.73	
**Study quality**						0.07
<8	3	2549	28387	12.08	6.16-19.65	
≥8	13	5440	23808	22.71	14.22-32.52	
**Diagnosis criteria**						0.23
KMI	14	7619	51606	17.13	12.61-22.19	
Face-to-face interview	2	370	589	48.49	4.31-94.35	

Risk factors	Studies	Event	Total	Prevalence (%)	95% CI (%)	P value
**Menopausal stage**						0.23
Premenopause	4	1526	17197	10.58	5.23-17.51	
Perimenopause	5	1184	8141	16.15	10.19-23.18	
Postmenopause	5	1764	10820	22.8	9.49-39.75	

Supplementary Table 13. Subgroup analysis and risk factors for **Change in the appearance, texture, or tone of my skin**

Subgroup	Studies	Event	Total	Prevalence (%)	95% CI (%)	P value
**Country**						**<0.01**
Korea	1	1438	1774	81.06	79.2-82.85	
India	7	637	1492	49.86	36.15-63.57	
Turkey	1	287	874	32.84	29.76-35.99	
Iran	2	538	902	40.13	0-95.98	
Ecuador	1	232	325	71.38	66.34-76.18	
Qatar	1	286	1158	24.7	22.25-27.22	
United Arab Emirates	1	96	390	24.62	20.46-29.02	
Australia	3	307	672	45.73	29.17-62.79	
Saudi	1	31	90	34.44	24.93-44.61	
**Continent**						**<0.01**
Asia	14	3313	6680	44.8	32.57-57.35	
South America	1	232	325	71.38	66.34-76.18	
Oceania	3	307	672	45.73	29.17-62.79	
**Income level**						0.88
High-Income	7	2158	4084	43.22	27.34-59.84	
Lower-Middle-Income	9	1175	2394	47.76	32.31-63.43	
Upper-Middle-Income	2	519	1199	52.21	16.56-86.59	
**Development status**						0.67
Developing	14	2001	4265	47.96	36.86-59.15	
Developed	4	1851	3412	41.29	16.14-69.17	
**Publication date**						0.8
Before 2011	6	762	1739	44.75	30.27-59.68	
After 2011	12	3090	5938	47.35	33.49-61.41	
**Study size**						0.8
<1000	16	2128	4745	45.57	35.41-55.92	
>1000	2	1724	2932	53.5	5.27-97.42	
**Study quality**						**<0.01**
<8	2	1670	2099	76.63	66.52-85.41	
≥8	16	2182	5578	42.49	32.75-52.53	

Risk factors	Studies	Event	Total	Prevalence (%)	95% CI (%)	P value
**Menopausal stage**						0.98
Premenopause	5	853	1595	44.46	24.68-65.19	
Perimenopause	6	770	1548	43.8	24.4-64.18	
Postmenopause	8	959	2109	46.1	31.81-60.72	

Supplementary Table 14. Subgroup analysis and risk factors for **Increased facial hair**

Subgroup	Studies	Event	Total	Prevalence (%)	95% CI (%)	P value
**Country**						**<0.01**
India	8	421	1490	28.86	14.86-45.27	
Malaysia	1	65	276	23.55	18.72-28.75	
Korea	1	451	1774	25.42	23.42-27.48	
Turkey	2	314	1006	38.85	18.47-61.49	
Iran	3	697	2299	31.16	18.09-45.97	
Egypt	1	221	450	49.11	44.49-53.73	
Ecuador	1	63	325	19.38	15.26-23.87	
Nigeria	1	6	186	3.23	1.08-6.34	
Qatar	1	186	1158	16.06	14-18.24	
United Arab Emirates	1	62	390	15.9	12.43-19.7	
Australia	3	207	672	30.42	22.25-39.26	
Saudi	1	31	90	34.44	24.93-44.61	
Libya	1	13	86	15.12	8.23-23.55	
**Continent**						**0.05**
Asia	18	2227	8483	28.47	21.24-36.29	
Africa	3	240	722	19.32	1.09-51.18	
South America	1	63	325	19.38	15.26-23.87	
Oceania	3	207	672	30.42	22.25-39.26	
**Income level**						0.91
Lower-Middle-Income	13	1345	4425	28.2	17.62-40.14	
Upper-Middle-Income	5	455	1693	26.74	16.22-38.77	
High-Income	7	937	4084	25.4	19.21-32.12	
**Development status**						0.21
Developing	20	1942	6514	28.4	20.92-36.53	
Developed	5	795	3688	22.11	16.33-28.47	
**Publication date**						0.8
Before 2011	10	1015	3828	26.23	17.14-36.47	
After 2011	15	1722	6374	27.84	19.67-36.83	
**Study size**						0.15
<1000	22	1776	5873	28.05	21.13-35.53	
>1000	3	961	4329	21.45	16.1-27.33	
**Study quality**						0.28
<8	2	514	2099	22.7	17.14-28.79	
≥8	23	2223	8103	27.64	21-34.8	
**Diagnosis criteria**						0.72

MENQOL	20	2063	7937	25.67	19.96-31.81	
Others	3	129	418	32.45	3.34-72.95	
Face-to-face interview	2	545	1847	35.54	13.06-62.09	

**Risk factors**	**Studies**	**Event**	**Total**	**Prevalence (%)**	**95% CI (%)**	**P value**
**Menopausal stage**						0.10
Premenopause	6	322	1655	17.99	14.79-21.44	
Perimenopause	8	504	2318	20.31	15.7-25.32	
Postmenopause	11	835	2982	28.27	19.32-38.17	
**Urban or rural**						**<0.01**
Rural	4	92	536	16.35	1.94-40.18	
Urban	1	135	250	54	47.79-60.15	

Supplementary Table 15. Subgroup analysis and risk factors for **Drying skin**

**Subgroup**	**Studies**	**Event**	**Total**	**Prevalence (%)**	**95% CI (%)**	**P value**
**Country**						**<0.01**
India	10	802	2123	40.43	29.52-51.84	
Malaysia	1	159	276	57.61	51.72-63.39	
Korea	1	1487	1774	83.82	82.07-85.5	
Spain	1	573	1059	54.11	51.1-57.1	
Singapore	1	137	495	27.68	23.82-31.71	
Turkey	2	428	1006	42.03	37.7-46.42	
Iran	4	1064	2649	42.32	20.18-66.21	
UK	2	938	4527	22.55	17.39-28.16	
Egypt	1	408	450	90.67	87.79-93.19	
Ecuador	2	600	776	78.85	63.55-90.85	
Qatar	1	338	1158	29.19	26.6-31.84	
United Arab Emirates	1	113	390	28.97	24.57-33.58	
Australia	3	365	672	53.24	32.74-73.18	
Taiwan	1	11766	20882	56.35	55.67-57.02	
Saudi	1	31	90	34.44	24.93-44.61	
Japan	1	624	1069	58.37	55.4-61.31	
Pakistan	1	74	308	24.03	19.41-28.97	
USA	1	305	1143	26.68	24.16-29.29	
Thailand	1	120	148	81.08	74.34-87.02	
Colombia	1	1074	1739	61.76	59.46-64.03	
China	1	152	296	51.35	45.65-57.04	
Nepal	1	176	2000	8.8	7.6-10.08	
**Continent**						**<0.01**
Asia	28	17471	34664	42.56	34.87-50.43	
Europe	3	1511	5586	32.85	14.71-54.15	

Africa	1	408	450	90.67	87.79-93.19	
South America	3	1674	2515	73.39	58.54-85.94	
Oceania	3	365	672	53.24	32.74-73.18	
North America	1	305	1143	26.68	24.16-29.29	
**Income level**						**0.03**
Lower-Middle-Income	17	2524	7530	40.93	29.54-52.82	
Upper-Middle-Income	8	2533	4241	62.01	49.42-73.83	
High-Income	14	16677	33259	43.11	32.63-53.9	
**Development status**						0.32
Developing	28	17029	33049	48.25	39.41-57.15	
Developed	11	4705	11981	40.5	28.59-53	
**Publication date**						0.43
Before 2011	15	3061	6978	49.92	37.2-62.65	
After 2011	24	18673	38052	43.62	34.99-52.45	
**Study size**						0.51
<1000	29	4175	8536	47.61	39.47-55.82	
>1000	10	17559	36494	41.59	26.46-57.58	
**Study quality**						**0.01**
<8	6	2718	4191	68.91	49.89-85.11	
≥8	33	19016	40839	41.79	34.91-48.85	
**Diagnosis criteria**						**<0.01**
Others	11	14758	31437	38.98	25.06-53.87	
MENQOL	20	4399	8203	49.37	40.49-58.28	
Face-to-face interview	6	1181	3200	41.17	19.63-64.65	
CS-10	2	1396	2190	66.47	56.77-75.52	

**Risk factors**	**Studies**	**Event**	**Total**	**Prevalence (%)**	**95% CI (%)**	**P value**
**Menopausal stage**						0.56
Premenopause	8	3814	7418	41.65	24.68-59.7	
Perimenopause	10	3986	7591	53.48	36.69-69.88	
Postmenopause	16	8210	14516	52.21	43.04-61.31	
**Urban or rural**						0.45
Rural	4	212	376	60.85	38.12-81.4	
Urban	2	406	549	73.84	46.74-93.65	

Supplementary Table 16 GRADE approach for assessing the certainty of evidence in the current study.

Quality assessment							No of participants	Effect		Quality	Importance
No of studies	Design	Risk of bias[1]	Inconsistency[1]	Indirectness	Imprecision[2]	Other considerations[3]		Relative(95% CI)	Absolute		
**Prevalence of hot flashes in middle-aged women**											
265	observational studies	very serious	serious	no serious indirectness	serious	reporting bias strong association	349,608	-	-	VERY LOW	CRITICAL
**Prevalence of sleep problems in middle-aged women**											
219	observational studies	very serious	serious	no serious indirectness	serious	reporting bias strong association	318,656	-	-	VERY LOW	CRITICAL
**Prevalence of heart discomfort in middle-aged women**											
135	observational studies	very serious	serious	no serious indirectness	serious	reporting bias strong association	170,526	-	-	VERY LOW	CRITICAL
**Prevalence of headache in middle-aged women**											
108	observational studies	very serious	serious	no serious indirectness	serious	reporting bias strong association	162,907	-	-	VERY LOW	CRITICAL
**Prevalence of joint and muscular discomfort in middle-aged women**											
195	observational studies	very serious	serious	no serious indirectness	serious	reporting bias strong association	236,615	-	-	VERY LOW	CRITICAL
**Prevalence of physical and mental exhaustion in middle-aged women**											
177	observational studies	very serious	serious	no serious indirectness	serious	reporting bias strong association	198,507	-	-	VERY LOW	CRITICAL
**Prevalence of depression in middle-aged women**											
204	observational studies	very serious	serious	no serious indirectness	serious	reporting bias strong association	300,449	-	-	VERY LOW	CRITICAL
**Prevalence of anxiety in middle-aged women**											
145	observational studies	very serious	serious	no serious indirectness	serious	reporting bias strong association	131,236	-	-	VERY LOW	CRITICAL
**Prevalence of irritability in middle-aged women**											
125	observational studies	very serious	serious	no serious indirectness	serious	reporting bias strong association	122,265	-	-	VERY LOW	CRITICAL
**Prevalence of mood swings in middle-aged women**											
35	observational studies	very serious	serious	no serious indirectness	serious	reporting bias strong association	81,926	-	-	VERY LOW	CRITICAL
**Prevalence of sexual problems in middle-aged women**											
156	observational studies	very serious	serious	no serious indirectness	serious	reporting bias strong association	180,618	-	-	VERY LOW	CRITICAL
**Prevalence of vaginal dryness in middle-aged women**											
155	observational studies	very serious	serious	no serious indirectness	serious	reporting bias strong association	167,055	-	-	VERY LOW	CRITICAL
**Prevalence of urinary problems in middle-aged women**											
171	observational studies	very serious	serious	no serious indirectness	serious	reporting bias strong association	200,250	-	-	VERY LOW	CRITICAL
**Prevalence of forgetfulness in middle-aged women**											
62	observational studies	very serious	serious	no serious indirectness	serious	reporting bias strong association	30,465	-	-	VERY LOW	CRITICAL

**Prevalence of difficult concentration in middle-aged women**											
44	observational studies	very serious	serious	no serious indirectness	serious	reporting bias strong association	63,950	-	-	VERY LOW	CRITICAL
**Prevalence of formication in middle-aged women**											
16	observational studies	very serious	serious	no serious indirectness	serious	reporting bias strong association	52,195	-	-	VERY LOW	CRITICAL
**Prevalence of change in the appearance, texture, or tone of skin in middle-aged women**											
18	observational studies	very serious	serious	no serious indirectness	serious	reporting bias strong association	7,677	-	-	VERY LOW	CRITICAL
**Prevalence of increased facial hair in middle-aged women**											
25	observational studies	very serious	serious	no serious indirectness	serious	reporting bias strong association	10,202	-	-	VERY LOW	CRITICAL
**Prevalence of drying skin in middle-aged women**											
39	observational studies	very serious	serious	no serious indirectness	serious	reporting bias strong association	45,030	-	-	VERY LOW	CRITICAL

[1] menopausal symptoms were diagnosed by a variety of diagnostic tools. More importantly, most studies were cross-sectional studies which may further increase the bias.

[2] The effect is large, and consideration of CIs alone suggests a robust effect, but the total sample size is not large enough and the number of events is small.

[3] The prevalence varied among studies from different countries and regions. Even studies from the same country and region varied a lot.

Reference

1. Lan Y, Huang Y, Song Y, et al. Prevalence, severity, and associated factors of menopausal symptoms in middle-aged Chinese women: a community-based cross-sectional study in southeast China. Menopause. 2017;24(10):1200-1207. doi:10.1097/GME.0000000000000906
2. Adhikari D, Bhurtyal A. Menopausal symptoms among middle-aged women and care providers' readiness to deliver menopausal services: an observational study in Kavrepalanchok, Nepal. Sex Reprod Health Matters. 2021;29(2):2141255. doi:10.1080/26410397.2022.2141255
3. Wang X, Wang L, Di J, Zhang X, Zhao G. Prevalence and risk factors for menopausal symptoms in middle-aged Chinese women: a community-based cross-sectional study. Menopause. 2021;28(11):1271-1278. Published 2021 Aug 30. doi:10.1097/GME.0000000000001850
4. Yang R, Zhou Y, Li C, Tao M. Association between pulse wave velocity and hot flashes/sweats in middle-aged women. Sci Rep. 2017;7(1):13854. Published 2017 Oct 23. doi:10.1038/s41598-017-13395-z
5. Olaolorun FM, Lawoyin TO. Experience of menopausal symptoms by women in an urban community in Ibadan, Nigeria. Menopause. 2009;16(4):822-830. doi:10.1097/gme.0b013e318198d6e7
6. Chedraui P, Pérez-López FR, Aguirre W, et al. Beliefs regarding menopausal hot flushes among climacteric women as assessed with the Hot Flush Beliefs Scale. Maturitas. 2010;66(3):298-304. doi:10.1016/j.maturitas.2010.03.019
7. Li L, Wu J, Pu D, et al. Factors associated with the age of natural menopause and menopausal symptoms in Chinese women. Maturitas. 2012;73(4):354-360. doi:10.1016/j.maturitas.2012.09.008
8. Zagalaz-Anula N, Hita-Contreras F, Martínez-Amat A, et al. The associations between menopausal symptoms and sleep quality in Spanish postmenopausal women. Climacteric. 2019;22(5):511-517. doi:10.1080/13697137.2019.1609439
9. Javadivala Z, Allahverdipour H, Asghari Jafarabadi M, Emami A. An Interventional strategy of physical activity promotion for reduction of menopause symptoms. Health Promot Perspect. 2020;10(4):383-392. Published 2020 Nov 7. doi:10.34172/hpp.2020.57
10. Nagaraj D, Ramesh N, Devraj D, Umman M, John AK, Johnson AR. Experience and Perceptions Regarding Menopause among Rural Women: A Cross-Sectional Hospital-Based Study in South Karnataka. J Midlife Health. 2021;12(3):199-205. doi:10.4103/jmh.JMH_196_20
11. Yisma E, Eshetu N, Ly S, Dessalegn B. Prevalence and severity of menopause symptoms among perimenopausal and postmenopausal women aged 30-49 years in Gulele sub-city of Addis Ababa, Ethiopia. BMC Womens Health. 2017;17(1):124. Published 2017 Dec 8. doi:10.1186/s12905-017-0484-x
12. Korkmaz H, Arslanca T, Tekin E, Kayabaşı B, Akbulut KG. Evaluation of the relationship between plasma viscosity and selected menopausal symptoms using the Menopause Rating Scale. Menopause. 2022;29(9):1071-1076. doi:10.1097/GME.0000000000002017
13. AlQuaiz AM, Tayel SA, Habiba FA. Assessment of symptoms of menopause and their severity among Saudi women in Riyadh. Ann Saudi Med. 2013;33(1):63-67. doi:10.5144/0256-4947.2013.63
14. Yu Q, Chae HD, Hsiao SM, et al. Prevalence, severity, and associated factors in women in East Asia with moderate-to-severe vasomotor symptoms associated with menopause. Menopause. 2022;29(5):553-563. Published 2022 May 1. doi:10.1097/GME.0000000000001949

15. Genazzani AR, Schneider HP, Panay N, Nijland EA. The European Menopause Survey 2005: women's perceptions on the menopause and postmenopausal hormone therapy. Gynecol Endocrinol. 2006;22(7):369-375. doi:10.1080/09513590600842463
16. Aaron R, Muliyil J, Abraham S. Medico-social dimensions of menopause: a cross-sectional study from rural south India. Natl Med J India. 2002;15(1):14-17.
17. Abdel-Salam DM, Mohamed RA, Alruwaili RR, Alhablani FS, Aldaghmi RM, ALghassab RE. Postmenopausal Symptoms and Their Correlates among Saudi Women Attending Different Primary Health Centers. Int J Environ Res Public Health. 2021;18(13):6831. Published 2021 Jun 25. doi:10.3390/ijerph18136831
18. Anderson D, Yoshizawa T, Gollschewski S, Atogami F, Courtney M. Relationship between menopausal symptoms and menopausal status in Australian and Japanese women: preliminary analysis. Nurs Health Sci. 2004;6(3):173-180. doi:10.1111/j.1442-2018.2004.00190.x
19. El Shafie K, Al Farsi Y, Al Zadjali N, Al Adawi S, Al Busaidi Z, Al Shafaee M. Menopausal symptoms among healthy, middle-aged Omani women as assessed with the Menopause Rating Scale. Menopause. 2011;18(10):1113-1119. doi:10.1097/gme.0b013e31821b82ee
20. Aloufi B, Hassanien NS. The Association of Menopausal Symptoms and Social Support Among Saudi Women at Primary Health Care Centers in Taif, Saudi Arabia. Cureus. 2022;14(6):e26122. Published 2022 Jun 20. doi:10.7759/cureus.26122
21. Blümel JE, Chedraui P, Baron G, et al. Menopausal symptoms appear before the menopause and persist 5 years beyond: a detailed analysis of a multinational study. Climacteric. 2012;15(6):542-551. doi:10.3109/13697137.2012.658462
22. Chou MF, Wun YT, Pang SM. Menopausal symptoms and the menopausal rating scale among midlife chinese women in Macau, China. Women Health. 2014;54(2):115-126. doi:10.1080/03630242.2013.871767
23. Fallahzadeh H. Age at natural menopause in Yazd, Islamic Republic of Iran. Menopause. 2007;14(5):900-904. doi:10.1097/gme.0b013e318032b2e6
24. Fuh JL, Wang SJ, Lee SJ, Lu SR, Juang KD. Quality of life and menopausal transition for middle-aged women on Kinmen island. Qual Life Res. 2003;12(1):53-61. doi:10.1023/a:1022074602928
25. Chedraui P, Pérez-López FR, Hidalgo L, et al. Evaluation of the presence and severity of menopausal symptoms among postmenopausal women screened for the metabolic syndrome. Gynecol Endocrinol. 2014;30(12):918-924. doi:10.3109/09513590.2014.971236
26. Chedraui P, Pérez-López FR, Mendoza M, et al. Severe menopausal symptoms in middle-aged women are associated to female and male factors. Arch Gynecol Obstet. 2010;281(5):879-885. doi:10.1007/s00404-009-1204-z
27. Ojeda E, Blümel JE, Vallejo MS, Lavín P. Climacteric symptoms in Quechua and Mestizo women from the Andean region of Cusco, Peru: effects of altitude and ethnicity. Maturitas. 2014;77(4):356-360. doi:10.1016/j.maturitas.2014.01.011
28. Ikeda T, Makita K, Ishitani K, Takamatsu K, Horiguchi F, Nozawa S. Status of climacteric symptoms among middle-aged to elderly Japanese women: comparison of general healthy women with women presenting at a menopausal clinic. J Obstet Gynaecol Res. 2005;31(2):164-171. doi:10.1111/j.1341-8076.2005.00268.x
29. Ishizuka B, Kudo Y, Tango T. Cross-sectional community survey of menopause symptoms among

Japanese women. Maturitas. 2008;61(3):260-267. doi:10.1016/j.maturitas.2008.07.006
30. Kapur P, Sinha B, Pereira BM. Measuring climacteric symptoms and age at natural menopause in an Indian population using the Greene Climacteric Scale. Menopause. 2009;16(2):378-384. doi:10.1097/gme.0b013e31818a2be9
31. Sharma S, Adhikari L, Karmacharya I, Kaphle M. Menopausal Symptoms among Postmenopausal Women of a Selected Municipality: A Cross-sectional Survey. JNMA J Nepal Med Assoc. 2021;59(243):1155-1160. Published 2021 Nov 15. doi:10.31729/jnma.7052
32. Qazi RA. Age, pattern of menopause, climacteric symptoms and associated problems among urban population of Hyderabad, Pakistan. J Coll Physicians Surg Pak. 2006;16(11):700-703.
33. Rahman SA, Zainudin SR, Mun VL. Assessment of menopausal symptoms using modified Menopause Rating Scale (MRS) among middle age women in Kuching, Sarawak, Malaysia. Asia Pac Fam Med. 2010;9(1):5. Published 2010 Feb 22. doi:10.1186/1447-056X-9-5
34. Shea JL. Chinese women's symptoms: relation to menopause, age and related attitudes. Climacteric. 2006;9(1):30-39. doi:10.1080/13697130500499914
35. Som N, Ray S. Menopause-specific quality of life of urban women in West Bengal, India. Menopause Int. 2012;18(3):99-105. doi:10.1258/mi.2012.011107
36. Syed Alwi SA, Lee PY, Awi I, Mallik PS, Md Haizal MN. The menopausal experience among indigenous women of Sarawak, Malaysia. Climacteric. 2009;12(6):548-556. doi:10.3109/13697130902919519
37. Waidyasekera H, Wijewardena K, Lindmark G, Naessen T. Menopausal symptoms and quality of life during the menopausal transition in Sri Lankan women. Menopause. 2009;16(1):164-170. doi:10.1097/gme.0b013e31817a8abd
38. Tomida M, Otsuka R, Tange C, et al. Vasomotor symptoms, sleep problems, and depressive symptoms in community-dwelling Japanese women. J Obstet Gynaecol Res. 2021;47(10):3677-3690. doi:10.1111/jog.14937
39. He Y, Tian J, Oddy WH, et al. The associations of childhood adiposity with menopausal symptoms in women aged 45-49 years: An Australian Cohort Study. Maturitas. 2021;143:81-88. doi:10.1016/j.maturitas.2020.09.008
40. Kumari A, Panigrahi A, Roy A, Panda J. Impaired Quality of Life and Its Determinants among Postmenopausal Women of Slum Communities in Bhubaneswar, India. J Midlife Health. 2020;11(3):149-155. doi:10.4103/jmh.JMH_111_19
41. Malacara JM, Canto de Cetina T, Bassol S, et al. Symptoms at pre- and postmenopause in rural and urban women from three States of Mexico. Maturitas. 2002;43(1):11-19. doi:10.1016/s0378-5122(02)00077-4
42. da Silva AR, d'Andretta Tanaka AC. Factors associated with menopausal symptom severity in middle-aged Brazilian women from the Brazilian Western Amazon. Maturitas. 2013;76(1):64-69. doi:10.1016/j.maturitas.2013.05.015
43. Shringarpure KS, Kharawala A, Panchal PP, Brahme KM, Baxi SR, Baxi RK. Prevalence of menopausal symptoms among women in Vadodara, Central Gujarat: The urban-rural divide. J Family Med Prim Care. 2022;11(10):6049-6055. doi:10.4103/jfmpc.jfmpc_115_22
44. Martínez JA, Palacios S, Chavida F, Pérez M. Urban-rural differences in Spanish menopausal women. Rural Remote Health. 2013;13(2):1865.
45. Andac T, Aslan E. Sexual life of women in the climacterium: A community-based study. Health Care Women Int. 2017;38(12):1344-1355. doi:10.1080/07399332.2017.1352588

46. Yang D, Haines CJ, Pan P, et al. Menopausal symptoms in mid-life women in southern China. Climacteric. 2008;11(4):329-336. doi:10.1080/13697130802239075
47. Yim G, Ahn Y, Chang Y, et al. Prevalence and severity of menopause symptoms and associated factors across menopause status in Korean women. Menopause. 2015;22(10):1108-1116. doi:10.1097/GME.0000000000000438
48. Yokota M, Makita K, Hirasawa A, Iwata T, Aoki D. Symptoms and effects of physical factors in Japanese middle-aged women. Menopause. 2016;23(9):974-983. doi:10.1097/GME.0000000000000660
49. Gast GC, Grobbee DE, Pop VJ, et al. Menopausal complaints are associated with cardiovascular risk factors. Hypertension. 2008;51(6):1492-1498. doi:10.1161/HYPERTENSIONAHA.107.106526
50. Du L, Xu B, Huang C, Zhu L, He N. Menopausal Symptoms and Perimenopausal Healthcare-Seeking Behavior in Women Aged 40-60 Years: A Community-Based Cross-Sectional Survey in Shanghai, China. Int J Environ Res Public Health. 2020;17(8):2640. Published 2020 Apr 12. doi:10.3390/ijerph17082640
51. Buhling KJ, Daniels BV, Studnitz FS, Eulenburg C, Mueck AO. The use of complementary and alternative medicine by women transitioning through menopause in Germany: results of a survey of women aged 45-60 years. Complement Ther Med. 2014;22(1):94-98. doi:10.1016/j.ctim.2013.12.004
52. Singh A, Pradhan SK. Menopausal symptoms of postmenopausal women in a rural community of Delhi, India: A cross-sectional study. J Midlife Health. 2014;5(2):62-67. doi:10.4103/0976-7800.133989
53. Kulkarni P, Savitha Rani BB, Kumar DS, Manjunath R. Burgeoning menopausal symptoms: An urgent public health concern. J Midlife Health. 2016;7(2):83-87. doi:10.4103/0976-7800.185329
54. Borker SA, Venugopalan PP, Bhat SN. Study of menopausal symptoms, and perceptions about menopause among women at a rural community in Kerala. J Midlife Health. 2013;4(3):182-187. doi:10.4103/0976-7800.118997
55. Rathnayake N, Lenora J, Alwis G, Lekamwasam S. Prevalence and Severity of Menopausal Symptoms and the Quality of Life in Middle-aged Women: A Study from Sri Lanka. Nurs Res Pract. 2019;2019:2081507. Published 2019 Jul 1. doi:10.1155/2019/2081507
56. Kwon DH, Lee JH, Ryu KJ, Park HT, Kim T. Vasomotor symptoms and the homeostatic model assessment of insulin-resistance in Korean postmenopausal women. Obstet Gynecol Sci. 2016;59(1):45-49. doi:10.5468/ogs.2016.59.1.45
57. Baquedano L, Coronado P, De la Viuda E, et al. Population-based survey on menopausal symptoms and treatment use. Climacteric. 2023;26(1):47-54. doi:10.1080/13697137.2022.2139598
58. Brzyski RG, Medrano MA, Hyatt-Santos JM, Ross JS. Quality of life in low-income menopausal women attending primary care clinics. Fertil Steril. 2001;76(1):44-50. doi:10.1016/s0015-0282(01)01852-0
59. Hwang JH, Lee K, Choi E, et al. Sleep Quality and Associated Factors in Premenopausal, Perimenopausal, and Postmenopausal Women in Korea: Findings from the K-Stori 2016. Nat Sci Sleep. 2021;13:1137-1145. Published 2021 Jul 14. doi:10.2147/NSS.S298397
60. Ahmadieh H, Jradi N. Prevalence of menopausal hot flashes in Lebanon: A cross-sectional study.

Int J Reprod Biomed. 2021;19(9):789-800. Published 2021 Oct 10. doi:10.18502/ijrm.v19i9.9711
61. Namgoung S, Chang Y, Woo CY, et al. Metabolically healthy and unhealthy obesity and risk of vasomotor symptoms in premenopausal women: cross-sectional and cohort studies. BJOG. 2022;129(11):1926-1934. doi:10.1111/1471-0528.17224
62. Pateliya K, Patel J, Parmar P, et al. A Cross Sectional Study To Assess Menopausal Symptoms Among Post-Menopausal Women At Kheda District. Journal of Pharmaceutical Negative Results. 2022;13:5173-5183. doi: 10.47750/pnr.2022.13.S09.638
63. Aariya S, Archana K, et al. Assessment of prevalence of vasomotor menopausal symptoms using a modified menopause rating scale(MRS) in Peri and postmenopausal women. International Journal of Research in Pharmaceutical Sciences. 2021; 12(1):136-140. doi: 10.26452/ijrps.v12i1.3968
64. Chim H, Tan BH, Ang CC, Chew EM, Chong YS, Saw SM. The prevalence of menopausal symptoms in a community in Singapore. Maturitas. 2002;41(4):275-282. doi:10.1016/s0378-5122(01)00299-7
65. Dhillon HK, Singh HJ, Shuib R, Hamid AM, Mohd Zaki Nik Mahmood N. Prevalence of menopausal symptoms in women in Kelantan, Malaysia. Maturitas. 2006;54(3):213-221. doi:10.1016/j.maturitas.2005.11.001
66. Li Y, Yu Q, Ma L, Sun Z, Yang X. Prevalence of depression and anxiety symptoms and their influence factors during menopausal transition and postmenopause in Beijing city. Maturitas. 2008;61(3):238-242. doi:10.1016/j.maturitas.2008.09.002
67. Santoro N, Komi J. Prevalence and impact of vaginal symptoms among postmenopausal women. J Sex Med. 2009;6(8):2133-2142. doi:10.1111/j.1743-6109.2009.01335.x
68. Chuni N, Sreeramareddy CT. Frequency of symptoms, determinants of severe symptoms, validity of and cut-off score for Menopause Rating Scale (MRS) as a screening tool: a cross-sectional survey among midlife Nepalese women. BMC Womens Health. 2011;11:30. Published 2011 Jun 14. doi:10.1186/1472-6874-11-30
69. Valadares AL, Pinto-Neto AM, de Souza MH, Osis MJ, da Costa Paiva LH. The prevalence of the components of low sexual function and associated factors in middle-aged women. J Sex Med. 2011;8(10):2851-2858. doi:10.1111/j.1743-6109.2011.02405.x
70. Nisar N, Sikandar R, Sohoo NA. Menopausal symptoms: prevalence, severity and correlation with sociodemographic and reproductive characteristics. A cross sectional community based survey from rural Sindh Pakistan. J Pak Med Assoc. 2015;65(4):409-413.
71. Zhang Y, Zhao X, Leonhart R, et al. A cross-cultural comparison of climacteric symptoms, self-esteem, and perceived social support between Mosuo women and Han Chinese women. Menopause. 2016;23(7):784-791. doi:10.1097/GME.0000000000000621
72. Agaba PA, Meloni ST, Sule HM, et al. Prevalence and predictors of severe menopause symptoms among HIV-positive and -negative Nigerian women. Int J STD AIDS. 2017;28(13):1325-1334. doi:10.1177/0956462417704778
73. AlQuaiz AM, Kazi A, Habib F, AlBugami M, AlDughaither A. Factors associated with different symptom domains among postmenopausal Saudi women in Riyadh, Saudi Arabia. Menopause. 2017;24(12):1392-1401. doi:10.1097/GME.0000000000000931
74. Grigoriou V, Augoulea A, Armeni E, et al. Prevalence of vasomotor, psychological, psychosomatic and sexual symptoms in perimenopausal and recently postmenopausal Greek

women: association with demographic, life-style and hormonal factors. Gynecol Endocrinol. 2013;29(2):125-128. doi:10.3109/09513590.2012.708801

75. Hunter MS, Gentry-Maharaj A, Ryan A, et al. Prevalence, frequency and problem rating of hot flushes persist in older postmenopausal women: impact of age, body mass index, hysterectomy, hormone therapy use, lifestyle and mood in a cross-sectional cohort study of 10,418 British women aged 54-65. BJOG. 2012;119(1):40-50. doi:10.1111/j.1471-0528.2011.03166.x
76. Haines CJ, Xing SM, Park KH, Holinka CF, Ausmanas MK. Prevalence of menopausal symptoms in different ethnic groups of Asian women and responsiveness to therapy with three doses of conjugated estrogens/medroxyprogesterone acetate: the Pan-Asia Menopause (PAM) study. Maturitas. 2005;52(3-4):264-276. doi:10.1016/j.maturitas.2005.03.012
77. Melby MK. Vasomotor symptom prevalence and language of menopause in Japan. Menopause. 2005;12(3):250-257. doi:10.1097/01.gme.0000146108.27840.d9?
78. Dennerstein L, Dudley EC, Hopper JL, Guthrie JR, Burger HG. A prospective population-based study of menopausal symptoms. Obstet Gynecol. 2000;96(3):351-358. doi:10.1016/s0029-7844(00)00930-3
79. Nappi RE, Nijland EA. Women's perception of sexuality around the menopause: outcomes of a European telephone survey. Eur J Obstet Gynecol Reprod Biol. 2008;137(1):10-16. doi:10.1016/j.ejogrb.2006.10.036
80. Gotmar A, Hammar M, Fredrikson M, et al. Symptoms in peri- and postmenopausal women in relation to testosterone concentrations: data from The Women's Health in the Lund Area (WHILA) study. Climacteric. 2008;11(4):304-314. doi:10.1080/13697130802249769
81. Górecka K, Krzyżanowska M. Prevalence of menopausal hormone therapy and alternative methods, health benefits experienced by peri- and postmenopausal Polish women. Prz Menopauzalny. 2022;21(1):27-36. doi:10.5114/pm.2022.113731
82. Nadia HS, Muna MA, Nahla KA, et al. Prevalence of menopausal symptoms and its relationship with socio-demographic factors among women above 45 years in Mosul, Iraq. Sociedad Latinoamericana de Hipertensión. 2021;16:1-7. doi: 10.5281/zenodo.5095349
83. Sheereen F, Kadarkar KS. Community-based appraisal of menopause-specific health problems and quality of life among women of rural Western Maharashtra. J Family Med Prim Care. 2022;11(11):7328-7334. doi:10.4103/jfmpc.jfmpc_1377_22
84. Kang HK, Kaur A, Dhiman A. Menopause-Specific Quality of Life of Rural Women. Indian J Community Med. 2021;46(2):273-276. doi:10.4103/ijcm.IJCM_665_20
85. Senthilvel S, Vasudevan S, Anju PS, Sukumaran A, Sureshbabu J. Assessment of Symptoms and Quality of Life among Postmenopausal Women in a Tertiary Care Hospital in Kochi, South India: A Hospital-based Descriptive Study. J Midlife Health. 2018;9(4):185-190. doi:10.4103/jmh.JMH_98_18
86. Karmakar N, Majumdar S, Dasgupta A, Das S. Quality of life among menopausal women: A community-based study in a rural area of West Bengal. J Midlife Health. 2017;8(1):21-27. doi:10.4103/jmh.JMH_78_16
87. Ganapathy T, SamiaSaud AF. Health-related quality of life among menopausal women. Archives of Medicine and Health Sciences. 2018; 6(1):16. doi: 10.4103/amhs.amhs_122_17
88. Sriprasert I, Pantasri T, Piyamongkol W, et al. An International Menopause Society study of vasomotor symptoms in Bangkok and Chiang Mai, Thailand. Climacteric. 2017;20(2):171-177.

doi:10.1080/13697137.2017.1284782
89. Tan MN, Kartal M, Guldal D. The effect of physical activity and body mass index on menopausal symptoms in Turkish women: a cross-sectional study in primary care. BMC Womens Health. 2014;14(1):38. Published 2014 Mar 6. doi:10.1186/1472-6874-14-38
90. Chopra S, Ranjan P, Verma A, et al. A cross sectional survey of 504 women regarding perceived risk factors and barriers to follow healthy lifestyle and association with sociodemographic factors and menopausal symptoms. Diabetes Metab Syndr. 2022;16(6):102529. doi:10.1016/j.dsx.2022.102529
91. Vaccaro CM, Capozzi A, Ettore G, et al. What women think about menopause: An Italian survey. Maturitas. 2021;147:47-52. doi:10.1016/j.maturitas.2021.03.007
92. Hachul H, Castro LS, Bezerra AG, et al. Hot flashes, insomnia, and the reproductive stages: a cross-sectional observation of women from the EPISONO study. J Clin Sleep Med. 2021;17(11):2257-2267. doi:10.5664/jcsm.9432
93. Saú HPF, Schmitt ACB, Cardoso MRA, Aldrighi JM. Prevalence of hot flashes in women of 40 to 65 years of age with metabolic syndrome. Rev Assoc Med Bras (1992). 2020;66(12):1628-1632. doi:10.1590/1806-9282.66.12.1628
94. Smith RL, Flaws JA, Gallicchio L. Does quitting smoking decrease the risk of midlife hot flashes? A longitudinal analysis. Maturitas. 2015;82(1):123-127. doi:10.1016/j.maturitas.2015.06.029
95. Zhao D, Lv G, Qi M, et al. The structure of menopausal syndrome: Using network analysis to understand unique symptomatic relationships. Int J Gynaecol Obstet. 2023;160(1):289-296. doi:10.1002/ijgo.14353
96. Han H, Xia X, Zheng H, et al. Factors associated with the high susceptibility to depression of women during the perimenopause. Brain Behav. 2023;13(1):e2826. doi:10.1002/brb3.2826
97. Ryu KJ, Park H, Park JS, et al. Vasomotor symptoms and carotid artery intima-media thickness among Korean midlife women. Maturitas. 2022;159:1-6. doi:10.1016/j.maturitas.2021.12.005
98. Salin S, Savukoski S, Tulppo M, et al. Does climacteric status impact regulation of the autonomic nervous system at the age of 46 years?. Climacteric. 2022;25(6):586-594. doi:10.1080/13697137.2022.2052842
99. Thakur J, Goswami M, Roy S. Do vasomotor and genitourinary symptoms of menopause vary between sedente and migrant groups? A study on the Oraon tribal populations of Eastern India. Am J Hum Biol. 2022;34(5):e23710. doi:10.1002/ajhb.23710
100. Tijerina A, Barrera Y, Solis-Pérez E, et al. Nutritional Risk Factors Associated with Vasomotor Symptoms in Women Aged 40-65 Years. Nutrients. 2022;14(13):2587. Published 2022 Jun 22. doi:10.3390/nu14132587
101. Koçoğlu F, Kocaöz S, Kara P, Aşcı Ö. Relationship between menopausal symptoms and sleep quality in women during the climacteric period: a cross-sectional study. J Obstet Gynaecol. 2022;42(6):2393-2398. doi:10.1080/01443615.2022.2062224
102. Arakane M, Castillo C, Rosero MF, Peñafiel R, Pérez-López FR, Chedraui P. Factors relating to insomnia during the menopausal transition as evaluated by the Insomnia Severity Index. Maturitas. 2011;69(2):157-161. doi:10.1016/j.maturitas.2011.02.015
103. AlDughaither A, AlMutairy H, AlAteeq M. Menopausal symptoms and quality of life among Saudi women visiting primary care clinics in Riyadh, Saudi Arabia. Int J Womens Health. 2015;7:645-653. Published 2015 Jun 29. doi:10.2147/IJWH.S84709
104. Ceylan B, Özerdoğan N. Menopausal symptoms and quality of life in Turkish women in the

climacteric period. Climacteric. 2014;17(6):705-712. doi:10.3109/13697137.2014.929108
105. Thakur M, Kaur M, Sinha AK. Assessment of menopausal symptoms in different transition phases using the Greene Climacteric Scale among rural women of North India. Ann Hum Biol. 2019;46(1):46-55. doi:10.1080/03014460.2019.1587508
106. Visvanathan K, Gallicchio L, Schilling C, et al. Cytochrome gene polymorphisms, serum estrogens, and hot flushes in midlife women. Obstet Gynecol. 2005;106(6):1372-1381. doi:10.1097/01.AOG.0000187308.67021.98
107. Kim MK, Seo SK, Chae HD, et al. Perceptions of Postmenopausal Symptoms and Treatment Options among Middle-Aged Korean Women. Yonsei Med J. 2017;58(3):533-539. doi:10.3349/ymj.2017.58.3.533
108. Sievert LL, Shreyer S, Boudreau A, Witkowski S, Brown DE. A comparison of stress, symptoms, physical activity, and adiposity among women at midlife before and during the pandemic. Womens Midlife Health. 2022;8(1):5. Published 2022 Apr 5. doi:10.1186/s40695-022-00075-w
109. Gerber LM, Sievert LL. Neighborhood disorder, exposure to violence, and perceived discrimination in relation to symptoms in midlife women. Womens Midlife Health. 2018;4:14. Published 2018 Oct 19. doi:10.1186/s40695-018-0043-0
110. Silvestrin T, Steenrod A, Coyne K, et al. Outcomes of implementing the women's health assessment tool and clinical decision support toolkit. Womens Health (Lond). 2016;12(3):313-323. doi:10.2217/whe.16.3
111. Ghazanfarpour M, Khadivzadeh T, Babakhanian M. Investigating the Relationship Between Sexual Function and Quality of Life in Menopausal Women. J Family Reprod Health. 2016;10(4):191-197.
112. Duffy OK, Iversen L, Hannaford PC. The impact and management of symptoms experienced at midlife: a community-based study of women in northeast Scotland. BJOG. 2012;119(5):554-564. doi:10.1111/j.1471-0528.2012.03276.x
113. Loutfy I, Abdel Aziz F, Dabbous NI, Hassan MH. Women's perception and experience of menopause: a community-based study in Alexandria, Egypt. East Mediterr Health J. 2006;12 Suppl 2:S93-S106.
114. Delavar MA, Hajiahmadi M. Age at menopause and measuring symptoms at midlife in a community in Babol, Iran. Menopause. 2011;18(11):1213-1218. doi:10.1097/gme.0b013e31821a7a3a
115. Chedraui P, Hidalgo L, Chavez D, Morocho N, Alvarado M, Huc A. Menopausal symptoms and associated risk factors among postmenopausal women screened for the metabolic syndrome. Arch Gynecol Obstet. 2007;275(3):161-168. doi:10.1007/s00404-006-0239-7
116. Chedraui P, Pérez-López FR, Schwager G, et al. Resilience and related factors during female Ecuadorian mid-life. Maturitas. 2012;72(2):152-156. doi:10.1016/j.maturitas.2012.03.004
117. Chedraui P, Pérez-López FR, Sánchez H, et al. Application of the 10-item Cervantes Scale among mid-aged Ecuadorian women for the assessment of menopausal symptoms. Maturitas. 2014;79(1):100-105. doi:10.1016/j.maturitas.2014.06.019
118. Suka M, Taniuchi A, Kudo Y, Sato S, Yoshida K, Ishizuka B. Self-assessed health and menopausal symptoms among 50-year-old Japanese women: cross-sectional surveys in Northern Kawasaki in 1998 and 2008. Menopause. 2010;17(1):166-173. doi:10.1097/gme.0b013e3181b6683f
119. Rahman S, Salehin F, Iqbal A. Menopausal symptoms assessment among middle age women

in Kushtia, Bangladesh. BMC Res Notes. 2011;4:188. Published 2011 Jun 15. doi:10.1186/1756-0500-4-188
120. Singh M. Early age of natural menopause in India, a biological marker for early preventive health programs. Climacteric. 2012;15(6):581-586. doi:10.3109/13697137.2011.643514
121. Aguilar-Zavala H, Pérez-Luque EL, Luna-Martínez F, et al. Symptoms at postmenopause: genetic and psychosocial factors. Menopause. 2012;19(10):1140-1145. doi:10.1097/gme.0b013e3182503bde
122. Agwu UM, Umeora OU, Ejikeme BN. Patterns of menopausal symptoms and adaptive ability in a rural population in South-east Nigeria. J Obstet Gynaecol. 2008;28(2):217-221. doi:10.1080/01443610801915637
123. Ahmed K, Jahan P, Nadia I, Ahmed F, Abdullah-Al-Emran. Assessment of Menopausal Symptoms among Early and Late Menopausal Midlife Bangladeshi Women and Their Impact on the Quality of Life. J Menopausal Med. 2016;22(1):39-46. doi:10.6118/jmm.2016.22.1.39
124. Gartoulla P, Bell RJ, Worsley R, Davis SR. Menopausal vasomotor symptoms are associated with poor self-assessed work ability. Maturitas. 2016;87:33-39. doi:10.1016/j.maturitas.2016.02.003
125. Berecki-Gisolf J, Begum N, Dobson AJ. Symptoms reported by women in midlife: menopausal transition or aging?. Menopause. 2009;16(5):1021-1029. doi:10.1097/gme.0b013e3181a8c49f
126. Bener A, Falah A. A measurement-specific quality-of-life satisfaction during premenopause, perimenopause and postmenopause in Arabian Qatari women. J Midlife Health. 2014;5(3):126-134. doi:10.4103/0976-7800.141190
127. Bener A, Rizk DE, Shaheen H, Micallef R, Osman N, Dunn EV. Measurement-specific quality-of-life satisfaction during the menopause in an Arabian Gulf country. Climacteric. 2000;3(1):43-49. doi:10.3109/13697130009167598
128. Im EO, Lee B, Chee W, Brown A, Dormire S. Menopausal symptoms among four major ethnic groups in the United States. West J Nurs Res. 2010;32(4):540-565. doi:10.1177/0193945909354343
129. Williams RE, Kalilani L, DiBenedetti DB, et al. Frequency and severity of vasomotor symptoms among peri- and postmenopausal women in the United States. Climacteric. 2008;11(1):32-43. doi:10.1080/13697130701744696
130. Ande AB, Omu OP, Ande OO, Olagbuji NB. Features and perceptions of menopausal women in Benin City, Nigeria. Ann Afr Med. 2011;10(4):300-304. doi:10.4103/1596-3519.87048
131. Andenæs R, Småstuen MC, Misvær N, Ribu L, Vistad I, Helseth S. Associations between menopausal hormone therapy and sleep disturbance in women during the menopausal transition and post-menopause: data from the Norwegian prescription database and the HUNT study. BMC Womens Health. 2020;20(1):64. Published 2020 Mar 30. doi:10.1186/s12905-020-00916-8
132. Khatoon A, Husain S, Husain S, Hussain S. An Overview of Menopausal Symptoms Using the Menopause Rating Scale in a Tertiary Care Center. J Midlife Health. 2018;9(3):150-154. doi:10.4103/jmh.JMH_31_18
133. Budakoğlu II, Ozcan C, Eroğlu D, Yanik F. Quality of life and postmenopausal symptoms among women in a rural district of the capital city of Turkey. Gynecol Endocrinol. 2007;23(7):404-409. doi:10.1080/09513590701444748

134. Sharanya Shre ES, Trout K, Singh SP, Singh AK, Mohan SK, Joshi A. Severity and clustering of menopausal symptoms among obese and nonobese postmenopausal women in India. J Pharm Bioallied Sci. 2016;8(2):106-111. doi:10.4103/0975-7406.171728
135. Damodaran P, Subramaniam R, Omar SZ, Nadkarni P, Paramsothy M. Profile of a menopause clinic in an urban population in Malaysia. Singapore Med J. 2000;41(9):431-435.
136. Zeleke BM, Bell RJ, Billah B, Davis SR. Vasomotor and sexual symptoms in older Australian women: a cross-sectional study. Fertil Steril. 2016;105(1):149-55.e1. doi:10.1016/j.fertnstert.2015.09.017
137. Masoudi M, Ahmadian H, Akbari M, et al. Correlation of the Severity of Physical and Psychological Symptoms and Severity of Insomnia in Postmenopausal Women. Journal of Kermanshah University of Medical Sciences. 2021;25(1). doi:25. 10.5812/jkums.110550.
138. S. Beura, L. Patnaik, M. Sahu, et al. Self-reported menopausal symptoms among postmenopausal women- A cross-sectional study in an urban slum of Odisha, India. European Journal of Molecular and Clinical Medicine. 2020;7(6) 849-859.
139. Thapa R, Yang Y. Menopausal symptoms and related factors among Cambodian women. Women Health. 2020;60(4):396-411. doi:10.1080/03630242.2019.1643815
140. Ibrahim ZM, Sayed Ahmed WA, El-Hamid SA. Prevalence of menopausal related symptoms and their impact on quality of life among Egyptian women. Clin Exp Obstet Gynecol. 2015;42(2):161-167.
141. Nusrat N, Nishat Z, Gulfareen H, Aftab M, Asia N. Knowledge, attitude and experience of menopause. J Ayub Med Coll Abbottabad. 2008;20(1):56-59.
142. K. Santhoshi Bhavani, S. Yamjala, P. Sadanandam, et al. Epidemiological Profile of Postmenopausal Women: A Cross Sectional Study. European Journal of Molecular and Clinical Medicine. 2022;9(1) 1294-1305.
143. Liu J, Eden J. Experience and attitudes toward menopause in Chinese women living in Sydney--a cross sectional survey. Maturitas. 2007;58(4):359-365. doi:10.1016/j.maturitas.2007.09.007
144. Fooladi E, Bell RJ, Masoumi M, Azizi M, Atarod Z, Davis SR. Bothersome menopausal symptoms amongst postmenopausal Iranian women. Climacteric. 2018;21(6):586-593. doi:10.1080/13697137.2018.1493452
145. Lee PS, Lee CL. Prevalence of symptoms and associated factors across menopause status in Taiwanese women. Menopause. 2020;28(2):182-188. Published 2020 Oct 12. doi:10.1097/GME.0000000000001662
146. M. Modoodi, F. Jalilvand, S. Zare, M, et al. Investigating the prevalence of menopausal complications and its related factors in women referred to Shahroud Health Centers in 2014. Revista Latinoamericana de Hipertension. 2020; 15(2):144-149
147. Kalhan M, Singhania K, Choudhary P, Verma S, Kaushal P, Singh T. Prevalence of Menopausal Symptoms and its Effect on Quality of Life among Rural Middle Aged Women (40-60 Years) of Haryana, India. Int J Appl Basic Med Res. 2020;10(3):183-188. doi:10.4103/ijabmr.IJABMR_428_19
148. Rindner L, Strömme G, Nordeman L, et al. Prevalence of somatic and urogenital symptoms as well as psychological health in women aged 45 to 55 attending primary health care: a cross-sectional study. BMC Womens Health. 2017;17(1):128. Published 2017 Dec 8. doi:10.1186/s12905-017-0480-1
149. H. M. Al-Musa, R. A. Ahmed, A. S. Alsamghan,et al. The prevalence of symptoms experienced

during menopause, influence of socio-demographic variables on symptoms and quality of life among women at Abha, Saudi Arabia. Biomedical Research (India). 2017;28(6):2587-2595

150. Moilanen J, Aalto AM, Hemminki E, Aro AR, Raitanen J, Luoto R. Prevalence of menopause symptoms and their association with lifestyle among Finnish middle-aged women. Maturitas. 2010;67(4):368-374. doi:10.1016/j.maturitas.2010.08.007
151. Bairy L, Adiga S, Bhat P, Bhat R. Prevalence of menopausal symptoms and quality of life after menopause in women from South India. Aust N Z J Obstet Gynaecol. 2009;49(1):106-109. doi:10.1111/j.1479-828X.2009.00955.x
152. Heinemann K, Rübig A, Strothmann A, Nahum GG, Heinemann LA. Prevalence and opinions of hormone therapy prior to the Women's Health Initiative: a multinational survey on four continents. J Womens Health (Larchmt). 2008;17(7):1151-1166. doi:10.1089/jwh.2007.0584
153. Uncu Y, Alper Z, Ozdemir H, Bilgel N, Uncu G. The perception of menopause and hormone therapy among women in Turkey. Climacteric. 2007;10(1):63-71. doi:10.1080/13697130601037324
154. Lawton BA, Rose SB, Cormack DM, Stanley J, Dowell AC. The menopause symptom profile of Maori and non-Maori women in New Zealand. Climacteric. 2008;11(6):467-474. doi:10.1080/13697130802351094
155. Liu J, Eden JA. The menopausal experience of Greek women living in Sydney. Menopause. 2008;15(3):476-481. doi:10.1097/gme.0b013e318154bda0
156. Lu J, Liu J, Eden J. The experience of menopausal symptoms by Arabic women in Sydney. Climacteric. 2007;10(1):72-79. doi:10.1080/13697130601159649
157. Saccomani S, Lui-Filho JF, Juliato CR, Gabiatti JR, Pedro AO, Costa-Paiva L. Does obesity increase the risk of hot flashes among midlife women?: a population-based study. Menopause. 2017;24(9):1065-1070. doi:10.1097/GME.0000000000000884
158. Im EO. Ethnic differences in symptoms experienced during the menopausal transition. Health Care Women Int. 2009;30(4):339-355. doi:10.1080/07399330802695002
159. Resmi S, Anil Bindu S, Benny P. V. Climacteric symptoms among women residing in a rural area of Kerala state-A cross-sectional study. Clinical Epidemiology and Global Health.2020;8(4): 1341-1344. doi: 10.1016/j.cegh.2020.05.008.
160. M. Ashrafi, S. K. Ashtiani, F. Malekzadeh, et al. Symptoms of natural menopause among Iranian women living in Tehran, Iran. Iranian Journal of Reproductive Medicine. 2010; 8(1):29-32
161. Ayranci U, Orsal O, Orsal O, Arslan G, Emeksiz DF. Menopause status and attitudes in a Turkish midlife female population: an epidemiological study. BMC Womens Health. 2010;10:1. Published 2010 Jan 11. doi:10.1186/1472-6874-10-1
162. Deveci SE, Açik Y, Dag DG, Tokdemir M, Gündoğdu C. The frequency of depression and menopause-related symptoms in postmenopausal women living in a province in Eastern Turkey, and the factors that affect depressive status. Med Sci Monit. 2010;16(4):PH40-PH47.
163. M. M. Sagdeo, D. Arora. Menopausal symptoms: A comparative study in rural and Urban Women. JK Science. 2011; 13(1):23-26
164. Oğurlu N, Küçük M, Aksu H. Influence of employment status on menopausal symptoms. Int J Gynaecol Obstet. 2011;112(3):204-207. doi:10.1016/j.ijgo.2010.10.010
165. Wang X, Zhao G, Di J, Wang L, Zhang X. Prevalence and risk factors for depressive and anxiety symptoms in middle-aged Chinese women: a community-based cross-sectional study. BMC

Womens Health. 2022;22(1):319. Published 2022 Jul 29. doi:10.1186/s12905-022-01908-6
166. Anolue FC, Dike E, Adogu P, Ebirim C. Women's experience of menopause in rural communities in Orlu, Eastern Nigeria. Int J Gynaecol Obstet. 2012;118(1):31-33. doi:10.1016/j.ijgo.2012.02.014
167. Mahajan N, Aggarwal M, Bagga A. Health issues of menopausal women in North India. J Midlife Health. 2012;3(2):84-87. doi:10.4103/0976-7800.104467
168. Mustafa GN, Sabir JM. Perception and experience regarding menopause among menopaused women attending teaching hospitals in Erbil City. Glob J Health Sci. 2012;4(3):170-178. Published 2012 Apr 28. doi:10.5539/gjhs.v4n3p170
169. Nappi RE, Kokot-Kierepa M. Vaginal Health: Insights, Views & Attitudes (VIVA) - results from an international survey. Climacteric. 2012;15(1):36-44. doi:10.3109/13697137.2011.647840
170. Sassoon SA, de Zambotti M, Colrain IM, Baker FC. Association between personality traits and DSM-IV diagnosis of insomnia in peri- and postmenopausal women. Menopause. 2014;21(6):602-611. doi:10.1097/GME.0000000000000192
171. Liu M, Wang Y, Li X, et al. A health survey of Beijing middle-aged registered nurses during menopause. Maturitas. 2013;74(1):84-88. doi:10.1016/j.maturitas.2012.10.006
172. Mohamed H, Lamadah Sahar, Al Z, et al. Quality of life among menopausal women. International Journal of Reproduction, Contraception, Obstetrics and Gynecology. 2014;3(1).doi:10.5455/2320-1770.ijrcog20140906.
173. Hashemzadeh M, Keramat A, Mollaahmadi L, Ghiasi A, Haseli A. The Role of Lifestyle in Developing and Maintaining Vasomotor Symptoms: A Cross-Sectional Study among Iranian Postmenopausal Women. J Midlife Health. 2019;10(4):184-191. doi:10.4103/jmh.JMH_64_19
174. Hestiantoro, A., Jasirwan, S.O., Wiwie, M., Shadrina, A., Ibrahim, N. and Astuti, B.P.K. Low estradiol levels escalate menopausal symptoms leading to mild cognitive impairment in postmenopausal women. Medical Journal of Indonesia. 2019;28(1):40-6. doi:https://doi.org/10.13181/mji.v28i1.2447.
175. Kong F, Wang J, Zhang C, Feng X, Zhang L, Zang H. Assessment of sexual activity and menopausal symptoms in middle-aged Chinese women using the Menopause Rating Scale. Climacteric. 2019;22(4):370-376. doi:10.1080/13697137.2018.1547702
176. Sharifi N, Najar S, Rezaii N, et al. The Association Between Menopausal Symptoms and General Health Among Iranian Women With Menopause: A Cross-Sectional Study. International Journal of Women’s Health and Reproduction Sciences. 2019;7(2):196–203. doi: 10.15296/ijwhr.2019.33
177. Taavoni S, Ekbatani NN, Haghani H. Postmenopausal Women's Quality of Sleep and its Related Factors. J Midlife Health. 2015;6(1):21-25. doi:10.4103/0976-7800.153611
178. Li S, Holm K, Gulanick M, Lanuza D. Perimenopause and the quality of life. Clin Nurs Res. 2000;9(1):6-26. doi:10.1177/105477380000900102
179. Pan HA, Wu MH, Hsu CC, Yao BL, Huang KE. The perception of menopause among women in Taiwan. Maturitas. 2002;41(4):269-274. doi:10.1016/s0378-5122(01)00279-1
180. Kasuga M, Makita K, Ishitani K, et al. Relation between climacteric symptoms and ovarian hypofunction in middle-aged and older Japanese women. Menopause. 2004;11(6 Pt 1):631-638. doi:10.1097/01.gme.0000119984.87302.30
181. Kaur S, Walia I, Singh A. How menopause affects the lives of women in suburban Chandigarh, India. Climacteric. 2004;7(2):175-180. doi:10.1080/13697130410001713779

182. Yahya S, Rehan N. Age, pattern and symptoms of menopause among rural women of Lahore. J Ayub Med Coll Abbottabad. 2002;14(3):9-12.
183. Gupta P, Sturdee DW, Hunter MS. Mid-age health in women from the Indian subcontinent (MAHWIS): general health and the experience of menopause in women. Climacteric. 2006;9(1):13-22. doi:10.1080/13697130500515776
184. Inayat K, Danish N, Hassan L. Symptoms Of Menopause In Peri And Postmenopausal Women And Their Attitude Towards Them. J Ayub Med Coll Abbottabad. 2017;29(3):477-480.
185. Mathialagan S, Ramasamy S, Nagandla K, Siew WF, Sreeramareddy CT. Menopause Rating Scale (MRS) in the Malay language-translation and validation in a multiethnic population of Selangor, Malaysia. BMC Womens Health. 2022;22(1):347. Published 2022 Aug 17. doi:10.1186/s12905-022-01922-8
186. Canário AC, Cabral PU, Spyrides MH, Giraldo PC, Eleutério J Jr, Gonçalves AK. The impact of physical activity on menopausal symptoms in middle-aged women. Int J Gynaecol Obstet. 2012;118(1):34-36. doi:10.1016/j.ijgo.2012.02.016
187. Rahman, M. M., M. A. Rahman, K. Alo, and F. Mehrin. Impact of Body Mass Index (BMI) and Physical Activities Among Menopausal Women: BMI and Physical Activities Among Menopausal Women. Bangladesh Medical Research Council Bulletin. 2020;46(2):109-14, doi:10.3329/bmrcb.v46i2.49020.
188. Abedzadeh-kalahroud M, Taebi M, Sadat Z. Prevalence and Severity of Menopause Symptoms and Related Factors among Women 40-60 Years in Kashan Iran. Nursing and Midwifery Studies, 2012;1:88-93. doi:10.5812/nms.8358
189. Diyu IANP, Satriani NLA. Menopausal symptoms in women aged 40-65 years in Indonesia. Ijhms.2022;5(2):169-76. doi:https://sloap.org/journal/index.php/ijhms/article/view/1896
190. Zhang JP, Wang YQ, Yan MQ, Li ZA, Du XP, Wu XQ. Menopausal Symptoms and Sleep Quality During Menopausal Transition and Postmenopause. Chin Med J (Engl). 2016;129(7):771-777. doi:10.4103/0366-6999.178961
191. Ryu KJ, Park H, Kim YJ, et al. Comparison of various menopausal symptoms and risk factor analysis in Korean women according to stage of menopause. Maturitas. 2020;140:41-48. doi:10.1016/j.maturitas.2020.05.023
192. Ma, Mingze et al. "A health survey of perimenopausal syndrome and mood disorders in perimenopause : a cross-sectional study in Shanghai." (2017).
193. Fu JX, Luo Y, Chen MZ, et al. Associations among menopausal status, menopausal symptoms, and depressive symptoms in midlife women in Hunan Province, China. Climacteric. 2020;23(3):259-266. doi:10.1080/13697137.2019.1703936
194. Liu X, Fu X, Du R, Chen Z, Sun J, Ding Y. Epidemiology and Risk Factors of Menopause Syndrome Among Uyghur, Han, and Kazak Women in Xinjiang, China. Med Sci Monit. 2018;24:8950-8958. Published 2018 Dec 10. doi:10.12659/MSM.909954
195. Li RX, Ma M, Xiao XR, Xu Y, Chen XY, Li B. Perimenopausal syndrome and mood disorders in perimenopause: prevalence, severity, relationships, and risk factors. Medicine (Baltimore). 2016;95(32):e4466. doi:10.1097/MD.0000000000004466
196. Joshi M, Nair S. Epidemiological Study to Assess the Menopausal Problems during Menopausal Transition in Middle Age Women of Vadodara, Gujarat, India. Indian Journal of Obstetrics and Gynecology Research. 2015;2:163. doi:10.5958/2394-2754.2015.00007.7.
197. Ghimire N, Dhakal P, Norrish D, et al. Menopausal Health Status of Women of Kapilvastu

District of Nepal. J Nepal Health Res Counc. 2015;13(31):182-187.
198. Joseph N, Nagaraj K, Saralaya V, Nelliyanil M, Rao PJ. Assessment of menopausal symptoms among women attending various outreach clinics in South Canara District of India. J Midlife Health. 2014;5(2):84-90. doi:10.4103/0976-7800.133996
199. Dasgupta D, Ray S. Vasomotor and urogenital problems at midlife: a study on rural and urban women in India. Ann Hum Biol. 2015;42(3):268-275. doi:10.3109/03014460.2014.941397
200. Sievert LL, Begum K, Sharmeen T, Chowdhury O, Muttukrishna S, Bentley G. Patterns of occurrence and concordance between subjective and objective hot flashes among Muslim and Hindu women in Sylhet, Bangladesh. Am J Hum Biol. 2008;20(5):598-604. doi:10.1002/ajhb.20785
201. Anil, B. S., Anitha, B., Jose, J. Prevalence of menopausal symptoms among women (menopausal for< 5 years) in a rural area in Kottayam, Kerala, India. Journal of Evolution of Medical and Dental Sciences, 2014;3(17), 4648-4657.doi: 10.14260/jemds/2014/2490
202. Srivastava M, Srivastava R, Pandit B. Presentation of menopausal symptoms: A village based community study. Asian Journal of Medical Sciences.2014; 6. doi:10.3126/ajms.v6i1.8431.
203. Rokhade, Chandramati J. et al. HEALTH RELATED COMPLICATIONS IN MENOPAUSAL WOMEN OF NORTH KARNATAKA. 2013.
204. Sun D, Shao H, Li C, Tao M. Sleep disturbance and correlates in menopausal women in Shanghai. J Psychosom Res. 2014;76(3):237-241. doi:10.1016/j.jpsychores.2013.12.002
205. Wong BWX, Chan YH, Kramer MS, et al. Factors associated with poor sleep quality in midlife Singaporean women: The Integrated Women's Health program (IWHP). Sleep Med X. 2022;5:100060. Published 2022 Dec 9. doi:10.1016/j.sleepx.2022.100060
206. Espírito Santo J, Hita-Contreras F, Marques de Loureiro NE, et al. Associations between the impact of menopausal symptoms and fall-related self-efficacy. Menopause. 2023;30(4):421-426. doi:10.1097/GME.0000000000002151
207. Xu Q, Zhao Y, Chen H, Jing J. Exploring Sleep Quality and Related Factors in Chinese Midlife Women. Health Care Women Int. 2016;37(6):620-635. doi:10.1080/07399332.2015.1037443
208. Lerner-Geva L, Boyko V, Blumstein T, Benyamini Y. The impact of education, cultural background, and lifestyle on symptoms of the menopausal transition: the Women's Health at Midlife Study. J Womens Health (Larchmt). 2010;19(5):975-985. doi:10.1089/jwh.2009.1381
209. Sweed HS, Elawam AE, Nabeel AM, Mortagy K. Postmenopausal symptoms among Egyptian geripausal women. East Mediterr Health J. 2012;18(3):213-220. doi:10.26719/2012.18.3.213
210. Taher YA, Ben Emhemed HM, Tawati AM. Menopausal age, related factors and climacteric symptoms in Libyan women. Climacteric. 2013;16(1):179-184. doi:10.3109/13697137.2012.682107
211. Ryan J, Burger HG, Szoeke C, et al. A prospective study of the association between endogenous hormones and depressive symptoms in postmenopausal women. Menopause. 2009;16(3):509-517. doi:10.1097/gme.0b013e31818d635f
212. Beigi M, Fahami F. A Comparative study on sexual dysfunctions before and after menopause. Iran J Nurs Midwifery Res. 2012;17(2 Suppl 1):S72-S75.
213. G K P, Arounassalame B. The quality of life during and after menopause among rural women. J Clin Diagn Res. 2013;7(1):135-139. doi:10.7860/JCDR/2012/4910.2688
214. Bernis C, Reher DS. Environmental contexts of menopause in Spain: comparative results from recent research. Menopause. 2007;14(4):777-787. doi:10.1097/gme.0b013e31803020ff

215. Dibonaventura MD, Chandran A, Hsu MA, Bushmakin A. Burden of vasomotor symptoms in France, Germany, Italy, Spain, and the United Kingdom. Int J Womens Health. 2013;5:261-269. Published 2013 May 24. doi:10.2147/IJWH.S39027
216. Freeman EW, Sammel MD, Lin H. Temporal associations of hot flashes and depression in the transition to menopause. Menopause. 2009;16(4):728-734. doi:10.1097/gme.0b013e3181967e16
217. Seib C, Anderson D, Lee K. Prevalence and correlates of sleep disturbance in postmenopausal women: the Australian Healthy Aging of Women (HOW) Study. J Womens Health (Larchmt). 2014;23(2):151-158. doi:10.1089/jwh.2013.4472
218. Chedraui P, Aguirre W, Hidalgo L, Fayad L. Assessing menopausal symptoms among healthy middle aged women with the Menopause Rating Scale. Maturitas. 2007;57(3):271-278. doi:10.1016/j.maturitas.2007.01.009
219. Ahlawat P, Singh MM, Garg S, Mala YM. Prevalence of Depression and its Association with Sociodemographic Factors in Postmenopausal Women in an Urban Resettlement Colony of Delhi. J Midlife Health. 2019;10(1):33-36. doi:10.4103/jmh.JMH_66_18
220. Bansal P, Chaudhary A, Soni RK, Sharma S, Gupta VK, Kaushal P. Depression and anxiety among middle-aged women: A community-based study. J Family Med Prim Care. 2015;4(4):576-581. doi:10.4103/2249-4863.174297
221. Dutta R, Rajendran P, Ramya S, et al. Prevalence of depression among the Post-Menopausal women in the field practice area of saveetha medical college and hospital, Thirumazhisai, Tamil Nadu. Indian Journal of Public Health Research and Development,2018; 9, 175-179.
222. Nayak S, Binil V, Christabel S.Depressive Symptoms and Bio-psychosocial Problems among Postmenopausal Women of Udupi District, Karnataka, India.J Clin of Diagn Res.2019; 13(1):VC01-VC04. doi:10.7860/JCDR/2019/38164/12455
223. Ps A, Das S, Philip S, et al. Prevalence of depression among middle aged women in the rural area of Kerala. Asian J Psychiatr. 2017;29:154-159. doi:10.1016/j.ajp.2017.05.016
224. Alam MM, Ahmed S, Dipti RK, Siddiquee RE, Hawlader MDH. The prevalence and associated factors of depression during pre-, peri-, and post-menopausal period among the middle-aged women of Dhaka city. Asian J Psychiatr. 2020;54:102312. doi:10.1016/j.ajp.2020.102312
225. Shin C, Lee S, Lee T, et al. Prevalence of insomnia and its relationship to menopausal status in middle-aged Korean women. Psychiatry Clin Neurosci. 2005;59(4):395-402. doi:10.1111/j.1440-1819.2005.01391.x
226. Fabbrini M, AricÃ I, Tramonti F, et al. Sleep disorders in menopause: results from an Italian Multicentric Study. Arch Ital Biol. 2015;153(2-3):204-213. doi:10.12871/0003982920152345
227. Luo M, Li J, Tang R, et al. Insomnia symptoms in relation to menopause among middle-aged Chinese women: Findings from a longitudinal cohort study. Maturitas. 2020;141:1-8. doi:10.1016/j.maturitas.2020.06.010
228. Geng L, Zheng Y, Zhou Y, Li C, Tao M. The prevalence and determinants of genitourinary syndrome of menopause in Chinese mid-life women: a single-center study. Climacteric. 2018;21(5):478-482. doi:10.1080/13697137.2018.1458832
229. Moral E, Delgado JL, Carmona F, et al. Genitourinary syndrome of menopause. Prevalence and quality of life in Spanish postmenopausal women. The GENISSE study. Climacteric. 2018;21(2):167-173. doi:10.1080/13697137.2017.1421921
230. Ahmed HM, Osman VA, Al-Alaf SK, Al-Tawil NG. Prevalence of urinary incontinence and

probable risk factors in a sample of kurdish women. Sultan Qaboos Univ Med J. 2013;13(2):269-274. doi:10.12816/0003233
231. Xu C, Chen M, Fu J, Meng Y, Qin S, Luo Y. Urinary incontinence status and risk factors in women aged 50-70 years: a cross-sectional study in Hunan, China. Int Urogynecol J. 2021;32(1):95-102. doi:10.1007/s00192-020-04259-8
232. Islam RM, Bell RJ, Hossain MB, Davis SR. Types of urinary incontinence in Bangladeshi women at midlife: Prevalence and risk factors. Maturitas. 2018;116:18-23. doi:10.1016/j.maturitas.2018.07.012
233. Yagmur Y, Gul S. Urinary incontinence in women aged 40 and older: Its prevalence, risk factors, and effect on quality of life. Niger J Clin Pract. 2021;24(2):186-192. doi:10.4103/njcp.njcp_626_18
234. Chung KF, Tang MK. Subjective sleep disturbance and its correlates in middle-aged Hong Kong Chinese women. Maturitas. 2006;53(4):396-404. doi:10.1016/j.maturitas.2005.07.001
235. Hsu HC, Lin MH. Exploring quality of sleep and its related factors among menopausal women. J Nurs Res. 2005;13(2):153-164. doi:10.1097/01.jnr.0000387536.60760.4e
236. Ward T, Scheid V, Tuffrey V. Women's mid-life health experiences in urban UK: an international comparison. Climacteric. 2010;13(3):278-288. doi:10.3109/13697130903197479
237. Pandey A, Karki C, Shrivastava VR, Shrestha D, Gautam P. Study of Menopausal Symptoms using Menopause Rating Scale at a Tertiary Care Center: A Descriptive Cross-sectional Study. JNMA J Nepal Med Assoc. 2020;58(230):725-728. Published 2020 Oct 15. doi:10.31729/jnma.5200
238. Abdelaziz EM, Elsharkawy NB, Mohamed SM. The relationship between sleep quality and menopausal symptoms among postmenopausal women in Saudi Arabia. Saudi Med J. 2022;43(4):401-407. doi:10.15537/smj.2022.43.4.20210682
239. Liao K, Gu Y, Liu M, et al. Association of dietary patterns with depressive symptoms in Chinese postmenopausal women. Br J Nutr. 2019;122(10):1168-1174. doi:10.1017/S0007114519001867
240. Sievert LL, Morrison L, Brown DE, Reza AM. Vasomotor symptoms among Japanese-American and European-American women living in Hilo, Hawaii. Menopause. 2007;14(2):261-269. doi:10.1097/01.gme.0000233496.13088.24
241. Sievert LL, Espinosa-Hernandez G. Attitudes toward menopause in relation to symptom experience in Puebla, Mexico. Women Health. 2003;38(2):93-106. doi:10.1300/J013v38n02_07
242. Obermeyer CM, Reynolds RF, Price K, Abraham A. Therapeutic decisions for menopause: results of the DAMES project in central Massachusetts. Menopause. 2004;11(4):456-465. doi:10.1097/01.gme.0000109318.11228.da
243. Keenan NL, Mark S, Fugh-Berman A, Browne D, Kaczmarczyk J, Hunter C. Severity of menopausal symptoms and use of both conventional and complementary/alternative therapies. Menopause. 2003;10(6):507-515. doi:10.1097/01.GME.0000064865.58809.3E
244. Jansson C, Johansson S, Lindh-Astrand L, Hoffmann M, Hammar M. The prevalence of symptoms possibly related to the climacteric in pre- and postmenopausal women in Linköping, Sweden. Maturitas. 2003;45(2):129-135. doi:10.1016/s0378-5122(03)00127-0
245. Zhao G, Wang L, Yan R, Dennerstein L. Menopausal symptoms: experience of Chinese women. Climacteric. 2000;3(2):135-144. doi:10.3109/13697130009167615

246. Lam PM, Leung TN, Haines C, Chung TK. Climacteric symptoms and knowledge about hormone replacement therapy among Hong Kong Chinese women aged 40-60 years. Maturitas. 2003;45(2):99-107. doi:10.1016/s0378-5122(03)00090-2
247. Gollschewski S, Anderson D, Skerman H, Lyons-Wall P. The use of complementary and alternative medications by menopausal women in South East Queensland. Womens Health Issues. 2004;14(5):165-171. doi:10.1016/j.whi.2004.08.001
248. Biri A, Bakar C, Maral I, Karabacak O, Bumin MA. Women with and without menopause over age of 40 in Turkey: consequences and treatment options. Maturitas. 2005;50(3):167-176. doi:10.1016/j.maturitas.2004.05.013
249. Obermeyer CM, Schulein M, Hajji N, Azelmat M. Menopause in Morocco: symptomatology and medical management. Maturitas. 2002;41(2):87-95. doi:10.1016/s0378-5122(01)00289-4
250. Lin HL, Hsiao MC, Liu YT, Chang CM. Perimenopause and incidence of depression in midlife women: a population-based study in Taiwan. Climacteric. 2013;16(3):381-386. doi:10.3109/13697137.2012.707706
251. Loh FH, Khin LW, Saw SM, Lee JJ, Gu K. The age of menopause and the menopause transition in a multiracial population: a nation-wide Singapore study. Maturitas. 2005;52(3-4):169-180. doi:10.1016/j.maturitas.2004.11.004
252. Chaopotong P, Titapant V, Boriboonhirunsarn D. Menopausal symptoms and knowledge towards daily life and hormone replacement therapy among menopausal women in Bangkok. J Med Assoc Thai. 2005;88(12):1768-1774.
253. Zhu Y, Wei J, Yang X, Zhu W, Zhang W. Investigation on prevalence and risk factors associated with genitourinary syndrome of menopause in middle-aged and older women in Beijing community: a cross sectional study. BMC Womens Health. 2022;22(1):558. Published 2022 Dec 30. doi:10.1186/s12905-022-02099-w
254. Huang KE, Xu L, I NN, Jaisamrarn U. The Asian Menopause Survey: knowledge, perceptions, hormone treatment and sexual function. Maturitas. 2010;65(3):276-283. doi:10.1016/j.maturitas.2009.11.015
255. Hunter MS, Gupta P, Chedraui P, et al. The International Menopause Study of Climate, Altitude, Temperature (IMS-CAT) and vasomotor symptoms. Climacteric. 2013;16(1):8-16. doi:10.3109/13697137.2012.699563
256. Anderson D, Yoshizawa T, Gollschewski S, Atogami F, Courtney M. Menopause in Australia and Japan: effects of country of residence on menopausal status and menopausal symptoms. Climacteric. 2004;7(2):165-174. doi:10.1080/13697130410001713760
257. Gold EB, Colvin A, Avis N, et al. Longitudinal analysis of the association between vasomotor symptoms and race/ethnicity across the menopausal transition: study of women's health across the nation. Am J Public Health. 2006;96(7):1226-1235. doi:10.2105/AJPH.2005.066936
258. Afshari P, Manochehri S, Tadayon M. Prevalence of Depression in Postmenopausal Women. Jundishapur Journal of Chronic Disease Care. 2015,4. doi:10.5812/jjcdc.27521v2.
259. Gonçalves B, Fagulha T, Ferreira A. A population-based assessment of the relationship between menopausal and depressive symptoms in Portuguese women. Health Care Women Int. 2013;34(1):86-100. doi:10.1080/07399332.2012.721413
260. Onya, O. and Otorkpa, C. Prevalence and Socio-Demographic Determinants of Depression in Women: A Comparison between Pre-Menopausal and Post-Menopausal Attendees of the General Outpatient Department in Fmc Lokoja. Open Journal of Depression,2018;7:51-59. doi:

10.4236/ojd.2018.73004.

261. Yen JY, Yang MS, Wang MH, Lai CY, Fang MS. The associations between menopausal syndrome and depression during pre-, peri-, and postmenopausal period among Taiwanese female aborigines. Psychiatry Clin Neurosci. 2009;63(5):678-684. doi:10.1111/j.1440-1819.2009.02001.x
262. Timur S, Sahin NH. The prevalence of depression symptoms and influencing factors among perimenopausal and postmenopausal women. Menopause. 2010;17(3):545-551. doi:10.1097/gme.0b013e3181cf8997
263. Zang H, He L, Chen Y, Ge J, Yao Y. The association of depression status with menopause symptoms among rural midlife women in China. Afr Health Sci. 2016;16(1):97-104. doi:10.4314/ahs.v16i1.13
264. Masjoudi M, Amjadi MA, Leyli EKN. Severity and Frequency of Menopausal Symptoms in Middle Aged Women, Rasht, Iran. J Clin Diagn Res. 2017;11(8):QC17-QC21. doi:10.7860/JCDR/2017/26994.10515
265. Kapoor E, Okuno M, Miller VM, et al. Association of adverse childhood experiences with menopausal symptoms: Results from the Data Registry on Experiences of Aging, Menopause and Sexuality (DREAMS). Maturitas. 2021;143:209-215. doi:10.1016/j.maturitas.2020.10.006
266. Durairaj A, Venkateshvaran S. Determinants of Menopausal Symptoms and Attitude Towards Menopause Among Midlife Women: A Cross-Sectional Study in South India. Cureus. 2022;14(9):e28718. Published 2022 Sep 3. doi:10.7759/cureus.28718
267. Bala K, Gupta R, Gupta RK, Akhtar N. Knowledge and Perceptions Regarding Climacteric Among Rural Women in Jammu District of UT of J and K, India: A Cross-Sectional Study. J Midlife Health. 2022;13(2):163-168. doi:10.4103/jmh.jmh_217_21
268. Stefanopoulou E, Shah D, Shah R, Gupta P, Sturdee DW, Hunter MS. An International Menopause Society study of climate, altitude, temperature (IMS-CAT) and vasomotor symptoms in urban Indian regions. Climacteric. 2014;17(4):417-424. doi:10.3109/13697137.2013.852169
269. Abou-Raya S, Sadek S, AbelBaqy M, et al. Relationship between sociodemographic, reproductive, and lifestyle factors and the severity of menopausal symptoms among Egyptian women in Alexandria. Menopause. 2016;23(8):888-893. doi:10.1097/GME.0000000000000635
270. Krajewska-Ferishah K, Kułak-Bejda A, Szyszko-Perłowska A, Shpakou A, Van Damme-Ostapowicz K, Chatzopulu A. Risk of Depression during Menopause in Women from Poland, Belarus, Belgium, and Greece. J Clin Med. 2022;11(12):3371. Published 2022 Jun 12. doi:10.3390/jcm11123371
271. Ogwumike OO, Kaka B, Adegbemigun O, Abiona T. Health-related and socio-demographic correlates of physical activity level amongst urban menopausal women in Nigeria. Maturitas. 2012;73(4):349-353. doi:10.1016/j.maturitas.2012.09.010
272. Ornat L, Martínez-Dearth R, Muñoz A, et al. Sexual function, satisfaction with life and menopausal symptoms in middle-aged women. Maturitas. 2013;75(3):261-269. doi:10.1016/j.maturitas.2013.04.007
273. Wang HL, Booth-LaForce C, Tang SM, Wu WR, Chen CH. Depressive symptoms in Taiwanese women during the peri- and post-menopause years: associations with demographic, health, and psychosocial characteristics. Maturitas. 2013;75(4):355-360.

doi:10.1016/j.maturitas.2013.04.021
274. Gao HL, Lin SQ, Wei Y, Chen Y, Wu ZL. The effect of age and menopausal status on musculoskeletal symptoms in Chinese women aged 35-64 years. Climacteric. 2013;16(6):639-645. doi:10.3109/13697137.2013.769095
275. Sierra B, Hidalgo LA, Chedraui PA. Measuring climacteric symptoms in an Ecuadorian population with the Greene Climacteric Scale. Maturitas. 2005;51(3):236-245. doi:10.1016/j.maturitas.2004.08.003
276. Castelo-Branco C, Palacios S, Mostajo D, Tobar C, von Helde S. Menopausal transition in Movima women, a Bolivian Native-American. Maturitas. 2005;51(4):380-385. doi:10.1016/j.maturitas.2004.09.004
277. Ahn S, Song R. Bone mineral density and perceived menopausal symptoms: factors influencing low back pain in postmenopausal women. J Adv Nurs. 2009;65(6):1228-1236. doi:10.1111/j.1365-2648.2009.04983.x
278. Jin F, Tao M, Teng Y, Shao H, Li C, Mills E. Knowledge and attitude towards menopause and hormone replacement therapy in Chinese women. Gynecol Obstet Invest. 2015;79(1):40-45. doi:10.1159/000365172
279. Pérez-López FR, Fernández-Alonso AM, Pérez-Roncero G, Chedraui P, Monterrosa-Castro A, Llaneza P. Assessment of menopause-related symptoms in mid-aged women with the 10-item Cervantes Scale. Maturitas. 2013;76(2):151-154. doi:10.1016/j.maturitas.2013.07.002
280. Huang Z, Shi J, Liu W, Wei S, Zhang Z. The influence of educational level in peri-menopause syndrome and quality of life among Chinese women. Gynecol Endocrinol. 2020;36(11):991-996. doi:10.1080/09513590.2020.1781081
281. Dasgupta D, Ray S. Attitude toward menopause and aging: a study on postmenopausal women of West Bengal. J Women Aging. 2013;25(1):66-79. doi:10.1080/08952841.2012.720203
282. Huseth-Zosel A, Strand M, Perry J. Socioeconomic differences in the menopausal experience of Chinese women. Post Reprod Health. 2014;20(3):98-103. doi:10.1177/2053369114544729
283. Ornat L, Martínez-Dearth R, Chedraui P, Pérez-López FR. Assessment of subjective sleep disturbance and related factors during female mid-life with the Jenkins Sleep Scale. Maturitas. 2014;77(4):344-350. doi:10.1016/j.maturitas.2014.01.005
284. Legorreta D, Montaño JA, Hernández I, Salinas C, Hernández-Bueno JA; AMEC Research Committee. Age at menopause, motives for consultation and symptoms reported by 40-59-year-old Mexican women. Climacteric. 2013;16(4):417-425. doi:10.3109/13697137.2012.696288
285. Aydin Y, Hassa H, Oge T, Yalcin OT, Mutlu FS. Frequency and determinants of urogenital symptoms in postmenopausal Islamic women. Menopause. 2014;21(2):182-187. doi:10.1097/GME.0b013e3182937966
286. Zhang L, Ruan X, Cui Y, Gu M, Mueck AO. Menopausal Symptoms and Associated Social and Environmental Factors in Midlife Chinese Women. Clin Interv Aging. 2020;15:2195-2208. Published 2020 Nov 16. doi:10.2147/CIA.S278976
287. Wu S, Shi Y, Zhao Q, Men K. The relationship between physical activity and the severity of menopausal symptoms: a cross-sectional study. BMC Womens Health. 2023;23(1):212. Published 2023 Apr 28. doi:10.1186/s12905-023-02347-7
288. Shea AK, Sohel N, Gilsing A, Mayhew AJ, Griffith LE, Raina P. Depression, hormone therapy, and the menopausal transition among women aged 45 to 64 years using Canadian Longitudinal

Study on aging baseline data. Menopause. 2020;27(7):763-770. doi:10.1097/GME.0000000000001540

289. Abdullah B, Moize B, Ismail BA, Zamri M, Mohd Nasir NF. Prevalence of menopausal symptoms, its effect to quality of life among Malaysian women and their treatment seeking behaviour. Med J Malaysia. 2017;72(2):94-99.

290. Chu K, Shui J, Ma L, et al. Biopsychosocial risk factors of depression during menopause transition in southeast China. BMC Womens Health. 2022;22(1):273. Published 2022 Jul 5. doi:10.1186/s12905-022-01710-4

291. Barghandan N, Dolatkhah N, Eslamian F, Ghafarifar N, Hashemian M. Association of depression, anxiety and menopausal-related symptoms with demographic, anthropometric and body composition indices in healthy postmenopausal women. BMC Womens Health. 2021;21(1):192. Published 2021 May 7. doi:10.1186/s12905-021-01338-w

292. Hickey M, Riach K, Kachouie R, Jack G. No sweat: managing menopausal symptoms at work. J Psychosom Obstet Gynaecol. 2017;38(3):202-209. doi:10.1080/0167482X.2017.1327520

293. Blell MT. Menopausal symptoms among British Pakistani women: a critique of the standard checklist approach. Menopause. 2015;22(1):79-87. doi:10.1097/GME.0000000000000256

294. Dienye PO, Judah F, Ndukwu G. Frequency of symptoms and health seeking behaviours of menopausal women in an out-patient clinic in Port Harcourt, Nigeria. Glob J Health Sci. 2013;5(4):39-47. Published 2013 Mar 18. doi:10.5539/gjhs.v5n4p39

295. Whiteley J, DiBonaventura Md, Wagner JS, Alvir J, Shah S. The impact of menopausal symptoms on quality of life, productivity, and economic outcomes. J Womens Health (Larchmt). 2013;22(11):983-990. doi:10.1089/jwh.2012.3719

296. Bener A, Saleh NM, Bakir A, Bhugra D. Depression, Anxiety, and Stress Symptoms in Menopausal Arab Women: Shedding More Light on a Complex Relationship. Ann Med Health Sci Res. 2016;6(4):224-231. doi:10.4103/amhsr.amhsr_341_15

297. Olowokere AE, Tope-Ajayi TO, Komolafe AO, Olajubu AO. Lifestyle practices and menopause-related symptoms among women in rural communities of Ado-Ekiti local government area, Nigeria. Post Reprod Health. 2021;27(2):66-76. doi:10.1177/2053369120971427

298. Zhu Y, Yang X, Fan X, et al. Decreased Sexual Desire among Middle-Aged and Old Women in China and Factors Influencing It: A Questionnaire-Based Study. Evid Based Complement Alternat Med. 2021;2021:6649242. Published 2021 May 25. doi:10.1155/2021/6649242

299. Zhang Q, Li F, Yu Y, Yu X, Sheng Q, Zhang X. Differential factors associated with hot flashes in Chinese perimenopausal and postmenopausal women. Maturitas. 2009;63(1):94-98. doi:10.1016/j.maturitas.2009.02.007

300. Salazar-Pousada D, Monterrosa-Castro A, Ojeda E, et al. Evaluation of depressive symptoms in mid-aged women: report of a multicenter South American study. Menopause. 2017;24(11):1282-1288. doi:10.1097/GME.0000000000000924

301. Whiteman MK, Staropoli CA, Langenberg PW, McCarter RJ, Kjerulff KH, Flaws JA. Smoking, body mass, and hot flashes in midlife women. Obstet Gynecol. 2003;101(2):264-272. doi:10.1016/s0029-7844(02)02593-0

302. Ruan X, Cui Y, Du J, Jin F, Mueck AO. Prevalence of climacteric symptoms comparing perimenopausal and postmenopausal Chinese women. J Psychosom Obstet Gynaecol. 2017;38(3):161-169. doi:10.1080/0167482X.2016.1244181

303. An SY, Kim Y, Kwon R, et al. Depressive symptoms and suicidality by menopausal stages among

middle-aged Korean women. Epidemiol Psychiatr Sci. 2022;31:e60. Published 2022 Aug 26. doi:10.1017/S2045796022000439
304. Devi G, Hahn K, Massimi S, Zhivotovskaya E. Prevalence of memory loss complaints and other symptoms associated with the menopause transition: a community survey. Gend Med. 2005;2(4):255-264. doi:10.1016/s1550-8579(05)80055-5
305. Li F, He F, Sun Q, et al. Reproductive history and risk of depressive symptoms in postmenopausal women: A cross-sectional study in eastern China. J Affect Disord. 2019;246:174-181. doi:10.1016/j.jad.2018.12.031
306. Xu J, Bartoces M, Neale AV, Dailey RK, Northrup J, Schwartz KL. Natural history of menopause symptoms in primary care patients: a MetroNet study. J Am Board Fam Pract. 2005;18(5):374-382. doi:10.3122/jabfm.18.5.374
307. Yılmaz S, Arslan I, Yengil Taci D. The effect of physical activity and depressive mood on menopausal symptoms in postmenopausal women. Int J Clin Pract. 2021;75(7):e14247. doi:10.1111/ijcp.14247
308. Islam RM, Bell RJ, Billah B, Hossain MB, Davis SR. Prevalence and severity of vasomotor symptoms and joint pain in women at midlife in Bangladesh: a population-based survey. Menopause. 2016;23(7):731-739. doi:10.1097/GME.0000000000000615
309. Shakhatreh FM, Mas'ad D. Menopausal symptoms and health problems of women aged 50-65 years in southern Jordan. Climacteric. 2006;9(4):305-311. doi:10.1080/13697130600861542
310. Obermeyer CM, Reher D, Saliba M. Symptoms, menopause status, and country differences: a comparative analysis from DAMES. Menopause. 2007;14(4):788-797. doi:10.1097/gme.0b013e318046eb4a
311. Al-Qutob R. Menopause-associated problems: types and magnitude. A study in the Ain Al-Basha area, Jordan. J Adv Nurs. 2001;33(5):613-620. doi:10.1046/j.1365-2648.2001.01706.x
312. Chae HD, Choi SY, Cho EJ, et al. Awareness and experience of menopausal symptom and hormone therapy in korean postmenopausal women. J Menopausal Med. 2014;20(1):7-13. doi:10.6118/jmm.2014.20.1.7
313. McVeigh C. Perimenopause: more than hot flushes and night sweats for some Australian women. J Obstet Gynecol Neonatal Nurs. 2005;34(1):21-27. doi:10.1177/0884217504272801
314. Lampio L, Polo-Kantola P, Polo O, Kauko T, Aittokallio J, Saaresranta T. Sleep in midlife women: effects of menopause, vasomotor symptoms, and depressive symptoms. Menopause. 2014;21(11):1217-1224. doi:10.1097/GME.0000000000000239
315. Oppermann K, Fuchs SC, Donato G, Bastos CA, Spritzer PM. Physical, psychological, and menopause-related symptoms and minor psychiatric disorders in a community-based sample of Brazilian premenopausal, perimenopausal, and postmenopausal women. Menopause. 2012;19(3):355-360. doi:10.1097/gme.0b013e31822ba026
316. An J, Li L. Urban-rural differences in epidemiology and risk factors of menopause syndrome in middle-aged Chinese women. Menopause. 2023;30(3):306-316. doi:10.1097/GME.0000000000002135
317. Kamal NN, Seedhom AE. Quality of life among postmenopausal women in rural Minia, Egypt. East Mediterr Health J. 2017;23(8):527-533. Published 2017 Oct 30.
318. Rajbhandari S, Subedi RK, Dangal G, et al. Menopausal Health Status of Nepalese Women. JNMA J Nepal Med Assoc. 2017;56(205):107-111.

319. Cortés YI, Coslov N, Richardson MK, Woods NF. Symptom experience during the late reproductive stage versus the menopausal transition in the Spanish-language Women Living Better survey. Menopause. 2023;30(3):260-266. doi:10.1097/GME.0000000000002132
320. Baig LA, Karim SA. Age at menopause, and knowledge of and attitudes to menopause, of women in Karachi, Pakistan. J Br Menopause Soc. 2006;12(2):71-74. doi:10.1258/136218006777525721
321. Chalise GD, Shrestha S, Thapa S, Bharati M, Pradhan S, Adhikari B. Health Problems experienced by Peri-menopausal Women and their Perception towards Menopause. J Nepal Health Res Counc. 2022;20(1):102-107. Published 2022 Jun 2. doi:10.33314/jnhrc.v20i01.3891
